# Supplementary material for: Unraveling the Complexity of Chikungunya Virus Infection Immunological and Genetic Insights in Acute and Chronic Patients
Source: Genes (Basel). 2024 Oct 24;15(11):1365. doi: 10.3390/genes15111365 (PMC11593632; doi:10.3390/genes15111365)
Supplement: Supplementary file 1 [file genes-15-01365-s001.zip › genes-3264747-supplementary.pdf]

Supplementary Materials:

Protocol S1: Procedure for extracting whole RNA from whole blood using Trizol LS and Chloroform

Executors: biomedical doctors, biologists, laboratory technicians.

Objective: standardization of the process of total RNA recovery from whole blood collection in a Tempus tube for the preservation of single-stranded genetic material

Materials and equipment required for execution

-Trizol LS

-Chloroform

-Absolute Ethanol (analytical purity)

-DNase and RNase free water

•eppendorff type microtubes 1.5 mL

•1000 µL single-channel micropipette

•200 µL single-channel micropipette

-20 µl single-channel micropipette

•10 µL single-channel micropipette

•1000 uL low retention tips with filter

•200 uL low retention tips with filter

•20 uL low retention tips with filter

•10 uL low retention tips with filter

-Qiagen RNeasy MinElute CleanUp kit

- Chemical exhaust cabin

•biological safety cabin for steps involving whole blood

-Refrigerated eppendorff microtube centrifuge 1.5mL (temperature stabilized at 4ºC)

1. Immediately after the blood sample has been collected and homogenized, add Trizol LS to the sample in a 3:1 ratio (e.g., if 250 µL of blood is obtained, add 750 µL of Trizol LS);
2. If the sample has a volume of less than 250 µL, fill the volume with RNase free water up to 250 µL;
3. Mix the solution by pipetting several times or vortexing briefly to homogenize;
4. Store at -80ºC temperature until shipment or further processing.

Processing Whole Blood/Trizol LS Samples for RNA Isolation

1. Thaw the sample and homogenize again with Trizol LS lysate by vortex;
2. Incubate for 5 minutes at room temperature (TA) and then spin the liquid down from the tube cap;
3. Process 1mL of blood lysate/Trizol LS (1:4 vol:vol – blood:trizol);
4. Add 200 µL of chloroform (1:5 chloroform:Trizol) and vortex the sample for 15 seconds;
5. Incubate the sample at room temperature (TA) for 10 minutes;
6. Centrifuge at 12,000 g for 15 minutes in a centrifuge refrigerated at 4ºC;
7. Transfer the aqueous phase to a new 1.5 mL eppendorff microtube;
8. Add 1.5 volumes of absolute ethanol to the aqueous phase, mix well by pipetting, DO NOT use vortex in this step;
9. Proceed to the RNeasy MinElute CleanUp kit purification and concentration protocol.

Purifying and concentrating the RNA extracted by Trizol-Chloroform using Qiagen RNeasy MinElute CleanUp kit

Steps to take before execution

-Add 4 volumes of ethanol (96-100%) to the concentrated RPE Buffer/Buffer as indicated on the bottle, in order to obtain the working solution.

•RLT buffer/buffer may show precipitates after storage. If necessary, resuspend the precipitate by heating (e.g., water bath at 37ºC) and then maintain the room temperature (15-25ºC).

1. Adjust the sample volume to 100 µL or 200 µL using RNase-free water. Add 350uL (if the initial volume is 100 µL) or 700 µL (if the initial volume is 200 µL) of RLT Buffer. Mix well;

2. Add 250 µL or 500 µL of 96-100% ethanol to dilute the RNA. Mix well by pipetting. DO NOT spin and proceed immediately to the next step;
3. Transfer the 700 µL sample to the RNeasy MinElute spin column attached to a 2 mL collection tube. Close the lid gently;
4. Spin for 15 seconds at ≥ 8,000 g (or ≥ 10,000 rpm). Discard the eluded. If the sample volume is greater than 700 µL, repeat steps 3 and 4;
5. Place the column in a new 2 mL collection tube. Add 500 µL of RPE buffer to the column. Gently close the lid;
6. Centrifuge for 15 seconds at ≥ 8,000 g (or ≥ 10,000 rpm) to flush the column. Discard the eluate and reuse the collection tube for the next step;
7. Add 500 µL of 80% Ethanol to the column. Close the lid gently and spin for 2 minutes at ≥ 8,000 g (or ≥ 10,000 rpm) to flush the column membrane;
8. Place the column in a new 2 mL collection tube. Open the column cover and spin at maximum speed for 5 minutes. Discard the elurate and the collection tube.

   To prevent damage to the lids, place the columns in the centrifuge with at least one empty position between the columns. Orient the eyelids so that they point in the opposite direction of the rotor rotation (e.g., if the rotor rotates clockwise, orient the caps counterclockwise).

   It is important to dry the column membrane, as the residual ethanol can interfere with the following reactions. Centrifugation with the lids open ensures that no ethanol is transported during RNA elution.
9. Place the RNeasy MinElute column in a new 1.5 ml collection tube (eppendorff type). Add 14 µl of RNase-free water directly to the center of the column membrane. Close the lid gently and centrifuge for 1 min at full speed to elute the RNA.

   Do not elute with less than 10 µL of RNase-free water, as the column membrane will not be sufficiently hydrated. The dead volume of the RNeasy MinElute rotating column is 2 µl: elution with 14 µl of RNase-free water results in an eluate of 12 µl.

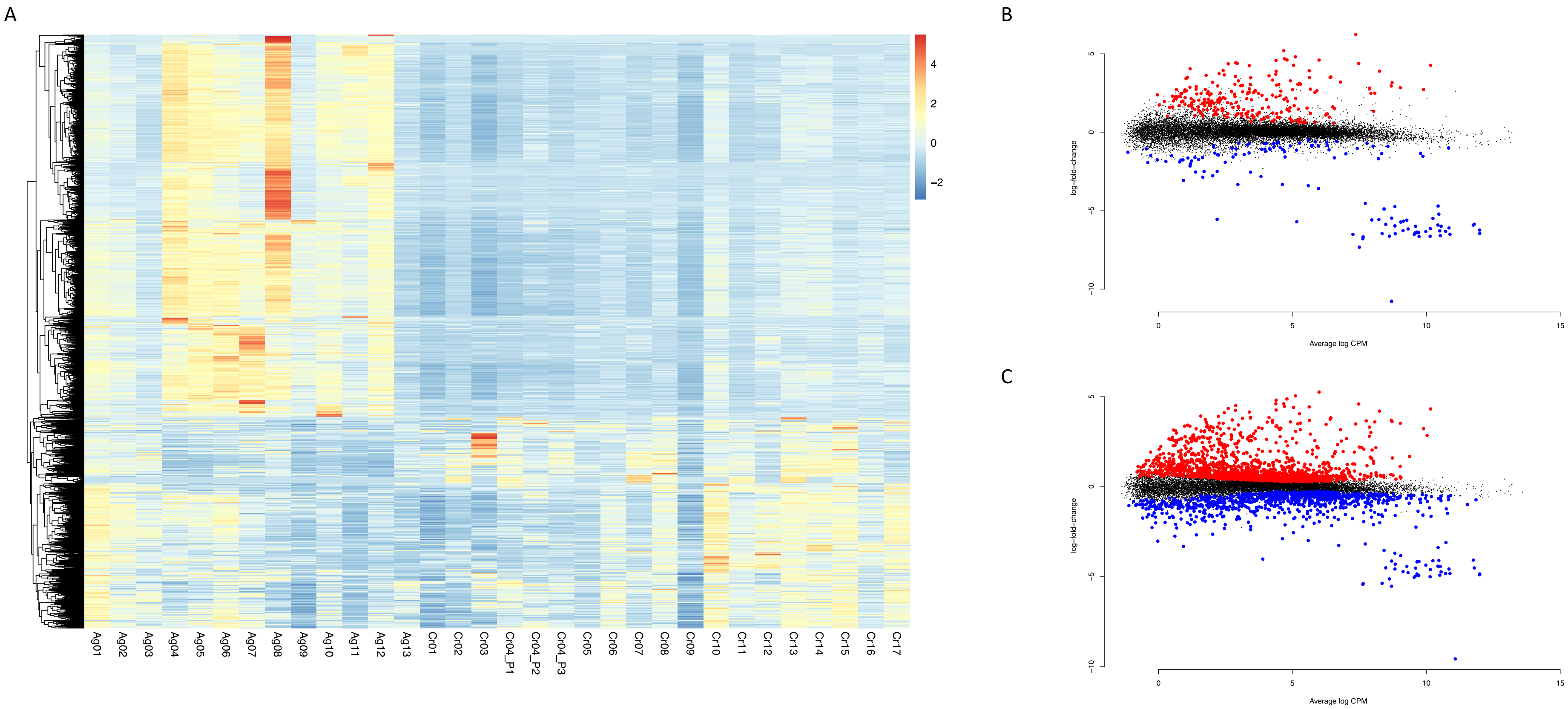

Figure S1: Differential gene expression among acute and chronic CHIKV individuals. A. Heatmap obtained from differentially expressed host transcripts between groups consisting of samples from acute and chronic individuals. Hierarchical clustering was applied to samples from the two clinical statuses (represented on the X-axis) and transcripts (Y-axis). B. Volcano plot of differentially expressed host genes between acute phase and control group samples, with average log CPM on the Y-axis and the log-fold change for each transcript on the Y-axis. Transcripts with p-value < 0.05 and Log fold-change > |1| are coloured red. C. Volcano plot of differentially expressed host genes between acute phase and chronic group

samples, with average log CPM on the Y-axis and the log-fold change for each transcript on the Y-axis. Transcripts with p-value < 0.05 and Log fold-change > |1| are coloured red.

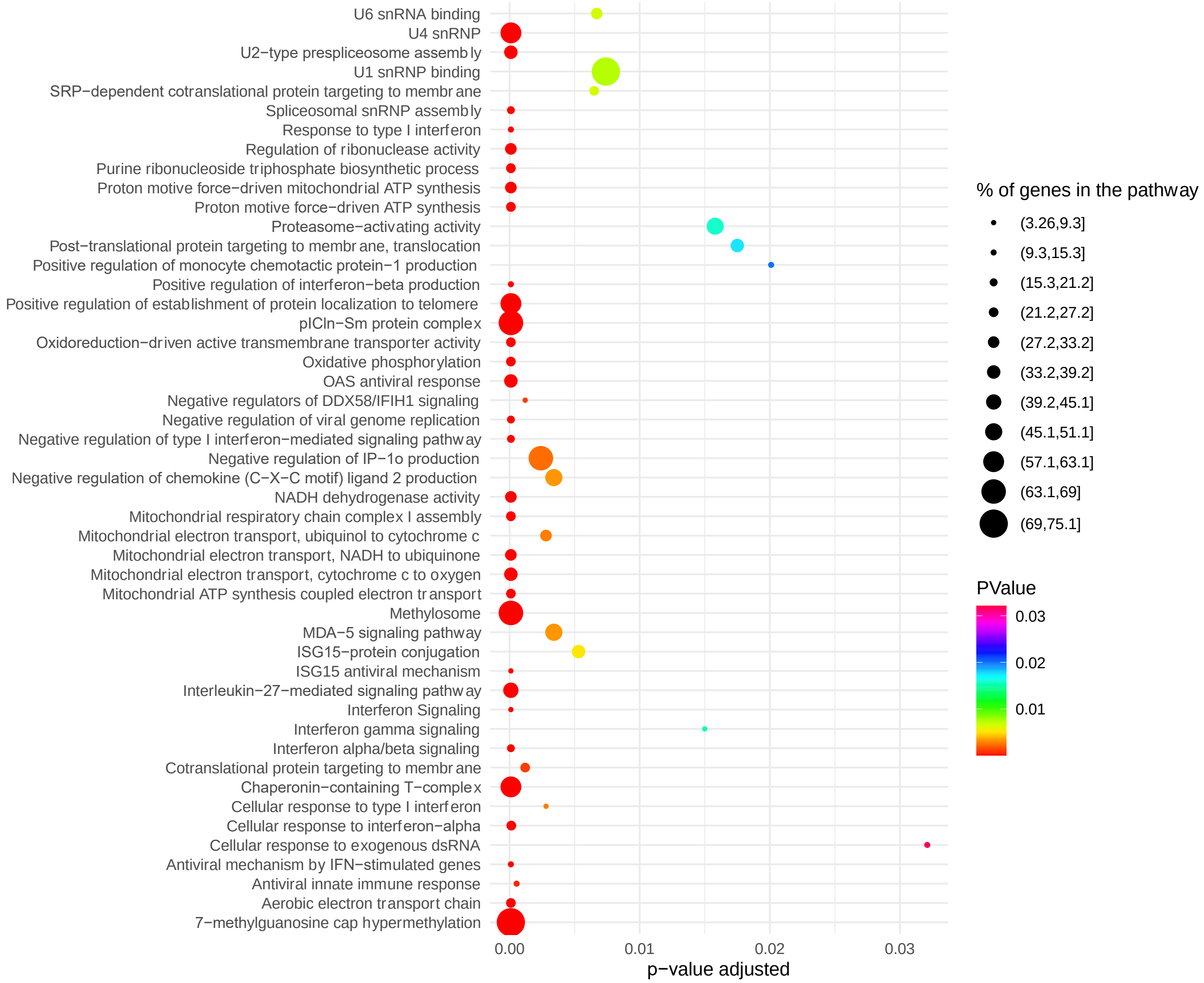

Figure S2: Functional enrichments pathways for the genes positively correlated with CHIKV acute patients.

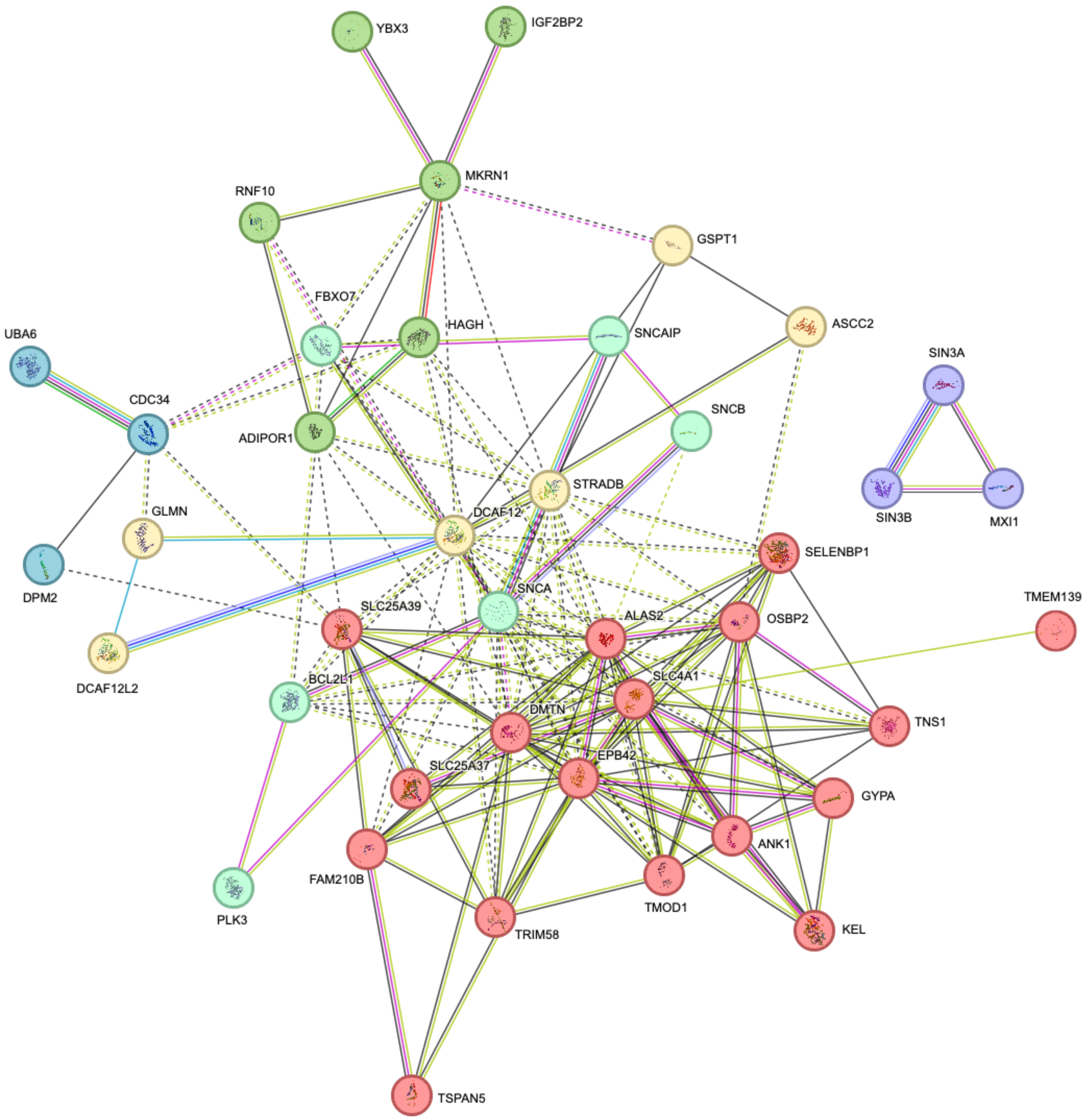

Figure S3: Network of genes components of Module 21, negatively correlated with acute individuals and positively correlated with CHIKV chronic patients.

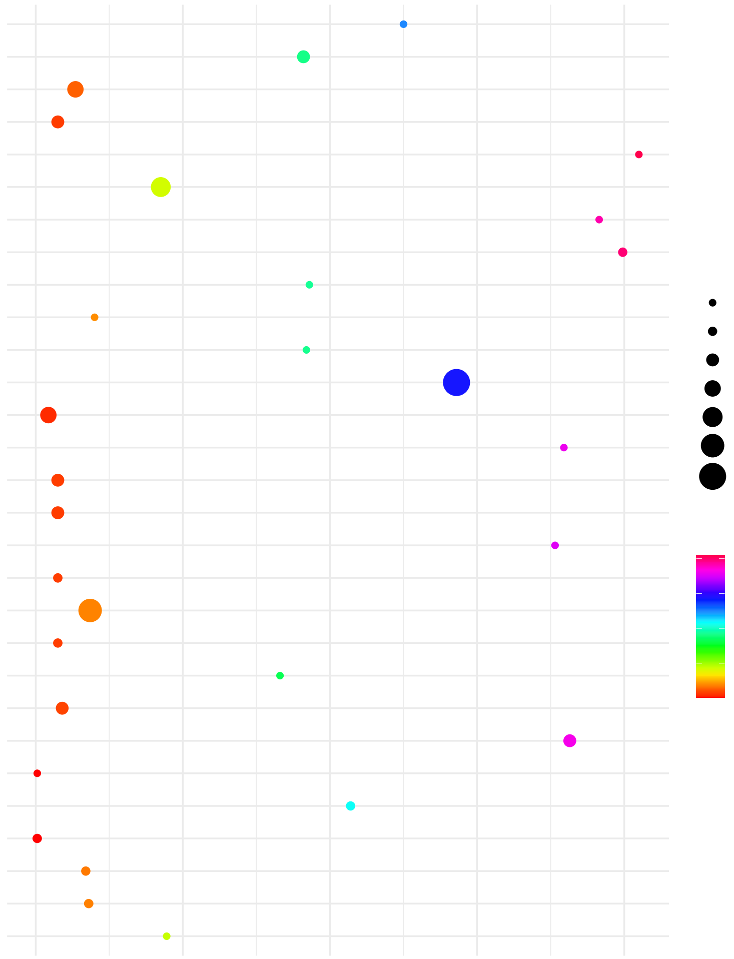

Figure S4: Functional enrichments pathways for the genes positively correlated with CHIKV chronic patients.;

Table S1: Drug and complementary therapies among CHIKV-chronic and acute individuals.;

Supplementary Table S1: Drug and complementary therapies among CHIKV-chronic and acute individuals.

	Chronic (n=19)	Acute (n=13)
**Drug therapy**		
Amitriptyline	8 (42.4%)	0 (0%)
Pregabalin	6 (31.8%)	0 (0%)
Codeine	4 (21.2%)	0 (0%)
Prednisolone	4 (21.2%)	1 (7.52%)
Cyclobenzaprine	1 (5.3%)	0 (0%)
**Complementary therapies**		
Physical activity	1 (5.3%)	0 (0%)
Acupuncture	4 (21.2%)	0 (0%)

Table S2: Metrics and Sequencing statistics regarding the reads length, reads counting, percentage of mapping and genes count for all study individuals.

Supplementary Table S2: Metrics and Sequencing statistics regarding the reads lenght, reads counting, percentage of mapping and genes count for all study individuals.

Sample	Read Length	Reads Forward	Reads Reverse	%Mapped Reads	Count Genes
Ag1	256	33902215	33902215	82.11	78022569
Ag2	254	28038807	28038807	81.07	65645008
Ag3	261	23500635	23500635	80.94	56920564
Ag4	249	24531112	24531112	90.11	51283181
Ag5	251	24807647	24807647	90.92	54212860
Ag6	249	31405715	31405715	89.3	64409066
Ag7	251	27629693	27629693	86.48	64930336
Ag8	252	30004248	30004248	83.46	72373970
Ag9	250	16173620	16173620	87.90	35991128
Ag10	260	23419856	23419856	84.84	56123335
Ag11	253	19758943	19758943	83.70	43723387
Ag12	257	23307806	23307806	81.77	51948299
Ag13	252	29294954	29294954	73.88	68775670
Cr1	257	19526041	19526041	71.75	47984705
Cr2	262	26029870	26029870	69.73	72182338
Cr3	266	21355610	21355610	76.3	53917885
Cr4P1	261	29100821	29100821	73.44	74766093
Cr4P2	259	27081830	27081830	75.91	63748047
Cr4P3	273	25344606	25344606	75.3	64931420
Cr5	264	20703691	20703691	75.8	53011549
Cr6	261	23374312	23374312	82.85	54275766
Cr7	261	27441735	27441735	71.9	72952301
Cr8	260	29113698	29113698	76.37	73480368
Cr9	269	11927140	11927140	76.02	29780355
Cr10	257	29161124	29161124	81.89	69974982

Cr11	273	25048801	25048801	72.69	69148033
Cr12	259	26029793	26029793	72.84	57988448
Cr13	250	33996343	33996343	75.33	91520491
Cr14	263	27966161	27966161	80.47	72949946
Cr15	263	33740267	33740267	68.92	82072286
Cr16	265	24564919	24564919	76.22	65994479
Cr17	272	21323567	21323567	84.46	56432252
Cont1	263	25892502	25892502	65.92	77292393
Cont2	264	24187139	24187139	74.68	55156880
Cont4	264	41527637	41527637	76.1	123637494
Cont5	255	30789660	30789660	82.97	74958309
Cont6	263	26566743	26566743	81.2	65648421

Table S3: Differentially expressed genes found comparing chronic individuals and control group.

Supplementary Table S3: Differentially expressed genes found comparing chronic individuals and control group.

Gene	logFC	logCPM	LR	PValue	FDR
DTL	3.149	1.903	100.452	<0.0001	<0.0001
RRM2	3.568	4.253	95.368	<0.0001	<0.0001
TOP2A	2.551	3.464	87.874	<0.0001	<0.0001
SHCBP1	3.204	2.303	86.667	<0.0001	<0.0001
IGLV3-25	5.040	5.110	84.921	<0.0001	<0.0001
IGHV2-5	4.252	4.390	84.878	<0.0001	<0.0001
MCM4	1.958	3.879	83.445	<0.0001	<0.0001
ASPM	2.846	2.337	83.245	<0.0001	<0.0001
MZB1	4.044	5.262	81.513	<0.0001	<0.0001
CD38	2.265	4.817	80.389	<0.0001	<0.0001
BUB1	3.176	2.829	79.107	<0.0001	<0.0001
IGHV5-51	5.246	5.993	78.594	<0.0001	<0.0001
MKI67	2.506	4.759	77.446	<0.0001	<0.0001
TXNDC5	3.300	7.874	75.731	<0.0001	<0.0001
EXO1	2.970	0.662	74.665	<0.0001	<0.0001
IGLC3	3.589	8.074	74.613	<0.0001	<0.0001
KNL1	2.240	1.945	72.837	<0.0001	<0.0001
IGHV4-61	3.832	4.125	72.616	<0.0001	<0.0001
OTOF	4.343	2.601	71.913	<0.0001	<0.0001
IGHV3-21	3.375	4.407	71.773	<0.0001	<0.0001
CDC6	3.269	1.854	71.303	<0.0001	<0.0001
IGKV1-12	3.803	3.428	71.110	<0.0001	<0.0001
CCNB2	3.088	1.721	70.794	<0.0001	<0.0001
TPX2	2.619	2.710	70.543	<0.0001	<0.0001
NCAPG	2.622	2.282	70.437	<0.0001	<0.0001
IGLV3-1	4.585	7.473	70.144	<0.0001	<0.0001

IGLV2-23	3.487	5.200	69.183	<0.0001	<0.0001
IGKV4-1	3.742	6.406	68.849	<0.0001	<0.0001
GLDC	3.722	1.746	67.993	<0.0001	<0.0001
PCLAF	3.043	1.717	67.941	<0.0001	<0.0001
MYBL2	3.232	3.864	67.320	<0.0001	<0.0001
ORC1	2.495	1.221	67.315	<0.0001	<0.0001
IGHG3	2.554	5.836	67.293	<0.0001	<0.0001
BHLHA15	4.100	2.455	67.158	<0.0001	<0.0001
SPAG5	2.278	2.576	67.102	<0.0001	<0.0001
IGHV4-59	4.424	5.625	67.097	<0.0001	<0.0001
IGHV3-48	3.676	4.257	66.182	<0.0001	<0.0001
IGLV7-46	4.598	4.378	66.176	<0.0001	<0.0001
IGHV1-69	4.799	4.389	65.720	<0.0001	<0.0001
CDT1	2.759	1.633	65.569	<0.0001	<0.0001
TNFRSF17	3.792	3.132	65.166	<0.0001	<0.0001
IGHM	4.310	10.158	64.799	<0.0001	<0.0001
IGHV3-33	3.524	5.299	64.427	<0.0001	<0.0001
DLGAP5	3.336	1.961	64.247	<0.0001	<0.0001
CCNA2	2.636	2.212	63.911	<0.0001	<0.0001
CEP55	2.741	1.696	63.902	<0.0001	<0.0001
IGKV2D-28	4.393	5.041	63.733	<0.0001	<0.0001
IGKJ1	3.007	7.929	63.119	<0.0001	<0.0001
JCHAIN	3.681	8.689	63.024	<0.0001	<0.0001
IGKV3-15	3.067	5.117	62.901	<0.0001	<0.0001
GTSE1	2.626	1.324	62.871	<0.0001	<0.0001
IGHV1-18	2.937	3.553	62.437	<0.0001	<0.0001
IGHV1-69D	4.458	4.698	62.388	<0.0001	<0.0001
IGKV3D-15	3.265	3.735	62.364	<0.0001	<0.0001
IGHV3-30	3.680	5.633	62.153	<0.0001	<0.0001
IGKV1-27	4.865	4.794	62.087	<0.0001	<0.0001

IGHV4-39	3.246	4.490	61.456	<0.0001	<0.0001
DERL3	3.430	1.816	60.587	<0.0001	<0.0001
PLK1	2.676	1.826	60.482	<0.0001	<0.0001
IGHJ6	3.516	9.028	60.195	<0.0001	<0.0001
BIRC5	3.257	2.044	60.068	<0.0001	<0.0001
IGLV3-10	4.233	2.869	60.010	<0.0001	<0.0001
CCNB1	2.443	2.261	59.976	<0.0001	<0.0001
IGHJ5	4.192	8.246	59.905	<0.0001	<0.0001
IGLV3-19	2.778	3.399	59.044	<0.0001	<0.0001
ZWINT	2.342	2.285	58.897	<0.0001	<0.0001
IGKV1D-33	2.735	3.140	58.874	<0.0001	<0.0001
IGKV1D-39	3.590	4.923	58.819	<0.0001	<0.0001
IGLV3-9	4.500	2.881	58.810	<0.0001	<0.0001
SPATS2L	2.623	4.034	58.572	<0.0001	<0.0001
HMMR	3.082	1.588	57.946	<0.0001	<0.0001
KIFC1	2.722	1.938	57.847	<0.0001	<0.0001
IGKV1-33	2.722	3.344	57.772	<0.0001	<0.0001
KIF2C	2.744	1.505	57.640	<0.0001	<0.0001
UHRF1	1.925	2.371	57.396	<0.0001	<0.0001
FOXM1	2.469	1.893	57.380	<0.0001	<0.0001
IGKC	3.214	9.896	56.497	<0.0001	<0.0001
IGLC2	3.604	8.720	55.678	<0.0001	<0.0001
KIF4A	2.781	0.992	55.483	<0.0001	<0.0001
TCN2	1.828	3.176	55.063	<0.0001	<0.0001
FAM111B	2.266	2.064	54.604	<0.0001	<0.0001
IGLV1-51	3.243	4.565	54.363	<0.0001	<0.0001
CDCA5	2.771	1.667	54.045	<0.0001	<0.0001
CDK1	2.699	1.533	53.849	<0.0001	<0.0001
IGLV6-57	4.601	4.675	53.846	<0.0001	<0.0001
IGLC6	3.358	1.171	52.891	<0.0001	<0.0001

IGLV7-43	4.135	3.388	52.874	<0.0001	<0.0001
IGHV4-4	3.700	2.748	52.122	<0.0001	<0.0001
KIF11	1.906	2.782	52.056	<0.0001	<0.0001
CENPM	2.561	0.624	51.972	<0.0001	<0.0001
IGKV1-17	2.847	3.067	51.78	<0.0001	<0.0001
EPHB2	2.023	2.483	51.751	<0.0001	<0.0001
IGLV1-44	3.818	6.432	51.483	<0.0001	<0.0001
GINS2	2.590	1.324	51.263	<0.0001	<0.0001
CDC20	3.315	1.93	51.189	<0.0001	<0.0001
ITM2C	2.152	5.953	51.076	<0.0001	<0.0001
CLSPN	1.970	1.828	50.953	<0.0001	<0.0001
RTP4	1.957	4.169	50.925	<0.0001	<0.0001
IGKV3-20	2.671	5.002	50.619	<0.0001	<0.0001
CHEK1	2.123	1.707	50.432	<0.0001	<0.0001
CDC25A	3.357	0.972	50.42	<0.0001	<0.0001
DEPDC1B	2.738	0.514	50.283	<0.0001	<0.0001
IGHV4-34	3.084	3.624	50.271	<0.0001	<0.0001
NT5DC2	2.174	2.416	50.156	<0.0001	<0.0001
RAD51	2.254	0.985	50.056	<0.0001	<0.0001
KLHL14	2.367	2.854	49.573	<0.0001	<0.0001
CENPF	1.825	2.773	48.921	<0.0001	<0.0001
IGHV3-7	2.770	4.541	48.737	<0.0001	<0.0001
IGKV1-16	2.956	3.04	48.657	<0.0001	<0.0001
IGHV3-43	3.905	2.236	48.584	<0.0001	<0.0001
ESCO2	2.605	0.46	48.397	<0.0001	<0.0001
IGKV1D-12	3.324	1.797	48.168	<0.0001	<0.0001
HJURP	2.868	1.306	48.125	<0.0001	<0.0001
PCNA	1.118	4.871	48.081	<0.0001	<0.0001
IGLL5	3.454	6.257	47.985	<0.0001	<0.0001
IRF7	1.672	6.506	47.119	<0.0001	<0.0001

IGHV1-46	2.812	2.722	46.764	<0.0001	<0.0001
IGLV1-36	3.579	1.777	46.63	<0.0001	<0.0001
IGHV3-11	3.579	4.056	46.618	<0.0001	<0.0001
TTK	2.666	0.835	46.316	<0.0001	<0.0001
FABP5	1.753	2.818	46.286	<0.0001	<0.0001
GMNN	1.563	2.135	46.267	<0.0001	<0.0001
STIL	1.794	1.239	46.031	<0.0001	<0.0001
IGHV3-66	3.062	2.018	45.951	<0.0001	<0.0001
IGHJ4	2.839	10.026	45.922	<0.0001	<0.0001
IGKV3D-11	2.765	1.322	45.474	<0.0001	<0.0001
IGKV3D-20	2.99	3.067	45.39	<0.0001	<0.0001
KIF23	1.78	1.502	45.371	<0.0001	<0.0001
MELK	2.647	0.864	44.981	<0.0001	<0.0001
BUB1B	2.639	1.427	44.812	<0.0001	<0.0001
EAF2	1.927	2.97	44.714	<0.0001	<0.0001
FKBP11	1.464	4.414	44.55	<0.0001	<0.0001
NCAPH	1.833	1.493	44.464	<0.0001	<0.0001
CDKN3	2.415	1.106	44.355	<0.0001	<0.0001
POLE2	2.075	0.023	44.152	<0.0001	<0.0001
IGLV8-61	3.846	3.44	43.624	<0.0001	<0.0001
FEN1	1.441	3.694	43.597	<0.0001	<0.0001
TYMS	2.943	2.175	43.596	<0.0001	<0.0001
UBE2C	1.961	1.618	43.548	<0.0001	<0.0001
SEC11C	1.928	5.545	43.512	<0.0001	<0.0001
KIF15	2.657	1.231	43.425	<0.0001	<0.0001
ESPL1	2.598	0.758	43.389	<0.0001	<0.0001
KPNA2	1.193	4.285	43.357	<0.0001	<0.0001
UBE2T	1.937	1.103	43.301	<0.0001	<0.0001
IGLV2-18	3.312	2.208	43.199	<0.0001	<0.0001
MCM2	1.463	3.722	43.19	<0.0001	<0.0001

SIGLEC1	3.16	5.293	42.663	<0.0001	<0.0001
OASL	1.833	6.57	42.45	<0.0001	<0.0001
MTHFD2	1.002	4.904	42.429	<0.0001	<0.0001
IGHV3-23	2.912	5.67	42.085	<0.0001	<0.0001
IGKV1-9	2.808	3.424	42.056	<0.0001	<0.0001
SKA1	2.536	0.613	41.969	<0.0001	<0.0001
GPRC5D	3.41	1.082	41.791	<0.0001	<0.0001
STMN1	1.442	4.925	41.466	<0.0001	<0.0001
FANCI	1.154	3.496	41.449	<0.0001	<0.0001
IGHV3-69-1	3.207	1.402	41.302	<0.0001	<0.0001
OAS1	2.191	7.494	41.267	<0.0001	<0.0001
IGLV2-11	2.43	4.387	40.908	<0.0001	<0.0001
MMACHC	2.183	2.926	40.805	<0.0001	<0.0001
MCM6	1.07	4.674	40.786	<0.0001	<0.0001
AURKA	1.751	1.816	40.636	<0.0001	<0.0001
GGH	2.055	1.99	40.554	<0.0001	<0.0001
PLAC8	1.129	6.363	40.441	<0.0001	<0.0001
CAV1	2.52	1.041	40.16	<0.0001	<0.0001
CENPN	1.356	2.273	39.958	<0.0001	<0.0001
SLC1A4	1.334	3.968	39.587	<0.0001	<0.0001
IGHV3-20	3.513	2.413	39.452	<0.0001	<0.0001
CHPF	1.747	2.807	39.346	<0.0001	<0.0001
PKMYT1	2.441	0.676	39.243	<0.0001	<0.0001
RACGAP1	1.092	3.106	39.189	<0.0001	<0.0001
IGLV5-45	2.762	0.992	39.165	<0.0001	<0.0001
TXNDC11	1.248	5.462	39.117	<0.0001	<0.0001
MIR3652	2.793	7.871	39.018	<0.0001	<0.0001
CENPE	1.622	1.975	38.823	<0.0001	<0.0001
TMOD1	-2.128	4.724	38.787	<0.0001	<0.0001
IGHV6-1	3.239	2.626	38.705	<0.0001	<0.0001

PLK4	1.55	1.725	38.688	<0.0001	<0.0001
AURKB	2.475	1.075	38.667	<0.0001	<0.0001
NUF2	1.961	1.135	38.555	<0.0001	<0.0001
TESC	-1.718	6.567	38.514	<0.0001	<0.0001
IGLV4-69	2.934	2.441	38.475	<0.0001	<0.0001
IGHV3-49	3.421	2.984	38.462	<0.0001	<0.0001
NME4	-4.437	9.271	38.389	<0.0001	<0.0001
LY6E	2.159	8.114	38.335	<0.0001	<0.0001
IGLC7	2.666	2.727	38.229	<0.0001	<0.0001
SPATS2	1.588	2.418	38.156	<0.0001	<0.0001
CHAF1B	1.365	1.19	38.11	<0.0001	<0.0001
USP18	2.638	3.32	38.058	<0.0001	<0.0001
CENPU	1.579	2.083	37.885	<0.0001	<0.0001
IGKJ5	2.146	5.95	37.196	<0.0001	<0.0001
GINS1	2.285	0.617	37.015	<0.0001	<0.0001
JUP	1.621	4.797	36.945	<0.0001	<0.0001
APOBEC3G	0.748	6.258	36.941	<0.0001	<0.0001
LNCRNA-IUR	-1.6	3.629	36.939	<0.0001	<0.0001
TIMELESS	1.051	3.28	36.888	<0.0001	<0.0001
POU2AF1	1.83	4.776	36.867	<0.0001	<0.0001
NME1	1.657	3.427	36.855	<0.0001	<0.0001
TP53INP2	-1.301	4.149	36.823	<0.0001	<0.0001
ISG15	2.527	6.822	36.756	<0.0001	<0.0001
MIR23AHG	-1.261	4.117	36.741	<0.0001	<0.0001
RNASE1	2.681	1.178	36.637	<0.0001	<0.0001
TLR7	1.08	4.802	36.597	<0.0001	<0.0001
IGHA1	2.661	7.02	36.552	<0.0001	<0.0001
FBXO5	1.104	2.567	36.437	<0.0001	<0.0001
PRDX4	1.339	3.474	36.329	<0.0001	<0.0001
HERC6	1.394	4.874	36.325	<0.0001	<0.0001

PDIA4	1.341	6.301	36.097	<0.0001	<0.0001
FAM234A	-4.612	10.147	35.979	<0.0001	<0.0001
IGLV9-49	3.915	1.576	35.889	<0.0001	<0.0001
IGHV1-2	2.652	3.605	35.711	<0.0001	<0.0001
AK1	-2.398	3.439	35.596	<0.0001	<0.0001
RAD51AP1	1.764	0.529	35.452	<0.0001	<0.0001
AXIN1	-4.524	11.789	35.421	<0.0001	<0.0001
METTL26	-4.139	8.704	35.413	<0.0001	<0.0001
IGHV4-31	3.527	2.261	35.381	<0.0001	<0.0001
PSMA8	1.123	2.103	35.344	<0.0001	<0.0001
RELL1	-1.284	4.624	35.281	<0.0001	<0.0001
RHOT2	-4.125	10.243	35.248	<0.0001	<0.0001
TCF19	1.071	3.323	35.141	<0.0001	<0.0001
TK1	2.453	2.285	35.136	<0.0001	<0.0001
CHAC2	1.753	1.38	35.13	<0.0001	<0.0001
FABP5P7	1.913	-0.294	34.957	<0.0001	<0.0001
SDF2L1	1.484	3.614	34.894	<0.0001	<0.0001
NDC80	1.393	2.379	34.889	<0.0001	<0.0001
SGIP1	-3.035	-0.028	34.628	<0.0001	<0.0001
UAP1	1.324	4.262	34.614	<0.0001	<0.0001
PSMC2	0.789	5.366	34.518	<0.0001	<0.0001
CDCA7	1.457	1.481	34.515	<0.0001	<0.0001
SNORD17	2.413	2.941	34.513	<0.0001	<0.0001
LMAN1	1.211	5.67	34.504	<0.0001	<0.0001
SLAMF7	1.1	6.372	34.461	<0.0001	<0.0001
IGHV3-53	2.873	2.715	34.428	<0.0001	<0.0001
PGAP6	-3.392	10.436	34.29	<0.0001	<0.0001
DYNC2I2	1.33	2.036	34.147	<0.0001	<0.0001
BBOF1	-1.732	4.06	34.047	<0.0001	<0.0001
WDR24	-4.53	9.597	33.993	<0.0001	<0.0001

EPSTI1	1.939	6.601	33.967	<0.0001	<0.0001
STUB1	-3.546	8.42	33.958	<0.0001	<0.0001
JMJD8	-3.702	8.822	33.848	<0.0001	<0.0001
IGHG1	3.159	6.795	33.782	<0.0001	<0.0001
ATAD2	0.86	4.081	33.774	<0.0001	<0.0001
OAS2	1.636	7.904	33.754	<0.0001	<0.0001
IGKV1-39	3.172	4.521	33.727	<0.0001	<0.0001
FBXL16	-5.014	10.14	33.327	<0.0001	<0.0001
CKS2	1.359	2.428	33.17	<0.0001	<0.0001
DHCR24	1.292	3.265	33.144	<0.0001	<0.0001
CENPW	1.813	0.836	33.094	<0.0001	<0.0001
RPL22L1	1.019	3.53	33.044	<0.0001	<0.0001
PSME2P1	1.379	0.462	32.987	<0.0001	<0.0001
GCH1	0.726	5.307	32.925	<0.0001	<0.0001
CAPN15	-4.107	10.466	32.881	<0.0001	<0.0001
PYCR1	3.091	0.644	32.821	<0.0001	<0.0001
TRIM69	0.85	6.35	32.818	<0.0001	<0.0001
KIF14	2.541	0.668	32.762	<0.0001	<0.0001
RAB11FIP3	-4.744	10.549	32.455	<0.0001	<0.0001
SCD	1.12	2.722	32.436	<0.0001	<0.0001
CNP	0.793	5.479	32.309	<0.0001	<0.0001
DNAJB11	1.097	5.435	32.259	<0.0001	<0.0001
C9orf78	-1.572	8.33	32.148	<0.0001	<0.0001
GINS4	1.453	1.087	32.08	<0.0001	<0.0001
RPS6KA5	-0.951	4.941	32.079	<0.0001	<0.0001
PTTG1	1.69	2.81	32.076	<0.0001	<0.0001
KIF20A	3.143	0.565	32.023	<0.0001	<0.0001
MYDGF	1.323	4.757	32.003	<0.0001	<0.0001
ANLN	2.024	0.838	32.001	<0.0001	<0.0001
IGHJ2P	2.323	6.685	31.987	<0.0001	<0.0001

MRPL28	-4.515	8.485	31.904	<0.0001	<0.0001
IGHV3-74	2.543	3.393	31.875	<0.0001	<0.0001
TROAP	2.647	0.388	31.871	<0.0001	<0.0001
EZH2	1.124	2.967	31.679	<0.0001	<0.0001
ZWILCH	0.993	2.513	31.659	<0.0001	<0.0001
HELLS	1.382	1.714	31.566	<0.0001	<0.0001
ATP5MC3	0.813	5.499	31.45	<0.0001	<0.0001
SPC24	2.224	0.084	31.383	<0.0001	<0.0001
ANTKMT	-4.581	8.826	31.264	<0.0001	<0.0001
ARHGAP42	1.743	0.992	31.086	<0.0001	<0.0001
HBQ1	-3.191	7.718	31.084	<0.0001	<0.0001
OR2W3	-2.233	5.388	31.05	<0.0001	<0.0001
ELL2	1.467	4.907	30.909	<0.0001	<0.0001
XBP1	1.247	7.328	30.897	<0.0001	<0.0001
PDIA5	1.428	2.415	30.735	<0.0001	<0.0001
FAM72B	1.554	-0.035	30.611	<0.0001	<0.0001
ASCC2	-1.454	7.654	30.521	<0.0001	<0.0001
CCNE2	1.588	0.854	30.481	<0.0001	<0.0001
NCAPG2	1.048	3.071	30.354	<0.0001	<0.0001
TPRG1-AS1	1.714	0.529	30.263	<0.0001	<0.0001
CIAO3	-4.835	10.718	30.234	<0.0001	<0.0001
C5AR2	-0.933	5.721	30.137	<0.0001	<0.0001
PPIB	1.257	7.257	29.981	<0.0001	<0.0001
GPR146	-1.468	4.705	29.954	<0.0001	<0.0001
MIR3176	-4.835	10.88	29.93	<0.0001	<0.0001
MCRIP2	-4.925	10.501	29.881	<0.0001	<0.0001
TRAM2	1.137	4.582	29.851	<0.0001	<0.0001
RECQL4	1.407	1.263	29.796	<0.0001	<0.0001
SGO2	1.13	2.122	29.776	<0.0001	<0.0001
PIGQ	-4.583	10.843	29.502	<0.0001	<0.0001

WDHD1	1.125	2.17	29.494	<0.0001	<0.0001
MT2A	1.389	4.515	29.363	<0.0001	<0.0001
CKAP2L	2.582	0.559	29.312	<0.0001	<0.0001
ENSG00000239920	-9.575	11.078	29.295	<0.0001	<0.0001
LUC7L	-4.903	11.986	29.278	<0.0001	<0.0001
INTS7	0.698	3.457	29.069	<0.0001	<0.0001
RAB40C	-4.857	11.991	29.026	<0.0001	<0.0001
SMC2	0.87	3.933	29.006	<0.0001	<0.0001
MCM7	0.9	5.36	28.949	<0.0001	<0.0001
POC1A	1.452	0.896	28.898	<0.0001	<0.0001
ZBTB32	1.779	1.238	28.774	<0.0001	<0.0001
RRM1	0.891	4.67	28.594	<0.0001	<0.0001
TARS1	0.683	5.432	28.482	<0.0001	<0.0001
CALU	0.814	5.012	28.389	<0.0001	<0.0001
TMEM258	0.932	4.729	28.387	<0.0001	<0.0001
HMGB3	1.672	1.64	28.366	<0.0001	<0.0001
SDC2	-1.968	1.357	28.22	<0.0001	<0.0001
LGALS3BP	1.434	5.031	28.215	<0.0001	<0.0001
GBP1P1	1.921	2.218	28.163	<0.0001	<0.0001
ORC6	1.593	0.217	28.112	<0.0001	<0.0001
HAGH	-1.385	6.316	28.092	<0.0001	<0.0001
TMEM106C	0.832	3.83	28.048	<0.0001	<0.0001
KIF18A	1.559	0.415	27.912	<0.0001	<0.0001
BRCA2	1.279	2.066	27.881	<0.0001	<0.0001
BCCIP	0.709	4.496	27.875	<0.0001	<0.0001
PRC1	2.263	0.55	27.85	<0.0001	<0.0001
MRPL13	0.809	3.412	27.841	<0.0001	<0.0001
TIMM17A	0.807	4.254	27.813	<0.0001	<0.0001
ADA	0.75	4.317	27.798	<0.0001	<0.0001
BLZF1	0.716	4.314	27.795	<0.0001	<0.0001

IGLC1	3.649	7.757	27.584	<0.0001	<0.0001
METRN	-4.952	9.713	27.576	<0.0001	<0.0001
ENSG00000286129	1.924	4.683	27.539	<0.0001	<0.0001
MANEA	1.117	3.763	27.514	<0.0001	<0.0001
OSBP2	-2.038	5.808	27.512	<0.0001	<0.0001
B4GALT2	1.478	1.434	27.497	<0.0001	<0.0001
DUSP5	1.172	4.135	27.492	<0.0001	<0.0001
IRF4	1.324	5.217	27.458	<0.0001	<0.0001
BAG1	-1.555	8.208	27.396	<0.0001	<0.0001
GFUS	-1.297	5.931	27.387	<0.0001	<0.0001
CASP10	0.643	5.222	27.311	<0.0001	<0.0001
IGHV1-24	2.543	1.407	27.21	<0.0001	<0.0001
TNFRSF13B	1.709	2.353	27.163	<0.0001	<0.0001
SAMD9L	1.294	8.03	27.099	<0.0001	<0.0001
IGLV2-14	2.23	5.91	27.075	<0.0001	<0.0001
DCLRE1A	1.042	3.286	27.006	<0.0001	<0.0001
NDUFA7	0.866	3.695	26.984	<0.0001	<0.0001
CDCA8	1.373	1.575	26.919	<0.0001	<0.0001
GBP3	1.208	5.065	26.791	<0.0001	<0.0001
IFI27L1	1.518	1.226	26.772	<0.0001	<0.0001
IFI6	1.914	7.308	26.704	<0.0001	<0.0001
PHGDH	1.407	1.641	26.627	<0.0001	<0.0001
SCARB2	0.678	5.543	26.571	<0.0001	<0.0001
PLEK2	-1.817	2.914	26.543	<0.0001	<0.0001
BOLA2B	0.97	3.557	26.498	<0.0001	<0.0001
TIGD3	-1.152	3.306	26.483	<0.0001	<0.0001
DECR2	-4.789	9.316	26.472	<0.0001	<0.0001
LAP3	1.286	6.354	26.463	<0.0001	<0.0001
PSAT1	1.254	2.216	26.458	<0.0001	<0.0001
IGKV1-8	1.817	1.008	26.454	<0.0001	<0.0001

DENND5B	1.607	2.898	26.382	<0.0001	<0.0001
UBE2S	0.861	3.929	26.331	<0.0001	<0.0001
BMAL2	1.641	0.775	26.229	<0.0001	<0.0001
ERLEC1	0.853	4.971	26.166	<0.0001	<0.0001
BTG3	0.904	1.93	26.149	<0.0001	<0.0001
BRIP1	1.126	1.011	26.101	<0.0001	<0.0001
ANXA4	0.606	4.829	26.096	<0.0001	<0.0001
LILRB4	1.075	4.993	26.079	<0.0001	<0.0001
VPS37C	-0.722	4.421	26.067	<0.0001	<0.0001
CCNF	1.294	2.029	26.057	<0.0001	<0.0001
C1QC	2.812	1.267	25.993	<0.0001	<0.0001
MANF	1.158	4.994	25.942	<0.0001	<0.0001
FAM83D	1.436	-0.024	25.941	<0.0001	<0.0001
CEP128	1.227	2.52	25.864	<0.0001	<0.0001
MLH1	0.619	4.05	25.838	<0.0001	<0.0001
HAGHL	-4.729	9.128	25.759	<0.0001	<0.0001
HYPK	0.831	3.953	25.746	<0.0001	<0.0001
MRPL27	0.799	3.317	25.707	<0.0001	<0.0001
PSMA5	0.654	5.761	25.703	<0.0001	<0.0001
CENPI	1.653	-0.063	25.622	<0.0001	<0.0001
PCK2	0.684	3.942	25.586	<0.0001	<0.0001
CA1	-3.273	6.728	25.584	<0.0001	<0.0001
MBNL3	-1.189	6.901	25.583	<0.0001	<0.0001
IFIH1	1.129	6.008	25.548	<0.0001	<0.0001
TSPAN5	-1.294	5.194	25.479	<0.0001	<0.0001
NUGGC	1.208	2.387	25.46	<0.0001	<0.0001
MAD2L1	1.182	2.127	25.411	<0.0001	<0.0001
H2AX	0.958	3.926	25.406	<0.0001	<0.0001
BOLA2	1.257	2.498	25.312	<0.0001	<0.0001
ABCB9	1.854	1.285	25.3	<0.0001	<0.0001

CIP2A	1.48	1.556	25.281	<0.0001	<0.0001
ENSG00000261659	-4.988	9.578	25.278	<0.0001	<0.0001
GATA1	-1.045	4.449	25.183	<0.0001	<0.0001
DDX60	1.341	6.173	25.122	<0.0001	<0.0001
ENSG00000289514	-1.695	2.988	25.112	<0.0001	<0.0001
IGKV2-24	2.532	2.995	25.049	<0.0001	<0.0001
NDUFA9	0.723	4.762	25.039	<0.0001	<0.0001
ALG14	1.465	1.259	25.03	<0.0001	<0.0001
C1QB	1.869	2.521	24.913	<0.0001	<0.0001
NEXN	1.323	3.743	24.896	<0.0001	<0.0001
BST2	0.845	6.326	24.852	<0.0001	<0.0001
SPCS2P4	1.289	1.681	24.63	<0.0001	<0.0001
TRIM58	-1.948	7.66	24.598	<0.0001	<0.0001
PPIAP31	1.108	-0.173	24.578	<0.0001	<0.0001
RBBP8	0.863	3.639	24.541	<0.0001	<0.0001
C2	1.769	2.722	24.524	<0.0001	<0.0001
IGF2BP2	-1.738	5.092	24.439	<0.0001	<0.0001
CIT	1.259	0.823	24.367	<0.0001	<0.0001
Y_RNA	-3.008	5.589	24.343	<0.0001	<0.0001
PTPRO	0.948	3.413	24.312	<0.0001	<0.0001
STRADB	-1.78	7.243	24.246	<0.0001	<0.0001
GPT2	1.375	1.017	24.226	<0.0001	<0.0001
TMEM126B	0.575	3.939	24.122	<0.0001	<0.0001
DNAJB2	-0.741	6.016	24.119	<0.0001	<0.0001
CCNE1	1.68	0.837	24.054	<0.0001	<0.0001
IGHGP	1.899	3.291	24.034	<0.0001	<0.0001
PLSCR1	1.251	6.223	24.019	<0.0001	<0.0001
LGALS1	0.985	6.745	23.994	<0.0001	<0.0001
AGO2	-0.751	6.396	23.977	<0.0001	<0.0001
UBB	-1.487	10.791	23.955	<0.0001	<0.0001

NUSAP1	1.84	2.506	23.928	<0.0001	<0.0001
IGHV1-3	2.414	2.344	23.86	<0.0001	<0.0001
SQLE	0.903	3.114	23.815	<0.0001	<0.0001
FBXO7	-1.588	9.871	23.809	<0.0001	<0.0001
IGKV2D-30	2.194	2.5	23.784	<0.0001	<0.0001
FAM72D	1.45	-0.362	23.78	<0.0001	<0.0001
IGF1R	-1.12	6.038	23.762	<0.0001	<0.0001
ATP5F1C	0.704	5.826	23.752	<0.0001	<0.0001
BIK	1.375	0.379	23.746	<0.0001	<0.0001
ACADM	0.661	4.435	23.723	<0.0001	<0.0001
FADS2	1.244	3.213	23.721	<0.0001	<0.0001
CLN8-AS1	-1.178	4.531	23.707	<0.0001	<0.0001
EBP	0.823	3.68	23.64	<0.0001	<0.0001
CKS1B	1.005	2.276	23.6	<0.0001	<0.0001
H2BC5	1.27	3.807	23.501	<0.0001	<0.0001
NASP	0.577	5.438	23.463	<0.0001	<0.0001
PDZK1IP1	-1.555	6.157	23.433	<0.0001	<0.0001
TUBB2B	-2.22	3.443	23.414	<0.0001	<0.0001
SNRPG	0.795	4.726	23.364	<0.0001	<0.0001
TOR3A	0.667	4.704	23.336	<0.0001	<0.0001
EPOP	1.162	-0.006	23.306	<0.0001	<0.0001
RFLNB	-1.165	6.994	23.255	<0.0001	<0.0001
HIBCH	0.869	2.839	23.222	<0.0001	<0.0001
NUP37	0.758	2.891	23.19	<0.0001	<0.0001
IGHV3-73	2.666	1.889	23.168	<0.0001	<0.0001
CCNG2	-0.64	6.301	23.109	<0.0001	<0.0001
GPR157	-0.959	2.165	23.074	<0.0001	<0.0001
IGKV2-30	2.074	3.736	23.072	<0.0001	<0.0001
ENSG00000228686	-1.699	-0.112	23.021	<0.0001	<0.0001
BAK1	0.85	5.347	23.012	<0.0001	<0.0001

HLA-H	1.587	4.985	22.986	<0.0001	<0.0001
CYCS	0.701	5.429	22.959	<0.0001	<0.0001
AARS1	0.758	5.274	22.932	<0.0001	<0.0001
PDIA6	1	6.453	22.93	<0.0001	<0.0001
ENSG00000262714	-1.046	4.203	22.91	<0.0001	<0.0001
POLL	-1.002	5.228	22.901	<0.0001	<0.0001
RPUSD1	-3.839	9.043	22.888	<0.0001	<0.0001
CASP7	0.723	4.293	22.879	<0.0001	<0.0001
SLC25A37	-1.238	10.743	22.829	<0.0001	<0.0001
IFITM3	1.673	9.374	22.82	<0.0001	<0.0001
APOBEC3B	1.345	3.792	22.818	<0.0001	<0.0001
C3AR1	0.926	5.622	22.815	<0.0001	<0.0001
OPTN	-0.997	7.461	22.812	<0.0001	<0.0001
WDR76	0.83	2.889	22.8	<0.0001	<0.0001
MRPS7	0.679	4.318	22.79	<0.0001	<0.0001
AKT1S1	-0.715	4.295	22.662	<0.0001	<0.0001
ODAD4	-1.919	1.743	22.636	<0.0001	<0.0001
BCL2L1	-1.656	8.363	22.523	<0.0001	<0.0001
FAM210B	-1.703	7.951	22.499	<0.0001	<0.0001
FAM98A	0.693	4.035	22.489	<0.0001	<0.0001
PSMA6	0.787	6.074	22.473	<0.0001	<0.0001
BLM	0.891	3.157	22.463	<0.0001	<0.0001
LRRC59	0.695	5.424	22.416	<0.0001	<0.0001
SFRP2	-2.758	0.61	22.392	<0.0001	<0.0001
CCT8	0.616	6.52	22.389	<0.0001	<0.0001
ENSG00000279386	-1.019	3.165	22.383	<0.0001	<0.0001
BMP8B	1.096	2.044	22.38	<0.0001	<0.0001
RANBP1	0.696	4.689	22.36	<0.0001	<0.0001
SEC24A	0.789	4.512	22.359	<0.0001	<0.0001
PCGF5	-0.878	7.709	22.341	<0.0001	<0.0001

ENSG00000285920	2.448	1.634	22.328	<0.0001	<0.0001
KNDC1	-1.826	1.234	22.291	<0.0001	<0.0001
TNS1	-1.708	6.306	22.266	<0.0001	<0.0001
TMEM164	-0.953	7.157	22.236	<0.0001	<0.0001
MCM3	0.581	5.306	22.218	<0.0001	<0.0001
TSPAN5-DT	-1.679	1.409	22.197	<0.0001	<0.0001
ANK1	-1.694	5.645	22.174	<0.0001	<0.0001
KIAA1958	1.094	2.402	22.109	<0.0001	<0.0001
CDC34	-1.164	6.611	22.098	<0.0001	<0.0001
PPM1A	-0.809	7.288	22.087	<0.0001	<0.0001
POMP	0.762	5.066	22.081	<0.0001	<0.0001
MT1E	1.369	0.881	22.033	<0.0001	<0.0001
BRCA1	0.754	3.554	22.004	<0.0001	<0.0001
IFI27L2	0.854	3.579	22	<0.0001	<0.0001
PSME2	0.866	6.917	21.958	<0.0001	<0.0001
CDH2	-2.106	1.177	21.953	<0.0001	<0.0001
SSR3	0.824	6.288	21.941	<0.0001	<0.0001
DSCC1	1.835	0.175	21.938	<0.0001	<0.0001
MRPS11	0.661	3.583	21.866	<0.0001	<0.0001
IGLV2-8	3.347	4.382	21.83	<0.0001	<0.0001
QPCTL	0.991	1.278	21.828	<0.0001	<0.0001
SIL1	0.907	3.575	21.813	<0.0001	<0.0001
ANKH	-0.751	5.686	21.779	<0.0001	<0.0001
ITGB7	0.713	5.769	21.773	<0.0001	<0.0001
PSME2P2	1.097	1.149	21.766	<0.0001	<0.0001
BLVRA	0.94	4.956	21.721	<0.0001	<0.0001
PPP3CA	-0.597	6.389	21.712	<0.0001	<0.0001
SELENOH	0.55	5.657	21.668	<0.0001	<0.0005
IGHV3-72	2.171	1.681	21.667	<0.0001	<0.0005
MDH1	0.583	5.262	21.65	<0.0001	<0.0005

ADIPOR1	-1.373	9.688	21.582	<0.0001	<0.0005
C3orf38	0.519	4.495	21.565	<0.0001	<0.0005
SNRPD1	0.732	4.13	21.558	<0.0001	<0.0005
C12orf4	0.703	3.342	21.547	<0.0001	<0.0005
P2RY6	1.504	0.854	21.534	<0.0001	<0.0005
BCAM	-3.327	0.933	21.526	<0.0001	<0.0005
CEP15	1.218	0.223	21.516	<0.0001	<0.0005
IFIT5	1.044	6.169	21.515	<0.0001	<0.0005
IFI27	3.476	7.363	21.511	<0.0001	<0.0005
PGM5	-2.119	2.49	21.507	<0.0001	<0.0005
RCC2	0.555	5.985	21.504	<0.0001	<0.0005
MAFB	0.642	5.492	21.491	<0.0001	<0.0005
CTSL	1.091	3.586	21.418	<0.0001	<0.0005
MED25	-0.791	6.597	21.407	<0.0001	<0.0005
CASP3	0.63	5.716	21.406	<0.0001	<0.0005
MRPL58	0.766	3.235	21.381	<0.0001	<0.0005
HYOU1	0.851	6.278	21.374	<0.0001	<0.0005
PREB	0.592	5.078	21.347	<0.0001	<0.0005
STT3A	0.792	5.91	21.331	<0.0001	<0.0005
RPS27L	0.657	4.409	21.31	<0.0001	<0.0005
OSTC	0.818	5.001	21.31	<0.0001	<0.0005
MPDU1	0.693	4.408	21.279	<0.0001	<0.0005
IGKV2D-29	2.122	1.027	21.264	<0.0001	<0.0005
RANP1	1.071	0.487	21.239	<0.0001	<0.0005
CUL1	0.582	5.539	21.232	<0.0001	<0.0005
NHLRC4	-5.378	8.347	21.23	<0.0001	<0.0005
IFI35	0.999	5.543	21.205	<0.0001	<0.0005
RTCB	0.609	5.275	21.195	<0.0001	<0.0005
ENSG00000260496	-5.393	7.639	21.189	<0.0001	<0.0005
TUBG1	0.961	2.75	21.128	<0.0001	<0.0005

DNA2	1.107	0.943	21.077	<0.0001	<0.0005
TEK	-2.094	0.638	21.042	<0.0001	<0.0005
PI4K2B	0.716	3.967	21.028	<0.0001	<0.0005
KLHDC7B	1.275	3.25	21.008	<0.0001	<0.0005
BCL2L12	0.831	2.768	20.995	<0.0001	<0.0005
PAQR4	0.801	2.664	20.994	<0.0001	<0.0005
NCAPH2	0.612	5.085	20.943	<0.0001	<0.0005
CHST12	0.676	4.925	20.929	<0.0001	<0.0005
NR6A1	-1.066	2.435	20.88	<0.0001	<0.0005
MRPS18B	0.622	4.676	20.831	<0.0001	<0.0005
FDXR	0.819	2.14	20.787	<0.0001	<0.0005
MAIP1	0.803	2.357	20.769	<0.0001	<0.0005
CACNA1H	-5.54	8.698	20.691	<0.0001	<0.0005
ZNG1A	0.648	3.899	20.691	<0.0001	<0.0005
SMIM24	-1.983	3.353	20.683	<0.0001	<0.0005
VANGL1	0.686	2.68	20.666	<0.0001	<0.0005
TOMM40L	0.63	3.036	20.642	<0.0001	<0.0005
LGMN	1.052	2.391	20.604	<0.0001	<0.0005
IFI44	1.436	3.531	20.601	<0.0001	<0.0005
OXCT1	0.621	4.238	20.594	<0.0001	<0.0005
ENSG00000285382	1.042	1.647	20.543	<0.0001	<0.0005
MAD2L1BP	0.631	4.24	20.519	<0.0001	<0.0005
SEC61G	0.919	3.971	20.506	<0.0001	<0.0005
CADM1	1.324	1.758	20.486	<0.0001	<0.0005
IGKV1-6	2.075	2.236	20.48	<0.0001	<0.0005
NDUFV2	0.675	5.126	20.434	<0.0001	<0.0005
SMC4	0.679	5.285	20.422	<0.0001	<0.0005
SELENOS	0.764	3.992	20.415	<0.0001	<0.0005
CRELD2	0.913	4.179	20.392	<0.0001	<0.0005
CCT2	0.612	5.513	20.262	<0.0001	<0.0005

SEL1L3	0.937	6.241	20.256	<0.0001	<0.0005
CCNC	0.709	4.583	20.19	<0.0001	<0.0005
BORA	0.896	2.065	20.186	<0.0001	<0.0005
UQCRQ	0.737	4.662	20.141	<0.0001	<0.0005
ENSG00000287642	-2.279	-0.244	20.137	<0.0001	<0.0005
WFIKKN1	-5.043	9.544	20.093	<0.0001	<0.0005
CARM1	-0.791	5.744	20.091	<0.0001	<0.0005
YY1AP1	-0.596	6.708	20.08	<0.0001	<0.0005
ALG5	0.849	3.583	20.073	<0.0001	<0.0005
SLC25A39	-1.656	10.174	20.016	<0.0001	<0.0005
SNCA	-2.116	8.023	20.001	<0.0001	<0.0005
GALM	0.759	4.314	19.97	<0.0001	<0.0005
MYL6B	1.116	1.83	19.948	<0.0001	<0.0005
SUB1	0.886	7.096	19.944	<0.0001	<0.0005
SEC61B	0.806	5.192	19.944	<0.0001	<0.0005
COX7A2	0.676	5.368	19.941	<0.0001	<0.0005
FECH	-1.809	5.526	19.931	<0.0001	<0.0005
SLC2A5	1.478	1.489	19.924	<0.0001	<0.0005
IFIT1	2.029	7.973	19.92	<0.0001	<0.0005
GMPR	-1.39	5.556	19.917	<0.0001	<0.0005
TUBB2A	-2.567	5.034	19.915	<0.0001	<0.0005
MCM8	0.841	2.5	19.907	<0.0001	<0.0005
NT5C3A	1	6.621	19.906	<0.0001	<0.0005
MXI1	-1.554	6.976	19.901	<0.0001	<0.0005
GLRX5	-1.419	6.043	19.888	<0.0001	<0.0005
ENSG00000293278	-2.017	-0.107	19.844	<0.0001	<0.0005
PHOSPHO1	-1.319	7.925	19.816	<0.0001	<0.0005
RGS16	1.516	0.557	19.804	<0.0001	<0.0005
GSPT1	-1.127	7.738	19.761	<0.0001	<0.0005
LINC02975	-0.947	4.145	19.727	<0.0001	<0.0005

MOXD1	1.765	0.057	19.726	<0.0001	<0.0005
RANBP10	-0.977	5.646	19.716	<0.0001	<0.0005
H2BC6-AS1	1.209	0.727	19.705	<0.0001	<0.0005
PSMF1	-0.811	7.289	19.682	<0.0001	<0.0005
CAMK1D	-0.917	6.574	19.652	<0.0001	<0.0005
IDH2	0.645	5.892	19.635	<0.0001	<0.0005
DDIT3	0.742	4.096	19.616	<0.0001	<0.0005
HSPE1	0.732	4.263	19.612	<0.0001	<0.0005
SCAMP5	1.231	1.69	19.591	<0.0001	<0.0005
GAS6	1.313	2.268	19.577	<0.0001	<0.0005
HVCN1	-0.627	5.965	19.573	<0.0001	<0.0005
EEF1E1	0.785	2.677	19.556	<0.0001	<0.0005
DNAJC9	0.755	4.397	19.538	<0.0001	<0.0005
EEF1AKMT4	1.108	0.788	19.533	<0.0001	<0.0005
ADGRE3	-0.898	6.793	19.512	<0.0001	<0.0005
SNRPGP10	0.899	0.798	19.511	<0.0001	<0.0005
ENSG00000293232	-5.132	8.616	19.494	<0.0001	<0.0005
IGHJ3	2.426	5.151	19.492	<0.0001	<0.0005
ELAPOR1	-1.545	5.929	19.477	<0.0001	<0.0005
CMPK2	1.808	5.742	19.466	<0.0001	<0.0005
FBXO9	-0.964	5.932	19.434	<0.0001	<0.0005
TOPORS	-0.596	5.816	19.419	<0.0001	<0.0005
SLC35B1	0.755	4.04	19.407	<0.0001	<0.0005
ENSG00000293331	-1.479	0.132	19.407	<0.0001	<0.0005
PSMA4	0.612	6.322	19.382	<0.0001	<0.0005
MTHFD1	0.65	4.306	19.369	<0.0001	<0.0005
EGR1	1.075	1.725	19.345	<0.0001	<0.0005
JAZF1	-0.775	5.254	19.31	<0.0001	<0.0005
ZBP1	0.912	5.784	19.254	<0.0001	<0.0005
FAM117B	-0.541	4.61	19.253	<0.0001	<0.0005

RFC4	0.927	2.438	19.244	<0.0001	<0.0005
ZNF395	-2.653	2.021	19.244	<0.0001	<0.0005
CCDC78	-4.86	9.65	19.221	<0.0001	<0.0005
SKI	-0.634	6.521	19.201	<0.0001	<0.0005
ENSG00000291048	-0.879	4.537	19.191	<0.0001	<0.0005
MPP1	-1.081	7.332	19.191	<0.0001	<0.0005
ENOSF1	0.998	3.28	19.108	<0.0001	<0.0005
CCT3	0.566	6.421	19.102	<0.0001	<0.0005
TIMM10	0.823	3.216	19.039	<0.0001	<0.0005
ISOC2	0.931	2.719	19.026	<0.0001	<0.0005
IGHA2	2.026	5.75	18.981	<0.0001	<0.0005
CNPPD1	-0.887	6.827	18.965	<0.0001	<0.0005
FARSB	0.664	4.01	18.938	<0.0001	<0.0005
WDR62	1.44	0.575	18.92	<0.0001	<0.0005
RPN2	0.779	7.131	18.913	<0.0001	<0.0005
MRPS18C	0.788	3.262	18.895	<0.0001	<0.0005
IFIT3	1.588	8.726	18.894	<0.0001	<0.0005
GPD2	0.497	4.73	18.844	<0.0001	<0.0005
ILF2	0.571	6.053	18.813	<0.0001	<0.0005
CAMKK1	-0.861	3.499	18.806	<0.0001	<0.0005
LMNB2	0.602	4.486	18.789	<0.0001	<0.0005
MGAT2	0.524	5.178	18.767	<0.0001	<0.0005
UBE2J1	0.859	7.314	18.735	<0.0001	<0.0005
CCDC13-AS2	-1.121	2.244	18.716	<0.0001	<0.0005
CHTF18	-4.367	9.979	18.702	<0.0001	<0.0005
RAN	0.614	6.658	18.698	<0.0001	<0.0005
IGLV1-40	2.776	3.493	18.696	<0.0001	<0.0005
ENSG00000292432	-5.437	7.629	18.69	<0.0001	<0.0005
TPGS2	-0.974	5.993	18.659	<0.0001	<0.0005
PSMB6	0.649	5.106	18.657	<0.0001	<0.0005

SERPING1	1.632	5.895	18.653	<0.0001	<0.0005
NFIX	-1.386	5.25	18.578	<0.0001	<0.0005
SEPTIN4	1.188	2.235	18.55	<0.0001	<0.0005
ECT2	0.852	2.463	18.531	<0.0001	<0.0005
ALAS2	-2.253	9.61	18.526	<0.0001	<0.0005
DPM2	-0.825	5.106	18.474	<0.0001	<0.0005
SPATA6	-0.839	2.057	18.466	<0.0001	<0.0005
ZRANB1	-0.65	4.965	18.46	<0.0001	<0.0005
TPPP	-1.14	1.886	18.427	<0.0001	<0.0005
NOMO1	0.6	5.457	18.409	<0.0001	<0.0005
DCAF12	-1.428	8.069	18.393	<0.0001	<0.0005
ACVRL1	1.396	0.28	18.381	<0.0001	<0.0005
COX5A	0.687	5.198	18.323	<0.0001	<0.0005
AFTPH-DT	1.108	0.852	18.323	<0.0001	<0.0005
CCDC167	1.007	2.863	18.319	<0.0001	<0.0005
ZNG1B	0.547	4.051	18.31	<0.0001	<0.0005
SLIRP	0.74	3.218	18.241	<0.0001	<0.0005
PSMA2	0.594	5.756	18.231	<0.0001	<0.0005
PPP1R15A	-0.649	6.732	18.224	<0.0001	<0.0005
XAF1	1.942	6.892	18.173	<0.0001	<0.0005
MAD2L2	0.665	4.623	18.168	<0.0001	<0.0005
MYO1D	1.154	2.826	18.167	<0.0001	<0.0005
DIPK1A	0.792	3.556	18.153	<0.0001	<0.0005
TMEM156	0.705	3.557	18.125	<0.0001	<0.0005
SRPRB	0.719	4.277	18.089	<0.0001	<0.0005
ENSG00000288156	-0.733	4.479	18.078	<0.0001	<0.0005
MYOF	0.844	4.883	18.076	<0.0001	<0.0005
ENSG00000293004	1.358	0.953	18.073	<0.0001	<0.0005
NDUFA4	0.61	5.369	18.068	<0.0001	<0.0005
NPL	-0.83	6.418	18.048	<0.0001	<0.0005

DHX58	0.962	4.188	18.043	<0.0001	<0.0005
COA6	0.67	3.263	18.023	<0.0001	<0.0005
AGRN	1.465	2.911	18.022	<0.0001	<0.0005
EPB42	-1.954	5.999	18.01	<0.0001	<0.0005
ALDH5A1	-0.838	4	18.006	<0.0001	<0.0005
FAM72A	1.234	0.084	17.992	<0.0001	<0.0005
MILR1	0.685	4.338	17.97	<0.0001	<0.005
INTS13	0.555	3.785	17.97	<0.0001	<0.005
SAR1B	0.583	4.46	17.959	<0.0001	<0.005
TMEM106A	0.63	3.004	17.937	<0.0001	<0.005
GBP6	1.353	1.561	17.935	<0.0001	<0.005
IFI44L	2.081	7.662	17.934	<0.0001	<0.005
YBX3	-1.612	8.834	17.928	<0.0001	<0.005
ENSG00000268903	-1.293	6.497	17.902	<0.0001	<0.005
MCOLN1	-0.684	4.701	17.896	<0.0001	<0.005
MS4A4A	1.102	2.637	17.865	<0.0001	<0.005
VRK2	0.586	3.744	17.844	<0.0001	<0.005
DTX4	-0.85	3.36	17.793	<0.0001	<0.005
PSMA3	0.533	5.266	17.755	<0.0001	<0.005
MRPL22	0.764	3.307	17.742	<0.0001	<0.005
MICB	0.55	4.791	17.684	<0.0001	<0.005
SLC25A4	1.004	1.911	17.68	<0.0001	<0.005
MRPL18	0.551	4.424	17.673	<0.0001	<0.005
MT1F	0.923	1.563	17.664	<0.0001	<0.005
TDRD7	0.65	4.962	17.664	<0.0001	<0.005
MTFR2	1.58	0.029	17.634	<0.0001	<0.005
PARPBP	1.153	0.898	17.613	<0.0001	<0.005
MTHFD1L	1.024	1.68	17.59	<0.0001	<0.005
NUCB2	0.58	5.216	17.589	<0.0001	<0.005
UCK2	0.779	2.702	17.577	<0.0001	<0.005

DMTN	-1.312	8.476	17.57	<0.0001	<0.005
MORF4L2	0.506	5.062	17.562	<0.0001	<0.005
ENSG00000284820	1.478	2.951	17.516	<0.0001	<0.005
FCHO1	-0.508	5.527	17.501	<0.0001	<0.005
MFSD2B	-1.372	3.439	17.482	<0.0001	<0.005
KEL	-1.428	2.347	17.462	<0.0001	<0.005
CNTNAP3	-1.719	4.331	17.46	<0.0001	<0.005
ARPC5L	0.581	4.688	17.457	<0.0001	<0.005
BTN2A2	0.538	4.276	17.456	<0.0001	<0.005
ENSG00000235105	-1.505	1.737	17.455	<0.0001	<0.005
PNRC2P1	0.8	0.338	17.452	<0.0001	<0.005
DHRS9	0.937	5.213	17.446	<0.0001	<0.005
SRSF8	-0.477	5.335	17.443	<0.0001	<0.005
TMPO-AS1	0.88	0.835	17.428	<0.0001	<0.005
CHAF1A	0.667	3.436	17.422	<0.0001	<0.005
CASTOR2	-0.803	3.166	17.336	<0.0001	<0.005
FADS1	0.755	3.029	17.335	<0.0001	<0.005
CIAO2B	0.545	4.393	17.327	<0.0001	<0.005
SELENOI	0.846	3.353	17.324	<0.0001	<0.005
SLC44A1	0.664	5.152	17.323	<0.0001	<0.005
RMI2	1.067	1.445	17.321	<0.0001	<0.005
MT1X	0.844	1.727	17.282	<0.0001	<0.005
ARHGAP11A	1.063	2.435	17.257	<0.0001	<0.005
TCP11L2	-0.879	5.726	17.251	<0.0001	<0.005
GGTA1	-1.036	3.989	17.25	<0.0001	<0.005
UBE2SP1	1.009	0.074	17.227	<0.0001	<0.005
SESN3	-0.759	6.881	17.226	<0.0001	<0.005
CLPTM1L	0.647	5.884	17.22	<0.0001	<0.005
GBP1	1.154	7.783	17.217	<0.0001	<0.005
ENSG00000289176	-1.128	3.762	17.213	<0.0001	<0.005

NDUFAB1	0.658	4.158	17.191	<0.0001	<0.005
PCSK1N	-1.321	1.706	17.187	<0.0001	<0.005
ST8SIA1	-0.975	2.098	17.181	<0.0001	<0.005
EPOR	-0.722	3.587	17.164	<0.0001	<0.005
ROMO1	0.756	3.451	17.162	<0.0001	<0.005
ST13	-0.593	7.145	17.161	<0.0001	<0.005
MPZL3	-0.76	6.304	17.12	<0.0001	<0.005
GNL2	0.519	4.798	17.106	<0.0001	<0.005
DTYMK	0.827	2.228	17.031	<0.0001	<0.005
PRDX6	-0.852	7.417	17.024	<0.0001	<0.005
PRKCA	-0.588	4.654	17.018	<0.0001	<0.005
POLA1	0.68	3.2	16.998	<0.0001	<0.005
ANXA2P2	0.868	1.859	16.995	<0.0001	<0.005
LARP7	0.445	5.165	16.975	<0.0001	<0.005
CNTNAP3P2	-2.304	0.677	16.938	<0.0001	<0.005
NET1	1.018	3.346	16.918	<0.0001	<0.005
MEOX1	-1.2	0.787	16.903	<0.0001	<0.005
FH	0.639	4.234	16.875	<0.0001	<0.005
TUBAP2	0.872	1.725	16.869	<0.0001	<0.005
GMPPA	0.673	3.996	16.852	<0.0001	<0.005
ENSG00000293141	-1.109	-0.196	16.849	<0.0001	<0.005
ATP5MC1	0.807	3.685	16.838	<0.0001	<0.005
ETV7	1.388	3.669	16.822	<0.0001	<0.005
RILP	-0.886	5.167	16.81	<0.0001	<0.005
CDCA4	0.67	2.687	16.804	<0.0001	<0.005
ZBTB18	-0.575	6.552	16.803	<0.0001	<0.005
LAG3	0.855	3.152	16.757	<0.0001	<0.005
MRPS12	0.656	3.366	16.749	<0.0001	<0.005
ENSG00000219928	0.938	0.447	16.728	<0.0001	<0.005
SHARPIN	-0.831	5.933	16.725	<0.0001	<0.005

PAICS	0.725	4.524	16.721	<0.0001	<0.005
CREB3L2	0.482	5.186	16.719	<0.0001	<0.005
HSPD1	0.583	6.508	16.717	<0.0001	<0.005
ITGB4	-1.863	1.645	16.691	<0.0001	<0.005
HSPA13	0.77	4.529	16.683	<0.0001	<0.005
FAM30A	1.172	4.104	16.68	<0.0001	<0.005
SELENBP1	-2.176	6.466	16.668	<0.0001	<0.005
HAUS1	0.668	3.29	16.654	<0.0001	<0.005
IPMK	-0.744	5.647	16.622	<0.0001	<0.005
DYNLT2B	0.848	0.572	16.618	<0.0001	<0.005
LARP4	0.537	4.344	16.615	<0.0001	<0.005
FIRRM	0.695	2.105	16.595	<0.0001	<0.005
PPA1	0.662	5.366	16.585	<0.0001	<0.005
DDX11L2	-1.199	2.779	16.581	<0.0001	<0.005
PPM1F	-0.745	6.854	16.537	<0.0001	<0.005
DARS2	0.544	3.261	16.503	<0.0001	<0.005
GART	0.47	4.977	16.493	<0.0001	<0.005
ATG9A	-0.661	5.223	16.489	<0.0001	<0.005
S100B	2.335	2.458	16.471	<0.0001	<0.005
AATK	-0.737	6.185	16.467	<0.0001	<0.005
RFC3	0.833	2.103	16.455	<0.0001	<0.005
PTAFR	-0.762	8.586	16.454	<0.0001	<0.005
APOBEC3F	0.574	3.884	16.427	<0.0001	<0.005
LAMC1	1.14	2.992	16.414	<0.0001	<0.005
SLC4A1	-2.009	8.259	16.383	<0.0001	<0.005
FAXDC2	-1.107	5.428	16.381	<0.0001	<0.005
GINS3	0.912	1.15	16.356	<0.0001	<0.005
RPGRIP1	-0.85	2.761	16.343	<0.0001	<0.005
LINC02887	-1.039	4.378	16.325	<0.0001	<0.005
SLC3A2	0.49	5.193	16.312	<0.0001	<0.005

DCTPP1	0.666	3.108	16.291	<0.0001	<0.005
CDC7	0.866	2.426	16.287	<0.0001	<0.005
FOXO3	-0.849	6.876	16.274	<0.0001	<0.005
PPIAP22	0.582	4.749	16.27	<0.0001	<0.005
TIMM8B	0.652	4.086	16.27	<0.0001	<0.005
RARS1	0.45	4.595	16.259	<0.0001	<0.005
BHLHE41	1.318	1.107	16.257	<0.0001	<0.005
METTL5	0.648	3.256	16.242	<0.0001	<0.005
SIRPB1	-0.787	8.205	16.228	<0.0001	<0.005
CYC1	0.556	4.706	16.195	<0.0001	<0.005
CNTNAP3B	-1.806	1.623	16.191	<0.0001	<0.005
DNAJC15	0.568	4.482	16.186	<0.0001	<0.005
CDK2	0.641	3.111	16.145	<0.0001	<0.005
SNRPGP15	1.112	-0.552	16.127	<0.0001	<0.005
ENSG00000250644	-4.032	3.893	16.118	<0.0001	<0.005
ZNF496-DT	1.086	0.42	16.118	<0.0001	<0.005
SLC25A19	0.778	2.175	16.104	<0.0001	<0.005
PROB1	1.114	0.751	16.101	<0.0001	<0.005
MICAL2	-1.102	7.347	16.098	<0.0001	<0.005
TMEM208	0.704	3.635	16.097	<0.0001	<0.005
VIPR1	-0.644	3.705	16.09	<0.0001	<0.005
RPA3	0.627	3.316	16.085	<0.0001	<0.005
PSMD14	0.652	4.624	16.083	<0.0001	<0.005
MRPL15	0.604	3.623	16.08	<0.0001	<0.005
FAM20C	1.051	2.714	16.075	<0.0001	<0.005
IDH1	0.505	5.096	16.07	<0.0001	<0.005
CHPT1	-0.94	5.963	16.031	<0.0001	<0.005
LDLR	0.747	4.071	16.027	<0.0001	<0.005
SPCS2	0.646	5.525	16.022	<0.0001	<0.005
COA4	0.595	3.629	16.019	<0.0001	<0.005

R3HDM4	-0.925	9.357	16.014	<0.0001	<0.005
LINC02772	-1.157	2.8	15.996	<0.0001	<0.005
GALK2	0.549	3.403	15.987	<0.0001	<0.005
ZER1	-0.655	6.172	15.971	<0.0001	<0.005
IL1RAP	-1.002	6.184	15.959	<0.0001	<0.005
EPHB4	-1.274	4.114	15.946	<0.0001	<0.005
NECTIN1	-0.666	3.927	15.935	<0.0001	<0.005
SGO1	1.173	0.412	15.932	<0.0001	<0.005
TRIM7	0.891	1.596	15.924	<0.0001	<0.005
BOLA3	0.774	1.899	15.922	<0.0001	<0.005
HID1	1.415	0.975	15.918	<0.0001	<0.005
SLC9A9	0.481	4.028	15.892	<0.0001	<0.005
SPATA24	0.792	0.793	15.876	<0.0001	<0.005
NSD2	0.572	4.752	15.863	<0.0001	<0.005
FBXO6	1.034	3.364	15.86	<0.0001	<0.005
HARBI1	0.641	1.707	15.858	<0.0001	<0.005
TBC1D17	-0.525	4.698	15.84	<0.0001	<0.005
MCTS1	0.492	4.627	15.838	<0.0001	<0.005
RRBP1	0.562	6.589	15.8	<0.0001	<0.005
CMTR1	0.61	5.488	15.793	<0.0001	<0.005
LYL1	-0.812	6.977	15.788	<0.0001	<0.005
ENSG00000273284	-0.995	-0.582	15.783	<0.0001	<0.005
BYSL	0.71	2.11	15.782	<0.0001	<0.005
RPL39L	1.072	0.859	15.777	<0.0001	<0.005
HGSNAT	-0.401	5.368	15.757	<0.0001	<0.005
NDUFA8	0.62	3.713	15.752	<0.0001	<0.005
UBE4B	-0.424	5.763	15.707	<0.0001	<0.005
XRCC2	1.302	-0.217	15.682	<0.0001	<0.005
EIF2S1	0.442	5.166	15.68	<0.0001	<0.005
TMED9	0.587	6.048	15.654	<0.0001	<0.005

PFN2	-1.037	0.726	15.638	<0.0001	<0.005
PITPNA	-0.534	6.598	15.633	<0.0001	<0.005
SRP19	0.447	4.438	15.615	<0.0001	<0.005
HMGB1P1	0.737	0.26	15.608	<0.0001	<0.005
FASTKD1	0.692	2.966	15.593	<0.0001	<0.005
GCAT	-1.588	1.19	15.587	<0.0001	<0.005
ALG8	0.679	3.591	15.578	<0.0001	<0.005
DCPS	0.623	4.365	15.577	<0.0001	<0.005
MKRN1	-0.955	8.537	15.575	<0.0001	<0.005
NQO1	0.957	0.702	15.563	<0.0001	<0.005
MIR600HG	-0.967	1.946	15.548	<0.0001	<0.005
ENSG00000288997	-1.063	1.122	15.488	<0.0001	<0.005
RNF123	-0.745	5.07	15.487	<0.0001	<0.005
AKR1A1	0.604	4.766	15.48	<0.0001	<0.005
ENSG00000282416	-0.941	4.552	15.472	<0.0001	<0.005
HBD	-3.111	10.73	15.466	<0.0001	<0.005
TIMM23	0.578	4.191	15.452	<0.0001	<0.005
TMEM60	0.623	3.511	15.434	<0.0001	<0.005
PARP11	0.526	3.903	15.431	<0.0001	<0.005
TMX1	0.425	5.095	15.431	<0.0001	<0.005
CXCL5	-1.456	4.486	15.431	<0.0001	<0.005
PEX2	0.407	4.681	15.422	<0.0001	<0.005
RBM38	-0.998	7.896	15.413	<0.0001	<0.005
ENSG00000285906	0.823	1.467	15.407	<0.0001	<0.005
EEPD1	-0.551	4.534	15.384	<0.0001	<0.005
IGHV3-13	2.102	1.209	15.372	<0.0001	<0.005
ENTPD7	0.615	3.185	15.356	<0.0001	<0.005
BLVRB	-0.971	7.157	15.343	<0.0001	<0.005
BATF	0.549	4.34	15.341	<0.0001	<0.005
PARP2	0.742	2.385	15.308	<0.0001	<0.005

WEE1	0.624	2.926	15.29	<0.0001	<0.005
EPB41L3	0.66	4.907	15.266	<0.0001	<0.005
RSAD2	2.171	7.58	15.258	<0.0001	<0.005
COX7B	0.602	5.687	15.24	<0.0001	<0.005
TIPIN	0.873	1.04	15.225	<0.0001	<0.005
ENSG00000282988	0.934	3.503	15.223	<0.0001	<0.005
H4C15	0.829	3.16	15.209	<0.0001	<0.005
GARS1	0.546	5.44	15.206	<0.0001	<0.005
AIFM1	0.522	4.133	15.194	<0.0001	<0.005
LINC02471	1.25	1.977	15.188	<0.0001	<0.005
MRPS22	0.484	4.672	15.173	<0.0001	<0.005
ENSG00000290027	1.22	1.913	15.147	<0.0001	<0.005
ADGRE2	-0.79	7.814	15.131	<0.0005	<0.005
MYCT1	1.169	1.421	15.13	<0.0005	<0.005
KLF1	-1.407	3.528	15.124	<0.0005	<0.005
IFIT2	1.123	9.023	15.112	<0.0005	<0.005
ENSG00000255224	1.032	0.384	15.073	<0.0005	<0.005
MRPL1	0.618	3.152	15.072	<0.0005	<0.005
ATP13A1	0.495	5.299	15.068	<0.0005	<0.005
LINC01890	-0.895	2.223	15.057	<0.0005	<0.005
LINC01002	-1.202	3.372	15.048	<0.0005	<0.005
SLFN11	0.501	5.105	15.044	<0.0005	<0.005
ORC3	0.482	3.796	15.043	<0.0005	<0.005
IMPA2	-0.85	5.435	15.042	<0.0005	<0.005
GMPPB	0.797	4.171	15.027	<0.0005	<0.005
ENSG00000250746	0.96	1.217	15.016	<0.0005	<0.005
RPL26L1	0.67	2.907	15.009	<0.0005	<0.005
PNPLA2	-0.603	6.689	14.977	<0.0005	<0.005
CDKN2C	0.72	2.59	14.976	<0.0005	<0.005
GATB	0.642	2.777	14.973	<0.0005	<0.005

KLF7	-0.62	6.126	14.968	<0.0005	<0.005
PNPT1	0.829	3.592	14.93	<0.0005	<0.005
PSMB5	0.556	3.974	14.92	<0.0005	<0.005
NCAL1	0.553	3.783	14.917	<0.0005	<0.005
ATP5MK	0.573	4.825	14.91	<0.0005	<0.005
ATOSB	-0.567	6.176	14.909	<0.0005	<0.005
ENSG00000278133	-0.702	1.812	14.901	<0.0005	<0.005
ZNF684	0.812	1.695	14.888	<0.0005	<0.005
ST13P4	-1.057	-1.106	14.88	<0.0005	<0.005
TCEAL9	1.125	0.174	14.877	<0.0005	<0.005
MRPL14	0.589	3.145	14.868	<0.0005	<0.005
RMND5A	-0.557	6.709	14.861	<0.0005	<0.005
MAST3	-0.62	6.839	14.861	<0.0005	<0.005
RUNDC3A	-1.51	5.559	14.853	<0.0005	<0.005
C11orf24	0.595	3.72	14.852	<0.0005	<0.005
PSMC3	0.595	5.504	14.848	<0.0005	<0.005
ENSG00000276136	-0.858	4.216	14.841	<0.0005	<0.005
KIT	-1	1.577	14.835	<0.0005	<0.005
CSTF3	0.468	3.723	14.813	<0.0005	<0.005
DLD	0.385	5.308	14.813	<0.0005	<0.005
FANCL	0.489	4.154	14.808	<0.0005	<0.005
TMEM126A	0.648	2.674	14.781	<0.0005	<0.005
ANGPT1	-1.101	2.029	14.775	<0.0005	<0.005
VDAC1	0.535	5.492	14.769	<0.0005	<0.005
CA3-AS1	-1.189	1.2	14.756	<0.0005	<0.005
CNIH3	-1.041	1.799	14.75	<0.0005	<0.005
PRPS2	0.443	3.977	14.747	<0.0005	<0.005
FOXO1	-0.432	5.631	14.743	<0.0005	<0.005
ENSG00000260022	-4.314	8.836	14.743	<0.0005	<0.005
AP1S1	0.629	3.15	14.73	<0.0005	<0.005

PBX1	-0.872	3.43	14.729	<0.0005	<0.005
PSME3IP1	0.479	6.3	14.723	<0.0005	<0.005
SNRNP25	0.794	2.764	14.71	<0.0005	<0.005
ALDH18A1	0.574	4.329	14.699	<0.0005	<0.005
LGALS3	-0.858	7.24	14.65	<0.0005	<0.005
SAE1	0.477	5.202	14.642	<0.0005	<0.005
MYL4	-1.094	6.236	14.616	<0.0005	<0.005
RNF144B	-0.612	6.072	14.606	<0.0005	<0.005
CPSF3	0.512	4.529	14.6	<0.0005	<0.005
SERTAD2	-0.45	5.494	14.6	<0.0005	<0.005
TRAJ16	1.952	0.744	14.589	<0.0005	<0.005
SRM	0.744	4.145	14.58	<0.0005	<0.005
KIF24	1.26	0.062	14.563	<0.0005	<0.005
CXCL10	1.592	1.682	14.543	<0.0005	<0.005
SLC48A1	-0.689	3.691	14.535	<0.0005	<0.005
CALR	0.71	7.937	14.521	<0.0005	<0.005
TRIM5	0.647	4.928	14.514	<0.0005	<0.005
DCAF13	0.458	4.14	14.469	<0.0005	<0.005
TAF9	0.433	4.518	14.463	<0.0005	<0.005
GLRX3	0.508	4.188	14.463	<0.0005	<0.005
ACP2	0.579	3.824	14.463	<0.0005	<0.005
IGHV3-15	1.768	1.343	14.462	<0.0005	<0.005
BRD4	-0.438	6.676	14.448	<0.0005	<0.005
HSP90AA2P	0.724	0.834	14.445	<0.0005	<0.005
IFT25	0.518	3.52	14.444	<0.0005	<0.005
COMMD1	0.653	2.981	14.44	<0.0005	<0.005
DNAJB9	0.61	4.188	14.44	<0.0005	<0.005
HSP90AA3P	0.77	0.111	14.438	<0.0005	<0.005
RPLP0P6	0.682	4.222	14.434	<0.0005	<0.005
RAB3IL1	-1.212	1.312	14.432	<0.0005	<0.005

ENSG00000279330	-1.237	-0.36	14.41	<0.0005	<0.005
KDM4B	-0.655	6.731	14.403	<0.0005	<0.005
SUV39H2	0.765	1.622	14.395	<0.0005	<0.005
RINT1	0.547	3.308	14.392	<0.0005	<0.005
MRPL47	0.564	3.78	14.386	<0.0005	<0.005
ANPEP	-0.805	8.516	14.377	<0.0005	<0.005
SEC14L1	-0.668	8.917	14.374	<0.0005	<0.005
RPGR	-0.66	3.496	14.364	<0.0005	<0.005
LINC02555	1.241	2.54	14.363	<0.0005	<0.005
RIN2	0.775	4.006	14.358	<0.0005	<0.005
AHSP	-1.409	6.032	14.335	<0.0005	<0.005
EPRS1	0.49	6.355	14.317	<0.0005	<0.005
GFOD2	-0.552	4.02	14.309	<0.0005	<0.005
SEC61A1	0.585	7.149	14.282	<0.0005	<0.005
ASCC1	0.445	3.651	14.279	<0.0005	<0.005
SEC14L3	-1.931	2.08	14.274	<0.0005	<0.005
CHMP5	0.588	5.906	14.273	<0.0005	<0.005
SPDL1	0.809	2.205	14.273	<0.0005	<0.005
SIMALR	1.779	0.55	14.269	<0.0005	<0.005
SMTNL1	1.43	1.424	14.243	<0.0005	<0.005
MDS2	-1.268	-0.113	14.219	<0.0005	<0.005
TMEM19	0.486	4.128	14.212	<0.0005	<0.005
MAPK6	0.521	4.282	14.21	<0.0005	<0.005
BZW1P2	0.534	2.382	14.208	<0.0005	<0.005
TONSL	0.716	1.654	14.202	<0.0005	<0.005
GTF2IP4	-0.646	6.733	14.19	<0.0005	<0.005
SLC43A3	0.475	4.782	14.189	<0.0005	<0.005
ACTL6A	0.48	3.967	14.187	<0.0005	<0.005
NDUFB8	0.592	5.426	14.171	<0.0005	<0.005
ABCC13	-1.79	3.03	14.168	<0.0005	<0.005

NRAS	0.402	5.762	14.166	<0.0005	<0.005
ARAP3	-0.737	6.12	14.155	<0.0005	<0.005
C11orf98	0.537	4.461	14.129	<0.0005	<0.005
IL15RA	0.674	2.883	14.127	<0.0005	<0.005
RHBDD3	0.756	1.882	14.112	<0.0005	<0.005
CEP68	-0.504	4.13	14.111	<0.0005	<0.005
HMGCS1	0.511	4.218	14.109	<0.0005	<0.005
TMEM62	0.706	3.122	14.089	<0.0005	<0.005
DYNC1I2	0.399	5.486	14.078	<0.0005	<0.005
NDUFS3	0.568	4.305	14.077	<0.0005	<0.005
RYBP	-0.587	6.538	14.071	<0.0005	<0.005
TOB1	-0.5	5.362	14.07	<0.0005	<0.005
BEND2	-1.156	3.657	14.068	<0.0005	<0.005
IBTK	0.528	5.089	14.064	<0.0005	<0.005
EIF2B2	0.489	3.915	14.057	<0.0005	<0.005
H4C14	0.821	3.29	14.052	<0.0005	<0.005
PPIL1	0.635	3.31	14.043	<0.0005	<0.005
ARV1	0.582	2.753	14.032	<0.0005	<0.005
EMP1	1.148	1.858	14.007	<0.0005	<0.005
ENSG00000250138	1.262	3.145	13.997	<0.0005	<0.005
NDUFA6	0.603	5.159	13.993	<0.0005	<0.005
GREM2	-1.693	-0.433	13.986	<0.0005	<0.005
CEROX1	-4.162	9.726	13.981	<0.0005	<0.005
ENSG00000286288	-0.891	2.308	13.974	<0.0005	<0.005
MRPL12	0.74	3.695	13.974	<0.0005	<0.005
MRPL40	0.705	3.294	13.968	<0.0005	<0.005
ENSG00000287855	-4.445	10.517	13.959	<0.0005	<0.005
DPCD	-1.231	2.179	13.95	<0.0005	<0.005
RCC1	0.52	3.955	13.95	<0.0005	<0.005
CHEK2	0.762	1.701	13.942	<0.0005	<0.005

PDXP	1.239	2.316	13.914	<0.0005	<0.005
ITLN1	-1.648	1.887	13.9	<0.0005	<0.005
FMC1	0.701	1.354	13.893	<0.0005	<0.005
SEM1	0.639	4.305	13.89	<0.0005	<0.005
NPRL3	-0.964	6.92	13.884	<0.0005	<0.005
ALG1	0.556	3.666	13.879	<0.0005	<0.005
TMX2	0.672	3.17	13.866	<0.0005	<0.005
COMMD4	0.537	3.316	13.866	<0.0005	<0.005
SLC25A12	0.501	3.513	13.861	<0.0005	<0.005
BNIP3L	-1.072	7.957	13.854	<0.0005	<0.005
ADPRH	0.605	3.796	13.853	<0.0005	<0.005
BPNT1	0.482	3.235	13.851	<0.0005	<0.005
INKA2	-0.808	6.908	13.849	<0.0005	<0.005
IFI16	0.667	8.669	13.847	<0.0005	<0.005
LMF1	-4.088	11.749	13.813	<0.0005	<0.005
COX6C	0.578	5.203	13.806	<0.0005	<0.005
ENSG00000232807	1.202	2.019	13.792	<0.0005	<0.005
CTPS1	0.617	2.93	13.788	<0.0005	<0.005
TRIP6	0.7	1.717	13.779	<0.0005	<0.005
DPP3	0.535	3.979	13.775	<0.0005	<0.005
SAMD4A	0.96	2.268	13.765	<0.0005	<0.005
OAS3	1.558	7.961	13.745	<0.0005	<0.005
DYNC1I2P1	0.782	-0.412	13.733	<0.0005	<0.005
SNRPB	0.428	5.913	13.726	<0.0005	<0.005
GTSF1	0.834	1.198	13.721	<0.0005	<0.005
IDE	0.503	4.36	13.715	<0.0005	<0.005
RFT1	0.516	3.661	13.711	<0.0005	<0.005
COPB1	0.364	6.404	13.698	<0.0005	<0.005
ENSG00000290385	-0.955	5.243	13.696	<0.0005	<0.005
ZDHHC12-DT	0.795	1.105	13.691	<0.0005	<0.005

ENSG00000255320	-0.589	3.321	13.686	<0.0005	<0.005
WDR90	-4.428	10.43	13.683	<0.0005	<0.005
TMEM65	-0.451	4.344	13.682	<0.0005	<0.005
FCHO2	-0.683	4.446	13.68	<0.0005	<0.005
NDC1	0.561	3.538	13.68	<0.0005	<0.005
GYPC	-1.183	10.114	13.662	<0.0005	<0.005
RWDD2B	0.732	2.276	13.657	<0.0005	<0.005
MASTL	0.786	2.589	13.638	<0.0005	<0.005
ENSG00000255801	-1.995	0.244	13.635	<0.0005	<0.005
UTP4	0.513	3.566	13.628	<0.0005	<0.005
COMMD3	0.53	4.424	13.627	<0.0005	<0.005
TRIM22	0.767	8.119	13.618	<0.0005	<0.005
ADH5	0.428	5.318	13.609	<0.0005	<0.005
BRICD5	-1.312	1.732	13.609	<0.0005	<0.005
MPZL1	-0.923	6.39	13.608	<0.0005	<0.005
TM7SF3	0.375	4.79	13.59	<0.0005	<0.005
MX1	1.406	8.029	13.585	<0.0005	<0.005
SMARCB1	0.636	5.067	13.582	<0.0005	<0.005
KCNH2	-1.013	1.596	13.581	<0.0005	<0.005
OSBPL1A	-0.714	3.065	13.575	<0.0005	<0.005
WASHC3	0.503	4.2	13.554	<0.0005	<0.005
SREBF2	0.418	6.053	13.529	<0.0005	<0.005
ENSG00000277534	0.859	-0.762	13.517	<0.0005	<0.005
CDKN2A	1.121	0.69	13.515	<0.0005	<0.005
ACSL5	0.398	5.756	13.506	<0.0005	<0.005
COQ5	0.54	3.46	13.504	<0.0005	<0.005
YBX1	-0.824	10.035	13.502	<0.0005	<0.005
ATG4A	0.502	3.394	13.491	<0.0005	<0.005
CNKSR1	1.826	0.392	13.483	<0.0005	<0.005
ST13P6	-0.79	-0.561	13.481	<0.0005	<0.005

PPP1CB-DT	-1.234	-0.374	13.475	<0.0005	<0.005
TMCO3	-0.546	4.742	13.458	<0.0005	<0.005
MPG	0.511	3.928	13.457	<0.0005	<0.005
MCM5	0.568	5.242	13.45	<0.0005	<0.005
MTDH	0.466	6.665	13.444	<0.0005	<0.005
VIT	1.51	-0.121	13.44	<0.0005	<0.005
MAP7	-1.092	2.234	13.419	<0.0005	<0.005
PARM1	1.103	2.056	13.403	<0.0005	<0.005
ZNF746	-0.516	5.954	13.398	<0.0005	<0.005
NEDD4L	-1.091	3.114	13.398	<0.0005	<0.005
UBXN6	-1.055	8.877	13.395	<0.0005	<0.005
SLC12A9	-0.638	6.188	13.373	<0.0005	<0.005
CDK4	0.577	3.632	13.371	<0.0005	<0.005
IGHG2	1.23	7.045	13.363	<0.0005	<0.005
UNC13B	1.339	0.55	13.359	<0.0005	<0.005
VDAC3	0.47	5.798	13.353	<0.0005	<0.005
RNF11	-0.878	6.987	13.321	<0.0005	<0.005
ERH	0.502	5.167	13.316	<0.0005	<0.005
FOXO4	-0.667	6.613	13.306	<0.0005	<0.005
ENSG00000259529	0.604	6.647	13.303	<0.0005	<0.005
IFIT1B	-1.784	5.049	13.283	<0.0005	<0.005
KAT8	-0.391	5.341	13.272	<0.0005	<0.005
RPP25	0.83	1.806	13.266	<0.0005	<0.005
FUT8	0.674	3.517	13.258	<0.0005	<0.005
LINC01001	-0.986	5.076	13.25	<0.0005	<0.005
GDI1	-0.438	6.558	13.25	<0.0005	<0.005
USP7-AS1	-1.081	1.547	13.218	<0.0005	<0.005
CIAPIN1	0.431	3.899	13.213	<0.0005	<0.005
NUS1P1	0.629	2.382	13.195	<0.0005	<0.005
TAP1	0.612	8.407	13.194	<0.0005	<0.005

UQCRFS1P1	0.751	1.249	13.191	<0.0005	<0.005
LDHA	0.633	7.704	13.186	<0.0005	<0.005
SOX8	-4.183	9.65	13.179	<0.0005	<0.005
TRDC	0.85	5.989	13.177	<0.0005	<0.005
FBXL20	-0.468	4.96	13.177	<0.0005	<0.005
PAK1IP1	0.628	2.76	13.174	<0.0005	<0.005
ENSG00000289887	-1.182	1.86	13.163	<0.0005	<0.005
KIF20B	0.591	3.423	13.161	<0.0005	<0.005
ABCC5	-0.763	4.535	13.159	<0.0005	<0.005
PSMD5	0.373	4.457	13.155	<0.0005	<0.005
PDCD2L	0.677	1.096	13.149	<0.0005	<0.005
DSC2	-1.01	5.301	13.141	<0.0005	<0.005
YIF1A	0.579	3.288	13.139	<0.0005	<0.005
HECW2-AS1	-0.77	1.211	13.132	<0.0005	<0.005
TRIM38	0.504	6.876	13.129	<0.0005	<0.005
ICAM2	0.496	4.909	13.124	<0.0005	<0.005
ENSG00000277830	-2.686	1.413	13.122	<0.0005	<0.005
PSMG1	0.644	2.579	13.114	<0.0005	<0.005
PPP2R1B	0.452	3.963	13.111	<0.0005	<0.005
CKAP5	0.407	5.473	13.104	<0.0005	<0.005
IGLV1-47	2.314	2.345	13.103	<0.0005	<0.005
DDX1	0.499	5.131	13.102	<0.0005	<0.005
CSE1L	0.424	5.212	13.089	<0.0005	<0.005
IRS2	-1.029	6.104	13.087	<0.0005	<0.005
ENSG00000290776	-1.114	1.144	13.082	<0.0005	<0.005
TJP1	0.617	2.561	13.069	<0.0005	<0.005
PLAAT3	0.711	3.004	13.063	<0.0005	<0.005
SRP72	0.445	6.209	13.06	<0.0005	<0.005
EED	0.405	3.809	13.033	<0.0005	<0.005
KNTC1	0.725	3.086	13.031	<0.0005	<0.005

PSPH	0.835	1.678	13.004	<0.0005	<0.005
GFPT1	0.507	4.245	13.004	<0.0005	<0.005
BARD1	0.668	2.213	12.992	<0.0005	<0.005
EIPR1	0.536	3.515	12.988	<0.0005	<0.005
H2BC17	1.667	2.538	12.96	<0.0005	<0.005
BPGM	-1.83	6.709	12.955	<0.0005	<0.005
SIRPB3P	-1.006	-0.7	12.954	<0.0005	<0.005
CCDC125	-0.593	4.401	12.95	<0.0005	<0.005
ENSG00000261338	-0.745	2.741	12.945	<0.0005	<0.005
MCEE	0.768	1.557	12.944	<0.0005	<0.005
SNX4	0.396	4.052	12.942	<0.0005	<0.005
ENSG00000290937	-0.965	6.225	12.936	<0.0005	<0.005
FLAD1	0.446	3.907	12.932	<0.0005	<0.005
AHCY	0.639	4.67	12.931	<0.0005	<0.005
RNF181	0.542	5.001	12.925	<0.0005	<0.005
INE1	-1.087	-0.267	12.923	<0.0005	<0.005
XKR8	-0.564	5.717	12.922	<0.0005	<0.005
PALM2AKAP2	0.693	5.467	12.896	<0.0005	<0.005
STYXL1	0.603	3.892	12.885	<0.0005	<0.005
HAX1	0.486	5.451	12.881	<0.0005	<0.005
CENPJ	0.831	1.762	12.874	<0.0005	<0.005
SLC35F2	0.762	2.12	12.863	<0.0005	<0.005
RTCA	0.431	4.122	12.857	<0.0005	<0.005
ADAMTS5	-1.138	0.063	12.855	<0.0005	<0.005
NDUFB4	0.494	5.122	12.854	<0.0005	<0.005
PSMD8	0.478	6.051	12.854	<0.0005	<0.005
PDCD5	0.523	3.835	12.841	<0.0005	<0.005
MRPL35	0.425	3.874	12.832	<0.0005	<0.005
MRPS31	0.491	3.322	12.826	<0.0005	<0.005
PUM3	0.48	3.594	12.814	<0.0005	<0.005

HSD17B10	0.589	4.706	12.813	<0.0005	<0.005
CBX7	-0.412	5.907	12.812	<0.0005	<0.005
ENSG00000234117	-1.25	1.093	12.811	<0.0005	<0.005
BSG	-0.773	8.417	12.807	<0.0005	<0.005
MADCAM1	-1.644	0.512	12.789	<0.0005	<0.005
CENPH	0.712	1.822	12.785	<0.0005	<0.005
CDK19	-0.478	5.639	12.766	<0.0005	<0.005
MED8	0.428	4.399	12.765	<0.0005	<0.005
CYSLTR1	0.587	5.288	12.757	<0.0005	<0.005
PPP3R1	-0.414	6.642	12.752	<0.0005	<0.005
OR51AB1P	-1.089	5.843	12.731	<0.0005	<0.005
CCN3	-1.208	3.987	12.729	<0.0005	<0.005
PRR11	0.7	3.571	12.719	<0.0005	<0.005
NDUFB3	0.579	5.104	12.714	<0.0005	<0.005
C17orf80	0.466	3.588	12.712	<0.0005	<0.005
ENSG00000286342	-1.804	0.839	12.711	<0.0005	<0.005
RPF2	0.548	3.26	12.703	<0.0005	<0.005
MIR6506	-0.695	7.461	12.68	<0.0005	<0.005
RNASE2CP	1.185	0.877	12.67	<0.0005	<0.005
SPX	-1.249	2.767	12.668	<0.0005	<0.005
WNT5B	1.144	0.482	12.666	<0.0005	<0.005
OTUD1	-0.406	4.728	12.662	<0.0005	<0.005
PRIM2	0.557	2.49	12.631	<0.0005	<0.005
KDELR2	0.525	5.473	12.631	<0.0005	<0.005
CAPN5	-0.763	2.441	12.623	<0.0005	<0.005
ENSG00000291144	0.798	4.173	12.609	<0.0005	<0.005
FLT1	-1.37	1.326	12.609	<0.0005	<0.005
KIAA0232	-0.518	6.584	12.598	<0.0005	<0.005
LRRC75A	-0.763	3.38	12.597	<0.0005	<0.005
EPB41	-0.725	8.58	12.596	<0.0005	<0.005

DAD1	0.582	5.871	12.588	<0.0005	<0.005
LINC01485	1.26	-0.479	12.538	<0.0005	<0.05
ATP5PF	0.593	5.187	12.538	<0.0005	<0.05
PRDX3	0.417	5.578	12.534	<0.0005	<0.05
ATF3	0.98	1.777	12.522	<0.0005	<0.05
NNT	0.416	5.282	12.518	<0.0005	<0.05
MTFP1	0.677	2.808	12.515	<0.0005	<0.05
TSPAN18	-0.618	4.504	12.512	<0.0005	<0.05
PGAM1P8	-1.124	2.205	12.501	<0.0005	<0.05
RNF10	-0.915	8.207	12.498	<0.0005	<0.05
FDPS	0.446	5.261	12.487	<0.0005	<0.05
ANXA2	0.624	8.123	12.484	<0.0005	<0.05
TMEM147	0.584	4.071	12.473	<0.0005	<0.05
EIF2B3	0.637	2.768	12.471	<0.0005	<0.05
GTF2IP1	-0.544	6.502	12.467	<0.0005	<0.05
CCDC90B	0.399	4.456	12.467	<0.0005	<0.05
ENSG00000285851	-1.684	2.388	12.467	<0.0005	<0.05
ABTB1	-0.72	8.623	12.458	<0.0005	<0.05
LMO2	0.518	6.114	12.458	<0.0005	<0.05
PHB1	0.518	5.304	12.454	<0.0005	<0.05
TMEM150B	0.795	2.126	12.453	<0.0005	<0.05
DDX11L17	-1.422	0.837	12.452	<0.0005	<0.05
AK2	0.377	6.18	12.441	<0.0005	<0.05
ZCCHC2	0.804	6.131	12.437	<0.0005	<0.05
GATAD2B	-0.366	5.865	12.435	<0.0005	<0.05
MARCHF8	-0.943	7.781	12.433	<0.0005	<0.05
SLC14A1	-1.158	3.849	12.427	<0.0005	<0.05
SGK1	-0.57	6.464	12.426	<0.0005	<0.05
ENSG00000288796	0.925	6.434	12.423	<0.0005	<0.05
GPR42	1.384	0.011	12.417	<0.0005	<0.05

GMCL1	-0.41	5.147	12.414	<0.0005	<0.05
ALYREF	0.579	4.79	12.407	<0.0005	<0.05
SUCLA2	0.469	3.852	12.404	<0.0005	<0.05
CRIP2	-1.15	2.353	12.401	<0.0005	<0.05
ALG2	0.456	3.973	12.386	<0.0005	<0.05
CASS4	-0.665	6.056	12.384	<0.0005	<0.05
COBLL1	0.939	3.336	12.376	<0.0005	<0.05
AKTIP	-0.427	4.585	12.363	<0.0005	<0.05
ERCC6L	1.268	0.114	12.361	<0.0005	<0.05
PAGE2B	-1.52	2.32	12.344	<0.0005	<0.05
ZNF516	-0.639	5.97	12.341	<0.0005	<0.05
LINC02035	-0.638	3.232	12.333	<0.0005	<0.05
SPACA6	-1.135	0.914	12.332	<0.0005	<0.05
METTL13	0.363	4.423	12.33	<0.0005	<0.05
GNPDA1	0.431	4.218	12.329	<0.0005	<0.05
PMVK	0.611	3.766	12.322	<0.0005	<0.05
TMPO	0.417	6.354	12.317	<0.0005	<0.05
NDUFAF1	0.555	3.073	12.309	<0.0005	<0.05
LINC00570	-1.255	2.528	12.306	<0.0005	<0.05
SSR4	0.611	6.25	12.295	<0.0005	<0.05
GZMB	0.999	7.033	12.294	<0.0005	<0.05
ENSG00000267082	-1.345	1.061	12.288	<0.0005	<0.05
CNPY2-AS1	0.598	3.364	12.283	<0.0005	<0.05
IGF2BP3	0.991	1.662	12.282	<0.0005	<0.05
NOL4L	-0.458	5.382	12.277	<0.0005	<0.05
RUVBL2	0.525	4.399	12.267	<0.0005	<0.05
COPS4	0.438	4.513	12.263	<0.0005	<0.05
SDC3	1.17	2.058	12.26	<0.0005	<0.05
CCDC153	-0.952	0.719	12.258	<0.0005	<0.05
IER3IP1	0.496	4.178	12.229	<0.0005	<0.05

PRMT5	0.445	4.53	12.229	<0.0005	<0.05
MTCH2	0.497	4.478	12.226	<0.0005	<0.05
SRRD	-0.771	4.089	12.214	<0.0005	<0.05
LSM7	0.565	4.077	12.203	<0.0005	<0.05
STAB1	0.652	5.571	12.184	<0.0005	<0.05
MRPL16	0.437	3.92	12.183	<0.0005	<0.05
PRDX1	0.555	6.019	12.165	<0.0005	<0.05
ATP1B3	0.486	5.312	12.159	<0.0005	<0.05
XPC	-0.385	5.805	12.158	<0.0005	<0.05
MRPL36	0.579	3.079	12.153	<0.0005	<0.05
MRPL51	0.561	4.864	12.15	<0.0005	<0.05
DRG1	0.444	4.406	12.13	<0.0005	<0.05
TLE3	-0.616	8.011	12.12	<0.0005	<0.05
OAF	0.642	3.649	12.12	<0.0005	<0.05
LSM2	0.545	3.599	12.114	<0.0005	<0.05
SARDH	-0.888	0.658	12.107	<0.0005	<0.05
RCN3	-1.094	3.245	12.099	<0.0005	<0.05
MIR4435-2HG	0.554	5.04	12.089	<0.0005	<0.05
ENSG00000285417	0.775	2.931	12.088	<0.0005	<0.05
ENSG00000260855	-1.545	-0.365	12.082	<0.0005	<0.05
LRR1	0.651	2.614	12.079	<0.0005	<0.05
GIMAP1-GIMAP5	-1.022	4.354	12.075	<0.0005	<0.05
PARP9	0.687	7.53	12.075	<0.0005	<0.05
NUS1	0.472	4.927	12.072	<0.0005	<0.05
PDZD11	0.517	3.204	12.069	<0.0005	<0.05
ENSG00000289223	-0.883	-0.341	12.067	<0.0005	<0.05
RGL1	0.693	2.299	12.067	<0.0005	<0.05
PTMS	-0.622	4.817	12.057	<0.0005	<0.05
NDUFB7	0.632	4.687	12.057	<0.0005	<0.05
IGHG4	1.222	4.11	12.056	<0.0005	<0.05

SP140	0.458	5.763	12.049	<0.0005	<0.05
XPO5	0.385	4.332	12.049	<0.0005	<0.05
VSIG2	-0.944	3.108	12.022	<0.0005	<0.05
RHAG	-1.617	1.987	12.013	<0.0005	<0.05
ENSG00000238035	-0.907	4.973	12.006	<0.0005	<0.05
KIFAP3	0.541	4.121	12.005	<0.0005	<0.05
RHD	-1.445	1.68	11.999	<0.0005	<0.05
GJD3	0.867	0.016	11.996	<0.0005	<0.05
USP12	-0.64	5.149	11.958	<0.0005	<0.05
ZXDC	-0.426	5.387	11.958	<0.0005	<0.05
ENSG00000289273	-2.899	4.625	11.956	<0.0005	<0.05
MMP25-AS1	-1.07	6.211	11.955	<0.0005	<0.05
NUP54	0.373	4.122	11.954	<0.0005	<0.05
CLEC9A	-1.08	1.641	11.947	<0.0005	<0.05
PRMT1	0.527	5.109	11.932	<0.0005	<0.05
DPAGT1	0.518	3.699	11.924	<0.0005	<0.05
PGM3	0.689	3.171	11.909	<0.0005	<0.05
PARP10	0.533	6.179	11.909	<0.0005	<0.05
MARK3	-0.483	6.486	11.904	<0.0005	<0.05
PTOV1	-0.473	5.306	11.902	<0.0005	<0.05
ENSG00000237094	-1.144	0.497	11.893	<0.0005	<0.05
TANGO2	-0.513	5.989	11.885	<0.0005	<0.05
EXOC3L1	1.28	0.581	11.878	<0.0005	<0.05
ENSG00000291221	0.989	4.298	11.869	<0.0005	<0.05
FAM117A	-0.544	6.467	11.862	<0.0005	<0.05
IL27	1.449	-0.461	11.86	<0.0005	<0.05
TMEM97	0.865	1.541	11.853	<0.0005	<0.05
RAP1BL	0.574	2.011	11.845	<0.0005	<0.05
MT-TG	-1	5.388	11.843	<0.0005	<0.05
BATF2	1.281	3.94	11.831	<0.0005	<0.05

BOLA2-SMG1P6	0.595	3.029	11.831	<0.0005	<0.05
SLC39A7	0.41	5.264	11.831	<0.0005	<0.05
HERC5	1.259	6.134	11.82	<0.0005	<0.05
DIRAS1	-1.04	-0.291	11.808	<0.0005	<0.05
MRPL42	0.45	4.194	11.804	<0.0005	<0.05
FKBP8	-0.999	11.532	11.802	<0.0005	<0.05
CTU2	0.566	2.337	11.791	<0.0005	<0.05
AAGAB	0.378	4.838	11.791	<0.0005	<0.05
TAL1	-0.774	4.988	11.788	<0.0005	<0.05
SCFD1	0.427	5.004	11.779	<0.0005	<0.05
TBL1X	-0.63	7.011	11.775	<0.005	<0.05
KPTN	0.705	1.451	11.771	<0.005	<0.05
ENSG00000280206	-0.523	4.799	11.761	<0.005	<0.05
PPP5C	0.504	4.343	11.758	<0.005	<0.05
SLC6A8	-1.245	4.85	11.744	<0.005	<0.05
FAM225B	1.019	0.588	11.735	<0.005	<0.05
PDK3	-0.529	6.052	11.733	<0.005	<0.05
WWC3	-0.522	6.37	11.719	<0.005	<0.05
ARMC6	0.441	3.705	11.717	<0.005	<0.05
PFDN6	0.461	3.807	11.71	<0.005	<0.05
TEFM	0.568	2.07	11.696	<0.005	<0.05
MAGED1	0.62	4.063	11.689	<0.005	<0.05
HBS1L	0.404	4.655	11.676	<0.005	<0.05
FANCC	0.608	1.593	11.675	<0.005	<0.05
USO1	0.392	5.826	11.675	<0.005	<0.05
RFC5	0.589	2.895	11.652	<0.005	<0.05
NEU1	0.429	5.049	11.652	<0.005	<0.05
TENT5A	0.447	5.338	11.651	<0.005	<0.05
DCUN1D5	0.478	3.278	11.65	<0.005	<0.05
LINC00963	-0.459	5.052	11.615	<0.005	<0.05

C19orf48P	0.631	2.856	11.612	<0.005	<0.05
MAGED2	0.364	5.317	11.611	<0.005	<0.05
TRIM14	0.44	6.093	11.603	<0.005	<0.05
ACAT2	0.524	3.482	11.603	<0.005	<0.05
ENSG00000244733	-0.838	-0.93	11.594	<0.005	<0.05
C16orf54	-0.522	7.648	11.59	<0.005	<0.05
LAMP3	1.276	2.195	11.59	<0.005	<0.05
MYADM	-0.75	8.547	11.588	<0.005	<0.05
POGLUT2	1.183	-0.558	11.585	<0.005	<0.05
UBBP4	-1.011	3.152	11.58	<0.005	<0.05
MYO1B	-1.142	-0.351	11.58	<0.005	<0.05
TRIAP1	0.527	3.266	11.579	<0.005	<0.05
TRAPPC2L	0.481	3.876	11.576	<0.005	<0.05
FUZ	-0.531	3.122	11.564	<0.005	<0.05
ENSG00000260563	-0.988	0.83	11.561	<0.005	<0.05
MAP2K7	-0.385	5.558	11.548	<0.005	<0.05
CENPP	0.847	2.119	11.541	<0.005	<0.05
MIR4697	-1.173	-0.586	11.541	<0.005	<0.05
CNN2P9	-0.993	-0.82	11.541	<0.005	<0.05
FRMD4A	-0.919	2.435	11.535	<0.005	<0.05
CD1C	-0.812	3.069	11.533	<0.005	<0.05
TRUB2	0.45	3.47	11.533	<0.005	<0.05
CPPED1	-0.717	8.74	11.52	<0.005	<0.05
ARHGAP23	1.464	-0.169	11.517	<0.005	<0.05
CCNI	-0.602	8.564	11.51	<0.005	<0.05
NABP2	0.507	3.661	11.504	<0.005	<0.05
CACNA1I	-0.833	3.242	11.498	<0.005	<0.05
TBC1D22A-DT	0.703	0.044	11.497	<0.005	<0.05
RAB6B	-1.02	2.156	11.496	<0.005	<0.05
DCAF6	-0.473	5.538	11.483	<0.005	<0.05

PARP12	0.693	5.479	11.466	<0.005	<0.05
ZNF552	-0.469	3.489	11.445	<0.005	<0.05
PACS1	-0.553	7.594	11.443	<0.005	<0.05
C4orf33	0.649	3.153	11.438	<0.005	<0.05
UBE2N	0.401	5.669	11.435	<0.005	<0.05
PEG13	-0.74	1.032	11.431	<0.005	<0.05
ENSG00000279392	0.655	0.878	11.427	<0.005	<0.05
KLC3	-1.644	2.435	11.403	<0.005	<0.05
ZNF652	-0.388	6.573	11.383	<0.005	<0.05
DBNDD1	-0.892	0.816	11.382	<0.005	<0.05
MOV10	0.584	4.892	11.375	<0.005	<0.05
TXNDC15	0.409	4.852	11.37	<0.005	<0.05
RIOK3	-1.072	6.815	11.355	<0.005	<0.05
PITHD1	-0.558	5.114	11.355	<0.005	<0.05
DNAJC1	0.471	4.562	11.352	<0.005	<0.05
MSRA	-0.604	3.733	11.345	<0.005	<0.05
KLHDC7B-DT	0.988	2.732	11.342	<0.005	<0.05
TUBB	0.568	7.93	11.339	<0.005	<0.05
SPCS1	0.465	5.632	11.334	<0.005	<0.05
BAG6	-0.517	7.838	11.332	<0.005	<0.05
PPP2R5B	-0.549	4.021	11.331	<0.005	<0.05
NLRP7	0.913	0.725	11.33	<0.005	<0.05
COX6A1P2	0.753	1.866	11.328	<0.005	<0.05
ZC3H15	0.343	5.673	11.326	<0.005	<0.05
ATRIP	0.478	3.933	11.323	<0.005	<0.05
FAM225A	1.032	0.888	11.319	<0.005	<0.05
EBNA1BP2	0.564	3.864	11.313	<0.005	<0.05
PCCB	0.442	3.534	11.309	<0.005	<0.05
IGF2R	-0.714	9.749	11.294	<0.005	<0.05
RTCA-AS1	-0.79	2.302	11.288	<0.005	<0.05

MR1	0.377	5.901	11.288	<0.005	<0.05
YARS1	0.413	5.302	11.286	<0.005	<0.05
ENOPH1	0.381	3.983	11.281	<0.005	<0.05
TFEC	0.634	4.618	11.278	<0.005	<0.05
SEC23B	0.389	5.682	11.273	<0.005	<0.05
ISM1	-1.093	0.399	11.271	<0.005	<0.05
NUP205	0.446	5.141	11.268	<0.005	<0.05
GDE1	-0.534	6.306	11.265	<0.005	<0.05
ENSG00000273893	-0.756	-0.274	11.257	<0.005	<0.05
ABCF2	0.457	4.618	11.248	<0.005	<0.05
RAB39A	0.923	1.635	11.246	<0.005	<0.05
ENSG00000232499	0.813	-0.606	11.23	<0.005	<0.05
AMACR	-0.66	3.179	11.218	<0.005	<0.05
POLR1H	0.438	3.753	11.21	<0.005	<0.05
MBD6	-0.598	6.55	11.209	<0.005	<0.05
GNPAT	0.339	4.764	11.205	<0.005	<0.05
VAMP5	0.743	4.259	11.201	<0.005	<0.05
DDX11L16	-1.101	2.663	11.197	<0.005	<0.05
CMC2	0.6	3.455	11.195	<0.005	<0.05
SCAP	-0.399	5.99	11.194	<0.005	<0.05
HSPBP1	0.557	3.19	11.194	<0.005	<0.05
STARD3NL	0.392	4.792	11.188	<0.005	<0.05
FAM104A	-0.547	6.248	11.178	<0.005	<0.05
AIMP2	0.617	2.356	11.177	<0.005	<0.05
ATAD5	0.788	1.554	11.175	<0.005	<0.05
RAB11FIP1	-0.596	8.557	11.167	<0.005	<0.05
GNL3	0.511	4.813	11.166	<0.005	<0.05
CACUL1	-0.385	6.09	11.165	<0.005	<0.05
ENSG00000185839	0.642	0.622	11.155	<0.005	<0.05
IL1R1	-0.733	3.354	11.152	<0.005	<0.05

C16orf87	0.534	2.493	11.151	<0.005	<0.05
ZNF706	0.421	4.612	11.143	<0.005	<0.05
XRCC4	0.486	3.422	11.137	<0.005	<0.05
MRPL3	0.417	5.045	11.136	<0.005	<0.05
PTPMT1	0.509	4.093	11.125	<0.005	<0.05
ERN1	-0.558	6.405	11.123	<0.005	<0.05
WASF2	-0.498	8.123	11.102	<0.005	<0.05
NDUFA12	0.487	4.617	11.1	<0.005	<0.05
ENSG00000230615	-1.105	3.01	11.097	<0.005	<0.05
ACAT1	1.061	3.63	11.079	<0.005	<0.05
RAB21	-0.331	6.036	11.079	<0.005	<0.05
OAZ1	-0.661	10.454	11.079	<0.005	<0.05
GLRX	0.547	6.754	11.077	<0.005	<0.05
TMEM63B	-0.592	4.136	11.076	<0.005	<0.05
TSHZ2	-0.765	2.334	11.064	<0.005	<0.05
FANCG	0.654	2.472	11.061	<0.005	<0.05
ALOX5	-0.652	7.847	11.057	<0.005	<0.05
SNRPC	0.492	4.714	11.049	<0.005	<0.05
ANAPC11	0.495	3.851	11.043	<0.005	<0.05
DNAJC6	-1.193	1.245	11.029	<0.005	<0.05
UBA52	-0.728	10.83	11.026	<0.005	<0.05
DCUN1D1	-0.509	5.491	11.02	<0.005	<0.05
NOP56	0.388	5.685	11.004	<0.005	<0.05
ENSG00000260917	-0.732	1.853	11.003	<0.005	<0.05
RIGI	0.742	6.828	10.997	<0.005	<0.05
CD274	0.93	4.354	10.996	<0.005	<0.05
MED24	0.414	4.721	10.991	<0.005	<0.05
AZI2	0.477	4.198	10.991	<0.005	<0.05
WDFY1	0.391	5.85	10.98	<0.005	<0.05
GCSH	0.981	0.456	10.979	<0.005	<0.05

POLR2K	0.442	4.187	10.97	<0.005	<0.05
POP1	0.607	2.213	10.969	<0.005	<0.05
ZW10	0.466	3.333	10.955	<0.005	<0.05
GSTCD	0.693	1.301	10.953	<0.005	<0.05
COL4A4	1.201	0.911	10.953	<0.005	<0.05
MRC2	-1.212	2.896	10.944	<0.005	<0.05
FAM168B	-0.363	6.116	10.94	<0.005	<0.05
INPP5K	-0.377	5.844	10.937	<0.005	<0.05
FAM3C	0.537	4.019	10.934	<0.005	<0.05
YPEL3-DT	-0.771	3.662	10.91	<0.005	<0.05
RGS17	-1.045	-0.853	10.901	<0.005	<0.05
SCO2	1.026	0.874	10.9	<0.005	<0.05
CNTNAP3C	-1.579	1.9	10.898	<0.005	<0.05
PTS	0.644	1.426	10.875	<0.005	<0.05
SNORD3A	2.047	1.757	10.873	<0.005	<0.05
HNRNPA3P3	0.564	0.916	10.86	<0.005	<0.05
PDHB	0.383	5.145	10.858	<0.005	<0.05
PSMB2	0.447	5.629	10.857	<0.005	<0.05
AGPS	0.337	5.176	10.853	<0.005	<0.05
RPF1	0.383	4.751	10.852	<0.005	<0.05
EXOSC10	0.363	5.133	10.851	<0.005	<0.05
PDCD1LG2	1.203	1.048	10.84	<0.005	<0.05
ATAD3A	0.553	3.119	10.839	<0.005	<0.05
ENSG00000287958	0.788	2.578	10.823	<0.005	<0.05
NFIC	-0.419	5.185	10.822	<0.005	<0.05
IGF2	-1.754	-0.59	10.818	<0.005	<0.05
CCDC170	-0.735	2.221	10.815	<0.005	<0.05
DNAAF11	-1.163	1.277	10.813	<0.005	<0.05
FAM136A	0.478	3.901	10.812	<0.005	<0.05
NFAM1	-0.601	8.656	10.811	<0.005	<0.05

NECTIN2	1.148	2.595	10.81	<0.005	<0.05
FPR3	1.305	2.193	10.803	<0.005	<0.05
CD300E	0.761	7.09	10.798	<0.005	<0.05
STXBP1	-0.818	0.811	10.795	<0.005	<0.05
SLC20A1	0.42	5.89	10.787	<0.005	<0.05
MIR29B2CHG	-1.099	0.283	10.785	<0.005	<0.05
PPP3CC	0.411	4.434	10.779	<0.005	<0.05
CCR2	0.607	6.961	10.776	<0.005	<0.05
SHMT2	0.495	5.056	10.774	<0.005	<0.05
OGFRL1	-0.565	8.276	10.773	<0.005	<0.05
DEF8	-0.482	6.508	10.772	<0.005	<0.05
GOT1	0.603	2.769	10.765	<0.005	<0.05
H2BC19P	-1.1	1.647	10.748	<0.005	<0.05
KIF27	-0.824	3.316	10.744	<0.005	<0.05
NOMO2	0.465	6.19	10.732	<0.005	<0.05
AVEN	0.61	2.121	10.727	<0.005	<0.05
WFS1	1.024	1.566	10.726	<0.005	<0.05
ENSG00000291215	-0.833	5.111	10.724	<0.005	<0.05
PLXNA4	-0.797	1.475	10.719	<0.005	<0.05
TLCD4	-1.136	1.16	10.714	<0.005	<0.05
PHF20	-0.331	6.124	10.712	<0.005	<0.05
ICMT	0.368	4.823	10.71	<0.005	<0.05
IFITM3P1	0.908	0.592	10.688	<0.005	<0.05
MIR3667HG	0.615	2.259	10.684	<0.005	<0.05
NUTM2A-AS1	-0.43	4.805	10.676	<0.005	<0.05
RTN1	-0.76	4.166	10.676	<0.005	<0.05
ANTXR2	-0.547	7.232	10.67	<0.005	<0.05
MRPL52	0.529	3.805	10.662	<0.005	<0.05
WASHC5	0.3	5.116	10.652	<0.005	<0.05
SRP9P1	0.47	2.181	10.646	<0.005	<0.05

DNMT3A	-0.42	5.132	10.642	<0.005	<0.05
PTGS2	-0.671	5.176	10.636	<0.005	<0.05
FBXO8	0.379	3.528	10.632	<0.005	<0.05
VAMP2	-0.378	6.432	10.628	<0.005	<0.05
HSPD1P1	0.684	1.097	10.623	<0.005	<0.05
LINC02340	-0.903	1.97	10.622	<0.005	<0.05
HMGN2P41	0.453	4.954	10.622	<0.005	<0.05
SIAH1	-0.423	3.854	10.621	<0.005	<0.05
HMGA1	0.542	6.187	10.62	<0.005	<0.05
TMEM45B	-0.844	2.082	10.597	<0.005	<0.05
PEPD	0.417	4.797	10.596	<0.005	<0.05
MIR3945HG	1.049	3.736	10.589	<0.005	<0.05
FILIP1L	-1.035	-0.147	10.589	<0.005	<0.05
PDHX	0.478	3.301	10.584	<0.005	<0.05
OXSM	0.557	1.758	10.583	<0.005	<0.05
TXNL4B	0.448	3.959	10.581	<0.005	<0.05
APOO	0.65	1.457	10.578	<0.005	<0.05
NEAT1	-0.975	7.057	10.575	<0.005	<0.05
MYOM2	1.973	4.478	10.569	<0.005	<0.05
SKA2	0.482	3.894	10.56	<0.005	<0.05
SLC22A23	-0.548	2.79	10.545	<0.005	<0.05
TMCC1	-0.668	5.929	10.54	<0.005	<0.05
MRPL37	0.509	4.393	10.537	<0.005	<0.05
EI24	0.398	4.355	10.533	<0.005	<0.05
SLC44A2	-0.538	8.51	10.528	<0.005	<0.05
TIMM44	0.494	3.494	10.527	<0.005	<0.05
MLEC	0.417	6.425	10.525	<0.005	<0.05
ZNF117	-0.675	5.464	10.521	<0.005	<0.05
SAMD3	0.526	5.415	10.497	<0.005	<0.05
SLC10A7	0.495	2.767	10.484	<0.005	<0.05

SPTB	-1.416	4.898	10.479	<0.005	<0.05
LRRC41	0.348	4.416	10.468	<0.005	<0.05
CA5B	-0.409	4.586	10.464	<0.005	<0.05
COX16	0.564	3.798	10.459	<0.005	<0.05
AGK	0.455	4.233	10.458	<0.005	<0.05
PAFAH1B2	-0.346	5.924	10.458	<0.005	<0.05
SLC39A14	0.655	2.55	10.452	<0.005	<0.05
SPTA1	-1.168	2.417	10.45	<0.005	<0.05
NDUFS8	0.482	4.058	10.449	<0.005	<0.05
PANX2	-0.781	4.825	10.444	<0.005	<0.05
VRK1	0.476	3.805	10.44	<0.005	<0.05
ZNF217	-0.487	7.441	10.424	<0.005	<0.05
SLC49A4	-0.508	3.344	10.423	<0.005	<0.05
CNPY2	0.593	3.497	10.422	<0.005	<0.05
MED20	0.572	3.15	10.419	<0.005	<0.05
ENSG00000284526	-0.846	4.66	10.408	<0.005	<0.05
TRBV28	-1.114	4.327	10.403	<0.005	<0.05
EXOSC3	0.378	3.861	10.399	<0.005	<0.05
CBR1	0.53	4.012	10.399	<0.005	<0.05
PACC1	0.471	2.58	10.391	<0.005	<0.05
SLC45A4	-0.648	6.234	10.384	<0.005	<0.05
UBR1	0.443	4.993	10.383	<0.005	<0.05
NMD3	0.36	4.293	10.383	<0.005	<0.05
GPR155	-0.417	4.953	10.377	<0.005	<0.05
NUP42	0.463	3.058	10.375	<0.005	<0.05
PECAM1	-0.498	9.459	10.369	<0.005	<0.05
MIR4432HG	-0.828	0.584	10.367	<0.005	<0.05
CCS	-0.444	3.644	10.364	<0.005	<0.05
CTSE	-2.014	1.077	10.362	<0.005	<0.05
MT-TR	-0.831	4.866	10.348	<0.005	<0.05

PLRG1	0.323	4.946	10.346	<0.005	<0.05
PDE3B	-0.453	5.936	10.344	<0.005	<0.05
C19orf12	0.437	3.913	10.334	<0.005	<0.05
PGAM5	0.418	3.788	10.329	<0.005	<0.05
CPSF7	-0.408	6.17	10.329	<0.005	<0.05
CCNY	-0.481	7.029	10.32	<0.005	<0.05
GNPTAB	0.406	6.185	10.316	<0.005	<0.05
TYMP	0.586	8.322	10.315	<0.005	<0.05
KCNQ5	0.694	1.354	10.313	<0.005	<0.05
HIP1	-0.656	6.283	10.308	<0.005	<0.05
SNRPEP4	0.868	-0.677	10.304	<0.005	<0.05
MAGT1	0.38	5.869	10.292	<0.005	<0.05
ITGB1BP1	0.386	4.822	10.286	<0.005	<0.05
SUV39H1	0.472	2.978	10.283	<0.005	<0.05
ATRAID	0.434	5.21	10.28	<0.005	<0.05
POLD1	0.5	3.397	10.276	<0.005	<0.05
SHISA7	-1.241	-0.103	10.268	<0.005	<0.05
GOSR2	0.375	4.698	10.264	<0.005	<0.05
POP5	0.499	3.097	10.256	<0.005	<0.05
ENSG00000270175	0.749	-0.347	10.255	<0.005	<0.05
GNAO1	-0.655	2.317	10.253	<0.005	<0.05
YWHAZP10	0.565	1.476	10.253	<0.005	<0.05
ZFAND2A	0.484	2.834	10.25	<0.005	<0.05
TMED3	0.448	4.388	10.243	<0.005	<0.05
PI3	-1.32	5.685	10.234	<0.005	<0.05
MHENCR	-0.709	3.158	10.233	<0.005	<0.05
SEPTIN5	-1.048	4.224	10.232	<0.005	<0.05
ABHD2	-0.57	8.224	10.225	<0.005	<0.05
ENSG00000279838	-0.831	-0.167	10.223	<0.005	<0.05
APOBEC3H	0.833	0.95	10.219	<0.005	<0.05

ENSG00000269044	-0.627	2.561	10.215	<0.005	<0.05
RPL7AP70	0.781	-0.403	10.207	<0.005	<0.05
ALCAM	-0.408	4.471	10.206	<0.005	<0.05
GLE1	0.297	5.567	10.195	<0.005	<0.05
ENSG00000286022	-1.297	2.623	10.195	<0.005	<0.05
LIMD1	0.421	5.087	10.192	<0.005	<0.05
PLK3	-0.479	3.908	10.186	<0.005	<0.05
EZH1	-0.403	5.627	10.182	<0.005	<0.05
MRPL17	0.518	3.405	10.176	<0.005	<0.05
PRKY	1.818	1.669	10.175	<0.005	<0.05
ZRSR2P1	-1.186	1.1	10.171	<0.005	<0.05
MED27	0.483	2.985	10.164	<0.005	<0.05
DDIAS	0.615	2.325	10.163	<0.005	<0.05
FDX1	0.473	3.658	10.162	<0.005	<0.05
TBC1D14	-0.556	7.057	10.162	<0.005	<0.05
MRPL34	0.422	3.837	10.159	<0.005	<0.05
SRP54	0.396	5.37	10.156	<0.005	<0.05
LARS2	0.475	3.517	10.156	<0.005	<0.05
CARMIL3	1.29	1.326	10.146	<0.005	<0.05
TMEM87A	0.31	5.147	10.133	<0.005	<0.05
EXOSC1	0.446	3.922	10.129	<0.005	<0.05
SLC17A9	1.027	2.222	10.127	<0.005	<0.05
GGACT	0.602	1.856	10.117	<0.005	<0.05
ENSG00000289469	0.874	0.118	10.113	<0.005	<0.05
JSRP1	1.071	-0.65	10.107	<0.005	<0.05
ASCC3	0.407	5.19	10.103	<0.005	<0.05
TVP23B	0.379	4.238	10.102	<0.005	<0.05
PROSER3	-0.427	3.225	10.092	<0.005	<0.05
LUNAR1	-1.248	-0.465	10.087	<0.005	<0.05
ACKR1	-1.718	0.551	10.08	<0.005	<0.05

ALDOC	-0.447	3.929	10.072	<0.005	<0.05
PLIN5	-0.882	2.916	10.065	<0.005	<0.05
THEM5	-0.866	3.447	10.063	<0.005	<0.05
ITGAX	-0.734	8.243	10.062	<0.005	<0.05
MAEA	-0.341	6.004	10.056	<0.005	<0.05
TUBA1B	0.543	7.817	10.05	<0.005	<0.05
AKIP1	0.586	2.278	10.05	<0.005	<0.05
EME2	-0.417	5.575	10.048	<0.005	<0.05
LIN52	0.497	2.669	10.04	<0.005	<0.05
LRRCC1	0.629	2.063	10.02	<0.005	<0.05
ST7L	0.53	2.448	10.017	<0.005	<0.05
UQCR10	0.48	5.088	9.999	<0.005	<0.05
RFX5	0.341	5.357	9.99	<0.005	<0.05
CRBN	0.364	4.874	9.977	<0.005	<0.05
DMAC1	0.437	3.764	9.972	<0.005	<0.05
RUBCNL	-0.608	5.574	9.958	<0.005	<0.05
JARID2	-0.391	6.524	9.956	<0.005	<0.05
INTS4P1	0.54	-0.252	9.953	<0.005	<0.05
COQ8A	-0.453	5.182	9.949	<0.005	<0.05
FASLG	0.586	3.681	9.947	<0.005	<0.05
NCOA7	0.48	4.904	9.947	<0.005	<0.05
ZKSCAN5	0.369	3.73	9.944	<0.005	<0.05
PLEKHJ1	0.358	4.334	9.943	<0.005	<0.05
TNFSF10	0.634	8.29	9.934	<0.005	<0.05
POMC	0.927	0.922	9.928	<0.005	<0.05
DYNC1LI1	-0.378	5.908	9.925	<0.005	<0.05
PRKAR1A	-0.457	8.4	9.925	<0.005	<0.05
TBC1D3L	-1.058	1.487	9.917	<0.005	<0.05
CREB3	0.439	3.801	9.912	<0.005	<0.05
NUP35	0.525	1.877	9.911	<0.005	<0.05

NOC3L	0.503	3.313	9.91	<0.005	<0.05
FAM3C2P	0.727	0.623	9.906	<0.005	<0.05
PI16	-0.805	2.223	9.897	<0.005	<0.05
TRAIP	1.142	-0.297	9.896	<0.005	<0.05
XPNPEP1	0.403	5.195	9.886	<0.005	<0.05
BTN3A2	0.517	7.795	9.885	<0.005	<0.05
EXTL3	-0.506	6.067	9.884	<0.005	<0.05
FKBP3	0.423	4.24	9.878	<0.005	<0.05
MYO7A	0.917	1.165	9.877	<0.005	<0.05
PADI2	-0.79	7.371	9.875	<0.005	<0.05
DDAH2	0.482	4.822	9.874	<0.005	<0.05
PML	0.596	6.12	9.868	<0.005	<0.05
SAPCD2	1.112	1.86	9.868	<0.005	<0.05
ENSG00000287779	-1.024	-0.575	9.866	<0.005	<0.05
TLR3	0.996	1.448	9.86	<0.005	<0.05
ENSG00000289564	-1.015	0.28	9.857	<0.005	<0.05
PGGHG	-0.814	7.299	9.857	<0.005	<0.05
ZBTB8OS	0.471	4.064	9.855	<0.005	<0.05
NMI	0.589	6.852	9.854	<0.005	<0.05
GYPA	-1.311	1.73	9.851	<0.005	<0.05
HSPA5	0.6	8.147	9.851	<0.005	<0.05
GLUD2	0.635	0.511	9.851	<0.005	<0.05
EIF2AK2	0.776	6.853	9.849	<0.005	<0.05
NACC2	-0.404	6.1	9.846	<0.005	<0.05
DBF4	0.448	3.369	9.833	<0.005	<0.05
NPEPPS	-0.406	6.575	9.824	<0.005	<0.05
MXD4	-0.415	5.865	9.823	<0.005	<0.05
ENSG00000274425	-0.94	7.869	9.814	<0.005	<0.05
C1QBP	0.464	5.264	9.813	<0.005	<0.05
EMC7	0.421	4.782	9.813	<0.005	<0.05

LEO1	0.393	3.927	9.812	<0.005	<0.05
SF3B5	0.478	5.182	9.81	<0.005	<0.05
FBXL17	-0.333	4.045	9.803	<0.005	<0.05
GNAQ	-0.54	7.139	9.796	<0.005	<0.05
MICOS10	0.415	4.336	9.793	<0.005	<0.05
SORL1	-0.79	10.547	9.79	<0.005	<0.05
UQCRH	0.539	5.791	9.783	<0.005	<0.05
SEPHS1	0.458	4.249	9.775	<0.005	<0.05
UQCRFS1	0.446	5.131	9.774	<0.005	<0.05
INPP5A	-0.436	4.168	9.763	<0.005	<0.05
NUDCD1	0.516	2.939	9.758	<0.005	<0.05
LPCAT2	-0.6	6.676	9.752	<0.005	<0.05
SHISA4	-1.322	3.692	9.752	<0.005	<0.05
DOCK5	-0.664	7.107	9.752	<0.005	<0.05
YWHAZP3	0.5	2.103	9.745	<0.005	<0.05
SPRED1	-0.68	1.892	9.742	<0.005	<0.05
CRADD	0.484	2.579	9.733	<0.005	<0.05
TRIM26	0.328	5.84	9.73	<0.005	<0.05
LINC01504	0.693	1.703	9.723	<0.005	<0.05
ZNF496	0.524	3.33	9.723	<0.005	<0.05
KATNBL1	-0.575	5.086	9.719	<0.005	<0.05
ORMDL2	0.445	4.239	9.71	<0.005	<0.05
CXorf38	-0.318	5.637	9.706	<0.005	<0.05
PWP1	0.363	4.588	9.704	<0.005	<0.05
NME2	0.536	6.171	9.704	<0.005	<0.05
RAB3D	-0.565	7.631	9.692	<0.005	<0.05
PKP4	-0.475	4.037	9.683	<0.005	<0.05
GPC1	0.918	-0.354	9.677	<0.005	<0.05
COA3	0.507	3.465	9.672	<0.005	<0.05
MPHOSPH6	0.443	2.832	9.665	<0.005	<0.05

SNHG25	0.767	-0.267	9.664	<0.005	<0.05
SIK1	0.727	1.774	9.662	<0.005	<0.05
GPR162	-0.799	2.883	9.657	<0.005	<0.05
RAD54B	1.269	-0.097	9.656	<0.005	<0.05
MTLN	0.594	2.472	9.652	<0.005	<0.05
LINC02458	-0.955	0.488	9.64	<0.005	<0.05
PPIA	0.506	8.42	9.632	<0.005	<0.05
ENSG00000283782	1.862	3.3	9.628	<0.005	<0.05
ULK1	-0.448	6.294	9.627	<0.005	<0.05
MRPS24	0.559	3.988	9.612	<0.005	<0.05
HMGN4	0.316	6.267	9.609	<0.005	<0.05
FLJ40194	-0.737	-0.451	9.609	<0.005	<0.05
COPS9	0.474	3.959	9.603	<0.005	<0.05
POP4	0.408	4.561	9.601	<0.005	<0.05
NAGLU	0.455	3.234	9.601	<0.005	<0.05
EPHX1	-0.513	4.037	9.597	<0.005	<0.05
ANG	0.863	1.239	9.594	<0.005	<0.05
CD69	0.526	4.212	9.588	<0.005	<0.05
CBX3	0.315	6.403	9.587	<0.005	<0.05
GYPB	-1.682	2.008	9.577	<0.005	<0.05
GARRE1	-0.484	4.147	9.577	<0.005	<0.05
SIGLEC11	1.167	0.008	9.57	<0.005	<0.05
PIGX	-0.453	5.183	9.57	<0.005	<0.05
COX8A	0.517	5.529	9.567	<0.005	<0.05
MIRLET7BHG	-1.007	0.838	9.564	<0.005	<0.05
NCK2	-0.516	6.26	9.561	<0.005	<0.05
HPS1	-0.705	5.94	9.551	<0.005	<0.05
NDUFC2	0.457	4.941	9.549	<0.005	<0.05
KRT1	-1.678	5.407	9.548	<0.005	<0.05
AEN	0.407	3.828	9.544	<0.005	<0.05

SLC22A17	-0.803	0.619	9.539	<0.005	<0.05
SERBP1P5	0.615	0.845	9.535	<0.005	<0.05
ELP5	0.395	3.9	9.525	<0.005	<0.05
ST3GAL5	0.442	4.102	9.525	<0.005	<0.05
GALE	0.527	2.055	9.524	<0.005	<0.05
MRPL39	0.542	3.014	9.523	<0.005	<0.05
ENSG00000260257	-1.005	1.096	9.513	<0.005	<0.05
CNNM3	-0.352	4.982	9.513	<0.005	<0.05
OAZ2	-0.539	7.52	9.506	<0.005	<0.05
HSPE1P18	-1.191	-0.107	9.506	<0.005	<0.05
CAPN2	0.395	7.505	9.506	<0.005	<0.05
TMEM255A	1.21	0.505	9.505	<0.005	<0.05
DBI	0.478	5.782	9.504	<0.005	<0.05
VPS54	0.35	4.323	9.504	<0.005	<0.05
USP14	0.358	4.905	9.501	<0.005	<0.05
ATF4	0.409	7.009	9.497	<0.005	<0.05
C8orf44-SGK3	-1.708	1.702	9.487	<0.005	<0.05
PAPSS2	-0.597	2.06	9.479	<0.005	<0.05
GNA12	-0.549	5.463	9.479	<0.005	<0.05
EDA	-0.884	0.542	9.477	<0.005	<0.05
COPB2	0.375	6.857	9.47	<0.005	<0.05
NAA20	0.359	4.022	9.465	<0.005	<0.05
GCNT4	-0.521	3.344	9.458	<0.005	<0.05
TBXAS1	-0.494	7.336	9.441	<0.005	<0.05
ENSG00000272468	-0.993	1.397	9.439	<0.005	<0.05
LTBP3	-0.548	4.739	9.437	<0.005	<0.05
NFU1	0.452	3.49	9.434	<0.005	<0.05
USP28	0.42	4.991	9.43	<0.005	<0.05
TNNT1	1.191	2.544	9.427	<0.005	<0.05
POLR2H	0.487	3.056	9.427	<0.005	<0.05

VWCE	-1.373	3.621	9.426	<0.005	<0.05
PIGU	0.514	2.763	9.426	<0.005	<0.05
TPRG1L	-0.447	6.15	9.423	<0.005	<0.05
ENSG00000286555	-0.839	1.992	9.422	<0.005	<0.05
TRIM10	-0.919	2.954	9.421	<0.005	<0.05
RAPGEF2	-0.513	6.149	9.417	<0.005	<0.05
SLC43A2	-0.557	7.52	9.416	<0.005	<0.05
LINC02863	-0.753	4.837	9.415	<0.005	<0.05
PER1	-0.851	4.358	9.405	<0.005	<0.05
LINC01215	-0.494	2.972	9.405	<0.005	<0.05
CCDC138	0.88	0.681	9.405	<0.005	<0.05
ENSG00000279088	-0.575	2.714	9.404	<0.005	<0.05
CREM	0.57	3.142	9.403	<0.005	<0.05
ZDHHC16	0.465	3.564	9.402	<0.005	<0.05
PRADC1	0.608	2.35	9.402	<0.005	<0.05
ENSG00000288473	0.619	3.401	9.401	<0.005	<0.05
LIG1	0.487	3.946	9.397	<0.005	<0.05
CCT7	0.4	6.492	9.396	<0.005	<0.05
SLFN12L	-1.469	3.565	9.395	<0.005	<0.05
C8orf76	0.386	3.2	9.394	<0.005	<0.05
RAB11B	-0.375	6.199	9.392	<0.005	<0.05
CPNE5	0.708	4.399	9.385	<0.005	<0.05
CLEC2B	0.584	7.181	9.383	<0.005	<0.05
C14orf132	-0.941	1.122	9.383	<0.005	<0.05
GTF3C6	0.463	4.194	9.379	<0.005	<0.05
NOP16	0.495	3.027	9.374	<0.005	<0.05
HSPA9	0.361	6.681	9.369	<0.005	<0.05
CCR9	0.932	1.123	9.364	<0.005	<0.05
TMC5	-1.263	0.13	9.359	<0.005	<0.05
NDUFC1	0.457	3.671	9.358	<0.005	<0.05

RRAS	0.509	3.768	9.358	<0.005	<0.05
DAPK2	-0.588	5.043	9.354	<0.005	<0.05
CCDC32	0.401	4.097	9.352	<0.005	<0.05
UTP14A	0.391	4.094	9.338	<0.005	<0.05
ENSG00000293339	0.469	4.172	9.338	<0.005	<0.05
HSPH1	0.397	5.611	9.336	<0.005	<0.05
BFAR	0.3	4.912	9.333	<0.005	<0.05
HAVCR2	0.463	4.701	9.329	<0.005	<0.05
PSMC6	0.318	5.479	9.328	<0.005	<0.05
PCBP3	-0.985	0.118	9.327	<0.005	<0.05
C1GALT1C1	0.397	4.231	9.317	<0.005	<0.05
PLIN4	-0.969	2.777	9.317	<0.005	<0.05
LDHAP4	1.269	0.382	9.317	<0.005	<0.05
GADD45GIP1	0.485	4.538	9.315	<0.005	<0.05
RCAN2	1.157	0.183	9.315	<0.005	<0.05
CD180	0.591	5.275	9.306	<0.005	<0.05
CLK3	-0.371	6.18	9.301	<0.005	<0.05
RPE	0.367	3.939	9.301	<0.005	<0.05
RNASEH2A	0.618	2.573	9.299	<0.005	<0.05
MRPS18A	0.525	3.762	9.296	<0.005	<0.05
ALG3	0.46	3.528	9.294	<0.005	<0.05
SNX25	0.593	2.841	9.293	<0.005	<0.05
PPP1CB	-0.39	7.411	9.288	<0.005	<0.05
TRGJP2	1.134	3.481	9.279	<0.005	<0.05
PEX19	0.279	4.93	9.272	<0.005	<0.05
RUVBL1	0.418	4.246	9.272	<0.005	<0.05
ISCA2	0.377	3.702	9.269	<0.005	<0.05
ARSG	-0.402	4.294	9.264	<0.005	<0.05
CCT4	0.353	6.081	9.256	<0.005	<0.05
H3P6	-0.646	7.801	9.251	<0.005	<0.05

MFSD3	0.588	1.339	9.247	<0.005	<0.05
MARCKSL1	-0.444	5.742	9.235	<0.005	<0.05
CMTM2	-0.739	6.466	9.235	<0.005	<0.05
ENSG00000278330	-0.887	-0.405	9.232	<0.005	<0.05
PALD1	0.873	0.157	9.227	<0.005	<0.05
SLC35C1	0.355	4.384	9.226	<0.005	<0.05
MT-ND4	-0.894	9.784	9.22	<0.005	<0.05
TOMM5	0.488	4.19	9.218	<0.005	<0.05
TPD52	0.68	4.065	9.216	<0.005	<0.05
ATP5PD	0.461	5.636	9.207	<0.005	<0.05
EIF4E	0.327	4.749	9.196	<0.005	<0.05
GTF2I	-0.372	6.718	9.194	<0.005	<0.05
POC5	0.437	2.872	9.194	<0.005	<0.05
PSMA1	0.391	5.786	9.188	<0.005	<0.05
TMEM214	0.428	5.006	9.186	<0.005	<0.05
ENSG00000279738	-1.385	1.544	9.184	<0.005	<0.05
TRBV29-1	-1.169	0.795	9.183	<0.005	<0.05
MNT	-0.326	5.183	9.18	<0.005	<0.05
DONSON	0.662	2.211	9.177	<0.005	<0.05
IFI30	0.545	9.022	9.174	<0.005	<0.05
GLO1	0.369	4.986	9.161	<0.005	<0.05
ENSG00000241489	-0.614	7.368	9.153	<0.005	<0.05
GM2A	0.498	5.987	9.139	<0.005	<0.05
ENSG00000276649	-0.712	3.351	9.138	<0.005	<0.05
FHDC1	-1.057	1.834	9.135	<0.005	<0.05
UVSSA	-0.693	4.201	9.135	<0.005	<0.05
TUBA1C	0.456	6.17	9.124	<0.005	<0.05
CHST11	-0.455	7.453	9.123	<0.005	<0.05
TOMM40	0.439	4.027	9.122	<0.005	<0.05
HSDL2	-0.552	5.952	9.119	<0.005	<0.05

LSM3	0.49	4.713	9.116	<0.005	<0.05
ZNRF2	0.381	3.798	9.116	<0.005	<0.05
WNK1	-0.503	8.339	9.114	<0.005	<0.05
ANKRD36BP2	1.352	1.575	9.113	<0.005	<0.05
ZFYVE26	0.393	4.969	9.111	<0.005	<0.05
ENSG00000266302	-1.209	0.728	9.11	<0.005	<0.05
C1QA	1.343	2.871	9.106	<0.005	<0.05
KREMEN1	-1.051	5.629	9.105	<0.005	<0.05
HCP5	0.392	7.31	9.103	<0.005	<0.05
RAB33A	0.571	1.903	9.103	<0.005	<0.05
ENSG00000282804	-1.109	-0.267	9.101	<0.005	<0.05
ALDH4A1	0.512	1.656	9.1	<0.005	<0.05
APOBEC3C	0.439	6.845	9.098	<0.005	<0.05
TTC9	-0.598	4.622	9.094	<0.005	<0.05
UQCC2	0.547	3.326	9.093	<0.005	<0.05
SKIC8	0.395	4.028	9.093	<0.005	<0.05
XYLT1	-0.401	5.222	9.091	<0.005	<0.05
BAMBI	1.095	0.315	9.088	<0.005	<0.05
TMEM160	0.58	3.059	9.088	<0.005	<0.05
NUTM2B-AS1	-0.434	4.012	9.086	<0.005	<0.05
BZW2	0.449	4.011	9.084	<0.005	<0.05
ASCL2	0.649	3.474	9.081	<0.005	<0.05
OSBPL2	-0.38	6.312	9.078	<0.005	<0.05
ZDHHC4	0.465	3.726	9.074	<0.005	<0.05
SLC7A1	0.427	4.616	9.068	<0.005	<0.05
TRPC1	-0.876	-0.252	9.066	<0.005	<0.05
ZNF33A	-0.387	6.498	9.065	<0.005	<0.05
METAP2	0.366	5.153	9.065	<0.005	<0.05
STAT6	-0.429	8.456	9.064	<0.005	<0.05
ENSG00000280060	-0.789	0.139	9.06	<0.005	<0.05

ENSG00000289700	-0.618	3.005	9.06	<0.005	<0.05
ENSG00000288887	0.838	3.563	9.052	<0.005	<0.05
KDM7A-DT	-0.705	3.533	9.044	<0.005	<0.05
CSTF2	0.439	3.227	9.04	<0.005	<0.05
RCAN3	-0.488	5.826	9.039	<0.005	<0.05
MYH9	-0.502	10.904	9.031	<0.005	<0.05
CYB5R1	-0.384	4.772	9.026	<0.005	<0.05
RGCC	-0.483	4.387	9.023	<0.005	<0.05
MRPL54	0.522	4.167	9.023	<0.005	<0.05
SOX6	-0.954	1.328	9.022	<0.005	<0.05
PXMP2	0.667	0.935	9.021	<0.005	<0.05
MRPS26	0.397	3.509	9.014	<0.005	<0.05
CHCHD2	0.484	6.651	9.013	<0.005	<0.05
UTP11	0.387	3.662	9.013	<0.005	<0.05
ENSG00000276418	0.694	1.66	9.01	<0.005	<0.05
PKN2	-0.418	5.793	9.006	<0.005	<0.05
OTX1	-1.185	1.397	9.006	<0.005	<0.05
FHL1	-0.85	4.136	9.004	<0.005	<0.05
NOMO3	0.428	5.286	9.004	<0.005	<0.05
GTPBP4	0.35	4.692	9.003	<0.005	<0.05
HSP90AA1	0.411	8.863	8.999	<0.005	<0.05
P2RX4	0.41	3.847	8.995	<0.005	<0.05
BTK	0.362	6.334	8.993	<0.005	<0.05
LARP1B	0.616	2.674	8.993	<0.005	<0.05
TIPRL	0.275	4.953	8.988	<0.005	<0.05
NTAQ1	0.599	1.45	8.987	<0.005	<0.05
PRDM8	-0.56	3.385	8.986	<0.005	<0.05
NAA15	0.369	4.924	8.985	<0.005	<0.05
MGRN1	-0.397	6.265	8.976	<0.005	<0.05
ENSG00000288924	-0.41	4.027	8.973	<0.005	<0.05

CDC27	0.314	5.388	8.965	<0.005	<0.05
APBB2	-1.226	0.019	8.958	<0.005	<0.05
COPS2	0.338	5.285	8.957	<0.005	<0.05
ABHD5	-0.551	6.405	8.955	<0.005	<0.05
H1-10	0.424	6.351	8.954	<0.005	<0.05
B3GNTL1	-0.809	3.596	8.954	<0.005	<0.05
ERGIC2	0.382	5.362	8.953	<0.005	<0.05
BLNK	0.663	3.131	8.953	<0.005	<0.05
CPNE2	-0.543	4.665	8.95	<0.005	<0.05
PPTC7	-0.369	6.082	8.942	<0.005	<0.05
MT-CYB	-0.741	11.851	8.941	<0.005	<0.05
ENSG00000285238	-0.679	6.81	8.935	<0.005	<0.05
EHD4	0.362	4.664	8.933	<0.005	<0.05
ENSG00000267940	-1.018	0.016	8.927	<0.005	<0.05
MTURN	-0.668	6.808	8.922	<0.005	<0.05
MSL3P1	0.66	0.354	8.919	<0.005	<0.05
CYBB	0.482	9.05	8.917	<0.005	<0.05
ACSS3	-1.022	0.746	8.915	<0.005	<0.05
TSPAN2	-0.63	5.274	8.909	<0.005	<0.05
AK6	0.502	2.692	8.907	<0.005	<0.05
RPP40	0.631	0.625	8.906	<0.005	<0.05
MIR29C	-1.172	4.562	8.904	<0.005	<0.05
HSPA14	0.459	2.987	8.902	<0.005	<0.05
SNRPE	0.49	4.273	8.901	<0.005	<0.05
CYTH2	-0.291	5.028	8.897	<0.005	<0.05
RPS27P19	0.62	1.879	8.887	<0.005	<0.05
STMP1	-0.612	5.957	8.886	<0.005	<0.05
PSMD10	0.394	4.19	8.881	<0.005	<0.05
CCRL2	0.623	2.304	8.864	<0.005	<0.05
JADE1	-0.32	5.592	8.861	<0.005	<0.05

NDUFB2	0.475	4.548	8.859	<0.005	<0.05
NME7	0.54	1.568	8.857	<0.005	<0.05
GRAMD1C	-0.797	2.389	8.851	<0.005	<0.05
STX17	0.342	4.723	8.845	<0.005	<0.05
ANAPC16	-0.293	6.314	8.841	<0.005	<0.05
BTN3A3	0.494	6.926	8.84	<0.005	<0.05
COPS8	0.355	4.206	8.84	<0.005	<0.05
ERF	-0.794	5.14	8.838	<0.005	<0.05
ENSG00000289039	-1.193	3.663	8.828	<0.005	<0.05
DERL2	0.36	4.878	8.825	<0.005	<0.05
EIF6	0.454	5.413	8.817	<0.005	<0.05
APTX	0.392	3.526	8.815	<0.005	<0.05
PSMB1	0.382	6.099	8.814	<0.005	<0.05
BAG2	0.49	2.206	8.812	<0.005	<0.05
SLC35E3	-0.41	3.461	8.81	<0.005	<0.05
AMOTL1	-1.609	2.188	8.809	<0.005	<0.05
ZSCAN18	-0.657	3.38	8.809	<0.005	<0.05
STMN3	-0.488	4.391	8.804	<0.005	<0.05
NPM1P39	0.676	-0.498	8.803	<0.005	<0.05
FOXRED1	0.538	2.979	8.797	<0.005	<0.05
GRIN3A	0.672	0.845	8.796	<0.005	<0.05
ARHGEF40	-0.609	6.132	8.795	<0.005	<0.05
DENND1B	0.469	4.285	8.794	<0.005	<0.05
BMP2K	-0.525	5.823	8.782	<0.005	<0.05
FZR1	-0.352	5.294	8.779	<0.005	<0.05
ENSG00000282339	-0.87	5.655	8.776	<0.005	<0.05
MAPRE3	-0.567	2.311	8.755	<0.005	<0.05
EIF2S2	0.38	6.107	8.751	<0.005	<0.05
DGCR5	-0.871	0.217	8.751	<0.005	<0.05
PTPRN	-1.064	0.427	8.747	<0.005	<0.05

MAL	-0.573	4.252	8.745	<0.005	<0.05
BCORL1	-0.459	4.562	8.744	<0.005	<0.05
MINDY1	-0.543	6.81	8.735	<0.005	<0.05
RFFL	-0.537	6.151	8.729	<0.005	<0.05
LDAH	0.447	3.13	8.725	<0.005	<0.05
COPS5	0.288	4.958	8.721	<0.005	<0.05
PDE7B	-1.007	-0.472	8.717	<0.005	<0.05
NELL2	-0.597	5.081	8.712	<0.005	<0.05
FAM174A	-0.489	3.922	8.711	<0.005	<0.05
MAF1	-0.502	7.034	8.708	<0.005	<0.05
FAF1	0.335	4.348	8.707	<0.005	<0.05
GBP4	0.645	6.939	8.705	<0.005	<0.05
CX3CR1	0.575	8.299	8.697	<0.005	<0.05
CHMP1B	-0.355	6.629	8.69	<0.005	<0.05
COX6A1	0.521	6.342	8.674	<0.005	<0.05
CASC3	-0.48	7.442	8.673	<0.005	<0.05
TBC1D22B	-0.404	4.138	8.67	<0.005	<0.05
DNAJC7	0.354	5.812	8.669	<0.005	<0.05
THOP1	0.487	3.041	8.668	<0.005	<0.05
BEND3	0.657	0.084	8.666	<0.005	<0.05
MRPL9	0.39	4.435	8.666	<0.005	<0.05
POLR3K	0.49	3.045	8.665	<0.005	<0.05
HNRNPA1P8	0.557	0.219	8.66	<0.005	<0.05
PPA2	0.417	4.204	8.659	<0.005	<0.05
MRPL20	0.38	4.682	8.657	<0.005	<0.05
DDB2	0.399	4.106	8.655	<0.005	<0.05
FAM13A	-0.459	4.171	8.654	<0.005	<0.05
TUBBP1	0.712	-0.012	8.654	<0.005	<0.05
TSC22D3	-0.651	9.256	8.653	<0.005	<0.05
IL15	0.577	2.342	8.649	<0.005	<0.05

FFAR3	1.126	1.016	8.648	<0.005	<0.05
RETREG2	-0.455	7.057	8.641	<0.005	<0.05
MRPL33	0.377	4.111	8.635	<0.005	<0.05
TRAPPC14	-0.458	5.417	8.631	<0.005	<0.05
SIAH2	-0.765	6.292	8.626	<0.005	<0.05
ARHGAP9	-0.472	7.903	8.625	<0.005	<0.05
ATP2C1	0.273	5.203	8.624	<0.005	<0.05
ABRACL	0.372	5.253	8.622	<0.005	<0.05
BANP	-0.378	4.467	8.617	<0.005	<0.05
HDAC8	0.427	2.772	8.617	<0.005	<0.05
ENSG00000281938	-2.327	2.458	8.613	<0.005	<0.05
CLDN23	0.773	-0.167	8.612	<0.005	<0.05
IFITM3P2	1.05	1.407	8.61	<0.005	<0.05
IKBKE	0.357	4.787	8.608	<0.005	<0.05
SLC16A1	0.505	3.567	8.606	<0.005	<0.05
MEGF6	-0.703	4.524	8.604	<0.005	<0.05
CES1	0.938	4.62	8.603	<0.005	<0.05
PHLDA3	1.114	-0.766	8.602	<0.005	<0.05
RTRAF	0.381	5.781	8.601	<0.005	<0.05
ETFB	0.789	4.914	8.6	<0.005	<0.05
ABCC6	-0.842	2.015	8.598	<0.005	<0.05
LRP8	0.585	2.529	8.589	<0.005	<0.05
ST20-AS1	-0.572	2.99	8.586	<0.005	<0.05
BANF1P3	0.644	0.186	8.583	<0.005	<0.05
ZNG1DP	0.536	1.25	8.577	<0.005	<0.05
MBOAT1	-0.428	4.527	8.576	<0.005	<0.05
EIF2AK1	-0.595	7.441	8.575	<0.005	<0.05
DLC1	-1.616	-0.224	8.571	<0.005	<0.05
ENSG00000261468	-0.785	0.667	8.569	<0.005	<0.05
ZPR1	0.34	4.742	8.566	<0.005	<0.05

E2F2	0.628	4.169	8.562	<0.005	<0.05
APOL3	0.402	6.153	8.562	<0.005	<0.05
PDGFRB	0.906	2.957	8.56	<0.005	<0.05
SVIL	-0.55	6.762	8.559	<0.005	<0.05
GPX1	-0.736	8.786	8.552	<0.005	<0.05
PACSIN2	-0.501	7.059	8.531	<0.005	<0.05
ZHX1	0.312	4.369	8.524	<0.005	<0.05
GDI2P2	0.523	1.247	8.523	<0.005	<0.05
LINC02785	0.927	0.576	8.522	<0.005	<0.05
ENSG00000290018	-0.651	7.604	8.521	<0.005	<0.05
GOLM2	-0.339	6.277	8.514	<0.005	<0.05
EARS2	0.474	3.096	8.502	<0.005	<0.05
AP2A1	-0.346	6.652	8.5	<0.005	<0.05
TIFA	0.479	4.646	8.498	<0.005	<0.05
MARCHF7	-0.427	7.606	8.497	<0.005	<0.05
CYP4F3	-0.784	6.839	8.495	<0.005	<0.05
MRPS21	0.388	4.521	8.494	<0.005	<0.05
GSTO1	0.443	5.889	8.491	<0.005	<0.05
ENSG00000288882	-1.233	2.844	8.482	<0.005	<0.05
KANK2	-1.011	2.071	8.478	<0.005	<0.05
SRP68	0.365	5.344	8.469	<0.005	<0.05
RNF8	0.407	3.152	8.465	<0.005	<0.05
WDR12	0.417	3.189	8.463	<0.005	<0.05
UQCRHL	0.689	2.49	8.459	<0.005	<0.05
ZNF653	-0.518	2.093	8.455	<0.005	<0.05
IMMT	0.312	5.587	8.454	<0.005	<0.05
SHMT1	0.56	2.916	8.453	<0.005	<0.05
CLEC11A	0.675	1.304	8.453	<0.005	<0.05
IL6R	-0.527	9.023	8.448	<0.005	<0.05
REEP4	0.37	4.074	8.445	<0.005	<0.05

JAML	-0.485	9.325	8.443	<0.005	<0.05
AGER	-0.684	8.091	8.443	<0.005	<0.05
POT1	0.328	3.905	8.441	<0.005	<0.05
PIP4K2A	-0.438	8.007	8.44	<0.005	<0.05
ENSG00000288961	-0.782	-0.189	8.439	<0.005	<0.05
ITM2B	-0.531	10.549	8.429	<0.005	<0.05
NHS	-0.679	3.099	8.418	<0.005	<0.05
ZBTB44	-0.341	6.285	8.416	<0.005	<0.05
HSPA1A	-0.526	7.978	8.414	<0.005	<0.05
RXRA	-0.46	7.979	8.408	<0.005	<0.05
ILVBL	0.467	3.187	8.405	<0.005	<0.05
CNTNAP2	-1.126	1.306	8.401	<0.005	<0.05
ENSG00000283515	-1.244	2.337	8.395	<0.005	<0.05
LINC01765	-1.016	0.577	8.395	<0.005	<0.05
ENSG00000279453	-0.596	1.938	8.393	<0.005	<0.05
ACOT1	0.66	0.512	8.387	<0.005	<0.05
ERG28	0.424	3.623	8.386	<0.005	<0.05
VTA1	0.304	5.263	8.384	<0.005	<0.05
GTF2E2	0.314	4.296	8.384	<0.005	<0.05
GOLT1B	0.354	4.231	8.384	<0.005	<0.05
UBA5	0.494	3.792	8.379	<0.005	<0.05
MRPS15	0.45	3.949	8.378	<0.005	<0.05
CABIN1	-0.384	6.617	8.377	<0.005	<0.05
GPKOW	0.336	4.478	8.368	<0.005	<0.05
LINC02580	-0.604	1.585	8.366	<0.005	<0.05
INTS14	0.371	4.027	8.366	<0.005	<0.05
PDE6D	0.367	3.675	8.357	<0.005	<0.05
METTL6	0.399	2.686	8.353	<0.005	<0.05
PLPP3	0.835	-0.018	8.349	<0.005	<0.05
ENSG00000270210	-0.98	-0.462	8.347	<0.005	<0.05

NAE1	0.328	4.132	8.345	<0.005	<0.05
HIKESHI	0.393	3.526	8.343	<0.005	<0.05
FAM157D	-0.918	2.997	8.34	<0.005	<0.05
CIRBP	-0.403	6.688	8.33	<0.005	<0.05
MRPL11	0.489	4.098	8.329	<0.005	<0.05
PPFIA1	-0.337	5.395	8.324	<0.005	<0.05
SNX27	-0.464	6.127	8.323	<0.005	<0.05
KPNA4	-0.281	6.375	8.322	<0.005	<0.05
WBP2	-0.502	7.915	8.321	<0.005	<0.05
SNRPB2	0.395	5.11	8.316	<0.005	<0.05
CDK5	0.54	2.493	8.314	<0.005	<0.05
VHL	-0.268	6.009	8.311	<0.005	<0.05
PCID2	0.351	4.244	8.309	<0.005	<0.05
MSMO1	0.437	3.427	8.308	<0.005	<0.05
RBM34	0.377	4.617	8.308	<0.005	<0.05
LPAR2	-1.02	1.936	8.301	<0.005	<0.05
EPS15L1	-0.349	5.754	8.298	<0.005	<0.05
MED6	0.322	4.065	8.298	<0.005	<0.05
PSMD1	0.316	5.742	8.293	<0.005	<0.05
ENSG00000261553	1.25	2.029	8.291	<0.005	<0.05
GLUL	-0.521	9.418	8.291	<0.005	<0.05
EPPK1	-0.885	2.078	8.29	<0.005	<0.05
CD300C	0.532	3.836	8.288	<0.005	<0.05
PPP1R14B	0.486	4.061	8.287	<0.005	<0.05
ORAI2	-0.497	7.012	8.286	<0.005	<0.05
DUSP16	-0.38	5.238	8.286	<0.005	<0.05
RNF141	-0.49	6.385	8.279	<0.005	<0.05
MRS2	0.348	3.867	8.274	<0.005	<0.05
GPBAR1	0.524	4.331	8.271	<0.005	<0.05
SGTB	-0.367	4.851	8.269	<0.005	<0.05

ENSG00000284685	-0.642	1.272	8.268	<0.005	<0.05
SLC39A9	0.271	5.312	8.264	<0.005	<0.05
CYB561D2	0.385	3.617	8.257	<0.005	<0.05
UBE2O	-0.758	5.493	8.256	<0.005	<0.05
ATF4P3	0.657	0.66	8.255	<0.005	<0.05
BANF1	0.401	5.039	8.254	<0.005	<0.05
DMAC2	0.355	4.167	8.247	<0.005	<0.05
UCHL5	0.339	4.44	8.247	<0.005	<0.05
LMBRD1	-0.356	6.127	8.246	<0.005	<0.05
MRPL46	0.5	2.703	8.245	<0.005	<0.05
TMEM39A	0.391	4.056	8.241	<0.005	<0.05
RBBP7	0.328	5.508	8.237	<0.005	<0.05
DPEP3	-0.929	1.86	8.236	<0.005	<0.05
ENSG00000289690	-0.816	0.742	8.234	<0.005	<0.05
PSEN2	0.583	1.949	8.23	<0.005	<0.05
SLC9A7	-0.468	3.259	8.228	<0.005	<0.05
SNRPF	0.453	3.955	8.226	<0.005	<0.05
TMEM185B	-0.352	4.617	8.221	<0.005	<0.05
NCOA4	-0.647	9.966	8.217	<0.005	<0.05
GATA2	-0.59	4.301	8.216	<0.005	<0.05
METTL14	0.3	4.588	8.212	<0.005	<0.05
NUDT3	-0.329	6.654	8.209	<0.005	<0.05
STAT1	0.618	8.756	8.206	<0.005	<0.05
TMEM268	0.415	4.126	8.205	<0.005	<0.05
PTMAP9	0.587	0.218	8.204	<0.005	<0.05
TUBA1A	-0.493	8.267	8.203	<0.005	<0.05
GPS2	-0.442	5.369	8.2	<0.005	<0.05
FUCA2	0.44	4.391	8.199	<0.005	<0.05
PLPPR2	-0.63	5.942	8.199	<0.005	<0.05
ANKDD1A	-0.696	3.846	8.198	<0.005	<0.05

EOLA1	0.404	3.581	8.194	<0.005	<0.05
ENSG00000275993	0.652	1.934	8.192	<0.005	<0.05
COX17	0.476	3.847	8.189	<0.005	<0.05
GPN1	0.314	4.555	8.184	<0.005	<0.05
RAD23A	-0.441	5.835	8.184	<0.005	<0.05
MDK	0.979	0.269	8.182	<0.005	<0.05
TAF4	-0.317	4.539	8.176	<0.005	<0.05
CABLES1	0.913	-0.103	8.173	<0.005	<0.05
SEMA3F-AS1	-1.197	-0.047	8.168	<0.005	<0.05
NATD1	-0.554	6.334	8.165	<0.005	<0.05
BOLA1	0.464	1.876	8.163	<0.005	<0.05
ENSG00000261172	-1.03	6.066	8.163	<0.005	<0.05
GATC	0.36	5.11	8.159	<0.005	<0.05
U2	1.752	3.669	8.159	<0.005	<0.05
MTMR10	-0.386	6.333	8.154	<0.005	<0.05
MRPS36	0.401	3.571	8.152	<0.005	<0.05
DDX11L10	-1.299	3.002	8.151	<0.005	<0.05
ENSG00000289172	-1.187	6.785	8.149	<0.005	<0.05
CYB5R3	-0.41	6.512	8.144	<0.005	<0.05
PHF11	0.401	5.473	8.142	<0.005	<0.05
FAM157C	-0.929	5.836	8.138	<0.005	<0.05
ABHD17AP1	0.483	2.016	8.137	<0.005	<0.05
GFM2	0.375	3.787	8.135	<0.005	<0.05
EGFL7	0.783	2.117	8.13	<0.005	<0.05
CD55	-0.57	7.602	8.128	<0.005	<0.05
PTPA	0.378	5.773	8.127	<0.005	<0.05
RPLP0P9	0.531	4.27	8.119	<0.005	<0.05
CXCL16	-0.517	6.145	8.113	<0.005	<0.05
MTIF2	0.338	4.166	8.108	<0.005	<0.05
HDGFL3	-0.45	3.815	8.108	<0.005	<0.05

PNOC	0.775	1.765	8.107	<0.005	<0.05
TSTD2	0.504	3.257	8.1	<0.005	<0.05
PCMTD1	-0.354	6.152	8.1	<0.005	<0.05
GRSF1	0.278	5.564	8.093	<0.005	<0.05
MBOAT2	-0.614	5.197	8.089	<0.005	<0.05
UBL7	-0.404	5.404	8.087	<0.005	<0.05
NR1D2	-0.368	5.157	8.086	<0.005	<0.05
MAP2K3	-0.489	7.262	8.084	<0.005	<0.05
EPHA1	-0.586	2.706	8.081	<0.005	<0.05
MALAT1	-0.91	8.379	8.078	<0.005	<0.05
CYB5B	0.326	5.294	8.077	<0.005	<0.05
MPLKIP	0.364	3.592	8.073	<0.005	<0.05
GUSB	0.333	5.154	8.07	<0.005	<0.05
BNIP1	0.562	1.758	8.067	<0.005	<0.05
ENSG00000287632	-1.19	-0.071	8.063	<0.005	<0.05
PYGL	-0.666	8.046	8.061	<0.005	<0.05
PPIH	0.462	3.734	8.061	<0.005	<0.05
EIF2S2P4	0.551	1.154	8.06	<0.005	<0.05
CCDC86-AS1	-0.801	3.197	8.058	<0.005	<0.05
MEI1	0.545	3.327	8.058	<0.005	<0.05
CTLA4	0.734	2.137	8.051	<0.005	<0.05
MAPK1	-0.439	7.982	8.046	<0.005	<0.05
PBXIP1	-0.455	8.366	8.044	<0.005	<0.05
RASSF2	-0.507	9.411	8.043	<0.005	<0.05
LRRC57	-0.334	3.779	8.039	<0.005	<0.05
EIF1B-AS1	0.694	1.407	8.037	<0.005	<0.05
RNF44	-0.438	7.288	8.036	<0.005	<0.05
IL1RN	0.59	6.959	8.036	<0.005	<0.05
DUT	0.429	4.469	8.033	<0.005	<0.05
HSPA1L	-0.424	2.998	8.033	<0.005	<0.05

PARP6	0.346	4.662	8.03	<0.005	<0.05
BBC3	-0.398	4.759	8.029	<0.005	<0.05
ENSG00000227355	-0.749	1.586	8.029	<0.005	<0.05
MTRR	0.414	4.381	8.025	<0.005	<0.05
ENSG00000284956	-0.632	9	8.021	<0.005	<0.05
PSMB7	0.37	5.687	8.019	<0.005	<0.05
MTX2	0.529	2.279	8.019	<0.005	<0.05
LTV1	0.388	3.862	8.015	<0.005	<0.05
PROC	-1.145	-0.073	8.015	<0.005	<0.05
CEP41	0.437	2.425	8.014	<0.005	<0.05
GMDS	0.469	2.793	8.001	<0.005	<0.05
TMTC4	0.619	1.966	8.001	<0.005	<0.05
ENSG00000289278	-1.103	-0.246	7.995	<0.005	<0.05
ABCG2	-1.725	0.406	7.993	<0.005	<0.05
SOD2	-0.568	10.819	7.99	<0.005	<0.05
PAIP2B	-0.565	2.124	7.989	<0.005	<0.05
ENSG00000260729	1.173	4.281	7.987	<0.005	<0.05
POMGNT1	0.374	3.796	7.984	<0.005	<0.05
TPPP3	-0.787	2.666	7.982	<0.005	<0.05
CEP20	0.329	4.362	7.981	<0.005	<0.05
RUFY4	1.171	0.078	7.981	<0.005	<0.05
TAGAP	-0.465	8.54	7.981	<0.005	<0.05
RFC1	0.295	5.384	7.978	<0.005	<0.05
PTGER4	-0.358	5.61	7.977	<0.005	<0.05
SUCLG1	0.339	5.164	7.976	<0.005	<0.05
UBR7	0.268	4.737	7.973	<0.005	<0.05
WDR45	-0.399	6.222	7.971	<0.005	<0.05
KY	-0.568	1.613	7.969	<0.005	<0.05
MKNK2	-0.424	8.147	7.968	<0.005	<0.05
ZDHHC13	0.377	3.275	7.968	<0.005	<0.05

CSF3R	-0.584	10.82	7.957	<0.005	<0.05
ENO2	-0.406	3.83	7.956	<0.005	<0.05
POLDIP2	0.342	5.263	7.955	<0.005	<0.05
NRSN2	0.715	-0.065	7.948	<0.005	<0.05
CCDC117	0.322	4.927	7.947	<0.005	<0.05
GID4	-0.458	4.138	7.936	<0.005	<0.05
LINC03034	1.176	0.131	7.936	<0.005	<0.05
ERP44	0.346	6.399	7.935	<0.005	<0.05
SYNPO2	0.9	0.486	7.934	<0.005	<0.05
HACD3	0.358	4.204	7.93	<0.005	<0.05
GOLPH3L	0.385	3.715	7.919	<0.005	<0.05
GABPB1-AS1	-0.704	2.692	7.916	<0.005	<0.05
NEMP2	0.399	2.874	7.909	<0.005	<0.05
HAT1	0.322	4.923	7.908	<0.005	<0.05
BOP1	0.42	3.858	7.907	<0.005	<0.05
DBF4B	0.569	1.379	7.9	<0.005	<0.05
OLFML2B	0.828	-0.003	7.896	<0.005	<0.05
MMP25	-0.62	8.915	7.893	<0.005	<0.05
PARP1	0.385	6.688	7.89	<0.005	<0.05
LIN9	0.582	1.357	7.888	<0.005	<0.05
SLC38A5	-0.608	5.31	7.888	<0.005	<0.05
YOD1	-0.669	5.05	7.885	<0.005	<0.05
NOP58	0.361	5.09	7.884	<0.005	<0.05
SRPK2	-0.358	7.242	7.877	<0.005	<0.05
NDUFB9	0.421	5.389	7.875	<0.005	<0.05
CAND1	0.313	5.916	7.872	<0.005	<0.05
CCPG1	-0.502	7.604	7.865	<0.005	<0.05
RRP9	0.47	2.718	7.863	<0.005	<0.05
UFSP2	0.447	3.58	7.863	<0.005	<0.05
USP16	0.3	5.239	7.862	<0.005	<0.05

SNU13	0.357	5.79	7.86	<0.005	<0.05
JOSD2	0.511	2.886	7.856	<0.005	<0.05
ENSG00000278600	-0.88	2.943	7.856	<0.005	<0.05
ENSG00000285952	-0.802	3.589	7.853	<0.005	<0.05
TMLHEP1	0.663	-0.659	7.852	<0.005	<0.05
RECQL	0.375	5.284	7.852	<0.005	<0.05
STOML2	0.421	4.584	7.848	<0.005	<0.05
SMG8	0.309	3.841	7.843	<0.05	<0.05
KLF3	-0.379	7.462	7.841	<0.05	<0.05
ENSG00000289366	0.741	2.373	7.832	<0.05	<0.05
NQO2	-0.567	5.673	7.829	<0.05	<0.05
CCNDBP1	-0.55	6.905	7.826	<0.05	<0.05
ZNF737	-0.428	3.612	7.825	<0.05	<0.05
NDUFS7	0.362	4.445	7.82	<0.05	<0.05
MT-ND6	-0.656	10.513	7.818	<0.05	<0.05
EIF4A1P2	0.604	-0.375	7.816	<0.05	<0.05
RAD51C	0.566	2.53	7.815	<0.05	<0.05
FAM118B	0.378	3.095	7.814	<0.05	<0.05
SLC37A1	0.327	4.068	7.797	<0.05	<0.05
SNRPD2	0.437	6.025	7.796	<0.05	<0.05
CRLS1	0.371	3.948	7.795	<0.05	<0.05
VPS4B	-0.322	6.591	7.795	<0.05	<0.05
NINJ2	-0.549	4.307	7.794	<0.05	<0.05
TRA2B	0.314	6.343	7.793	<0.05	<0.05
MFAP1	0.291	4.958	7.79	<0.05	<0.05
TRGV2	0.725	1.678	7.789	<0.05	<0.05
SPINDOC	0.454	2.826	7.782	<0.05	<0.05
KPNA1	-0.327	6.072	7.779	<0.05	<0.05
UQCRC2	0.33	6.289	7.774	<0.05	<0.05
ZNF865	-0.372	4.988	7.773	<0.05	<0.05

VSIG1	-0.474	2.952	7.773	<0.05	<0.05
PHF13	-0.389	3.351	7.771	<0.05	<0.05
HES4	1.196	1.769	7.767	<0.05	<0.05
SRBD1	0.357	4.661	7.763	<0.05	<0.05
CCDC85B	0.402	4.708	7.759	<0.05	<0.05
MAGOHB	0.463	2.735	7.759	<0.05	<0.05
GPX4	-0.447	6.536	7.755	<0.05	<0.05
KCNIP2	-0.874	-0.292	7.751	<0.05	<0.05
FASTKD3	0.487	2.171	7.749	<0.05	<0.05
MRPL41	0.438	3.696	7.743	<0.05	<0.05
ZUP1	0.385	2.838	7.742	<0.05	<0.05
CCR1	0.597	7.687	7.736	<0.05	<0.05
WASHC1	-0.586	4.129	7.736	<0.05	<0.05
GPR180	0.542	2.677	7.734	<0.05	<0.05
HNRNPA1P54	0.551	-0.694	7.732	<0.05	<0.05
RB1	0.319	5.498	7.731	<0.05	<0.05
GTF2B	0.324	5.459	7.726	<0.05	<0.05
POLA2	0.546	2.775	7.725	<0.05	<0.05
BTN3A1	0.417	7.657	7.722	<0.05	<0.05
ITFG1	0.338	4.906	7.721	<0.05	<0.05
FUNDC2	-0.48	5.947	7.716	<0.05	<0.05
YBX1P10	-0.638	2.421	7.715	<0.05	<0.05
XRCC6P2	0.618	0.226	7.712	<0.05	<0.05
ID2	0.364	6.327	7.7	<0.05	<0.05
MRPL57	0.392	3.839	7.699	<0.05	<0.05
LYPD2	-1.489	0.31	7.693	<0.05	<0.05
MRPL23	0.505	4.024	7.692	<0.05	<0.05
DHX40	-0.291	5.043	7.681	<0.05	<0.05
PTPN22	0.368	5.241	7.672	<0.05	<0.05
LMTK3	-0.527	1.752	7.666	<0.05	<0.05

ENSG00000279267	-0.942	0.59	7.664	<0.05	<0.05
ENSG00000289768	1.35	4.294	7.654	<0.05	<0.05
TIMM21	0.499	2.509	7.653	<0.05	<0.05
PRIM1	0.608	2.728	7.652	<0.05	<0.05
TMEM256	0.5	3.199	7.648	<0.05	<0.05
MARCHF1	0.409	6.198	7.644	<0.05	<0.05
EFCAB6	0.543	1.847	7.637	<0.05	<0.05
H2AC20	0.732	0.522	7.633	<0.05	<0.05
NORAD	-0.374	7.874	7.632	<0.05	<0.05
HS3ST3B1	-0.407	3.85	7.63	<0.05	<0.05
CREB1	-0.34	6.694	7.628	<0.05	<0.05
NDUFS4	0.452	3.666	7.625	<0.05	<0.05
SEC14L5	-0.864	2.044	7.619	<0.05	<0.05
FARSA	0.378	4.57	7.619	<0.05	<0.05
SEC31A	0.295	6.399	7.618	<0.05	<0.05
ENSG00000287255	-1.037	1.046	7.614	<0.05	<0.05
MACROD1	0.59	0.109	7.613	<0.05	<0.05
SUSD6	-0.459	7.366	7.609	<0.05	<0.05
MYO1F	-0.453	9.845	7.609	<0.05	<0.05
LSM1	0.386	4.585	7.608	<0.05	<0.05
DYRK1B	-0.361	4.362	7.602	<0.05	<0.05
CENPO	0.813	2.112	7.602	<0.05	<0.05
ENSG00000293293	0.644	-0.561	7.6	<0.05	<0.05
YIPF5	0.285	4.807	7.596	<0.05	<0.05
ADHFE1	-0.944	1.368	7.592	<0.05	<0.05
GPR18	0.382	3.685	7.586	<0.05	<0.05
CD2AP	0.362	4.801	7.584	<0.05	<0.05
ENSG00000286760	0.837	-0.108	7.583	<0.05	<0.05
LINC00891	0.744	0.356	7.582	<0.05	<0.05
TMEM43	-0.373	6.903	7.58	<0.05	<0.05

ENSG00000274272	-0.87	5.635	7.579	<0.05	<0.05
ATG16L2	-0.835	7.96	7.577	<0.05	<0.05
NTHL1	0.509	2.098	7.576	<0.05	<0.05
MIR646HG	-0.82	1.762	7.574	<0.05	<0.05
RAB18	-0.329	6.171	7.569	<0.05	<0.05
CYP51A1	1.214	3.342	7.566	<0.05	<0.05
ZC3HC1	0.414	2.41	7.561	<0.05	<0.05
LAGE3	0.516	2.535	7.56	<0.05	<0.05
CCSAP	-0.318	4.651	7.553	<0.05	<0.05
TMEM119	1.02	0.814	7.553	<0.05	<0.05
ENSG00000289346	-0.502	2.699	7.551	<0.05	<0.05
ACOX1	-0.46	6.453	7.549	<0.05	<0.05
MICALL2	-0.68	0.585	7.548	<0.05	<0.05
GSK3B	-0.335	6.473	7.545	<0.05	<0.05
IPO4	0.952	2.723	7.537	<0.05	<0.05
MRTO4	0.455	3.605	7.534	<0.05	<0.05
MBOAT7	-0.532	8.812	7.53	<0.05	<0.05
ARF4	0.412	5.914	7.529	<0.05	<0.05
COL18A1	-0.637	5.944	7.528	<0.05	<0.05
IFNG	0.674	1.589	7.526	<0.05	<0.05
TBK1	0.272	5.482	7.525	<0.05	<0.05
MMP24OS	-0.413	5.322	7.522	<0.05	<0.05
RPL15P2	0.556	1.779	7.519	<0.05	<0.05
CNOT3	-0.28	5.655	7.519	<0.05	<0.05
CDK18	0.639	0.735	7.51	<0.05	<0.05
BMERB1	-0.657	2.661	7.507	<0.05	<0.05
SHLD2	0.324	4.455	7.503	<0.05	<0.05
XPOT	0.283	5.463	7.499	<0.05	<0.05
MRPL49	0.269	4.955	7.497	<0.05	<0.05
CHST7	-0.441	3.541	7.495	<0.05	<0.05

LRRC40	0.412	3.204	7.493	<0.05	<0.05
CDC23	0.333	4.021	7.49	<0.05	<0.05
TNRC6C	-0.384	5.49	7.478	<0.05	<0.05
NUAK2	-0.447	6.581	7.476	<0.05	<0.05
SMIM20	0.437	3.051	7.475	<0.05	<0.05
IDH3A	0.417	4.574	7.475	<0.05	<0.05
ENSG00000272501	-0.548	3.811	7.474	<0.05	<0.05
KIAA0319	-0.71	1.725	7.468	<0.05	<0.05
H4C12	0.644	0.077	7.468	<0.05	<0.05
E2F1	0.896	2.923	7.468	<0.05	<0.05
UBASH3B	0.284	5.239	7.468	<0.05	<0.05
SMIM15	0.353	4.305	7.461	<0.05	<0.05
TRAJ49	-1.119	2.462	7.46	<0.05	<0.05
FICD	0.496	2.187	7.458	<0.05	<0.05
NDUFB11	0.424	5.029	7.458	<0.05	<0.05
ATP2B1	-0.419	6.907	7.458	<0.05	<0.05
CBX3P9	0.665	-0.485	7.456	<0.05	<0.05
CNDP2	0.344	6.294	7.456	<0.05	<0.05
OSBPL8	-0.465	7.845	7.455	<0.05	<0.05
CCR8	-0.97	0.309	7.455	<0.05	<0.05
C1orf216	0.407	3.005	7.453	<0.05	<0.05
ATG9B	-0.596	1.713	7.452	<0.05	<0.05
HAUS8	0.414	2.515	7.452	<0.05	<0.05
SNX18	-0.363	6.72	7.448	<0.05	<0.05
CCT5	0.365	6.925	7.442	<0.05	<0.05
C1orf43	0.341	6.655	7.44	<0.05	<0.05
TXNDC17	0.406	4.049	7.439	<0.05	<0.05
CWF19L1	0.269	4.634	7.435	<0.05	<0.05
TSPAN7	-1.461	-0.165	7.433	<0.05	<0.05
SHFL	0.376	5.293	7.427	<0.05	<0.05

SLC35A5	0.325	4.725	7.426	<0.05	<0.05
THEMIS2	-0.406	8.085	7.42	<0.05	<0.05
MTND2P28	-0.698	9.785	7.419	<0.05	<0.05
TRIM39	-0.289	4.42	7.419	<0.05	<0.05
GBP5	0.636	8.74	7.417	<0.05	<0.05
COX18	0.401	3.095	7.413	<0.05	<0.05
RASSF3	-0.418	8.258	7.412	<0.05	<0.05
OMA1	1.287	1.7	7.408	<0.05	<0.05
CXCL6	-0.86	-0.392	7.405	<0.05	<0.05
PLA2G15	0.459	3.024	7.404	<0.05	<0.05
MARCHF6	-0.348	6.521	7.404	<0.05	<0.05
SLX9	0.435	3.11	7.402	<0.05	<0.05
ZNF79	0.359	3.279	7.394	<0.05	<0.05
DHFR	0.729	4.703	7.393	<0.05	<0.05
TATDN2	0.284	6.192	7.39	<0.05	<0.05
TAP2	0.49	7.241	7.375	<0.05	<0.05
RASGRP4	-0.495	7.157	7.375	<0.05	<0.05
MVB12A	0.459	4.052	7.374	<0.05	<0.05
AQP3	0.525	5.533	7.371	<0.05	<0.05
INTS12	0.365	3.392	7.37	<0.05	<0.05
AHSA1	0.269	5.578	7.369	<0.05	<0.05
TRAPPC2B	0.579	1.082	7.364	<0.05	<0.05
ATP5MF	0.403	5.576	7.363	<0.05	<0.05
UBE2E1	0.341	4.588	7.363	<0.05	<0.05
IARS1	0.349	5.148	7.362	<0.05	<0.05
NDUFA13	0.436	5.646	7.361	<0.05	<0.05
INPP4B	-0.488	5.151	7.358	<0.05	<0.05
ATP2A2	0.333	6.07	7.354	<0.05	<0.05
UBASH3A	-0.396	4.365	7.353	<0.05	<0.05
FKBP7	0.852	-0.542	7.351	<0.05	<0.05

POLR2L	0.452	4.585	7.351	<0.05	<0.05
CRACDL	-0.702	1.455	7.351	<0.05	<0.05
TBC1D30	-0.571	1.916	7.349	<0.05	<0.05
ENSG00000243273	0.959	0.998	7.348	<0.05	<0.05
UNC119	-0.346	5.917	7.345	<0.05	<0.05
ATOX1	0.327	4.112	7.343	<0.05	<0.05
ENSG00000289382	0.704	1.585	7.343	<0.05	<0.05
NIBAN1	-0.65	9.43	7.342	<0.05	<0.05
FAM177A1	-0.313	4.246	7.341	<0.05	<0.05
MIX23	0.557	2.089	7.337	<0.05	<0.05
SSB	0.312	5.601	7.336	<0.05	<0.05
TNFRSF10D	-0.56	1.826	7.334	<0.05	<0.05
KAT2B	-0.517	6.552	7.334	<0.05	<0.05
LRRC20	0.61	1.181	7.331	<0.05	<0.05
GABPB1-IT1	-0.494	3.421	7.329	<0.05	<0.05
SFXN4	0.556	2.12	7.327	<0.05	<0.05
PDHA1	0.318	4.658	7.326	<0.05	<0.05
ENSG00000279884	-0.726	3.668	7.326	<0.05	<0.05
RENBP	-0.909	2.476	7.323	<0.05	<0.05
DDX18	0.301	5.973	7.312	<0.05	<0.05
NGLY1	0.297	4.827	7.31	<0.05	<0.05
ENSG00000255508	-0.682	3.901	7.309	<0.05	<0.05
GNGT2	0.495	3.722	7.308	<0.05	<0.05
AHCTF1	-0.466	6.764	7.302	<0.05	<0.05
ADAM28	-0.521	3.819	7.296	<0.05	<0.05
LIMA1	0.386	3.816	7.296	<0.05	<0.05
CAPG	0.453	6.411	7.295	<0.05	<0.05
CLEC4A	0.469	5.608	7.295	<0.05	<0.05
SULF2	-0.565	7.396	7.292	<0.05	<0.05
SLC19A1	-0.45	6.084	7.291	<0.05	<0.05

HNRNPAB	0.349	5.701	7.286	<0.05	<0.05
ENSG00000284292	-1.673	2.961	7.285	<0.05	<0.05
CYB561D1	-0.342	4.693	7.285	<0.05	<0.05
RNASET2	-0.515	8.008	7.284	<0.05	<0.05
GAL3ST4	-0.669	1.328	7.276	<0.05	<0.05
LTB	-0.452	7.97	7.27	<0.05	<0.05
DHRS7	-0.408	6.68	7.27	<0.05	<0.05
IKBIP	-0.516	5.638	7.269	<0.05	<0.05
RNY1	1.575	4.819	7.267	<0.05	<0.05
CRTC2	-0.279	5.926	7.265	<0.05	<0.05
LY75	-0.503	6.307	7.265	<0.05	<0.05
TCF4	0.435	4.327	7.261	<0.05	<0.05
CBX1	-0.293	5.477	7.261	<0.05	<0.05
TSHZ3	-0.503	3.972	7.257	<0.05	<0.05
TTC38	0.606	5.081	7.257	<0.05	<0.05
GNPNAT1	0.408	3.267	7.255	<0.05	<0.05
KLRC3	1.079	3.757	7.254	<0.05	<0.05
TRAFD1	0.414	6.702	7.251	<0.05	<0.05
PCBP2	-0.41	8.77	7.244	<0.05	<0.05
H2BC9	0.865	4.239	7.235	<0.05	<0.05
MRPS14	0.346	3.787	7.229	<0.05	<0.05
TALDO1	-0.543	9.03	7.227	<0.05	<0.05
SLC30A9	0.297	4.547	7.226	<0.05	<0.05
NABP1	-0.647	6.846	7.226	<0.05	<0.05
EXOSC7	0.443	3.044	7.223	<0.05	<0.05
BRWD3	-0.489	6.149	7.222	<0.05	<0.05
EDEM1	0.459	6.388	7.221	<0.05	<0.05
AGO4	-0.486	7.236	7.221	<0.05	<0.05
DPEP2	-0.523	6.88	7.219	<0.05	<0.05
ATG5	0.289	4.504	7.217	<0.05	<0.05

IL13RA1	-0.599	7.678	7.216	<0.05	<0.05
MIS12	0.348	3.901	7.215	<0.05	<0.05
ENSG00000249806	-0.789	-0.668	7.202	<0.05	<0.05
CASP1	0.385	7.688	7.202	<0.05	<0.05
ATP8B4	0.401	3.634	7.198	<0.05	<0.05
MRPL24	0.42	3.843	7.197	<0.05	<0.05
TAGLN2	-0.56	10.188	7.196	<0.05	<0.05
MPV17L2	0.461	2.15	7.196	<0.05	<0.05
TRPV2	0.287	5.301	7.192	<0.05	<0.05
STRN4	-0.311	6.122	7.191	<0.05	<0.05
CD200	-0.609	1.842	7.19	<0.05	<0.05
MYO7B	-0.805	2.872	7.19	<0.05	<0.05
ENSG00000266709	0.835	0.352	7.189	<0.05	<0.05
PBDC1	0.399	3.647	7.188	<0.05	<0.05
RWDD2A	0.581	1.301	7.186	<0.05	<0.05
PNO1	0.421	3.093	7.186	<0.05	<0.05
LINC02979	-0.971	-0.613	7.183	<0.05	<0.05
MCCC2	0.347	4.259	7.181	<0.05	<0.05
SH3BGRL2	-0.772	5.453	7.178	<0.05	<0.05
REPS2	-0.602	5.564	7.176	<0.05	<0.05
ENSG00000268170	-1.136	0.905	7.174	<0.05	<0.05
SLC25A17	0.447	2.637	7.174	<0.05	<0.05
NUDT4	-0.398	5.496	7.17	<0.05	<0.05
AGTPBP1	-0.345	6.582	7.168	<0.05	<0.05
GNAS	-0.492	9.989	7.164	<0.05	<0.05
SLC8A1	-0.486	5.22	7.163	<0.05	<0.05
PSMC1P1	0.438	2.352	7.163	<0.05	<0.05
TYW1	0.335	3.714	7.162	<0.05	<0.05
ENSG00000227598	-0.879	1.888	7.16	<0.05	<0.05
NRGN	-0.676	8.609	7.157	<0.05	<0.05

SLCO3A1	-0.356	6.516	7.153	<0.05	<0.05
CRISPLD2	-0.649	6.803	7.146	<0.05	<0.05
ENSG00000290034	-0.769	3.084	7.144	<0.05	<0.05
LACTB2	0.434	2.27	7.14	<0.05	<0.05
PIWIL4	0.646	1.115	7.136	<0.05	<0.05
LAX1	0.398	5.229	7.131	<0.05	<0.05
ZNF740	0.295	6.639	7.131	<0.05	<0.05
ENSG00000279722	-0.637	0.397	7.127	<0.05	<0.05
SIRPG	-0.566	3.836	7.124	<0.05	<0.05
MFSD13A	0.478	2.388	7.121	<0.05	<0.05
DUSP3	0.356	5.254	7.121	<0.05	<0.05
ABCE1	0.321	5.159	7.118	<0.05	<0.05
LINC00926	-0.892	4.079	7.117	<0.05	<0.05
MRPS34	0.45	4.897	7.1	<0.05	<0.05
MSANTD7	0.325	3.595	7.097	<0.05	<0.05
NANS	0.333	5.064	7.093	<0.05	<0.05
ZDHHC7	-0.287	6.346	7.085	<0.05	<0.05
DDX21	0.303	6.415	7.084	<0.05	<0.05
HPS5	0.312	4.306	7.082	<0.05	<0.05
TBC1D2	0.345	4.558	7.073	<0.05	<0.05
ZNF92	0.349	4.166	7.073	<0.05	<0.05
TRNAU1AP	0.357	3.453	7.072	<0.05	<0.05
IL12RB2	0.693	2.528	7.071	<0.05	<0.05
MESD	0.328	5.049	7.071	<0.05	<0.05
RMDN3	0.362	3.562	7.06	<0.05	<0.05
NAXE	0.376	4.958	7.059	<0.05	<0.05
ENSG00000289130	-0.728	4.356	7.058	<0.05	<0.05
NUP107	0.329	4.204	7.055	<0.05	<0.05
LINC01036	-1.163	2.556	7.055	<0.05	<0.05
RBM45	0.415	2.383	7.054	<0.05	<0.05

MTCH1	-0.279	6.183	7.047	<0.05	<0.05
ENSG00000279744	-0.948	0.124	7.043	<0.05	<0.05
CHCHD1	0.397	3.772	7.042	<0.05	<0.05
CALCOCO1	-0.327	6.656	7.041	<0.05	<0.05
CCT6A	0.3	6.236	7.041	<0.05	<0.05
LINC01480	0.912	0.335	7.038	<0.05	<0.05
ADGRE5	-0.483	9.531	7.031	<0.05	<0.05
FANCM	0.577	1.814	7.028	<0.05	<0.05
ROGDI	-0.448	4.056	7.028	<0.05	<0.05
ENSG00000261471	-0.793	1.805	7.027	<0.05	<0.05
FARS2	0.44	2.668	7.027	<0.05	<0.05
TPMT	0.392	4.383	7.026	<0.05	<0.05
CENPL	0.476	1.902	7.023	<0.05	<0.05
CPM	-0.503	3.3	7.023	<0.05	<0.05
CYB5R4	-0.389	6.706	7.016	<0.05	<0.05
TMEM135	0.463	2.567	7.003	<0.05	<0.05
HMGN2P5	0.394	3.185	7.003	<0.05	<0.05
CHCHD2P2	0.811	-0.802	6.995	<0.05	<0.05
LINC01094	-0.754	1.819	6.991	<0.05	<0.05
HIF1AN	-0.249	5.909	6.991	<0.05	<0.05
ALS2CL	-0.869	2.056	6.984	<0.05	<0.05
GDAP1	0.516	1.578	6.984	<0.05	<0.05
BUB3	0.279	5.724	6.983	<0.05	<0.05
E2F5	0.535	3.56	6.982	<0.05	<0.05
ENSG00000264772	0.834	3.432	6.981	<0.05	<0.05
KAZN	-0.954	2.183	6.97	<0.05	<0.05
GZMA	0.494	6.807	6.967	<0.05	<0.05
MT-TL2	-0.675	5.019	6.966	<0.05	<0.05
CENPBD2P	-0.286	4.377	6.963	<0.05	<0.05
TMEM25	-0.474	1.557	6.962	<0.05	<0.05

NEK7	-0.341	6.457	6.961	<0.05	<0.05
PITPNC1	-0.301	5.637	6.961	<0.05	<0.05
TDG	0.333	4.449	6.959	<0.05	<0.05
TEF	-0.411	3.138	6.956	<0.05	<0.05
SNN	-0.441	7.641	6.955	<0.05	<0.05
ELOF1	-0.527	5.323	6.95	<0.05	<0.05
YARS2	0.409	2.527	6.947	<0.05	<0.05
QRSL1	0.357	3.768	6.947	<0.05	<0.05
ENSG00000261915	-1.218	2.396	6.944	<0.05	<0.05
SLC35E4	0.64	0.693	6.941	<0.05	<0.05

Table S4: Differentially expressed genes found comparing acute individuals and control group.

Supplementary Table S4: Differentially expressed genes found comparing acute individuals and control group.

genes	logFC	logCPM	LR	PValue	FDR
GNPTG	-5.59	7.964	95.078	<0.0001	<0.0001
UBE2I	-5.668	9.139	95.05	<0.0001	<0.0001
UNKL	-6.507	7.256	91.611	<0.0001	<0.0001
UQCC4	-5.705	5.16	86.282	<0.0001	<0.0001
BAIAP3	-7.328	7.498	85.146	<0.0001	<0.0001
CLCN7	-5.574	8.226	84.958	<0.0001	<0.0001
HSD11B1L	-5.551	2.188	76.463	<0.0001	<0.0001
TSR3	-3.592	5.974	65.214	<0.0001	<0.0001
FAM234A	-6.144	10.147	46.024	<0.0001	<0.0001
CACNA1H	-10.774	8.698	45.459	<0.0001	<0.0001
PGAP6	-4.705	10.436	45.376	<0.0001	<0.0001
NME4	-5.607	9.271	44.885	<0.0001	<0.0001
STUB1	-4.89	8.42	44.744	<0.0001	<0.0001
RHOT2	-5.495	10.243	44.578	<0.0001	<0.0001
METTL26	-5.479	8.704	44.323	<0.0001	<0.0001
AXIN1	-5.866	11.789	43.298	<0.0001	<0.0001
WDR24	-5.996	9.597	42.977	<0.0001	<0.0001
HBQ1	-4.531	7.718	42.555	<0.0001	<0.0001
FBXL16	-6.636	10.14	42.509	<0.0001	<0.0001
JMJD8	-4.736	8.822	39.348	<0.0001	<0.0001
MRPL28	-5.872	8.485	39.176	<0.0001	<0.0001
MIR3176	-6.503	10.88	39.029	<0.0001	<0.0001
ANTKMT	-5.973	8.826	38.619	<0.0001	<0.0001
CAPN15	-5.222	10.466	38.428	<0.0001	<0.0001
RAB11FIP3	-5.993	10.549	38.238	<0.0001	<0.0001
PIGQ	-6.101	10.843	37.681	<0.0001	<0.0001
CIAO3	-6.316	10.718	37.663	<0.0001	<0.0001

RAB40C	-6.46	11.991	37.215	<0.0001	<0.0001
MCRIP2	-6.291	10.501	35.989	<0.0001	<0.0001
LUC7L	-6.239	11.986	35.035	<0.0001	<0.0001
RPUSD1	-5.728	9.043	34.708	<0.0001	<0.0001
METRN	-6.408	9.713	33.919	<0.0001	<0.0001
HAGHL	-6.27	9.128	32.79	<0.0001	<0.0001
DECR2	-6.165	9.316	32.186	<0.0001	<0.0001
ENSG00000261659	-6.419	9.578	30.839	<0.0001	<0.0001
CHTF18	-6.332	9.979	27.262	<0.0001	<0.0001
AK1	-2.549	3.439	26.298	<0.0001	<0.0005
WFIKKN1	-6.518	9.544	24.712	<0.0001	<0.0005
ENSG00000260496	-6.677	7.639	24.542	<0.0001	<0.0005
MEOX1	-1.893	0.787	24.509	<0.0001	<0.0005
ARHGAP42	2.695	0.992	24.485	<0.0001	<0.0005
IGLV3-25	4.804	5.111	24.092	<0.0001	<0.0005
CCDC78	-6.367	9.65	24.063	<0.0001	<0.0005
ENSG00000293232	-6.629	8.616	23.992	<0.0001	<0.0005
RNASE1	4.041	1.178	23.923	<0.0001	<0.0005
CEROX1	-6.652	9.726	23.702	<0.0001	<0.0005
NHLRC4	-6.461	8.347	23.456	<0.0001	<0.0005
IGHV2-5	3.881	4.39	23.181	<0.0001	<0.0005
IGHG3	2.533	5.836	23.026	<0.0001	<0.005
CD38	1.979	4.817	22.679	<0.0001	<0.005
ENSG00000260022	-6.472	8.836	22.667	<0.0001	<0.005
OTOF	4.355	2.601	22.545	<0.0001	<0.005
ENSG00000292432	-6.789	7.629	21.949	<0.0001	<0.005
Y_RNA	-3.41	5.589	21.674	<0.0001	<0.005
TOP2A	2.067	3.464	21.427	<0.0001	<0.005
MKI67	2.18	4.759	21.334	<0.0001	<0.005
DTL	2.363	1.904	21.174	<0.0001	<0.005

ENSG00000287855	-6.595	10.517	21.114	<0.0001	<0.005
IGHV4-61	3.594	4.125	21.032	<0.0001	<0.005
SOX8	-6.405	9.65	20.906	<0.0001	<0.005
EPHB2	2.146	2.483	20.905	<0.0001	<0.005
IGHV1-69D	4.683	4.698	20.798	<0.0001	<0.005
TESC	-1.633	6.567	20.795	<0.0001	<0.005
ENSG00000289474	-2.824	3.824	20.546	<0.0001	<0.005
HVCN1	-0.882	5.965	20.287	<0.0001	<0.005
IGLV3-1	4.377	7.473	20.221	<0.0001	<0.005
LMF1	-5.921	11.749	20.03	<0.0001	<0.005
IGHM	4.26	10.158	19.866	<0.0001	<0.005
IGLV6-57	5.197	4.675	19.778	<0.0001	<0.005
IGKV4-1	3.476	6.406	19.707	<0.0001	<0.005
MCM4	1.526	3.879	19.692	<0.0001	<0.005
IGHV5-51	4.596	5.993	19.632	<0.0001	<0.005
GBP3	1.688	5.065	19.549	<0.0001	<0.005
IGHV3-21	3.003	4.407	19.54	<0.0001	<0.005
SHCBP1	2.498	2.303	19.29	<0.0001	<0.005
TXNDC5	2.812	7.874	19.258	<0.0001	<0.005
BAG1	-1.691	8.208	19.148	<0.0001	<0.005
CENPE	1.868	1.975	18.976	<0.0001	<0.005
IGKV2D-28	4.264	5.041	18.975	<0.0001	<0.005
C13orf46	2.272	0.642	18.96	<0.0001	<0.005
IGHV1-69	4.576	4.389	18.794	<0.0001	<0.005
ATAD2	0.998	4.081	18.599	<0.0001	<0.005
STIL	1.873	1.239	18.583	<0.0001	<0.005
NT5DC2	2.204	2.416	18.481	<0.0001	<0.005
IGHV3-30	3.48	5.633	18.343	<0.0001	<0.005
IGKV1D-33	2.575	3.14	18.277	<0.0001	<0.005
IGHV3-48	3.325	4.257	18.243	<0.0001	<0.005

IGHV4-39	3.035	4.49	18.231	<0.0001	<0.005
RFLNB	-1.375	6.994	18.201	<0.0001	<0.005
ORC1	2.132	1.221	18.191	<0.0001	<0.005
IGLV2-23	3.041	5.2	18.132	<0.0001	<0.005
NCAPG	2.185	2.282	18.02	<0.0001	<0.005
IGKV3-20	2.711	5.002	17.836	<0.0001	<0.005
BHLHA15	3.65	2.455	17.821	<0.0001	<0.005
IGLV3-9	4.423	2.881	17.799	<0.0001	<0.005
ST8SIA1	-1.332	2.098	17.785	<0.0001	<0.005
IGKV1-33	2.542	3.344	17.705	<0.0001	<0.005
TIMELESS	1.149	3.28	17.694	<0.0001	<0.005
CCDC13-AS2	-1.452	2.244	17.664	<0.0001	<0.005
ENSG00000284292	-3.334	2.961	17.57	<0.0001	<0.005
IGLV3-19	2.552	3.399	17.535	<0.0001	<0.005
WDR90	-5.917	10.43	17.522	<0.0001	<0.005
IGKV1-27	4.603	4.794	17.52	<0.0001	<0.005
PLK4	1.693	1.725	17.421	<0.0001	<0.005
IGHV4-59	3.915	5.625	17.375	<0.0001	<0.005
CLSPN	1.882	1.828	17.356	<0.0001	<0.005
ENSG00000263731	-1.762	-0.048	17.343	<0.0001	<0.005
IGLV7-43	4.243	3.388	17.31	<0.0001	<0.005
SPAG5	1.884	2.576	17.251	<0.0001	<0.005
OLFM1	-2.539	1.384	17.25	<0.0001	<0.005
KCNQ5	1.446	1.354	17.238	<0.0001	<0.005
MZB1	3.115	5.262	17.197	<0.0001	<0.005
ASPM	2.106	2.337	17.183	<0.0001	<0.005
SPACA6	-1.763	0.914	17.005	<0.0001	<0.05
BUB1	2.422	2.829	16.922	<0.0001	<0.05
ENSG00000274383	-1.936	-0.401	16.823	<0.0001	<0.05
IGHJ5	3.888	8.246	16.784	<0.0001	<0.05

MARCKSL1	-0.833	5.742	16.765	<0.0001	<0.05
IGKV2D-29	3.409	1.027	16.742	<0.0001	<0.05
IGHV3-33	3.055	5.299	16.697	<0.0001	<0.05
IRF7	1.613	6.506	16.652	<0.0001	<0.05
EMP1	2.106	1.858	16.621	<0.0001	<0.05
IGHV1-18	2.527	3.553	16.464	<0.0001	<0.05
IGKV1D-39	3.272	4.923	16.458	<0.0001	<0.05
CCNE2	1.932	0.854	16.432	<0.0001	<0.05
DDX11L2	-1.579	2.779	16.393	<0.0001	<0.05
GPR162	-1.407	2.883	16.391	<0.0001	<0.05
IGHV3-66	3.181	2.018	16.376	<0.0001	<0.05
KLHL14	2.274	2.854	16.354	<0.0001	<0.05
IFI27	6.224	7.363	16.267	<0.0001	<0.05
CDC6	2.581	1.854	16.215	<0.0001	<0.05
IGKV1-12	3.04	3.428	16.047	<0.0001	<0.05
RRM2	2.363	4.253	15.868	<0.0001	<0.05
KNL1	1.674	1.945	15.792	<0.0001	<0.05
CHEK1	1.935	1.707	15.567	<0.0001	<0.05
IGLV1-36	3.626	1.777	15.447	<0.0001	<0.05
KIAA1958	1.474	2.402	15.427	<0.0001	<0.05
ORC6	1.95	0.217	15.351	<0.0001	<0.05
CAV1	2.647	1.041	15.32	<0.0001	<0.05
IGKJ1	2.468	7.929	15.289	<0.0001	<0.05
IGHV3-20	3.929	2.413	15.222	<0.0001	<0.05
CASP10	0.736	5.222	15.126	<0.0005	<0.05
GCAT	-2.02	1.19	15.103	<0.0005	<0.05
BRCA1	0.975	3.554	15.06	<0.0005	<0.05
ZWINT	1.933	2.285	15.052	<0.0005	<0.05
IGKV3D-20	2.959	3.067	15.009	<0.0005	<0.05
ENSG00000283761	3.054	2.851	14.975	<0.0005	<0.05

EXO1	2.143	0.662	14.871	<0.0005	<0.05
C5AR2	-0.895	5.721	14.832	<0.0005	<0.05
TRAM2	1.272	4.582	14.737	<0.0005	<0.05
IGLV7-46	3.693	4.378	14.734	<0.0005	<0.05
FANCI	1.081	3.496	14.714	<0.0005	<0.05
MS4A3	-1.325	3.38	14.703	<0.0005	<0.05
IGLC2	3.156	8.72	14.602	<0.0005	<0.05
IGLV1-44	3.524	6.432	14.533	<0.0005	<0.05
CCNB1	1.962	2.261	14.533	<0.0005	<0.05
CDH2	-2.155	1.177	14.477	<0.0005	<0.05
IGLV3-10	3.558	2.869	14.477	<0.0005	<0.05
ENSG00000273447	-1.282	-1.144	14.394	<0.0005	<0.05
ENSG00000260257	-1.644	1.096	14.357	<0.0005	<0.05
TNFRSF17	2.978	3.132	14.319	<0.0005	<0.05
GPRC5D	3.496	1.082	14.311	<0.0005	<0.05
JCHAIN	2.946	8.689	14.262	<0.0005	<0.05
EZH2	1.193	2.967	14.238	<0.0005	<0.05
RELL1	-1.091	4.624	14.219	<0.0005	<0.05
IGKC	2.714	9.896	14.213	<0.0005	<0.05
ZNF395	-2.776	2.021	14.173	<0.0005	<0.05
TTK	2.463	0.835	14.14	<0.0005	<0.05
C9orf78	-1.365	8.33	14.114	<0.0005	<0.05
CENPF	1.581	2.773	14.099	<0.0005	<0.05
TEKT4P2	-1.614	1.642	14.098	<0.0005	<0.05
IGHV1-46	2.605	2.722	14.061	<0.0005	<0.05
RECQL4	1.561	1.263	14.022	<0.0005	<0.05
CCDC86-AS1	-1.424	3.197	13.994	<0.0005	<0.05
MELK	2.471	0.864	13.993	<0.0005	<0.05
GGH	1.983	1.99	13.906	<0.0005	<0.05
IGHJ6	2.839	9.028	13.897	<0.0005	<0.05

BLZF1	0.78	4.314	13.875	<0.0005	<0.05
CHRAC1	-0.622	4.482	13.841	<0.0005	<0.05
SPINT2	-0.684	5.276	13.832	<0.0005	<0.05
KIF11	1.577	2.782	13.753	<0.0005	<0.05
DIRAS1	-1.489	-0.291	13.724	<0.0005	<0.05
ENSG00000290027	1.928	1.913	13.7	<0.0005	<0.05
FAM111B	1.843	2.064	13.677	<0.0005	<0.05
GTSE1	1.982	1.324	13.663	<0.0005	<0.05
SPATS2L	2.08	4.034	13.66	<0.0005	<0.05
UHRF1	1.501	2.371	13.635	<0.0005	<0.05
BUB1B	2.435	1.427	13.623	<0.0005	<0.05
HMMR	2.474	1.588	13.604	<0.0005	<0.05
KNDC1	-1.835	1.234	13.599	<0.0005	<0.05
DENND5B	1.909	2.898	13.594	<0.0005	<0.05
IGLC6	2.863	1.171	13.557	<0.0005	<0.05
ENSG00000285444	-1.089	3.968	13.55	<0.0005	<0.05
KNTC1	1.168	3.086	13.503	<0.0005	<0.05
TP53INP2	-1.055	4.149	13.478	<0.0005	<0.05
CCNI	-0.9	8.564	13.475	<0.0005	<0.05
GLDC	2.726	1.746	13.466	<0.0005	<0.05
MANEA	1.241	3.763	13.44	<0.0005	<0.05
LMAN1	1.198	5.67	13.435	<0.0005	<0.05
PRDX6	-1.032	7.417	13.433	<0.0005	<0.05
NUP205	0.748	5.141	13.421	<0.0005	<0.05
ENSG00000288924	-0.701	4.027	13.403	<0.0005	<0.05
ENSG00000255320	-0.808	3.321	13.388	<0.0005	<0.05
GGTA1	-1.227	3.989	13.377	<0.0005	<0.05
VPS37C	-0.717	4.421	13.371	<0.0005	<0.05
LINC02458	-1.502	0.488	13.359	<0.0005	<0.05
IGLC3	2.487	8.074	13.353	<0.0005	<0.05

SUV39H2	1.163	1.622	13.351	<0.0005	<0.05
PSAT1	1.429	2.216	13.331	<0.0005	<0.05
HERC6	1.349	4.874	13.326	<0.0005	<0.05
SMC4	0.848	5.285	13.3	<0.0005	<0.05
ELL2	1.558	4.907	13.286	<0.0005	<0.05
ENSG00000288684	4.385	2.941	13.282	<0.0005	<0.05
TCN2	1.433	3.176	13.28	<0.0005	<0.05
MTHFD2	0.87	4.904	13.268	<0.0005	<0.05
ENSG00000282988	1.398	3.503	13.258	<0.0005	<0.05
MYBL2	2.353	3.864	13.247	<0.0005	<0.05
TMOD1	-1.579	4.724	13.225	<0.0005	<0.05
BCAM	-3.074	0.933	13.207	<0.0005	<0.05
NCAPH	1.608	1.493	13.184	<0.0005	<0.05
FBXO7	-1.539	9.871	13.16	<0.0005	<0.05
TPX2	1.82	2.71	13.152	<0.0005	<0.05
IGKV3D-15	2.477	3.735	13.129	<0.0005	<0.05
NDC80	1.363	2.379	13.104	<0.0005	<0.05
DEPDC1B	2.3	0.514	13.091	<0.0005	<0.05
NUDT3	-0.586	6.654	13.079	<0.0005	<0.05
KIF4A	2.203	0.992	13.021	<0.0005	<0.05
SEC24A	0.937	4.512	12.965	<0.0005	<0.05
MIR3652	2.751	7.871	12.963	<0.0005	<0.05
IRF4	1.467	5.217	12.963	<0.0005	<0.05
IGHV4-34	2.629	3.624	12.94	<0.0005	<0.05
IGHV3-23	2.744	5.67	12.898	<0.0005	<0.05
AMOTL1	-2.497	2.188	12.864	<0.0005	<0.05
TMEM119	2.266	0.814	12.845	<0.0005	<0.05
IGLV2-14	2.639	5.91	12.824	<0.0005	<0.05
CCNA2	1.91	2.212	12.818	<0.0005	<0.05
DLGAP5	2.439	1.961	12.787	<0.0005	<0.05

KIF14	2.715	0.668	12.782	<0.0005	<0.05
BRCA2	1.385	2.066	12.753	<0.0005	<0.05
ENSG00000261338	-1.012	2.741	12.749	<0.0005	<0.05
IGHV3-74	2.767	3.393	12.747	<0.0005	<0.05
IGLV1-51	2.615	4.565	12.666	<0.0005	<0.05
IGHV3-7	2.346	4.541	12.615	<0.0005	<0.05
IGHV4-31	3.734	2.261	12.561	<0.0005	<0.05
SQLE	1.027	3.114	12.548	<0.0005	<0.05
BORA	1.112	2.065	12.539	<0.0005	<0.05
SPATA6	-0.947	2.057	12.532	<0.0005	<0.05
ENSG00000241666	-1.861	0.274	12.53	<0.0005	<0.05
EAF2	1.649	2.97	12.503	<0.0005	<0.05
IGLV2-11	2.239	4.387	12.502	<0.0005	<0.05
HLA-DQA1	-1.127	6.366	12.497	<0.0005	<0.05
CCNB2	2.09	1.721	12.474	<0.0005	<0.05
ENSG00000237400	-0.872	2.062	12.424	<0.0005	<0.05
CKAP5	0.602	5.473	12.402	<0.0005	<0.05
GPR146	-1.248	4.705	12.387	<0.0005	<0.05
ZFYVE26	0.701	4.969	12.377	<0.0005	<0.05
C8orf44-SGK3	-2.495	1.702	12.377	<0.0005	<0.05
RGS16	1.994	0.557	12.361	<0.0005	<0.05
ENSG00000291048	-0.964	4.537	12.351	<0.0005	<0.05
GMNN	1.278	2.135	12.351	<0.0005	<0.05
SMC2	0.881	3.933	12.339	<0.0005	<0.05
IGHV1-2	2.652	3.605	12.302	<0.0005	<0.05
MASTL	1.18	2.589	12.267	<0.0005	<0.05
ESPL1	2.285	0.758	12.235	<0.0005	<0.05
WDHD1	1.139	2.17	12.186	<0.0005	<0.05
LRRC61	-1.066	2.895	12.111	<0.0005	<0.05
CACNG6	-1.738	1.494	12.104	<0.0005	<0.05

MCM2	1.225	3.722	12.086	<0.005	<0.05
SGO2	1.134	2.122	12.084	<0.005	<0.05
KIF2C	2.039	1.505	12.08	<0.005	<0.05
RAD51	1.78	0.985	12.039	<0.005	<0.05
POLE2	1.736	0.023	12.028	<0.005	<0.05
RTN1	-1.097	4.166	11.943	<0.005	<0.05
TONSL	1.026	1.654	11.907	<0.005	<0.05
B9D1	2.379	-0.047	11.89	<0.005	<0.05
PDK1	0.945	4.434	11.886	<0.005	<0.05
IGLV8-61	3.468	3.44	11.881	<0.005	<0.05
IGHV1-24	2.911	1.407	11.841	<0.005	<0.05
IGLV2-18	2.947	2.208	11.827	<0.005	<0.05
AKT1S1	-0.716	4.295	11.795	<0.005	<0.05
DERL3	2.479	1.816	11.794	<0.005	<0.05
GBP1P1	2.063	2.218	11.755	<0.005	<0.05
IGKV3-15	2.161	5.117	11.741	<0.005	<0.05
UAP1	1.223	4.262	11.736	<0.005	<0.05
FKBP11	1.187	4.414	11.723	<0.005	<0.05
IGHV3-49	3.272	2.984	11.721	<0.005	<0.05
NOMO1	0.735	5.457	11.716	<0.005	<0.05
ENSG00000286129	2.09	4.683	11.682	<0.005	<0.05
FAM30A	1.595	4.104	11.679	<0.005	<0.05
DBP	-0.726	3.953	11.678	<0.005	<0.05
LAMC1	1.557	2.992	11.668	<0.005	<0.05
TOPBP1	0.63	5.376	11.625	<0.005	<0.05
USP18	2.452	3.32	11.621	<0.005	<0.05
UBR1	0.717	4.993	11.593	<0.005	<0.05
PNRC2P1	1.016	0.338	11.586	<0.005	<0.05
TCF19	0.959	3.323	11.577	<0.005	<0.05
PGAM1P8	-1.441	2.205	11.568	<0.005	<0.05

INTS7	0.674	3.457	11.558	<0.005	<0.05
MT-ND4	-1.349	9.784	11.551	<0.005	<0.05
JUP	1.455	4.797	11.531	<0.005	<0.05
TRGV2	1.418	1.678	11.511	<0.005	<0.05
SDC2	-1.612	1.357	11.445	<0.005	<0.05
ECT2	1.05	2.463	11.424	<0.005	<0.05
IGHG1	3.193	6.795	11.404	<0.005	<0.05
FBXO5	0.962	2.567	11.403	<0.005	<0.05
JADE1	-0.514	5.592	11.384	<0.005	<0.05
TRAPPC6A	-0.737	3.381	11.352	<0.005	<0.05
RNASET2	-0.89	8.008	11.347	<0.005	<0.05
DDX11L17	-1.775	0.837	11.325	<0.005	<0.05
CEP55	1.847	1.696	11.302	<0.005	<0.05
RABEP1	0.643	5.43	11.292	<0.005	<0.05
FCER1A	-1.141	5.151	11.274	<0.005	<0.05
SAMD9L	1.334	8.03	11.273	<0.005	<0.05
GATA2	-0.953	4.301	11.266	<0.005	<0.05
ATAD5	1.26	1.554	11.252	<0.005	<0.05
NAAA	-0.843	5.712	11.239	<0.005	<0.05
MPZL3	-0.848	6.304	11.233	<0.005	<0.05
RAD51AP1	1.596	0.529	11.232	<0.005	<0.05
SKA1	2.146	0.613	11.161	<0.005	<0.05
SEL1L3	1.095	6.241	11.161	<0.005	<0.05
UBA52	-1.004	10.83	11.157	<0.005	<0.05
CDT1	1.812	1.633	11.097	<0.005	<0.05
DHCR24	1.182	3.265	11.092	<0.005	<0.05
MXD4	-0.619	5.865	11.08	<0.005	<0.05
XPO1	0.577	6.52	11.062	<0.005	<0.05
CDC25A	2.596	0.972	11.043	<0.005	<0.05
HSPA1L	-0.696	2.998	11.037	<0.005	<0.05

PDIA4	1.172	6.301	11.022	<0.005	<0.05
OASL	1.501	6.57	11.013	<0.005	<0.05
KIF23	1.393	1.502	11.006	<0.005	<0.05
KIF18A	1.578	0.415	11.006	<0.005	<0.05
CENPO	1.588	2.112	11	<0.005	<0.05
LTA4H	-0.713	7.784	10.983	<0.005	<0.05
PGM5	-1.912	2.49	10.963	<0.005	<0.05
NET1	1.307	3.346	10.95	<0.005	<0.05
NUF2	1.687	1.135	10.94	<0.005	<0.05
RNF182	-2.865	1.653	10.93	<0.005	<0.05
RTEL1	1.052	2.893	10.891	<0.005	<0.05
KIFC1	1.903	1.938	10.874	<0.005	<0.05
IGHGP	2.136	3.291	10.863	<0.005	<0.05
EIF4G3	0.64	5.841	10.858	<0.005	<0.05
FOXM1	1.722	1.893	10.838	<0.005	<0.05
ENSG00000289273	-3.326	4.625	10.799	<0.005	<0.05
OXCT1	0.689	4.238	10.787	<0.005	<0.05
SLAMF7	0.961	6.372	10.786	<0.005	<0.05
CEP295	1.103	3.688	10.775	<0.005	<0.05
ANKDD1A	-1.092	3.846	10.773	<0.005	<0.05
RIC1	0.664	5.434	10.758	<0.005	<0.05
FLJ40194	-1.067	-0.451	10.732	<0.005	<0.05
SKIC3	0.617	5.249	10.725	<0.005	<0.05
ENSG00000285417	1.152	2.931	10.712	<0.005	<0.05
RPL22L1	0.902	3.53	10.705	<0.005	<0.05
LDLR	0.951	4.071	10.703	<0.005	<0.05
TMEM106C	0.793	3.83	10.684	<0.005	<0.05
IGHV3-69-1	2.741	1.402	10.675	<0.005	<0.05
SEC11C	1.534	5.545	10.647	<0.005	<0.05
BMAL2	1.702	0.775	10.627	<0.005	<0.05

COL4A4	1.977	0.911	10.623	<0.005	<0.05
SPG21	-0.638	6.286	10.617	<0.005	<0.05
LIN9	1.059	1.357	10.608	<0.005	<0.05
AURKA	1.425	1.816	10.598	<0.005	<0.05
ZBTB32	1.759	1.238	10.592	<0.005	<0.05
HLA-DOA	-0.875	4.917	10.586	<0.005	<0.05

Table S5: Differentially expressed genes found comparing acute individuals and chronic group.

Supplementary Table S5: Differentially expressed genes found comparing acute individuals and chronic group.

genes	logFC	logCPM	LR	PValue	FDR
DTL	3.149	1.904	100.452	<0.0001	<0.0001
RRM2	3.568	4.253	95.368	<0.0001	<0.0001
TOP2A	2.551	3.464	87.874	<0.0001	<0.0001
SHCBP1	3.204	2.303	86.667	<0.0001	<0.0001
IGLV3-25	5.04	5.111	84.921	<0.0001	<0.0001
IGHV2-5	4.252	4.39	84.878	<0.0001	<0.0001
MCM4	1.958	3.879	83.445	<0.0001	<0.0001
ASPM	2.846	2.337	83.245	<0.0001	<0.0001
MZB1	4.044	5.262	81.513	<0.0001	<0.0001
CD38	2.265	4.817	80.389	<0.0001	<0.0001
BUB1	3.176	2.829	79.107	<0.0001	<0.0001
IGHV5-51	5.246	5.993	78.594	<0.0001	<0.0001
MKI67	2.506	4.759	77.446	<0.0001	<0.0001
TXNDC5	3.3	7.874	75.731	<0.0001	<0.0001
EXO1	2.97	0.662	74.665	<0.0001	<0.0001
IGLC3	3.589	8.074	74.613	<0.0001	<0.0001
KNL1	2.24	1.945	72.837	<0.0001	<0.0001
IGHV4-61	3.832	4.125	72.616	<0.0001	<0.0001
OTOF	4.343	2.601	71.913	<0.0001	<0.0001
IGHV3-21	3.375	4.407	71.773	<0.0001	<0.0001
CDC6	3.269	1.854	71.303	<0.0001	<0.0001
IGKV1-12	3.803	3.428	71.11	<0.0001	<0.0001
CCNB2	3.088	1.721	70.794	<0.0001	<0.0001
TPX2	2.619	2.71	70.543	<0.0001	<0.0001
NCAPG	2.622	2.282	70.437	<0.0001	<0.0001
IGLV3-1	4.585	7.473	70.144	<0.0001	<0.0001
IGLV2-23	3.487	5.2	69.183	<0.0001	<0.0001

IGKV4-1	3.742	6.406	68.849	<0.0001	<0.0001
GLDC	3.722	1.746	67.993	<0.0001	<0.0001
PCLAF	3.043	1.717	67.941	<0.0001	<0.0001
MYBL2	3.232	3.864	67.32	<0.0001	<0.0001
ORC1	2.495	1.221	67.315	<0.0001	<0.0001
IGHG3	2.554	5.836	67.293	<0.0001	<0.0001
BHLHA15	4.1	2.455	67.158	<0.0001	<0.0001
SPAG5	2.278	2.576	67.102	<0.0001	<0.0001
IGHV4-59	4.424	5.625	67.097	<0.0001	<0.0001
IGHV3-48	3.676	4.257	66.182	<0.0001	<0.0001
IGLV7-46	4.598	4.378	66.176	<0.0001	<0.0001
IGHV1-69	4.799	4.389	65.72	<0.0001	<0.0001
CDT1	2.759	1.633	65.569	<0.0001	<0.0001
TNFRSF17	3.792	3.132	65.166	<0.0001	<0.0001
IGHM	4.31	10.158	64.799	<0.0001	<0.0001
IGHV3-33	3.524	5.299	64.427	<0.0001	<0.0001
DLGAP5	3.336	1.961	64.247	<0.0001	<0.0001
CCNA2	2.636	2.212	63.911	<0.0001	<0.0001
CEP55	2.741	1.696	63.902	<0.0001	<0.0001
IGKV2D-28	4.393	5.041	63.733	<0.0001	<0.0001
IGKJ1	3.007	7.929	63.119	<0.0001	<0.0001
JCHAIN	3.681	8.689	63.024	<0.0001	<0.0001
IGKV3-15	3.067	5.117	62.901	<0.0001	<0.0001
GTSE1	2.626	1.324	62.871	<0.0001	<0.0001
IGHV1-18	2.937	3.553	62.437	<0.0001	<0.0001
IGHV1-69D	4.458	4.698	62.388	<0.0001	<0.0001
IGKV3D-15	3.265	3.735	62.364	<0.0001	<0.0001
IGHV3-30	3.68	5.633	62.153	<0.0001	<0.0001
IGKV1-27	4.865	4.794	62.087	<0.0001	<0.0001
IGHV4-39	3.246	4.49	61.456	<0.0001	<0.0001

DERL3	3.43	1.816	60.587	<0.0001	<0.0001
PLK1	2.676	1.826	60.482	<0.0001	<0.0001
IGHJ6	3.516	9.028	60.195	<0.0001	<0.0001
BIRC5	3.257	2.044	60.068	<0.0001	<0.0001
IGLV3-10	4.233	2.869	60.01	<0.0001	<0.0001
CCNB1	2.443	2.261	59.976	<0.0001	<0.0001
IGHJ5	4.192	8.246	59.905	<0.0001	<0.0001
IGLV3-19	2.778	3.399	59.044	<0.0001	<0.0001
ZWINT	2.342	2.285	58.897	<0.0001	<0.0001
IGKV1D-33	2.735	3.14	58.874	<0.0001	<0.0001
IGKV1D-39	3.59	4.923	58.819	<0.0001	<0.0001
IGLV3-9	4.5	2.881	58.81	<0.0001	<0.0001
SPATS2L	2.623	4.034	58.572	<0.0001	<0.0001
HMMR	3.082	1.588	57.946	<0.0001	<0.0001
KIFC1	2.722	1.938	57.847	<0.0001	<0.0001
IGKV1-33	2.722	3.344	57.772	<0.0001	<0.0001
KIF2C	2.744	1.505	57.64	<0.0001	<0.0001
UHRF1	1.925	2.371	57.396	<0.0001	<0.0001
FOXM1	2.469	1.893	57.38	<0.0001	<0.0001
IGKC	3.214	9.896	56.497	<0.0001	<0.0001
IGLC2	3.604	8.72	55.678	<0.0001	<0.0001
KIF4A	2.781	0.992	55.483	<0.0001	<0.0001
TCN2	1.828	3.176	55.063	<0.0001	<0.0001
FAM111B	2.266	2.064	54.604	<0.0001	<0.0001
IGLV1-51	3.243	4.565	54.363	<0.0001	<0.0001
CDCA5	2.771	1.667	54.045	<0.0001	<0.0001
CDK1	2.699	1.533	53.849	<0.0001	<0.0001
IGLV6-57	4.601	4.675	53.846	<0.0001	<0.0001
IGLC6	3.358	1.171	52.891	<0.0001	<0.0001
IGLV7-43	4.135	3.388	52.874	<0.0001	<0.0001

IGHV4-4	3.7	2.748	52.122	<0.0001	<0.0001
KIF11	1.906	2.782	52.056	<0.0001	<0.0001
CENPM	2.561	0.624	51.972	<0.0001	<0.0001
IGKV1-17	2.847	3.067	51.78	<0.0001	<0.0001
EPHB2	2.023	2.483	51.751	<0.0001	<0.0001
IGLV1-44	3.818	6.432	51.483	<0.0001	<0.0001
GINS2	2.59	1.324	51.263	<0.0001	<0.0001
CDC20	3.315	1.93	51.189	<0.0001	<0.0001
ITM2C	2.152	5.953	51.076	<0.0001	<0.0001
CLSPN	1.97	1.828	50.953	<0.0001	<0.0001
RTP4	1.957	4.169	50.925	<0.0001	<0.0001
IGKV3-20	2.671	5.002	50.619	<0.0001	<0.0001
CHEK1	2.123	1.707	50.432	<0.0001	<0.0001
CDC25A	3.357	0.972	50.42	<0.0001	<0.0001
DEPDC1B	2.738	0.514	50.283	<0.0001	<0.0001
IGHV4-34	3.084	3.624	50.271	<0.0001	<0.0001
NT5DC2	2.174	2.416	50.156	<0.0001	<0.0001
RAD51	2.254	0.985	50.056	<0.0001	<0.0001
KLHL14	2.367	2.854	49.573	<0.0001	<0.0001
CENPF	1.825	2.773	48.921	<0.0001	<0.0001
IGHV3-7	2.77	4.541	48.737	<0.0001	<0.0001
IGKV1-16	2.956	3.04	48.657	<0.0001	<0.0001
IGHV3-43	3.905	2.236	48.584	<0.0001	<0.0001
ESCO2	2.605	0.46	48.397	<0.0001	<0.0001
IGKV1D-12	3.324	1.797	48.168	<0.0001	<0.0001
HJURP	2.868	1.306	48.125	<0.0001	<0.0001
PCNA	1.118	4.871	48.081	<0.0001	<0.0001
IGLL5	3.454	6.257	47.985	<0.0001	<0.0001
IRF7	1.672	6.506	47.119	<0.0001	<0.0001
IGHV1-46	2.812	2.722	46.764	<0.0001	<0.0001

IGLV1-36	3.579	1.777	46.63	<0.0001	<0.0001
IGHV3-11	3.579	4.056	46.618	<0.0001	<0.0001
TTK	2.666	0.835	46.316	<0.0001	<0.0001
FABP5	1.753	2.818	46.286	<0.0001	<0.0001
GMNN	1.563	2.135	46.267	<0.0001	<0.0001
STIL	1.794	1.239	46.031	<0.0001	<0.0001
IGHV3-66	3.062	2.018	45.951	<0.0001	<0.0001
IGHJ4	2.839	10.026	45.922	<0.0001	<0.0001
IGKV3D-11	2.765	1.322	45.474	<0.0001	<0.0001
IGKV3D-20	2.99	3.067	45.39	<0.0001	<0.0001
KIF23	1.78	1.502	45.371	<0.0001	<0.0001
MELK	2.647	0.864	44.981	<0.0001	<0.0001
BUB1B	2.639	1.427	44.812	<0.0001	<0.0001
EAF2	1.927	2.97	44.714	<0.0001	<0.0001
FKBP11	1.464	4.414	44.55	<0.0001	<0.0001
NCAPH	1.833	1.493	44.464	<0.0001	<0.0001
CDKN3	2.415	1.106	44.355	<0.0001	<0.0001
POLE2	2.075	0.023	44.152	<0.0001	<0.0001
IGLV8-61	3.846	3.44	43.624	<0.0001	<0.0001
FEN1	1.441	3.694	43.597	<0.0001	<0.0001
TYMS	2.943	2.175	43.596	<0.0001	<0.0001
UBE2C	1.961	1.618	43.548	<0.0001	<0.0001
SEC11C	1.928	5.545	43.512	<0.0001	<0.0001
KIF15	2.657	1.231	43.425	<0.0001	<0.0001
ESPL1	2.598	0.758	43.389	<0.0001	<0.0001
KPNA2	1.193	4.285	43.357	<0.0001	<0.0001
UBE2T	1.937	1.103	43.301	<0.0001	<0.0001
IGLV2-18	3.312	2.208	43.199	<0.0001	<0.0001
MCM2	1.463	3.722	43.19	<0.0001	<0.0001
SIGLEC1	3.16	5.293	42.663	<0.0001	<0.0001

OASL	1.833	6.57	42.45	<0.0001	<0.0001
MTHFD2	1.002	4.904	42.429	<0.0001	<0.0001
IGHV3-23	2.912	5.67	42.085	<0.0001	<0.0001
IGKV1-9	2.808	3.424	42.056	<0.0001	<0.0001
SKA1	2.536	0.613	41.969	<0.0001	<0.0001
GPRC5D	3.41	1.082	41.791	<0.0001	<0.0001
STMN1	1.442	4.925	41.466	<0.0001	<0.0001
FANCI	1.154	3.496	41.449	<0.0001	<0.0001
IGHV3-69-1	3.207	1.402	41.302	<0.0001	<0.0001
OAS1	2.191	7.494	41.267	<0.0001	<0.0001
IGLV2-11	2.43	4.387	40.908	<0.0001	<0.0001
MMACHC	2.183	2.926	40.805	<0.0001	<0.0001
MCM6	1.07	4.674	40.786	<0.0001	<0.0001
AURKA	1.751	1.816	40.636	<0.0001	<0.0001
GGH	2.055	1.99	40.554	<0.0001	<0.0001
PLAC8	1.129	6.363	40.441	<0.0001	<0.0001
CAV1	2.52	1.041	40.16	<0.0001	<0.0001
CENPN	1.356	2.273	39.958	<0.0001	<0.0001
SLC1A4	1.334	3.968	39.587	<0.0001	<0.0001
IGHV3-20	3.513	2.413	39.452	<0.0001	<0.0001
CHPF	1.747	2.807	39.346	<0.0001	<0.0001
PKMYT1	2.441	0.676	39.243	<0.0001	<0.0001
RACGAP1	1.092	3.106	39.189	<0.0001	<0.0001
IGLV5-45	2.762	0.992	39.165	<0.0001	<0.0001
TXNDC11	1.248	5.462	39.117	<0.0001	<0.0001
MIR3652	2.793	7.871	39.018	<0.0001	<0.0001
CENPE	1.622	1.975	38.823	<0.0001	<0.0001
TMOD1	-2.128	4.724	38.787	<0.0001	<0.0001
IGHV6-1	3.239	2.626	38.705	<0.0001	<0.0001
PLK4	1.55	1.725	38.688	<0.0001	<0.0001

AURKB	2.475	1.075	38.667	<0.0001	<0.0001
NUF2	1.961	1.135	38.555	<0.0001	<0.0001
TESC	-1.718	6.567	38.514	<0.0001	<0.0001
IGLV4-69	2.934	2.441	38.475	<0.0001	<0.0001
IGHV3-49	3.421	2.984	38.462	<0.0001	<0.0001
NME4	-4.437	9.271	38.389	<0.0001	<0.0001
LY6E	2.159	8.114	38.335	<0.0001	<0.0001
IGLC7	2.666	2.727	38.229	<0.0001	<0.0001
SPATS2	1.588	2.418	38.156	<0.0001	<0.0001
CHAF1B	1.365	1.19	38.11	<0.0001	<0.0001
USP18	2.638	3.32	38.058	<0.0001	<0.0001
CENPU	1.579	2.083	37.885	<0.0001	<0.0001
IGKJ5	2.146	5.95	37.196	<0.0001	<0.0001
GINS1	2.285	0.617	37.015	<0.0001	<0.0001
JUP	1.621	4.797	36.945	<0.0001	<0.0001
APOBEC3G	0.748	6.258	36.941	<0.0001	<0.0001
LNCRNA-IUR	-1.6	3.629	36.939	<0.0001	<0.0001
TIMELESS	1.051	3.28	36.888	<0.0001	<0.0001
POU2AF1	1.83	4.776	36.867	<0.0001	<0.0001
NME1	1.657	3.427	36.855	<0.0001	<0.0001
TP53INP2	-1.301	4.149	36.823	<0.0001	<0.0001
ISG15	2.527	6.822	36.756	<0.0001	<0.0001
MIR23AHG	-1.261	4.117	36.741	<0.0001	<0.0001
RNASE1	2.681	1.178	36.637	<0.0001	<0.0001
TLR7	1.08	4.802	36.597	<0.0001	<0.0001
IGHA1	2.661	7.02	36.552	<0.0001	<0.0001
FBXO5	1.104	2.567	36.437	<0.0001	<0.0001
PRDX4	1.339	3.474	36.329	<0.0001	<0.0001
HERC6	1.394	4.874	36.325	<0.0001	<0.0001
PDIA4	1.341	6.301	36.097	<0.0001	<0.0001

FAM234A	-4.612	10.147	35.979	<0.0001	<0.0001
IGLV9-49	3.915	1.576	35.889	<0.0001	<0.0001
IGHV1-2	2.652	3.605	35.711	<0.0001	<0.0001
AK1	-2.398	3.439	35.596	<0.0001	<0.0001
RAD51AP1	1.764	0.529	35.452	<0.0001	<0.0001
AXIN1	-4.524	11.789	35.421	<0.0001	<0.0001
METTL26	-4.139	8.704	35.413	<0.0001	<0.0001
IGHV4-31	3.527	2.261	35.381	<0.0001	<0.0001
PSMA8	1.123	2.103	35.344	<0.0001	<0.0001
RELL1	-1.284	4.624	35.281	<0.0001	<0.0001
RHOT2	-4.125	10.243	35.248	<0.0001	<0.0001
TCF19	1.071	3.323	35.141	<0.0001	<0.0001
TK1	2.453	2.285	35.136	<0.0001	<0.0001
CHAC2	1.753	1.38	35.13	<0.0001	<0.0001
FABP5P7	1.913	-0.294	34.957	<0.0001	<0.0001
SDF2L1	1.484	3.614	34.894	<0.0001	<0.0001
NDC80	1.393	2.379	34.889	<0.0001	<0.0001
SGIP1	-3.035	-0.028	34.628	<0.0001	<0.0001
UAP1	1.324	4.262	34.614	<0.0001	<0.0001
PSMC2	0.789	5.366	34.518	<0.0001	<0.0001
CDCA7	1.457	1.481	34.515	<0.0001	<0.0001
SNORD17	2.413	2.941	34.513	<0.0001	<0.0001
LMAN1	1.211	5.67	34.504	<0.0001	<0.0001
SLAMF7	1.1	6.372	34.461	<0.0001	<0.0001
IGHV3-53	2.873	2.715	34.428	<0.0001	<0.0001
PGAP6	-3.392	10.436	34.29	<0.0001	<0.0001
DYNC2I2	1.33	2.036	34.147	<0.0001	<0.0001
BBOF1	-1.732	4.06	34.047	<0.0001	<0.0001
WDR24	-4.53	9.597	33.993	<0.0001	<0.0001
EPSTI1	1.939	6.601	33.967	<0.0001	<0.0001

STUB1	-3.546	8.42	33.958	<0.0001	<0.0001
JMJD8	-3.702	8.822	33.848	<0.0001	<0.0001
IGHG1	3.159	6.795	33.782	<0.0001	<0.0001
ATAD2	0.86	4.081	33.774	<0.0001	<0.0001
OAS2	1.636	7.904	33.754	<0.0001	<0.0001
IGKV1-39	3.172	4.521	33.727	<0.0001	<0.0001
FBXL16	-5.014	10.14	33.327	<0.0001	<0.0001
CKS2	1.359	2.428	33.17	<0.0001	<0.0001
DHCR24	1.292	3.265	33.144	<0.0001	<0.0001
CENPW	1.813	0.836	33.094	<0.0001	<0.0001
RPL22L1	1.019	3.53	33.044	<0.0001	<0.0001
PSME2P1	1.379	0.462	32.987	<0.0001	<0.0001
GCH1	0.726	5.307	32.925	<0.0001	<0.0001
CAPN15	-4.107	10.466	32.881	<0.0001	<0.0001
PYCR1	3.091	0.644	32.821	<0.0001	<0.0001
TRIM69	0.85	6.35	32.818	<0.0001	<0.0001
KIF14	2.541	0.668	32.762	<0.0001	<0.0001
RAB11FIP3	-4.744	10.549	32.455	<0.0001	<0.0001
SCD	1.12	2.722	32.436	<0.0001	<0.0001
CNP	0.793	5.479	32.309	<0.0001	<0.0001
DNAJB11	1.097	5.435	32.259	<0.0001	<0.0001
C9orf78	-1.572	8.33	32.148	<0.0001	<0.0001
GINS4	1.453	1.087	32.08	<0.0001	<0.0001
RPS6KA5	-0.951	4.941	32.079	<0.0001	<0.0001
PTTG1	1.69	2.81	32.076	<0.0001	<0.0001
KIF20A	3.143	0.565	32.023	<0.0001	<0.0001
MYDGF	1.323	4.757	32.003	<0.0001	<0.0001
ANLN	2.024	0.838	32.001	<0.0001	<0.0001
IGHJ2P	2.323	6.685	31.987	<0.0001	<0.0001
MRPL28	-4.515	8.485	31.904	<0.0001	<0.0001

IGHV3-74	2.543	3.393	31.875	<0.0001	<0.0001
TROAP	2.647	0.388	31.871	<0.0001	<0.0001
EZH2	1.124	2.967	31.679	<0.0001	<0.0001
ZWILCH	0.993	2.513	31.659	<0.0001	<0.0001
HELLS	1.382	1.714	31.566	<0.0001	<0.0001
ATP5MC3	0.813	5.499	31.45	<0.0001	<0.0001
SPC24	2.224	0.084	31.383	<0.0001	<0.0001
ANTKMT	-4.581	8.826	31.264	<0.0001	<0.0001
ARHGAP42	1.743	0.992	31.086	<0.0001	<0.0001
HBQ1	-3.191	7.718	31.084	<0.0001	<0.0001
OR2W3	-2.233	5.388	31.05	<0.0001	<0.0001
ELL2	1.467	4.907	30.909	<0.0001	<0.0001
XBP1	1.247	7.328	30.897	<0.0001	<0.0001
PDIA5	1.428	2.415	30.735	<0.0001	<0.0001
FAM72B	1.554	-0.035	30.611	<0.0001	<0.0001
ASCC2	-1.454	7.654	30.521	<0.0001	<0.0001
CCNE2	1.588	0.854	30.481	<0.0001	<0.0001
NCAPG2	1.048	3.071	30.354	<0.0001	<0.0001
TPRG1-AS1	1.714	0.529	30.263	<0.0001	<0.0001
CIAO3	-4.835	10.718	30.234	<0.0001	<0.0001
C5AR2	-0.933	5.721	30.137	<0.0001	<0.0001
PPIB	1.257	7.257	29.981	<0.0001	<0.0001
GPR146	-1.468	4.705	29.954	<0.0001	<0.0001
MIR3176	-4.835	10.88	29.93	<0.0001	<0.0001
MCRIP2	-4.925	10.501	29.881	<0.0001	<0.0001
TRAM2	1.137	4.582	29.851	<0.0001	<0.0001
RECQL4	1.407	1.263	29.796	<0.0001	<0.0001
SGO2	1.13	2.122	29.776	<0.0001	<0.0001
PIGQ	-4.583	10.843	29.502	<0.0001	<0.0001
WDHD1	1.125	2.17	29.494	<0.0001	<0.0001

MT2A	1.389	4.515	29.363	<0.0001	<0.0001
CKAP2L	2.582	0.559	29.312	<0.0001	<0.0001
ENSG00000239920	-9.575	11.078	29.295	<0.0001	<0.0001
LUC7L	-4.903	11.986	29.278	<0.0001	<0.0001
INTS7	0.698	3.457	29.069	<0.0001	<0.0001
RAB40C	-4.857	11.991	29.026	<0.0001	<0.0001
SMC2	0.87	3.933	29.006	<0.0001	<0.0001
MCM7	0.9	5.36	28.949	<0.0001	<0.0001
POC1A	1.452	0.896	28.898	<0.0001	<0.0001
ZBTB32	1.779	1.238	28.774	<0.0001	<0.0001
RRM1	0.891	4.67	28.594	<0.0001	<0.0001
TARS1	0.683	5.432	28.482	<0.0001	<0.0001
CALU	0.814	5.012	28.389	<0.0001	<0.0001
TMEM258	0.932	4.729	28.387	<0.0001	<0.0001
HMGB3	1.672	1.64	28.366	<0.0001	<0.0001
SDC2	-1.968	1.357	28.22	<0.0001	<0.0001
LGALS3BP	1.434	5.031	28.215	<0.0001	<0.0001
GBP1P1	1.921	2.218	28.163	<0.0001	<0.0001
ORC6	1.593	0.217	28.112	<0.0001	<0.0001
HAGH	-1.385	6.316	28.092	<0.0001	<0.0001
TMEM106C	0.832	3.83	28.048	<0.0001	<0.0001
KIF18A	1.559	0.415	27.912	<0.0001	<0.0001
BRCA2	1.279	2.066	27.881	<0.0001	<0.0001
BCCIP	0.709	4.496	27.875	<0.0001	<0.0001
PRC1	2.263	0.55	27.85	<0.0001	<0.0001
MRPL13	0.809	3.412	27.841	<0.0001	<0.0001
TIMM17A	0.807	4.254	27.813	<0.0001	<0.0001
ADA	0.75	4.317	27.798	<0.0001	<0.0001
BLZF1	0.716	4.314	27.795	<0.0001	<0.0001
IGLC1	3.649	7.757	27.584	<0.0001	<0.0001

METRN	-4.952	9.713	27.576	<0.0001	<0.0001
ENSG00000286129	1.924	4.683	27.539	<0.0001	<0.0001
MANEA	1.117	3.763	27.514	<0.0001	<0.0001
OSBP2	-2.038	5.808	27.512	<0.0001	<0.0001
B4GALT2	1.478	1.434	27.497	<0.0001	<0.0001
DUSP5	1.172	4.135	27.492	<0.0001	<0.0001
IRF4	1.324	5.217	27.458	<0.0001	<0.0001
BAG1	-1.555	8.208	27.396	<0.0001	<0.0001
GFUS	-1.297	5.931	27.387	<0.0001	<0.0001
CASP10	0.643	5.222	27.311	<0.0001	<0.0001
IGHV1-24	2.543	1.407	27.21	<0.0001	<0.0001
TNFRSF13B	1.709	2.353	27.163	<0.0001	<0.0001
SAMD9L	1.294	8.03	27.099	<0.0001	<0.0001
IGLV2-14	2.23	5.91	27.075	<0.0001	<0.0001
DCLRE1A	1.042	3.286	27.006	<0.0001	<0.0001
NDUFA7	0.866	3.695	26.984	<0.0001	<0.0001
CDCA8	1.373	1.575	26.919	<0.0001	<0.0001
GBP3	1.208	5.065	26.791	<0.0001	<0.0001
IFI27L1	1.518	1.226	26.772	<0.0001	<0.0001
IFI6	1.914	7.308	26.704	<0.0001	<0.0001
PHGDH	1.407	1.641	26.627	<0.0001	<0.0001
SCARB2	0.678	5.543	26.571	<0.0001	<0.0001
PLEK2	-1.817	2.914	26.543	<0.0001	<0.0001
BOLA2B	0.97	3.557	26.498	<0.0001	<0.0001
TIGD3	-1.152	3.306	26.483	<0.0001	<0.0001
DECR2	-4.789	9.316	26.472	<0.0001	<0.0001
LAP3	1.286	6.354	26.463	<0.0001	<0.0001
PSAT1	1.254	2.216	26.458	<0.0001	<0.0001
IGKV1-8	1.817	1.008	26.454	<0.0001	<0.0001
DENND5B	1.607	2.898	26.382	<0.0001	<0.0001

UBE2S	0.861	3.929	26.331	<0.0001	<0.0001
BMAL2	1.641	0.775	26.229	<0.0001	<0.0001
ERLEC1	0.853	4.971	26.166	<0.0001	<0.0001
BTG3	0.904	1.93	26.149	<0.0001	<0.0001
BRIP1	1.126	1.011	26.101	<0.0001	<0.0001
ANXA4	0.606	4.829	26.096	<0.0001	<0.0001
LILRB4	1.075	4.993	26.079	<0.0001	<0.0001
VPS37C	-0.722	4.421	26.067	<0.0001	<0.0001
CCNF	1.294	2.029	26.057	<0.0001	<0.0001
C1QC	2.812	1.267	25.993	<0.0001	<0.0001
MANF	1.158	4.994	25.942	<0.0001	<0.0001
FAM83D	1.436	-0.024	25.941	<0.0001	<0.0001
CEP128	1.227	2.52	25.864	<0.0001	<0.0001
MLH1	0.619	4.05	25.838	<0.0001	<0.0001
HAGHL	-4.729	9.128	25.759	<0.0001	<0.0001
HYPK	0.831	3.953	25.746	<0.0001	<0.0001
MRPL27	0.799	3.317	25.707	<0.0001	<0.0001
PSMA5	0.654	5.761	25.703	<0.0001	<0.0001
CENPI	1.653	-0.063	25.622	<0.0001	<0.0001
PCK2	0.684	3.942	25.586	<0.0001	<0.0001
CA1	-3.273	6.728	25.584	<0.0001	<0.0001
MBNL3	-1.189	6.901	25.583	<0.0001	<0.0001
IFIH1	1.129	6.008	25.548	<0.0001	<0.0001
TSPAN5	-1.294	5.194	25.479	<0.0001	<0.0001
NUGGC	1.208	2.387	25.46	<0.0001	<0.0001
MAD2L1	1.182	2.127	25.411	<0.0001	<0.0001
H2AX	0.958	3.926	25.406	<0.0001	<0.0001
BOLA2	1.257	2.498	25.312	<0.0001	<0.0001
ABCB9	1.854	1.285	25.3	<0.0001	<0.0001
CIP2A	1.48	1.556	25.281	<0.0001	<0.0001

ENSG00000261659	-4.988	9.578	25.278	<0.0001	<0.0001
GATA1	-1.045	4.449	25.183	<0.0001	<0.0001
DDX60	1.341	6.173	25.122	<0.0001	<0.0001
ENSG00000289514	-1.695	2.988	25.112	<0.0001	<0.0001
IGKV2-24	2.532	2.995	25.049	<0.0001	<0.0001
NDUFA9	0.723	4.762	25.039	<0.0001	<0.0001
ALG14	1.465	1.259	25.03	<0.0001	<0.0001
C1QB	1.869	2.521	24.913	<0.0001	<0.0001
NEXN	1.323	3.743	24.896	<0.0001	<0.0001
BST2	0.845	6.326	24.852	<0.0001	<0.0001
SPCS2P4	1.289	1.681	24.63	<0.0001	<0.0001
TRIM58	-1.948	7.66	24.598	<0.0001	<0.0001
PPIAP31	1.108	-0.173	24.578	<0.0001	<0.0001
RBBP8	0.863	3.639	24.541	<0.0001	<0.0001
C2	1.769	2.722	24.524	<0.0001	<0.0001
IGF2BP2	-1.738	5.092	24.439	<0.0001	<0.0001
CIT	1.259	0.823	24.367	<0.0001	<0.0001
Y_RNA	-3.008	5.589	24.343	<0.0001	<0.0001
PTPRO	0.948	3.413	24.312	<0.0001	<0.0001
STRADB	-1.78	7.243	24.246	<0.0001	<0.0001
GPT2	1.375	1.017	24.226	<0.0001	<0.0001
TMEM126B	0.575	3.939	24.122	<0.0001	<0.0001
DNAJB2	-0.741	6.016	24.119	<0.0001	<0.0001
CCNE1	1.68	0.837	24.054	<0.0001	<0.0001
IGHGP	1.899	3.291	24.034	<0.0001	<0.0001
PLSCR1	1.251	6.223	24.019	<0.0001	<0.0001
LGALS1	0.985	6.745	23.994	<0.0001	<0.0001
AGO2	-0.751	6.396	23.977	<0.0001	<0.0001
UBB	-1.487	10.791	23.955	<0.0001	<0.0001
NUSAP1	1.84	2.506	23.928	<0.0001	<0.0001

IGHV1-3	2.414	2.344	23.86	<0.0001	<0.0001
SQLE	0.903	3.114	23.815	<0.0001	<0.0001
FBXO7	-1.588	9.871	23.809	<0.0001	<0.0001
IGKV2D-30	2.194	2.5	23.784	<0.0001	<0.0001
FAM72D	1.45	-0.362	23.78	<0.0001	<0.0001
IGF1R	-1.12	6.038	23.762	<0.0001	<0.0001
ATP5F1C	0.704	5.826	23.752	<0.0001	<0.0001
BIK	1.375	0.379	23.746	<0.0001	<0.0001
ACADM	0.661	4.435	23.723	<0.0001	<0.0001
FADS2	1.244	3.213	23.721	<0.0001	<0.0001
CLN8-AS1	-1.178	4.531	23.707	<0.0001	<0.0001
EBP	0.823	3.68	23.64	<0.0001	<0.0001
CKS1B	1.005	2.276	23.6	<0.0001	<0.0001
H2BC5	1.27	3.807	23.501	<0.0001	<0.0001
NASP	0.577	5.438	23.463	<0.0001	<0.0001
PDZK1IP1	-1.555	6.157	23.433	<0.0001	<0.0001
TUBB2B	-2.22	3.443	23.414	<0.0001	<0.0001
SNRPG	0.795	4.726	23.364	<0.0001	<0.0001
TOR3A	0.667	4.704	23.336	<0.0001	<0.0001
EPOP	1.162	-0.006	23.306	<0.0001	<0.0001
RFLNB	-1.165	6.994	23.255	<0.0001	<0.0001
HIBCH	0.869	2.839	23.222	<0.0001	<0.0001
NUP37	0.758	2.891	23.19	<0.0001	<0.0001
IGHV3-73	2.666	1.889	23.168	<0.0001	<0.0001
CCNG2	-0.64	6.301	23.109	<0.0001	<0.0001
GPR157	-0.959	2.165	23.074	<0.0001	<0.0001
IGKV2-30	2.074	3.736	23.072	<0.0001	<0.0001
ENSG00000228686	-1.699	-0.112	23.021	<0.0001	<0.0001
BAK1	0.85	5.347	23.012	<0.0001	<0.0001
HLA-H	1.587	4.985	22.986	<0.0001	<0.0001

CYCS	0.701	5.429	22.959	<0.0001	<0.0001
AARS1	0.758	5.274	22.932	<0.0001	<0.0001
PDIA6	1	6.453	22.93	<0.0001	<0.0001
ENSG00000262714	-1.046	4.203	22.91	<0.0001	<0.0001
POLL	-1.002	5.228	22.901	<0.0001	<0.0001
RPUSD1	-3.839	9.043	22.888	<0.0001	<0.0001
CASP7	0.723	4.293	22.879	<0.0001	<0.0001
SLC25A37	-1.238	10.743	22.829	<0.0001	<0.0001
IFITM3	1.673	9.374	22.82	<0.0001	<0.0001
APOBEC3B	1.345	3.792	22.818	<0.0001	<0.0001
C3AR1	0.926	5.622	22.815	<0.0001	<0.0001
OPTN	-0.997	7.461	22.812	<0.0001	<0.0001
WDR76	0.83	2.889	22.8	<0.0001	<0.0001
MRPS7	0.679	4.318	22.79	<0.0001	<0.0001
AKT1S1	-0.715	4.295	22.662	<0.0001	<0.0001
ODAD4	-1.919	1.743	22.636	<0.0001	<0.0001
BCL2L1	-1.656	8.363	22.523	<0.0001	<0.0001
FAM210B	-1.703	7.951	22.499	<0.0001	<0.0001
FAM98A	0.693	4.035	22.489	<0.0001	<0.0001
PSMA6	0.787	6.074	22.473	<0.0001	<0.0001
BLM	0.891	3.157	22.463	<0.0001	<0.0001
LRRC59	0.695	5.424	22.416	<0.0001	<0.0001
SFRP2	-2.758	0.61	22.392	<0.0001	<0.0001
CCT8	0.616	6.52	22.389	<0.0001	<0.0001
ENSG00000279386	-1.019	3.165	22.383	<0.0001	<0.0001
BMP8B	1.096	2.044	22.38	<0.0001	<0.0001
RANBP1	0.696	4.689	22.36	<0.0001	<0.0001
SEC24A	0.789	4.512	22.359	<0.0001	<0.0001
PCGF5	-0.878	7.709	22.341	<0.0001	<0.0001
ENSG00000285920	2.448	1.634	22.328	<0.0001	<0.0001

KNDC1	-1.826	1.234	22.291	<0.0001	<0.0001
TNS1	-1.708	6.306	22.266	<0.0001	<0.0001
TMEM164	-0.953	7.157	22.236	<0.0001	<0.0001
MCM3	0.581	5.306	22.218	<0.0001	<0.0001
TSPAN5-DT	-1.679	1.409	22.197	<0.0001	<0.0001
ANK1	-1.694	5.645	22.174	<0.0001	<0.0001
KIAA1958	1.094	2.402	22.109	<0.0001	<0.0001
CDC34	-1.164	6.611	22.098	<0.0001	<0.0001
PPM1A	-0.809	7.288	22.087	<0.0001	<0.0001
POMP	0.762	5.066	22.081	<0.0001	<0.0001
MT1E	1.369	0.881	22.033	<0.0001	<0.0001
BRCA1	0.754	3.554	22.004	<0.0001	<0.0001
IFI27L2	0.854	3.579	22	<0.0001	<0.0001
PSME2	0.866	6.917	21.958	<0.0001	<0.0001
CDH2	-2.106	1.177	21.953	<0.0001	<0.0001
SSR3	0.824	6.288	21.941	<0.0001	<0.0001
DSCC1	1.835	0.175	21.938	<0.0001	<0.0001
MRPS11	0.661	3.583	21.866	<0.0001	<0.0001
IGLV2-8	3.347	4.382	21.83	<0.0001	<0.0001
QPCTL	0.991	1.278	21.828	<0.0001	<0.0001
SIL1	0.907	3.575	21.813	<0.0001	<0.0001
ANKH	-0.751	5.686	21.779	<0.0001	<0.0001
ITGB7	0.713	5.769	21.773	<0.0001	<0.0001
PSME2P2	1.097	1.149	21.766	<0.0001	<0.0001
BLVRA	0.94	4.956	21.721	<0.0001	<0.0001
PPP3CA	-0.597	6.389	21.712	<0.0001	<0.0001
SELENOH	0.55	5.657	21.668	<0.0001	<0.0005
IGHV3-72	2.171	1.681	21.667	<0.0001	<0.0005
MDH1	0.583	5.262	21.65	<0.0001	<0.0005
ADIPOR1	-1.373	9.688	21.582	<0.0001	<0.0005

C3orf38	0.519	4.495	21.565	<0.0001	<0.0005
SNRPD1	0.732	4.13	21.558	<0.0001	<0.0005
C12orf4	0.703	3.342	21.547	<0.0001	<0.0005
P2RY6	1.504	0.854	21.534	<0.0001	<0.0005
BCAM	-3.327	0.933	21.526	<0.0001	<0.0005
CEP15	1.218	0.223	21.516	<0.0001	<0.0005
IFIT5	1.044	6.169	21.515	<0.0001	<0.0005
IFI27	3.476	7.363	21.511	<0.0001	<0.0005
PGM5	-2.119	2.49	21.507	<0.0001	<0.0005
RCC2	0.555	5.985	21.504	<0.0001	<0.0005
MAFB	0.642	5.492	21.491	<0.0001	<0.0005
CTSL	1.091	3.586	21.418	<0.0001	<0.0005
MED25	-0.791	6.597	21.407	<0.0001	<0.0005
CASP3	0.63	5.716	21.406	<0.0001	<0.0005
MRPL58	0.766	3.235	21.381	<0.0001	<0.0005
HYOU1	0.851	6.278	21.374	<0.0001	<0.0005
PREB	0.592	5.078	21.347	<0.0001	<0.0005
STT3A	0.792	5.91	21.331	<0.0001	<0.0005
RPS27L	0.657	4.409	21.31	<0.0001	<0.0005
OSTC	0.818	5.001	21.31	<0.0001	<0.0005
MPDU1	0.693	4.408	21.279	<0.0001	<0.0005
IGKV2D-29	2.122	1.027	21.264	<0.0001	<0.0005
RANP1	1.071	0.487	21.239	<0.0001	<0.0005
CUL1	0.582	5.539	21.232	<0.0001	<0.0005
NHLRC4	-5.378	8.347	21.23	<0.0001	<0.0005
IFI35	0.999	5.543	21.205	<0.0001	<0.0005
RTCB	0.609	5.275	21.195	<0.0001	<0.0005
ENSG00000260496	-5.393	7.639	21.189	<0.0001	<0.0005
TUBG1	0.961	2.75	21.128	<0.0001	<0.0005
DNA2	1.107	0.943	21.077	<0.0001	<0.0005

TEK	-2.094	0.638	21.042	<0.0001	<0.0005
PI4K2B	0.716	3.967	21.028	<0.0001	<0.0005
KLHDC7B	1.275	3.25	21.008	<0.0001	<0.0005
BCL2L12	0.831	2.768	20.995	<0.0001	<0.0005
PAQR4	0.801	2.664	20.994	<0.0001	<0.0005
NCAPH2	0.612	5.085	20.943	<0.0001	<0.0005
CHST12	0.676	4.925	20.929	<0.0001	<0.0005
NR6A1	-1.066	2.435	20.88	<0.0001	<0.0005
MRPS18B	0.622	4.676	20.831	<0.0001	<0.0005
FDXR	0.819	2.14	20.787	<0.0001	<0.0005
MAIP1	0.803	2.357	20.769	<0.0001	<0.0005
CACNA1H	-5.54	8.698	20.691	<0.0001	<0.0005
ZNG1A	0.648	3.899	20.691	<0.0001	<0.0005
SMIM24	-1.983	3.353	20.683	<0.0001	<0.0005
VANGL1	0.686	2.68	20.666	<0.0001	<0.0005
TOMM40L	0.63	3.036	20.642	<0.0001	<0.0005
LGMN	1.052	2.391	20.604	<0.0001	<0.0005
IFI44	1.436	3.531	20.601	<0.0001	<0.0005
OXCT1	0.621	4.238	20.594	<0.0001	<0.0005
ENSG00000285382	1.042	1.647	20.543	<0.0001	<0.0005
MAD2L1BP	0.631	4.24	20.519	<0.0001	<0.0005
SEC61G	0.919	3.971	20.506	<0.0001	<0.0005
CADM1	1.324	1.758	20.486	<0.0001	<0.0005
IGKV1-6	2.075	2.236	20.48	<0.0001	<0.0005
NDUFV2	0.675	5.126	20.434	<0.0001	<0.0005
SMC4	0.679	5.285	20.422	<0.0001	<0.0005
SELENOS	0.764	3.992	20.415	<0.0001	<0.0005
CRELD2	0.913	4.179	20.392	<0.0001	<0.0005
CCT2	0.612	5.513	20.262	<0.0001	<0.0005
SEL1L3	0.937	6.241	20.256	<0.0001	<0.0005

CCNC	0.709	4.583	20.19	<0.0001	<0.0005
BORA	0.896	2.065	20.186	<0.0001	<0.0005
UQCRQ	0.737	4.662	20.141	<0.0001	<0.0005
ENSG00000287642	-2.279	-0.244	20.137	<0.0001	<0.0005
WFIKKN1	-5.043	9.544	20.093	<0.0001	<0.0005
CARM1	-0.791	5.744	20.091	<0.0001	<0.0005
YY1AP1	-0.596	6.708	20.08	<0.0001	<0.0005
ALG5	0.849	3.583	20.073	<0.0001	<0.0005
SLC25A39	-1.656	10.174	20.016	<0.0001	<0.0005
SNCA	-2.116	8.023	20.001	<0.0001	<0.0005
GALM	0.759	4.314	19.97	<0.0001	<0.0005
MYL6B	1.116	1.83	19.948	<0.0001	<0.0005
SUB1	0.886	7.096	19.944	<0.0001	<0.0005
SEC61B	0.806	5.192	19.944	<0.0001	<0.0005
COX7A2	0.676	5.368	19.941	<0.0001	<0.0005
FECH	-1.809	5.526	19.931	<0.0001	<0.0005
SLC2A5	1.478	1.489	19.924	<0.0001	<0.0005
IFIT1	2.029	7.973	19.92	<0.0001	<0.0005
GMPR	-1.39	5.556	19.917	<0.0001	<0.0005
TUBB2A	-2.567	5.034	19.915	<0.0001	<0.0005
MCM8	0.841	2.5	19.907	<0.0001	<0.0005
NT5C3A	1	6.621	19.906	<0.0001	<0.0005
MXI1	-1.554	6.976	19.901	<0.0001	<0.0005
GLRX5	-1.419	6.043	19.888	<0.0001	<0.0005
ENSG00000293278	-2.017	-0.107	19.844	<0.0001	<0.0005
PHOSPHO1	-1.319	7.925	19.816	<0.0001	<0.0005
RGS16	1.516	0.557	19.804	<0.0001	<0.0005
GSPT1	-1.127	7.738	19.761	<0.0001	<0.0005
LINC02975	-0.947	4.145	19.727	<0.0001	<0.0005
MOXD1	1.765	0.057	19.726	<0.0001	<0.0005

RANBP10	-0.977	5.646	19.716	<0.0001	<0.0005
H2BC6-AS1	1.209	0.727	19.705	<0.0001	<0.0005
PSMF1	-0.811	7.289	19.682	<0.0001	<0.0005
CAMK1D	-0.917	6.574	19.652	<0.0001	<0.0005
IDH2	0.645	5.892	19.635	<0.0001	<0.0005
DDIT3	0.742	4.096	19.616	<0.0001	<0.0005
HSPE1	0.732	4.263	19.612	<0.0001	<0.0005
SCAMP5	1.231	1.69	19.591	<0.0001	<0.0005
GAS6	1.313	2.268	19.577	<0.0001	<0.0005
HVCN1	-0.627	5.965	19.573	<0.0001	<0.0005
EEF1E1	0.785	2.677	19.556	<0.0001	<0.0005
DNAJC9	0.755	4.397	19.538	<0.0001	<0.0005
EEF1AKMT4	1.108	0.788	19.533	<0.0001	<0.0005
ADGRE3	-0.898	6.793	19.512	<0.0001	<0.0005
SNRPGP10	0.899	0.798	19.511	<0.0001	<0.0005
ENSG00000293232	-5.132	8.616	19.494	<0.0001	<0.0005
IGHJ3	2.426	5.151	19.492	<0.0001	<0.0005
ELAPOR1	-1.545	5.929	19.477	<0.0001	<0.0005
CMPK2	1.808	5.742	19.466	<0.0001	<0.0005
FBXO9	-0.964	5.932	19.434	<0.0001	<0.0005
TOPORS	-0.596	5.816	19.419	<0.0001	<0.0005
SLC35B1	0.755	4.04	19.407	<0.0001	<0.0005
ENSG00000293331	-1.479	0.132	19.407	<0.0001	<0.0005
PSMA4	0.612	6.322	19.382	<0.0001	<0.0005
MTHFD1	0.65	4.306	19.369	<0.0001	<0.0005
EGR1	1.075	1.725	19.345	<0.0001	<0.0005
JAZF1	-0.775	5.254	19.31	<0.0001	<0.0005
ZBP1	0.912	5.784	19.254	<0.0001	<0.0005
FAM117B	-0.541	4.61	19.253	<0.0001	<0.0005
RFC4	0.927	2.438	19.244	<0.0001	<0.0005

ZNF395	-2.653	2.021	19.244	<0.0001	<0.0005
CCDC78	-4.86	9.65	19.221	<0.0001	<0.0005
SKI	-0.634	6.521	19.201	<0.0001	<0.0005
ENSG00000291048	-0.879	4.537	19.191	<0.0001	<0.0005
MPP1	-1.081	7.332	19.191	<0.0001	<0.0005
ENOSF1	0.998	3.28	19.108	<0.0001	<0.0005
CCT3	0.566	6.421	19.102	<0.0001	<0.0005
TIMM10	0.823	3.216	19.039	<0.0001	<0.0005
ISOC2	0.931	2.719	19.026	<0.0001	<0.0005
IGHA2	2.026	5.75	18.981	<0.0001	<0.0005
CNPPD1	-0.887	6.827	18.965	<0.0001	<0.0005
FARSB	0.664	4.01	18.938	<0.0001	<0.0005
WDR62	1.44	0.575	18.92	<0.0001	<0.0005
RPN2	0.779	7.131	18.913	<0.0001	<0.0005
MRPS18C	0.788	3.262	18.895	<0.0001	<0.0005
IFIT3	1.588	8.726	18.894	<0.0001	<0.0005
GPD2	0.497	4.73	18.844	<0.0001	<0.0005
ILF2	0.571	6.053	18.813	<0.0001	<0.0005
CAMKK1	-0.861	3.499	18.806	<0.0001	<0.0005
LMNB2	0.602	4.486	18.789	<0.0001	<0.0005
MGAT2	0.524	5.178	18.767	<0.0001	<0.0005
UBE2J1	0.859	7.314	18.735	<0.0001	<0.0005
CCDC13-AS2	-1.121	2.244	18.716	<0.0001	<0.0005
CHTF18	-4.367	9.979	18.702	<0.0001	<0.0005
RAN	0.614	6.658	18.698	<0.0001	<0.0005
IGLV1-40	2.776	3.493	18.696	<0.0001	<0.0005
ENSG00000292432	-5.437	7.629	18.69	<0.0001	<0.0005
TPGS2	-0.974	5.993	18.659	<0.0001	<0.0005
PSMB6	0.649	5.106	18.657	<0.0001	<0.0005
SERPING1	1.632	5.895	18.653	<0.0001	<0.0005

NFIX	-1.386	5.25	18.578	<0.0001	<0.0005
SEPTIN4	1.188	2.235	18.55	<0.0001	<0.0005
ECT2	0.852	2.463	18.531	<0.0001	<0.0005
ALAS2	-2.253	9.61	18.526	<0.0001	<0.0005
DPM2	-0.825	5.106	18.474	<0.0001	<0.0005
SPATA6	-0.839	2.057	18.466	<0.0001	<0.0005
ZRANB1	-0.65	4.965	18.46	<0.0001	<0.0005
TPPP	-1.14	1.886	18.427	<0.0001	<0.0005
NOMO1	0.6	5.457	18.409	<0.0001	<0.0005
DCAF12	-1.428	8.069	18.393	<0.0001	<0.0005
ACVRL1	1.396	0.28	18.381	<0.0001	<0.0005
COX5A	0.687	5.198	18.323	<0.0001	<0.0005
AFTPH-DT	1.108	0.852	18.323	<0.0001	<0.0005
CCDC167	1.007	2.863	18.319	<0.0001	<0.0005
ZNG1B	0.547	4.051	18.31	<0.0001	<0.0005
SLIRP	0.74	3.218	18.241	<0.0001	<0.0005
PSMA2	0.594	5.756	18.231	<0.0001	<0.0005
PPP1R15A	-0.649	6.732	18.224	<0.0001	<0.0005
XAF1	1.942	6.892	18.173	<0.0001	<0.0005
MAD2L2	0.665	4.623	18.168	<0.0001	<0.0005
MYO1D	1.154	2.826	18.167	<0.0001	<0.0005
DIPK1A	0.792	3.556	18.153	<0.0001	<0.0005
TMEM156	0.705	3.557	18.125	<0.0001	<0.0005
SRPRB	0.719	4.277	18.089	<0.0001	<0.0005
ENSG00000288156	-0.733	4.479	18.078	<0.0001	<0.0005
MYOF	0.844	4.883	18.076	<0.0001	<0.0005
ENSG00000293004	1.358	0.953	18.073	<0.0001	<0.0005
NDUFA4	0.61	5.369	18.068	<0.0001	<0.0005
NPL	-0.83	6.418	18.048	<0.0001	<0.0005
DHX58	0.962	4.188	18.043	<0.0001	<0.0005

COA6	0.67	3.263	18.023	<0.0001	<0.0005
AGRN	1.465	2.911	18.022	<0.0001	<0.0005
EPB42	-1.954	5.999	18.01	<0.0001	<0.0005
ALDH5A1	-0.838	4	18.006	<0.0001	<0.005
FAM72A	1.234	0.084	17.992	<0.0001	<0.005
MILR1	0.685	4.338	17.97	<0.0001	<0.005
INTS13	0.555	3.785	17.97	<0.0001	<0.005
SAR1B	0.583	4.46	17.959	<0.0001	<0.005
TMEM106A	0.63	3.004	17.937	<0.0001	<0.005
GBP6	1.353	1.561	17.935	<0.0001	<0.005
IFI44L	2.081	7.662	17.934	<0.0001	<0.005
YBX3	-1.612	8.834	17.928	<0.0001	<0.005
ENSG00000268903	-1.293	6.497	17.902	<0.0001	<0.005
MCOLN1	-0.684	4.701	17.896	<0.0001	<0.005
MS4A4A	1.102	2.637	17.865	<0.0001	<0.005
VRK2	0.586	3.744	17.844	<0.0001	<0.005
DTX4	-0.85	3.36	17.793	<0.0001	<0.005
PSMA3	0.533	5.266	17.755	<0.0001	<0.005
MRPL22	0.764	3.307	17.742	<0.0001	<0.005
MICB	0.55	4.791	17.684	<0.0001	<0.005
SLC25A4	1.004	1.911	17.68	<0.0001	<0.005
MRPL18	0.551	4.424	17.673	<0.0001	<0.005
MT1F	0.923	1.563	17.664	<0.0001	<0.005
TDRD7	0.65	4.962	17.664	<0.0001	<0.005
MTFR2	1.58	0.029	17.634	<0.0001	<0.005
PARPBP	1.153	0.898	17.613	<0.0001	<0.005
MTHFD1L	1.024	1.68	17.59	<0.0001	<0.005
NUCB2	0.58	5.216	17.589	<0.0001	<0.005
UCK2	0.779	2.702	17.577	<0.0001	<0.005
DMTN	-1.312	8.476	17.57	<0.0001	<0.005

MORF4L2	0.506	5.062	17.562	<0.0001	<0.005
ENSG00000284820	1.478	2.951	17.516	<0.0001	<0.005
FCHO1	-0.508	5.527	17.501	<0.0001	<0.005
MFSD2B	-1.372	3.439	17.482	<0.0001	<0.005
KEL	-1.428	2.347	17.462	<0.0001	<0.005
CNTNAP3	-1.719	4.331	17.46	<0.0001	<0.005
ARPC5L	0.581	4.688	17.457	<0.0001	<0.005
BTN2A2	0.538	4.276	17.456	<0.0001	<0.005
ENSG00000235105	-1.505	1.737	17.455	<0.0001	<0.005
PNRC2P1	0.8	0.338	17.452	<0.0001	<0.005
DHRS9	0.937	5.213	17.446	<0.0001	<0.005
SRSF8	-0.477	5.335	17.443	<0.0001	<0.005
TMPO-AS1	0.88	0.835	17.428	<0.0001	<0.005
CHAF1A	0.667	3.436	17.422	<0.0001	<0.005
CASTOR2	-0.803	3.166	17.336	<0.0001	<0.005
FADS1	0.755	3.029	17.335	<0.0001	<0.005
CIAO2B	0.545	4.393	17.327	<0.0001	<0.005
SELENOI	0.846	3.353	17.324	<0.0001	<0.005
SLC44A1	0.664	5.152	17.323	<0.0001	<0.005
RMI2	1.067	1.445	17.321	<0.0001	<0.005
MT1X	0.844	1.727	17.282	<0.0001	<0.005
ARHGAP11A	1.063	2.435	17.257	<0.0001	<0.005
TCP11L2	-0.879	5.726	17.251	<0.0001	<0.005
GGTA1	-1.036	3.989	17.25	<0.0001	<0.005
UBE2SP1	1.009	0.074	17.227	<0.0001	<0.005
SESN3	-0.759	6.881	17.226	<0.0001	<0.005
CLPTM1L	0.647	5.884	17.22	<0.0001	<0.005
GBP1	1.154	7.783	17.217	<0.0001	<0.005
ENSG00000289176	-1.128	3.762	17.213	<0.0001	<0.005
NDUFAB1	0.658	4.158	17.191	<0.0001	<0.005

PCSK1N	-1.321	1.706	17.187	<0.0001	<0.005
ST8SIA1	-0.975	2.098	17.181	<0.0001	<0.005
EPOR	-0.722	3.587	17.164	<0.0001	<0.005
ROMO1	0.756	3.451	17.162	<0.0001	<0.005
ST13	-0.593	7.145	17.161	<0.0001	<0.005
MPZL3	-0.76	6.304	17.12	<0.0001	<0.005
GNL2	0.519	4.798	17.106	<0.0001	<0.005
DTYMK	0.827	2.228	17.031	<0.0001	<0.005
PRDX6	-0.852	7.417	17.024	<0.0001	<0.005
PRKCA	-0.588	4.654	17.018	<0.0001	<0.005
POLA1	0.68	3.2	16.998	<0.0001	<0.005
ANXA2P2	0.868	1.859	16.995	<0.0001	<0.005
LARP7	0.445	5.165	16.975	<0.0001	<0.005
CNTNAP3P2	-2.304	0.677	16.938	<0.0001	<0.005
NET1	1.018	3.346	16.918	<0.0001	<0.005
MEOX1	-1.2	0.787	16.903	<0.0001	<0.005
FH	0.639	4.234	16.875	<0.0001	<0.005
TUBAP2	0.872	1.725	16.869	<0.0001	<0.005
GMPPA	0.673	3.996	16.852	<0.0001	<0.005
ENSG00000293141	-1.109	-0.196	16.849	<0.0001	<0.005
ATP5MC1	0.807	3.685	16.838	<0.0001	<0.005
ETV7	1.388	3.669	16.822	<0.0001	<0.005
RILP	-0.886	5.167	16.81	<0.0001	<0.005
CDCA4	0.67	2.687	16.804	<0.0001	<0.005
ZBTB18	-0.575	6.552	16.803	<0.0001	<0.005
LAG3	0.855	3.152	16.757	<0.0001	<0.005
MRPS12	0.656	3.366	16.749	<0.0001	<0.005
ENSG00000219928	0.938	0.447	16.728	<0.0001	<0.005
SHARPIN	-0.831	5.933	16.725	<0.0001	<0.005
PAICS	0.725	4.524	16.721	<0.0001	<0.005

CREB3L2	0.482	5.186	16.719	<0.0001	<0.005
HSPD1	0.583	6.508	16.717	<0.0001	<0.005
ITGB4	-1.863	1.645	16.691	<0.0001	<0.005
HSPA13	0.77	4.529	16.683	<0.0001	<0.005
FAM30A	1.172	4.104	16.68	<0.0001	<0.005
SELENBP1	-2.176	6.466	16.668	<0.0001	<0.005
HAUS1	0.668	3.29	16.654	<0.0001	<0.005
IPMK	-0.744	5.647	16.622	<0.0001	<0.005
DYNLT2B	0.848	0.572	16.618	<0.0001	<0.005
LARP4	0.537	4.344	16.615	<0.0001	<0.005
FIRRM	0.695	2.105	16.595	<0.0001	<0.005
PPA1	0.662	5.366	16.585	<0.0001	<0.005
DDX11L2	-1.199	2.779	16.581	<0.0001	<0.005
PPM1F	-0.745	6.854	16.537	<0.0001	<0.005
DARS2	0.544	3.261	16.503	<0.0001	<0.005
GART	0.47	4.977	16.493	<0.0001	<0.005
ATG9A	-0.661	5.223	16.489	<0.0001	<0.005
S100B	2.335	2.458	16.471	<0.0001	<0.005
AATK	-0.737	6.185	16.467	<0.0001	<0.005
RFC3	0.833	2.103	16.455	<0.0001	<0.005
PTAFR	-0.762	8.586	16.454	<0.0001	<0.005
APOBEC3F	0.574	3.884	16.427	<0.0001	<0.005
LAMC1	1.14	2.992	16.414	<0.0001	<0.005
SLC4A1	-2.009	8.259	16.383	<0.0001	<0.005
FAXDC2	-1.107	5.428	16.381	<0.0001	<0.005
GINS3	0.912	1.15	16.356	<0.0001	<0.005
RPGRIP1	-0.85	2.761	16.343	<0.0001	<0.005
LINC02887	-1.039	4.378	16.325	<0.0001	<0.005
SLC3A2	0.49	5.193	16.312	<0.0001	<0.005
DCTPP1	0.666	3.108	16.291	<0.0001	<0.005

CDC7	0.866	2.426	16.287	<0.0001	<0.005
FOXO3	-0.849	6.876	16.274	<0.0001	<0.005
PPIAP22	0.582	4.749	16.27	<0.0001	<0.005
TIMM8B	0.652	4.086	16.27	<0.0001	<0.005
RARS1	0.45	4.595	16.259	<0.0001	<0.005
BHLHE41	1.318	1.107	16.257	<0.0001	<0.005
METTL5	0.648	3.256	16.242	<0.0001	<0.005
SIRPB1	-0.787	8.205	16.228	<0.0001	<0.005
CYC1	0.556	4.706	16.195	<0.0001	<0.005
CNTNAP3B	-1.806	1.623	16.191	<0.0001	<0.005
DNAJC15	0.568	4.482	16.186	<0.0001	<0.005
CDK2	0.641	3.111	16.145	<0.0001	<0.005
SNRPGP15	1.112	-0.552	16.127	<0.0001	<0.005
ENSG00000250644	-4.032	3.893	16.118	<0.0001	<0.005
ZNF496-DT	1.086	0.42	16.118	<0.0001	<0.005
SLC25A19	0.778	2.175	16.104	<0.0001	<0.005
PROB1	1.114	0.751	16.101	<0.0001	<0.005
MICAL2	-1.102	7.347	16.098	<0.0001	<0.005
TMEM208	0.704	3.635	16.097	<0.0001	<0.005
VIPR1	-0.644	3.705	16.09	<0.0001	<0.005
RPA3	0.627	3.316	16.085	<0.0001	<0.005
PSMD14	0.652	4.624	16.083	<0.0001	<0.005
MRPL15	0.604	3.623	16.08	<0.0001	<0.005
FAM20C	1.051	2.714	16.075	<0.0001	<0.005
IDH1	0.505	5.096	16.07	<0.0001	<0.005
CHPT1	-0.94	5.963	16.031	<0.0001	<0.005
LDLR	0.747	4.071	16.027	<0.0001	<0.005
SPCS2	0.646	5.525	16.022	<0.0001	<0.005
COA4	0.595	3.629	16.019	<0.0001	<0.005
R3HDM4	-0.925	9.357	16.014	<0.0001	<0.005

LINC02772	-1.157	2.8	15.996	<0.0001	<0.005
GALK2	0.549	3.403	15.987	<0.0001	<0.005
ZER1	-0.655	6.172	15.971	<0.0001	<0.005
IL1RAP	-1.002	6.184	15.959	<0.0001	<0.005
EPHB4	-1.274	4.114	15.946	<0.0001	<0.005
NECTIN1	-0.666	3.927	15.935	<0.0001	<0.005
SGO1	1.173	0.412	15.932	<0.0001	<0.005
TRIM7	0.891	1.596	15.924	<0.0001	<0.005
BOLA3	0.774	1.899	15.922	<0.0001	<0.005
HID1	1.415	0.975	15.918	<0.0001	<0.005
SLC9A9	0.481	4.028	15.892	<0.0001	<0.005
SPATA24	0.792	0.793	15.876	<0.0001	<0.005
NSD2	0.572	4.752	15.863	<0.0001	<0.005
FBXO6	1.034	3.364	15.86	<0.0001	<0.005
HARBI1	0.641	1.707	15.858	<0.0001	<0.005
TBC1D17	-0.525	4.698	15.84	<0.0001	<0.005
MCTS1	0.492	4.627	15.838	<0.0001	<0.005
RRBP1	0.562	6.589	15.8	<0.0001	<0.005
CMTR1	0.61	5.488	15.793	<0.0001	<0.005
LYL1	-0.812	6.977	15.788	<0.0001	<0.005
ENSG00000273284	-0.995	-0.582	15.783	<0.0001	<0.005
BYSL	0.71	2.11	15.782	<0.0001	<0.005
RPL39L	1.072	0.859	15.777	<0.0001	<0.005
HGSNAT	-0.401	5.368	15.757	<0.0001	<0.005
NDUFA8	0.62	3.713	15.752	<0.0001	<0.005
UBE4B	-0.424	5.763	15.707	<0.0001	<0.005
XRCC2	1.302	-0.217	15.682	<0.0001	<0.005
EIF2S1	0.442	5.166	15.68	<0.0001	<0.005
TMED9	0.587	6.048	15.654	<0.0001	<0.005
PFN2	-1.037	0.726	15.638	<0.0001	<0.005

PITPNA	-0.534	6.598	15.633	<0.0001	<0.005
SRP19	0.447	4.438	15.615	<0.0001	<0.005
HMGB1P1	0.737	0.26	15.608	<0.0001	<0.005
FASTKD1	0.692	2.966	15.593	<0.0001	<0.005
GCAT	-1.588	1.19	15.587	<0.0001	<0.005
ALG8	0.679	3.591	15.578	<0.0001	<0.005
DCPS	0.623	4.365	15.577	<0.0001	<0.005
MKRN1	-0.955	8.537	15.575	<0.0001	<0.005
NQO1	0.957	0.702	15.563	<0.0001	<0.005
MIR600HG	-0.967	1.946	15.548	<0.0001	<0.005
ENSG00000288997	-1.063	1.122	15.488	<0.0001	<0.005
RNF123	-0.745	5.07	15.487	<0.0001	<0.005
AKR1A1	0.604	4.766	15.48	<0.0001	<0.005
ENSG00000282416	-0.941	4.552	15.472	<0.0001	<0.005
HBD	-3.111	10.73	15.466	<0.0001	<0.005
TIMM23	0.578	4.191	15.452	<0.0001	<0.005
TMEM60	0.623	3.511	15.434	<0.0001	<0.005
PARP11	0.526	3.903	15.431	<0.0001	<0.005
TMX1	0.425	5.095	15.431	<0.0001	<0.005
CXCL5	-1.456	4.486	15.431	<0.0001	<0.005
PEX2	0.407	4.681	15.422	<0.0001	<0.005
RBM38	-0.998	7.896	15.413	<0.0001	<0.005
ENSG00000285906	0.823	1.467	15.407	<0.0001	<0.005
EEPD1	-0.551	4.534	15.384	<0.0001	<0.005
IGHV3-13	2.102	1.209	15.372	<0.0001	<0.005
ENTPD7	0.615	3.185	15.356	<0.0001	<0.005
BLVRB	-0.971	7.157	15.343	<0.0001	<0.005
BATF	0.549	4.34	15.341	<0.0001	<0.005
PARP2	0.742	2.385	15.308	<0.0001	<0.005
WEE1	0.624	2.926	15.29	<0.0001	<0.005

EPB41L3	0.66	4.907	15.266	<0.0001	<0.005
RSAD2	2.171	7.58	15.258	<0.0001	<0.005
COX7B	0.602	5.687	15.24	<0.0001	<0.005
TIPIN	0.873	1.04	15.225	<0.0001	<0.005
ENSG00000282988	0.934	3.503	15.223	<0.0001	<0.005
H4C15	0.829	3.16	15.209	<0.0001	<0.005
GARS1	0.546	5.44	15.206	<0.0001	<0.005
AIFM1	0.522	4.133	15.194	<0.0001	<0.005
LINC02471	1.25	1.977	15.188	<0.0001	<0.005
MRPS22	0.484	4.672	15.173	<0.0001	<0.005
ENSG00000290027	1.22	1.913	15.147	<0.0001	<0.005
ADGRE2	-0.79	7.814	15.131	<0.0005	<0.005
MYCT1	1.169	1.421	15.13	<0.0005	<0.005
KLF1	-1.407	3.528	15.124	<0.0005	<0.005
IFIT2	1.123	9.023	15.112	<0.0005	<0.005
ENSG00000255224	1.032	0.384	15.073	<0.0005	<0.005
MRPL1	0.618	3.152	15.072	<0.0005	<0.005
ATP13A1	0.495	5.299	15.068	<0.0005	<0.005
LINC01890	-0.895	2.223	15.057	<0.0005	<0.005
LINC01002	-1.202	3.372	15.048	<0.0005	<0.005
SLFN11	0.501	5.105	15.044	<0.0005	<0.005
ORC3	0.482	3.796	15.043	<0.0005	<0.005
IMPA2	-0.85	5.435	15.042	<0.0005	<0.005
GMPPB	0.797	4.171	15.027	<0.0005	<0.005
ENSG00000250746	0.96	1.217	15.016	<0.0005	<0.005
RPL26L1	0.67	2.907	15.009	<0.0005	<0.005
PNPLA2	-0.603	6.689	14.977	<0.0005	<0.005
CDKN2C	0.72	2.59	14.976	<0.0005	<0.005
GATB	0.642	2.777	14.973	<0.0005	<0.005
KLF7	-0.62	6.126	14.968	<0.0005	<0.005

PNPT1	0.829	3.592	14.93	<0.0005	<0.005
PSMB5	0.556	3.974	14.92	<0.0005	<0.005
NCAL1	0.553	3.783	14.917	<0.0005	<0.005
ATP5MK	0.573	4.825	14.91	<0.0005	<0.005
ATOSB	-0.567	6.176	14.909	<0.0005	<0.005
ENSG00000278133	-0.702	1.812	14.901	<0.0005	<0.005
ZNF684	0.812	1.695	14.888	<0.0005	<0.005
ST13P4	-1.057	-1.106	14.88	<0.0005	<0.005
TCEAL9	1.125	0.174	14.877	<0.0005	<0.005
MRPL14	0.589	3.145	14.868	<0.0005	<0.005
RMND5A	-0.557	6.709	14.861	<0.0005	<0.005
MAST3	-0.62	6.839	14.861	<0.0005	<0.005
RUNDC3A	-1.51	5.559	14.853	<0.0005	<0.005
C11orf24	0.595	3.72	14.852	<0.0005	<0.005
PSMC3	0.595	5.504	14.848	<0.0005	<0.005
ENSG00000276136	-0.858	4.216	14.841	<0.0005	<0.005
KIT	-1	1.577	14.835	<0.0005	<0.005
CSTF3	0.468	3.723	14.813	<0.0005	<0.005
DLD	0.385	5.308	14.813	<0.0005	<0.005
FANCL	0.489	4.154	14.808	<0.0005	<0.005
TMEM126A	0.648	2.674	14.781	<0.0005	<0.005
ANGPT1	-1.101	2.029	14.775	<0.0005	<0.005
VDAC1	0.535	5.492	14.769	<0.0005	<0.005
CA3-AS1	-1.189	1.2	14.756	<0.0005	<0.005
CNIH3	-1.041	1.799	14.75	<0.0005	<0.005
PRPS2	0.443	3.977	14.747	<0.0005	<0.005
FOXO1	-0.432	5.631	14.743	<0.0005	<0.005
ENSG00000260022	-4.314	8.836	14.743	<0.0005	<0.005
AP1S1	0.629	3.15	14.73	<0.0005	<0.005
PBX1	-0.872	3.43	14.729	<0.0005	<0.005

PSME3IP1	0.479	6.3	14.723	<0.0005	<0.005
SNRNP25	0.794	2.764	14.71	<0.0005	<0.005
ALDH18A1	0.574	4.329	14.699	<0.0005	<0.005
LGALS3	-0.858	7.24	14.65	<0.0005	<0.005
SAE1	0.477	5.202	14.642	<0.0005	<0.005
MYL4	-1.094	6.236	14.616	<0.0005	<0.005
RNF144B	-0.612	6.072	14.606	<0.0005	<0.005
CPSF3	0.512	4.529	14.6	<0.0005	<0.005
SERTAD2	-0.45	5.494	14.6	<0.0005	<0.005
TRAJ16	1.952	0.744	14.589	<0.0005	<0.005
SRM	0.744	4.145	14.58	<0.0005	<0.005
KIF24	1.26	0.062	14.563	<0.0005	<0.005
CXCL10	1.592	1.682	14.543	<0.0005	<0.005
SLC48A1	-0.689	3.691	14.535	<0.0005	<0.005
CALR	0.71	7.937	14.521	<0.0005	<0.005
TRIM5	0.647	4.928	14.514	<0.0005	<0.005
DCAF13	0.458	4.14	14.469	<0.0005	<0.005
TAF9	0.433	4.518	14.463	<0.0005	<0.005
GLRX3	0.508	4.188	14.463	<0.0005	<0.005
ACP2	0.579	3.824	14.463	<0.0005	<0.005
IGHV3-15	1.768	1.343	14.462	<0.0005	<0.005
BRD4	-0.438	6.676	14.448	<0.0005	<0.005
HSP90AA2P	0.724	0.834	14.445	<0.0005	<0.005
IFT25	0.518	3.52	14.444	<0.0005	<0.005
COMMD1	0.653	2.981	14.44	<0.0005	<0.005
DNAJB9	0.61	4.188	14.44	<0.0005	<0.005
HSP90AA3P	0.77	0.111	14.438	<0.0005	<0.005
RPLP0P6	0.682	4.222	14.434	<0.0005	<0.005
RAB3IL1	-1.212	1.312	14.432	<0.0005	<0.005
ENSG00000279330	-1.237	-0.36	14.41	<0.0005	<0.005

KDM4B	-0.655	6.731	14.403	<0.0005	<0.005
SUV39H2	0.765	1.622	14.395	<0.0005	<0.005
RINT1	0.547	3.308	14.392	<0.0005	<0.005
MRPL47	0.564	3.78	14.386	<0.0005	<0.005
ANPEP	-0.805	8.516	14.377	<0.0005	<0.005
SEC14L1	-0.668	8.917	14.374	<0.0005	<0.005
RPGR	-0.66	3.496	14.364	<0.0005	<0.005
LINC02555	1.241	2.54	14.363	<0.0005	<0.005
RIN2	0.775	4.006	14.358	<0.0005	<0.005
AHSP	-1.409	6.032	14.335	<0.0005	<0.005
EPRS1	0.49	6.355	14.317	<0.0005	<0.005
GFOD2	-0.552	4.02	14.309	<0.0005	<0.005
SEC61A1	0.585	7.149	14.282	<0.0005	<0.005
ASCC1	0.445	3.651	14.279	<0.0005	<0.005
SEC14L3	-1.931	2.08	14.274	<0.0005	<0.005
CHMP5	0.588	5.906	14.273	<0.0005	<0.005
SPDL1	0.809	2.205	14.273	<0.0005	<0.005
SIMALR	1.779	0.55	14.269	<0.0005	<0.005
SMTNL1	1.43	1.424	14.243	<0.0005	<0.005
MDS2	-1.268	-0.113	14.219	<0.0005	<0.005
TMEM19	0.486	4.128	14.212	<0.0005	<0.005
MAPK6	0.521	4.282	14.21	<0.0005	<0.005
BZW1P2	0.534	2.382	14.208	<0.0005	<0.005
TONSL	0.716	1.654	14.202	<0.0005	<0.005
GTF2IP4	-0.646	6.733	14.19	<0.0005	<0.005
SLC43A3	0.475	4.782	14.189	<0.0005	<0.005
ACTL6A	0.48	3.967	14.187	<0.0005	<0.005
NDUFB8	0.592	5.426	14.171	<0.0005	<0.005
ABCC13	-1.79	3.03	14.168	<0.0005	<0.005
NRAS	0.402	5.762	14.166	<0.0005	<0.005

ARAP3	-0.737	6.12	14.155	<0.0005	<0.005
C11orf98	0.537	4.461	14.129	<0.0005	<0.005
IL15RA	0.674	2.883	14.127	<0.0005	<0.005
RHBDD3	0.756	1.882	14.112	<0.0005	<0.005
CEP68	-0.504	4.13	14.111	<0.0005	<0.005
HMGCS1	0.511	4.218	14.109	<0.0005	<0.005
TMEM62	0.706	3.122	14.089	<0.0005	<0.005
DYNC1I2	0.399	5.486	14.078	<0.0005	<0.005
NDUFS3	0.568	4.305	14.077	<0.0005	<0.005
RYBP	-0.587	6.538	14.071	<0.0005	<0.005
TOB1	-0.5	5.362	14.07	<0.0005	<0.005
BEND2	-1.156	3.657	14.068	<0.0005	<0.005
IBTK	0.528	5.089	14.064	<0.0005	<0.005
EIF2B2	0.489	3.915	14.057	<0.0005	<0.005
H4C14	0.821	3.29	14.052	<0.0005	<0.005
PPIL1	0.635	3.31	14.043	<0.0005	<0.005
ARV1	0.582	2.753	14.032	<0.0005	<0.005
EMP1	1.148	1.858	14.007	<0.0005	<0.005
ENSG00000250138	1.262	3.145	13.997	<0.0005	<0.005
NDUFA6	0.603	5.159	13.993	<0.0005	<0.005
GREM2	-1.693	-0.433	13.986	<0.0005	<0.005
CEROX1	-4.162	9.726	13.981	<0.0005	<0.005
ENSG00000286288	-0.891	2.308	13.974	<0.0005	<0.005
MRPL12	0.74	3.695	13.974	<0.0005	<0.005
MRPL40	0.705	3.294	13.968	<0.0005	<0.005
ENSG00000287855	-4.445	10.517	13.959	<0.0005	<0.005
DPCD	-1.231	2.179	13.95	<0.0005	<0.005
RCC1	0.52	3.955	13.95	<0.0005	<0.005
CHEK2	0.762	1.701	13.942	<0.0005	<0.005
PDXP	1.239	2.316	13.914	<0.0005	<0.005

ITLN1	-1.648	1.887	13.9	<0.0005	<0.005
FMC1	0.701	1.354	13.893	<0.0005	<0.005
SEM1	0.639	4.305	13.89	<0.0005	<0.005
NPRL3	-0.964	6.92	13.884	<0.0005	<0.005
ALG1	0.556	3.666	13.879	<0.0005	<0.005
TMX2	0.672	3.17	13.866	<0.0005	<0.005
COMMD4	0.537	3.316	13.866	<0.0005	<0.005
SLC25A12	0.501	3.513	13.861	<0.0005	<0.005
BNIP3L	-1.072	7.957	13.854	<0.0005	<0.005
ADPRH	0.605	3.796	13.853	<0.0005	<0.005
BPNT1	0.482	3.235	13.851	<0.0005	<0.005
INKA2	-0.808	6.908	13.849	<0.0005	<0.005
IFI16	0.667	8.669	13.847	<0.0005	<0.005
LMF1	-4.088	11.749	13.813	<0.0005	<0.005
COX6C	0.578	5.203	13.806	<0.0005	<0.005
ENSG00000232807	1.202	2.019	13.792	<0.0005	<0.005
CTPS1	0.617	2.93	13.788	<0.0005	<0.005
TRIP6	0.7	1.717	13.779	<0.0005	<0.005
DPP3	0.535	3.979	13.775	<0.0005	<0.005
SAMD4A	0.96	2.268	13.765	<0.0005	<0.005
OAS3	1.558	7.961	13.745	<0.0005	<0.005
DYNC1I2P1	0.782	-0.412	13.733	<0.0005	<0.005
SNRPB	0.428	5.913	13.726	<0.0005	<0.005
GTSF1	0.834	1.198	13.721	<0.0005	<0.005
IDE	0.503	4.36	13.715	<0.0005	<0.005
RFT1	0.516	3.661	13.711	<0.0005	<0.005
COPB1	0.364	6.404	13.698	<0.0005	<0.005
ENSG00000290385	-0.955	5.243	13.696	<0.0005	<0.005
ZDHHC12-DT	0.795	1.105	13.691	<0.0005	<0.005
ENSG00000255320	-0.589	3.321	13.686	<0.0005	<0.005

WDR90	-4.428	10.43	13.683	<0.0005	<0.005
TMEM65	-0.451	4.344	13.682	<0.0005	<0.005
FCHO2	-0.683	4.446	13.68	<0.0005	<0.005
NDC1	0.561	3.538	13.68	<0.0005	<0.005
GYPC	-1.183	10.114	13.662	<0.0005	<0.005
RWDD2B	0.732	2.276	13.657	<0.0005	<0.005
MASTL	0.786	2.589	13.638	<0.0005	<0.005
ENSG00000255801	-1.995	0.244	13.635	<0.0005	<0.005
UTP4	0.513	3.566	13.628	<0.0005	<0.005
COMMD3	0.53	4.424	13.627	<0.0005	<0.005
TRIM22	0.767	8.119	13.618	<0.0005	<0.005
ADH5	0.428	5.318	13.609	<0.0005	<0.005
BRICD5	-1.312	1.732	13.609	<0.0005	<0.005
MPZL1	-0.923	6.39	13.608	<0.0005	<0.005
TM7SF3	0.375	4.79	13.59	<0.0005	<0.005
MX1	1.406	8.029	13.585	<0.0005	<0.005
SMARCB1	0.636	5.067	13.582	<0.0005	<0.005
KCNH2	-1.013	1.596	13.581	<0.0005	<0.005
OSBPL1A	-0.714	3.065	13.575	<0.0005	<0.005
WASHC3	0.503	4.2	13.554	<0.0005	<0.005
SREBF2	0.418	6.053	13.529	<0.0005	<0.005
ENSG00000277534	0.859	-0.762	13.517	<0.0005	<0.005
CDKN2A	1.121	0.69	13.515	<0.0005	<0.005
ACSL5	0.398	5.756	13.506	<0.0005	<0.005
COQ5	0.54	3.46	13.504	<0.0005	<0.005
YBX1	-0.824	10.035	13.502	<0.0005	<0.005
ATG4A	0.502	3.394	13.491	<0.0005	<0.005
CNKSR1	1.826	0.392	13.483	<0.0005	<0.005
ST13P6	-0.79	-0.561	13.481	<0.0005	<0.005
PPP1CB-DT	-1.234	-0.374	13.475	<0.0005	<0.005

TMCO3	-0.546	4.742	13.458	<0.0005	<0.005
MPG	0.511	3.928	13.457	<0.0005	<0.005
MCM5	0.568	5.242	13.45	<0.0005	<0.005
MTDH	0.466	6.665	13.444	<0.0005	<0.005
VIT	1.51	-0.121	13.44	<0.0005	<0.005
MAP7	-1.092	2.234	13.419	<0.0005	<0.005
PARM1	1.103	2.056	13.403	<0.0005	<0.005
ZNF746	-0.516	5.954	13.398	<0.0005	<0.005
NEDD4L	-1.091	3.114	13.398	<0.0005	<0.005
UBXN6	-1.055	8.877	13.395	<0.0005	<0.005
SLC12A9	-0.638	6.188	13.373	<0.0005	<0.005
CDK4	0.577	3.632	13.371	<0.0005	<0.005
IGHG2	1.23	7.045	13.363	<0.0005	<0.005
UNC13B	1.339	0.55	13.359	<0.0005	<0.005
VDAC3	0.47	5.798	13.353	<0.0005	<0.005
RNF11	-0.878	6.987	13.321	<0.0005	<0.005
ERH	0.502	5.167	13.316	<0.0005	<0.005
FOXO4	-0.667	6.613	13.306	<0.0005	<0.005
ENSG00000259529	0.604	6.647	13.303	<0.0005	<0.005
IFIT1B	-1.784	5.049	13.283	<0.0005	<0.005
KAT8	-0.391	5.341	13.272	<0.0005	<0.005
RPP25	0.83	1.806	13.266	<0.0005	<0.005
FUT8	0.674	3.517	13.258	<0.0005	<0.005
LINC01001	-0.986	5.076	13.25	<0.0005	<0.005
GDI1	-0.438	6.558	13.25	<0.0005	<0.005
USP7-AS1	-1.081	1.547	13.218	<0.0005	<0.005
CIAPIN1	0.431	3.899	13.213	<0.0005	<0.005
NUS1P1	0.629	2.382	13.195	<0.0005	<0.005
TAP1	0.612	8.407	13.194	<0.0005	<0.005
UQCRFS1P1	0.751	1.249	13.191	<0.0005	<0.005

LDHA	0.633	7.704	13.186	<0.0005	<0.005
SOX8	-4.183	9.65	13.179	<0.0005	<0.005
TRDC	0.85	5.989	13.177	<0.0005	<0.005
FBXL20	-0.468	4.96	13.177	<0.0005	<0.005
PAK1IP1	0.628	2.76	13.174	<0.0005	<0.005
ENSG00000289887	-1.182	1.86	13.163	<0.0005	<0.005
KIF20B	0.591	3.423	13.161	<0.0005	<0.005
ABCC5	-0.763	4.535	13.159	<0.0005	<0.005
PSMD5	0.373	4.457	13.155	<0.0005	<0.005
PDCD2L	0.677	1.096	13.149	<0.0005	<0.005
DSC2	-1.01	5.301	13.141	<0.0005	<0.005
YIF1A	0.579	3.288	13.139	<0.0005	<0.005
HECW2-AS1	-0.77	1.211	13.132	<0.0005	<0.005
TRIM38	0.504	6.876	13.129	<0.0005	<0.005
ICAM2	0.496	4.909	13.124	<0.0005	<0.005
ENSG00000277830	-2.686	1.413	13.122	<0.0005	<0.005
PSMG1	0.644	2.579	13.114	<0.0005	<0.005
PPP2R1B	0.452	3.963	13.111	<0.0005	<0.005
CKAP5	0.407	5.473	13.104	<0.0005	<0.005
IGLV1-47	2.314	2.345	13.103	<0.0005	<0.005
DDX1	0.499	5.131	13.102	<0.0005	<0.005
CSE1L	0.424	5.212	13.089	<0.0005	<0.005
IRS2	-1.029	6.104	13.087	<0.0005	<0.005
ENSG00000290776	-1.114	1.144	13.082	<0.0005	<0.005
TJP1	0.617	2.561	13.069	<0.0005	<0.005
PLAAT3	0.711	3.004	13.063	<0.0005	<0.005
SRP72	0.445	6.209	13.06	<0.0005	<0.005
EED	0.405	3.809	13.033	<0.0005	<0.005
KNTC1	0.725	3.086	13.031	<0.0005	<0.005
PSPH	0.835	1.678	13.004	<0.0005	<0.005

GFPT1	0.507	4.245	13.004	<0.0005	<0.005
BARD1	0.668	2.213	12.992	<0.0005	<0.005
EIPR1	0.536	3.515	12.988	<0.0005	<0.005
H2BC17	1.667	2.538	12.96	<0.0005	<0.005
BPGM	-1.83	6.709	12.955	<0.0005	<0.005
SIRPB3P	-1.006	-0.7	12.954	<0.0005	<0.005
CCDC125	-0.593	4.401	12.95	<0.0005	<0.005
ENSG00000261338	-0.745	2.741	12.945	<0.0005	<0.005
MCEE	0.768	1.557	12.944	<0.0005	<0.005
SNX4	0.396	4.052	12.942	<0.0005	<0.005
ENSG00000290937	-0.965	6.225	12.936	<0.0005	<0.005
FLAD1	0.446	3.907	12.932	<0.0005	<0.005
AHCY	0.639	4.67	12.931	<0.0005	<0.005
RNF181	0.542	5.001	12.925	<0.0005	<0.005
INE1	-1.087	-0.267	12.923	<0.0005	<0.005
XKR8	-0.564	5.717	12.922	<0.0005	<0.005
PALM2AKAP2	0.693	5.467	12.896	<0.0005	<0.005
STYXL1	0.603	3.892	12.885	<0.0005	<0.005
HAX1	0.486	5.451	12.881	<0.0005	<0.005
CENPJ	0.831	1.762	12.874	<0.0005	<0.005
SLC35F2	0.762	2.12	12.863	<0.0005	<0.005
RTCA	0.431	4.122	12.857	<0.0005	<0.005
ADAMTS5	-1.138	0.063	12.855	<0.0005	<0.005
NDUFB4	0.494	5.122	12.854	<0.0005	<0.005
PSMD8	0.478	6.051	12.854	<0.0005	<0.005
PDCD5	0.523	3.835	12.841	<0.0005	<0.005
MRPL35	0.425	3.874	12.832	<0.0005	<0.005
MRPS31	0.491	3.322	12.826	<0.0005	<0.005
PUM3	0.48	3.594	12.814	<0.0005	<0.005
HSD17B10	0.589	4.706	12.813	<0.0005	<0.005

CBX7	-0.412	5.907	12.812	<0.0005	<0.005
ENSG00000234117	-1.25	1.093	12.811	<0.0005	<0.005
BSG	-0.773	8.417	12.807	<0.0005	<0.005
MADCAM1	-1.644	0.512	12.789	<0.0005	<0.005
CENPH	0.712	1.822	12.785	<0.0005	<0.005
CDK19	-0.478	5.639	12.766	<0.0005	<0.005
MED8	0.428	4.399	12.765	<0.0005	<0.005
CYSLTR1	0.587	5.288	12.757	<0.0005	<0.005
PPP3R1	-0.414	6.642	12.752	<0.0005	<0.005
OR51AB1P	-1.089	5.843	12.731	<0.0005	<0.005
CCN3	-1.208	3.987	12.729	<0.0005	<0.005
PRR11	0.7	3.571	12.719	<0.0005	<0.005
NDUFB3	0.579	5.104	12.714	<0.0005	<0.005
C17orf80	0.466	3.588	12.712	<0.0005	<0.005
ENSG00000286342	-1.804	0.839	12.711	<0.0005	<0.005
RPF2	0.548	3.26	12.703	<0.0005	<0.005
MIR6506	-0.695	7.461	12.68	<0.0005	<0.005
RNASE2CP	1.185	0.877	12.67	<0.0005	<0.005
SPX	-1.249	2.767	12.668	<0.0005	<0.005
WNT5B	1.144	0.482	12.666	<0.0005	<0.005
OTUD1	-0.406	4.728	12.662	<0.0005	<0.005
PRIM2	0.557	2.49	12.631	<0.0005	<0.005
KDELR2	0.525	5.473	12.631	<0.0005	<0.005
CAPN5	-0.763	2.441	12.623	<0.0005	<0.005
ENSG00000291144	0.798	4.173	12.609	<0.0005	<0.005
FLT1	-1.37	1.326	12.609	<0.0005	<0.005
KIAA0232	-0.518	6.584	12.598	<0.0005	<0.005
LRRC75A	-0.763	3.38	12.597	<0.0005	<0.005
EPB41	-0.725	8.58	12.596	<0.0005	<0.005
DAD1	0.582	5.871	12.588	<0.0005	<0.005

LINC01485	1.26	-0.479	12.538	<0.0005	<0.05
ATP5PF	0.593	5.187	12.538	<0.0005	<0.05
PRDX3	0.417	5.578	12.534	<0.0005	<0.05
ATF3	0.98	1.777	12.522	<0.0005	<0.05
NNT	0.416	5.282	12.518	<0.0005	<0.05
MTFP1	0.677	2.808	12.515	<0.0005	<0.05
TSPAN18	-0.618	4.504	12.512	<0.0005	<0.05
PGAM1P8	-1.124	2.205	12.501	<0.0005	<0.05
RNF10	-0.915	8.207	12.498	<0.0005	<0.05
FDPS	0.446	5.261	12.487	<0.0005	<0.05
ANXA2	0.624	8.123	12.484	<0.0005	<0.05
TMEM147	0.584	4.071	12.473	<0.0005	<0.05
EIF2B3	0.637	2.768	12.471	<0.0005	<0.05
GTF2IP1	-0.544	6.502	12.467	<0.0005	<0.05
CCDC90B	0.399	4.456	12.467	<0.0005	<0.05
ENSG00000285851	-1.684	2.388	12.467	<0.0005	<0.05
ABTB1	-0.72	8.623	12.458	<0.0005	<0.05
LMO2	0.518	6.114	12.458	<0.0005	<0.05
PHB1	0.518	5.304	12.454	<0.0005	<0.05
TMEM150B	0.795	2.126	12.453	<0.0005	<0.05
DDX11L17	-1.422	0.837	12.452	<0.0005	<0.05
AK2	0.377	6.18	12.441	<0.0005	<0.05
ZCCHC2	0.804	6.131	12.437	<0.0005	<0.05
GATAD2B	-0.366	5.865	12.435	<0.0005	<0.05
MARCHF8	-0.943	7.781	12.433	<0.0005	<0.05
SLC14A1	-1.158	3.849	12.427	<0.0005	<0.05
SGK1	-0.57	6.464	12.426	<0.0005	<0.05
ENSG00000288796	0.925	6.434	12.423	<0.0005	<0.05
GPR42	1.384	0.011	12.417	<0.0005	<0.05
GMCL1	-0.41	5.147	12.414	<0.0005	<0.05

ALYREF	0.579	4.79	12.407	<0.0005	<0.05
SUCLA2	0.469	3.852	12.404	<0.0005	<0.05
CRIP2	-1.15	2.353	12.401	<0.0005	<0.05
ALG2	0.456	3.973	12.386	<0.0005	<0.05
CASS4	-0.665	6.056	12.384	<0.0005	<0.05
COBLL1	0.939	3.336	12.376	<0.0005	<0.05
AKTIP	-0.427	4.585	12.363	<0.0005	<0.05
ERCC6L	1.268	0.114	12.361	<0.0005	<0.05
PAGE2B	-1.52	2.32	12.344	<0.0005	<0.05
ZNF516	-0.639	5.97	12.341	<0.0005	<0.05
LINC02035	-0.638	3.232	12.333	<0.0005	<0.05
SPACA6	-1.135	0.914	12.332	<0.0005	<0.05
METTL13	0.363	4.423	12.33	<0.0005	<0.05
GNPDA1	0.431	4.218	12.329	<0.0005	<0.05
PMVK	0.611	3.766	12.322	<0.0005	<0.05
TMPO	0.417	6.354	12.317	<0.0005	<0.05
NDUFAF1	0.555	3.073	12.309	<0.0005	<0.05
LINC00570	-1.255	2.528	12.306	<0.0005	<0.05
SSR4	0.611	6.25	12.295	<0.0005	<0.05
GZMB	0.999	7.033	12.294	<0.0005	<0.05
ENSG00000267082	-1.345	1.061	12.288	<0.0005	<0.05
CNPY2-AS1	0.598	3.364	12.283	<0.0005	<0.05
IGF2BP3	0.991	1.662	12.282	<0.0005	<0.05
NOL4L	-0.458	5.382	12.277	<0.0005	<0.05
RUVBL2	0.525	4.399	12.267	<0.0005	<0.05
COPS4	0.438	4.513	12.263	<0.0005	<0.05
SDC3	1.17	2.058	12.26	<0.0005	<0.05
CCDC153	-0.952	0.719	12.258	<0.0005	<0.05
IER3IP1	0.496	4.178	12.229	<0.0005	<0.05
PRMT5	0.445	4.53	12.229	<0.0005	<0.05

MTCH2	0.497	4.478	12.226	<0.0005	<0.05
SRRD	-0.771	4.089	12.214	<0.0005	<0.05
LSM7	0.565	4.077	12.203	<0.0005	<0.05
STAB1	0.652	5.571	12.184	<0.0005	<0.05
MRPL16	0.437	3.92	12.183	<0.0005	<0.05
PRDX1	0.555	6.019	12.165	<0.0005	<0.05
ATP1B3	0.486	5.312	12.159	<0.0005	<0.05
XPC	-0.385	5.805	12.158	<0.0005	<0.05
MRPL36	0.579	3.079	12.153	<0.0005	<0.05
MRPL51	0.561	4.864	12.15	<0.0005	<0.05
DRG1	0.444	4.406	12.13	<0.0005	<0.05
TLE3	-0.616	8.011	12.12	<0.0005	<0.05
OAF	0.642	3.649	12.12	<0.0005	<0.05
LSM2	0.545	3.599	12.114	<0.0005	<0.05
SARDH	-0.888	0.658	12.107	<0.0005	<0.05
RCN3	-1.094	3.245	12.099	<0.0005	<0.05
MIR4435-2HG	0.554	5.04	12.089	<0.005	<0.05
ENSG00000285417	0.775	2.931	12.088	<0.005	<0.05
ENSG00000260855	-1.545	-0.365	12.082	<0.005	<0.05
LRR1	0.651	2.614	12.079	<0.005	<0.05
GIMAP1-GIMAP5	-1.022	4.354	12.075	<0.005	<0.05
PARP9	0.687	7.53	12.075	<0.005	<0.05
NUS1	0.472	4.927	12.072	<0.005	<0.05
PDZD11	0.517	3.204	12.069	<0.005	<0.05
ENSG00000289223	-0.883	-0.341	12.067	<0.005	<0.05
RGL1	0.693	2.299	12.067	<0.005	<0.05
PTMS	-0.622	4.817	12.057	<0.005	<0.05
NDUFB7	0.632	4.687	12.057	<0.005	<0.05
IGHG4	1.222	4.11	12.056	<0.005	<0.05
SP140	0.458	5.763	12.049	<0.005	<0.05

XPO5	0.385	4.332	12.049	<0.005	<0.05
VSIG2	-0.944	3.108	12.022	<0.005	<0.05
RHAG	-1.617	1.987	12.013	<0.005	<0.05
ENSG00000238035	-0.907	4.973	12.006	<0.005	<0.05
KIFAP3	0.541	4.121	12.005	<0.005	<0.05
RHD	-1.445	1.68	11.999	<0.005	<0.05
GJD3	0.867	0.016	11.996	<0.005	<0.05
USP12	-0.64	5.149	11.958	<0.005	<0.05
ZXDC	-0.426	5.387	11.958	<0.005	<0.05
ENSG00000289273	-2.899	4.625	11.956	<0.005	<0.05
MMP25-AS1	-1.07	6.211	11.955	<0.005	<0.05
NUP54	0.373	4.122	11.954	<0.005	<0.05
CLEC9A	-1.08	1.641	11.947	<0.005	<0.05
PRMT1	0.527	5.109	11.932	<0.005	<0.05
DPAGT1	0.518	3.699	11.924	<0.005	<0.05
PGM3	0.689	3.171	11.909	<0.005	<0.05
PARP10	0.533	6.179	11.909	<0.005	<0.05
MARK3	-0.483	6.486	11.904	<0.005	<0.05
PTOV1	-0.473	5.306	11.902	<0.005	<0.05
ENSG00000237094	-1.144	0.497	11.893	<0.005	<0.05
TANGO2	-0.513	5.989	11.885	<0.005	<0.05
EXOC3L1	1.28	0.581	11.878	<0.005	<0.05
ENSG00000291221	0.989	4.298	11.869	<0.005	<0.05
FAM117A	-0.544	6.467	11.862	<0.005	<0.05
IL27	1.449	-0.461	11.86	<0.005	<0.05
TMEM97	0.865	1.541	11.853	<0.005	<0.05
RAP1BL	0.574	2.011	11.845	<0.005	<0.05
MT-TG	-1	5.388	11.843	<0.005	<0.05
BATF2	1.281	3.94	11.831	<0.005	<0.05
BOLA2-SMG1P6	0.595	3.029	11.831	<0.005	<0.05

SLC39A7	0.41	5.264	11.831	<0.005	<0.05
HERC5	1.259	6.134	11.82	<0.005	<0.05
DIRAS1	-1.04	-0.291	11.808	<0.005	<0.05
MRPL42	0.45	4.194	11.804	<0.005	<0.05
FKBP8	-0.999	11.532	11.802	<0.005	<0.05
CTU2	0.566	2.337	11.791	<0.005	<0.05
AAGAB	0.378	4.838	11.791	<0.005	<0.05
TAL1	-0.774	4.988	11.788	<0.005	<0.05
SCFD1	0.427	5.004	11.779	<0.005	<0.05
TBL1X	-0.63	7.011	11.775	<0.005	<0.05
KPTN	0.705	1.451	11.771	<0.005	<0.05
ENSG00000280206	-0.523	4.799	11.761	<0.005	<0.05
PPP5C	0.504	4.343	11.758	<0.005	<0.05
SLC6A8	-1.245	4.85	11.744	<0.005	<0.05
FAM225B	1.019	0.588	11.735	<0.005	<0.05
PDK3	-0.529	6.052	11.733	<0.005	<0.05
WWC3	-0.522	6.37	11.719	<0.005	<0.05
ARMC6	0.441	3.705	11.717	<0.005	<0.05
PFDN6	0.461	3.807	11.71	<0.005	<0.05
TEFM	0.568	2.07	11.696	<0.005	<0.05
MAGED1	0.62	4.063	11.689	<0.005	<0.05
HBS1L	0.404	4.655	11.676	<0.005	<0.05
FANCC	0.608	1.593	11.675	<0.005	<0.05
USO1	0.392	5.826	11.675	<0.005	<0.05
RFC5	0.589	2.895	11.652	<0.005	<0.05
NEU1	0.429	5.049	11.652	<0.005	<0.05
TENT5A	0.447	5.338	11.651	<0.005	<0.05
DCUN1D5	0.478	3.278	11.65	<0.005	<0.05
LINC00963	-0.459	5.052	11.615	<0.005	<0.05
C19orf48P	0.631	2.856	11.612	<0.005	<0.05

MAGED2	0.364	5.317	11.611	<0.005	<0.05
TRIM14	0.44	6.093	11.603	<0.005	<0.05
ACAT2	0.524	3.482	11.603	<0.005	<0.05
ENSG00000244733	-0.838	-0.93	11.594	<0.005	<0.05
C16orf54	-0.522	7.648	11.59	<0.005	<0.05
LAMP3	1.276	2.195	11.59	<0.005	<0.05
MYADM	-0.75	8.547	11.588	<0.005	<0.05
POGLUT2	1.183	-0.558	11.585	<0.005	<0.05
UBBP4	-1.011	3.152	11.58	<0.005	<0.05
MYO1B	-1.142	-0.351	11.58	<0.005	<0.05
TRIAP1	0.527	3.266	11.579	<0.005	<0.05
TRAPPC2L	0.481	3.876	11.576	<0.005	<0.05
FUZ	-0.531	3.122	11.564	<0.005	<0.05
ENSG00000260563	-0.988	0.83	11.561	<0.005	<0.05
MAP2K7	-0.385	5.558	11.548	<0.005	<0.05
CENPP	0.847	2.119	11.541	<0.005	<0.05
MIR4697	-1.173	-0.586	11.541	<0.005	<0.05
CNN2P9	-0.993	-0.82	11.541	<0.005	<0.05
FRMD4A	-0.919	2.435	11.535	<0.005	<0.05
CD1C	-0.812	3.069	11.533	<0.005	<0.05
TRUB2	0.45	3.47	11.533	<0.005	<0.05
CPPED1	-0.717	8.74	11.52	<0.005	<0.05
ARHGAP23	1.464	-0.169	11.517	<0.005	<0.05
CCNI	-0.602	8.564	11.51	<0.005	<0.05
NABP2	0.507	3.661	11.504	<0.005	<0.05
CACNA1I	-0.833	3.242	11.498	<0.005	<0.05
TBC1D22A-DT	0.703	0.044	11.497	<0.005	<0.05
RAB6B	-1.02	2.156	11.496	<0.005	<0.05
DCAF6	-0.473	5.538	11.483	<0.005	<0.05
PARP12	0.693	5.479	11.466	<0.005	<0.05

ZNF552	-0.469	3.489	11.445	<0.005	<0.05
PACS1	-0.553	7.594	11.443	<0.005	<0.05
C4orf33	0.649	3.153	11.438	<0.005	<0.05
UBE2N	0.401	5.669	11.435	<0.005	<0.05
PEG13	-0.74	1.032	11.431	<0.005	<0.05
ENSG00000279392	0.655	0.878	11.427	<0.005	<0.05
KLC3	-1.644	2.435	11.403	<0.005	<0.05
ZNF652	-0.388	6.573	11.383	<0.005	<0.05
DBNDD1	-0.892	0.816	11.382	<0.005	<0.05
MOV10	0.584	4.892	11.375	<0.005	<0.05
TXNDC15	0.409	4.852	11.37	<0.005	<0.05
RIOK3	-1.072	6.815	11.355	<0.005	<0.05
PITHD1	-0.558	5.114	11.355	<0.005	<0.05
DNAJC1	0.471	4.562	11.352	<0.005	<0.05
MSRA	-0.604	3.733	11.345	<0.005	<0.05
KLHDC7B-DT	0.988	2.732	11.342	<0.005	<0.05
TUBB	0.568	7.93	11.339	<0.005	<0.05
SPCS1	0.465	5.632	11.334	<0.005	<0.05
BAG6	-0.517	7.838	11.332	<0.005	<0.05
PPP2R5B	-0.549	4.021	11.331	<0.005	<0.05
NLRP7	0.913	0.725	11.33	<0.005	<0.05
COX6A1P2	0.753	1.866	11.328	<0.005	<0.05
ZC3H15	0.343	5.673	11.326	<0.005	<0.05
ATRIP	0.478	3.933	11.323	<0.005	<0.05
FAM225A	1.032	0.888	11.319	<0.005	<0.05
EBNA1BP2	0.564	3.864	11.313	<0.005	<0.05
PCCB	0.442	3.534	11.309	<0.005	<0.05
IGF2R	-0.714	9.749	11.294	<0.005	<0.05
RTCA-AS1	-0.79	2.302	11.288	<0.005	<0.05
MR1	0.377	5.901	11.288	<0.005	<0.05

YARS1	0.413	5.302	11.286	<0.005	<0.05
ENOPH1	0.381	3.983	11.281	<0.005	<0.05
TFEC	0.634	4.618	11.278	<0.005	<0.05
SEC23B	0.389	5.682	11.273	<0.005	<0.05
ISM1	-1.093	0.399	11.271	<0.005	<0.05
NUP205	0.446	5.141	11.268	<0.005	<0.05
GDE1	-0.534	6.306	11.265	<0.005	<0.05
ENSG00000273893	-0.756	-0.274	11.257	<0.005	<0.05
ABCF2	0.457	4.618	11.248	<0.005	<0.05
RAB39A	0.923	1.635	11.246	<0.005	<0.05
ENSG00000232499	0.813	-0.606	11.23	<0.005	<0.05
AMACR	-0.66	3.179	11.218	<0.005	<0.05
POLR1H	0.438	3.753	11.21	<0.005	<0.05
MBD6	-0.598	6.55	11.209	<0.005	<0.05
GNPAT	0.339	4.764	11.205	<0.005	<0.05
VAMP5	0.743	4.259	11.201	<0.005	<0.05
DDX11L16	-1.101	2.663	11.197	<0.005	<0.05
CMC2	0.6	3.455	11.195	<0.005	<0.05
SCAP	-0.399	5.99	11.194	<0.005	<0.05
HSPBP1	0.557	3.19	11.194	<0.005	<0.05
STARD3NL	0.392	4.792	11.188	<0.005	<0.05
FAM104A	-0.547	6.248	11.178	<0.005	<0.05
AIMP2	0.617	2.356	11.177	<0.005	<0.05
ATAD5	0.788	1.554	11.175	<0.005	<0.05
RAB11FIP1	-0.596	8.557	11.167	<0.005	<0.05
GNL3	0.511	4.813	11.166	<0.005	<0.05
CACUL1	-0.385	6.09	11.165	<0.005	<0.05
ENSG00000185839	0.642	0.622	11.155	<0.005	<0.05
IL1R1	-0.733	3.354	11.152	<0.005	<0.05
C16orf87	0.534	2.493	11.151	<0.005	<0.05

ZNF706	0.421	4.612	11.143	<0.005	<0.05
XRCC4	0.486	3.422	11.137	<0.005	<0.05
MRPL3	0.417	5.045	11.136	<0.005	<0.05
PTPMT1	0.509	4.093	11.125	<0.005	<0.05
ERN1	-0.558	6.405	11.123	<0.005	<0.05
WASF2	-0.498	8.123	11.102	<0.005	<0.05
NDUFA12	0.487	4.617	11.1	<0.005	<0.05
ENSG00000230615	-1.105	3.01	11.097	<0.005	<0.05
ACAT1	1.061	3.63	11.079	<0.005	<0.05
RAB21	-0.331	6.036	11.079	<0.005	<0.05
OAZ1	-0.661	10.454	11.079	<0.005	<0.05
GLRX	0.547	6.754	11.077	<0.005	<0.05
TMEM63B	-0.592	4.136	11.076	<0.005	<0.05
TSHZ2	-0.765	2.334	11.064	<0.005	<0.05
FANCG	0.654	2.472	11.061	<0.005	<0.05
ALOX5	-0.652	7.847	11.057	<0.005	<0.05
SNRPC	0.492	4.714	11.049	<0.005	<0.05
ANAPC11	0.495	3.851	11.043	<0.005	<0.05
DNAJC6	-1.193	1.245	11.029	<0.005	<0.05
UBA52	-0.728	10.83	11.026	<0.005	<0.05
DCUN1D1	-0.509	5.491	11.02	<0.005	<0.05
NOP56	0.388	5.685	11.004	<0.005	<0.05
ENSG00000260917	-0.732	1.853	11.003	<0.005	<0.05
RIGI	0.742	6.828	10.997	<0.005	<0.05
CD274	0.93	4.354	10.996	<0.005	<0.05
MED24	0.414	4.721	10.991	<0.005	<0.05
AZI2	0.477	4.198	10.991	<0.005	<0.05
WDFY1	0.391	5.85	10.98	<0.005	<0.05
GCSH	0.981	0.456	10.979	<0.005	<0.05
POLR2K	0.442	4.187	10.97	<0.005	<0.05

POP1	0.607	2.213	10.969	<0.005	<0.05
ZW10	0.466	3.333	10.955	<0.005	<0.05
GSTCD	0.693	1.301	10.953	<0.005	<0.05
COL4A4	1.201	0.911	10.953	<0.005	<0.05
MRC2	-1.212	2.896	10.944	<0.005	<0.05
FAM168B	-0.363	6.116	10.94	<0.005	<0.05
INPP5K	-0.377	5.844	10.937	<0.005	<0.05
FAM3C	0.537	4.019	10.934	<0.005	<0.05
YPEL3-DT	-0.771	3.662	10.91	<0.005	<0.05
RGS17	-1.045	-0.853	10.901	<0.005	<0.05
SCO2	1.026	0.874	10.9	<0.005	<0.05
CNTNAP3C	-1.579	1.9	10.898	<0.005	<0.05
PTS	0.644	1.426	10.875	<0.005	<0.05
SNORD3A	2.047	1.757	10.873	<0.005	<0.05
HNRNPA3P3	0.564	0.916	10.86	<0.005	<0.05
PDHB	0.383	5.145	10.858	<0.005	<0.05
PSMB2	0.447	5.629	10.857	<0.005	<0.05
AGPS	0.337	5.176	10.853	<0.005	<0.05
RPF1	0.383	4.751	10.852	<0.005	<0.05
EXOSC10	0.363	5.133	10.851	<0.005	<0.05
PDCD1LG2	1.203	1.048	10.84	<0.005	<0.05
ATAD3A	0.553	3.119	10.839	<0.005	<0.05
ENSG00000287958	0.788	2.578	10.823	<0.005	<0.05
NFIC	-0.419	5.185	10.822	<0.005	<0.05
IGF2	-1.754	-0.59	10.818	<0.005	<0.05
CCDC170	-0.735	2.221	10.815	<0.005	<0.05
DNAAF11	-1.163	1.277	10.813	<0.005	<0.05
FAM136A	0.478	3.901	10.812	<0.005	<0.05
NFAM1	-0.601	8.656	10.811	<0.005	<0.05
NECTIN2	1.148	2.595	10.81	<0.005	<0.05

FPR3	1.305	2.193	10.803	<0.005	<0.05
CD300E	0.761	7.09	10.798	<0.005	<0.05
STXBP1	-0.818	0.811	10.795	<0.005	<0.05
SLC20A1	0.42	5.89	10.787	<0.005	<0.05
MIR29B2CHG	-1.099	0.283	10.785	<0.005	<0.05
PPP3CC	0.411	4.434	10.779	<0.005	<0.05
CCR2	0.607	6.961	10.776	<0.005	<0.05
SHMT2	0.495	5.056	10.774	<0.005	<0.05
OGFRL1	-0.565	8.276	10.773	<0.005	<0.05
DEF8	-0.482	6.508	10.772	<0.005	<0.05
GOT1	0.603	2.769	10.765	<0.005	<0.05
H2BC19P	-1.1	1.647	10.748	<0.005	<0.05
KIF27	-0.824	3.316	10.744	<0.005	<0.05
NOMO2	0.465	6.19	10.732	<0.005	<0.05
AVEN	0.61	2.121	10.727	<0.005	<0.05
WFS1	1.024	1.566	10.726	<0.005	<0.05
ENSG00000291215	-0.833	5.111	10.724	<0.005	<0.05
PLXNA4	-0.797	1.475	10.719	<0.005	<0.05
TLCD4	-1.136	1.16	10.714	<0.005	<0.05
PHF20	-0.331	6.124	10.712	<0.005	<0.05
ICMT	0.368	4.823	10.71	<0.005	<0.05
IFITM3P1	0.908	0.592	10.688	<0.005	<0.05
MIR3667HG	0.615	2.259	10.684	<0.005	<0.05
NUTM2A-AS1	-0.43	4.805	10.676	<0.005	<0.05
RTN1	-0.76	4.166	10.676	<0.005	<0.05
ANTXR2	-0.547	7.232	10.67	<0.005	<0.05
MRPL52	0.529	3.805	10.662	<0.005	<0.05
WASHC5	0.3	5.116	10.652	<0.005	<0.05
SRP9P1	0.47	2.181	10.646	<0.005	<0.05
DNMT3A	-0.42	5.132	10.642	<0.005	<0.05

PTGS2	-0.671	5.176	10.636	<0.005	<0.05
FBXO8	0.379	3.528	10.632	<0.005	<0.05
VAMP2	-0.378	6.432	10.628	<0.005	<0.05
HSPD1P1	0.684	1.097	10.623	<0.005	<0.05
LINC02340	-0.903	1.97	10.622	<0.005	<0.05
HMGN2P41	0.453	4.954	10.622	<0.005	<0.05
SIAH1	-0.423	3.854	10.621	<0.005	<0.05
HMGA1	0.542	6.187	10.62	<0.005	<0.05
TMEM45B	-0.844	2.082	10.597	<0.005	<0.05
PEPD	0.417	4.797	10.596	<0.005	<0.05
MIR3945HG	1.049	3.736	10.589	<0.005	<0.05
FILIP1L	-1.035	-0.147	10.589	<0.005	<0.05
PDHX	0.478	3.301	10.584	<0.005	<0.05
OXSM	0.557	1.758	10.583	<0.005	<0.05
TXNL4B	0.448	3.959	10.581	<0.005	<0.05
APOO	0.65	1.457	10.578	<0.005	<0.05
NEAT1	-0.975	7.057	10.575	<0.005	<0.05
MYOM2	1.973	4.478	10.569	<0.005	<0.05
SKA2	0.482	3.894	10.56	<0.005	<0.05
SLC22A23	-0.548	2.79	10.545	<0.005	<0.05
TMCC1	-0.668	5.929	10.54	<0.005	<0.05
MRPL37	0.509	4.393	10.537	<0.005	<0.05
EI24	0.398	4.355	10.533	<0.005	<0.05
SLC44A2	-0.538	8.51	10.528	<0.005	<0.05
TIMM44	0.494	3.494	10.527	<0.005	<0.05
MLEC	0.417	6.425	10.525	<0.005	<0.05
ZNF117	-0.675	5.464	10.521	<0.005	<0.05
SAMD3	0.526	5.415	10.497	<0.005	<0.05
SLC10A7	0.495	2.767	10.484	<0.005	<0.05
SPTB	-1.416	4.898	10.479	<0.005	<0.05

LRRC41	0.348	4.416	10.468	<0.005	<0.05
CA5B	-0.409	4.586	10.464	<0.005	<0.05
COX16	0.564	3.798	10.459	<0.005	<0.05
AGK	0.455	4.233	10.458	<0.005	<0.05
PAFAH1B2	-0.346	5.924	10.458	<0.005	<0.05
SLC39A14	0.655	2.55	10.452	<0.005	<0.05
SPTA1	-1.168	2.417	10.45	<0.005	<0.05
NDUFS8	0.482	4.058	10.449	<0.005	<0.05
PANX2	-0.781	4.825	10.444	<0.005	<0.05
VRK1	0.476	3.805	10.44	<0.005	<0.05
ZNF217	-0.487	7.441	10.424	<0.005	<0.05
SLC49A4	-0.508	3.344	10.423	<0.005	<0.05
CNPY2	0.593	3.497	10.422	<0.005	<0.05
MED20	0.572	3.15	10.419	<0.005	<0.05
ENSG00000284526	-0.846	4.66	10.408	<0.005	<0.05
TRBV28	-1.114	4.327	10.403	<0.005	<0.05
EXOSC3	0.378	3.861	10.399	<0.005	<0.05
CBR1	0.53	4.012	10.399	<0.005	<0.05
PACC1	0.471	2.58	10.391	<0.005	<0.05
SLC45A4	-0.648	6.234	10.384	<0.005	<0.05
UBR1	0.443	4.993	10.383	<0.005	<0.05
NMD3	0.36	4.293	10.383	<0.005	<0.05
GPR155	-0.417	4.953	10.377	<0.005	<0.05
NUP42	0.463	3.058	10.375	<0.005	<0.05
PECAM1	-0.498	9.459	10.369	<0.005	<0.05
MIR4432HG	-0.828	0.584	10.367	<0.005	<0.05
CCS	-0.444	3.644	10.364	<0.005	<0.05
CTSE	-2.014	1.077	10.362	<0.005	<0.05
MT-TR	-0.831	4.866	10.348	<0.005	<0.05
PLRG1	0.323	4.946	10.346	<0.005	<0.05

PDE3B	-0.453	5.936	10.344	<0.005	<0.05
C19orf12	0.437	3.913	10.334	<0.005	<0.05
PGAM5	0.418	3.788	10.329	<0.005	<0.05
CPSF7	-0.408	6.17	10.329	<0.005	<0.05
CCNY	-0.481	7.029	10.32	<0.005	<0.05
GNPTAB	0.406	6.185	10.316	<0.005	<0.05
TYMP	0.586	8.322	10.315	<0.005	<0.05
KCNQ5	0.694	1.354	10.313	<0.005	<0.05
HIP1	-0.656	6.283	10.308	<0.005	<0.05
SNRPEP4	0.868	-0.677	10.304	<0.005	<0.05
MAGT1	0.38	5.869	10.292	<0.005	<0.05
ITGB1BP1	0.386	4.822	10.286	<0.005	<0.05
SUV39H1	0.472	2.978	10.283	<0.005	<0.05
ATRAID	0.434	5.21	10.28	<0.005	<0.05
POLD1	0.5	3.397	10.276	<0.005	<0.05
SHISA7	-1.241	-0.103	10.268	<0.005	<0.05
GOSR2	0.375	4.698	10.264	<0.005	<0.05
POP5	0.499	3.097	10.256	<0.005	<0.05
ENSG00000270175	0.749	-0.347	10.255	<0.005	<0.05
GNAO1	-0.655	2.317	10.253	<0.005	<0.05
YWHAZP10	0.565	1.476	10.253	<0.005	<0.05
ZFAND2A	0.484	2.834	10.25	<0.005	<0.05
TMED3	0.448	4.388	10.243	<0.005	<0.05
PI3	-1.32	5.685	10.234	<0.005	<0.05
MHENCR	-0.709	3.158	10.233	<0.005	<0.05
SEPTIN5	-1.048	4.224	10.232	<0.005	<0.05
ABHD2	-0.57	8.224	10.225	<0.005	<0.05
ENSG00000279838	-0.831	-0.167	10.223	<0.005	<0.05
APOBEC3H	0.833	0.95	10.219	<0.005	<0.05
ENSG00000269044	-0.627	2.561	10.215	<0.005	<0.05

RPL7AP70	0.781	-0.403	10.207	<0.005	<0.05
ALCAM	-0.408	4.471	10.206	<0.005	<0.05
GLE1	0.297	5.567	10.195	<0.005	<0.05
ENSG00000286022	-1.297	2.623	10.195	<0.005	<0.05
LIMD1	0.421	5.087	10.192	<0.005	<0.05
PLK3	-0.479	3.908	10.186	<0.005	<0.05
EZH1	-0.403	5.627	10.182	<0.005	<0.05
MRPL17	0.518	3.405	10.176	<0.005	<0.05
PRKY	1.818	1.669	10.175	<0.005	<0.05
ZRSR2P1	-1.186	1.1	10.171	<0.005	<0.05
MED27	0.483	2.985	10.164	<0.005	<0.05
DDIAS	0.615	2.325	10.163	<0.005	<0.05
FDX1	0.473	3.658	10.162	<0.005	<0.05
TBC1D14	-0.556	7.057	10.162	<0.005	<0.05
MRPL34	0.422	3.837	10.159	<0.005	<0.05
SRP54	0.396	5.37	10.156	<0.005	<0.05
LARS2	0.475	3.517	10.156	<0.005	<0.05
CARMIL3	1.29	1.326	10.146	<0.005	<0.05
TMEM87A	0.31	5.147	10.133	<0.005	<0.05
EXOSC1	0.446	3.922	10.129	<0.005	<0.05
SLC17A9	1.027	2.222	10.127	<0.005	<0.05
GGACT	0.602	1.856	10.117	<0.005	<0.05
ENSG00000289469	0.874	0.118	10.113	<0.005	<0.05
JSRP1	1.071	-0.65	10.107	<0.005	<0.05
ASCC3	0.407	5.19	10.103	<0.005	<0.05
TVP23B	0.379	4.238	10.102	<0.005	<0.05
PROSER3	-0.427	3.225	10.092	<0.005	<0.05
LUNAR1	-1.248	-0.465	10.087	<0.005	<0.05
ACKR1	-1.718	0.551	10.08	<0.005	<0.05
ALDOC	-0.447	3.929	10.072	<0.005	<0.05

PLIN5	-0.882	2.916	10.065	<0.005	<0.05
THEM5	-0.866	3.447	10.063	<0.005	<0.05
ITGAX	-0.734	8.243	10.062	<0.005	<0.05
MAEA	-0.341	6.004	10.056	<0.005	<0.05
TUBA1B	0.543	7.817	10.05	<0.005	<0.05
AKIP1	0.586	2.278	10.05	<0.005	<0.05
EME2	-0.417	5.575	10.048	<0.005	<0.05
LIN52	0.497	2.669	10.04	<0.005	<0.05
LRRCC1	0.629	2.063	10.02	<0.005	<0.05
ST7L	0.53	2.448	10.017	<0.005	<0.05
UQCR10	0.48	5.088	9.999	<0.005	<0.05
RFX5	0.341	5.357	9.99	<0.005	<0.05
CRBN	0.364	4.874	9.977	<0.005	<0.05
DMAC1	0.437	3.764	9.972	<0.005	<0.05
RUBCNL	-0.608	5.574	9.958	<0.005	<0.05
JARID2	-0.391	6.524	9.956	<0.005	<0.05
INTS4P1	0.54	-0.252	9.953	<0.005	<0.05
COQ8A	-0.453	5.182	9.949	<0.005	<0.05
FASLG	0.586	3.681	9.947	<0.005	<0.05
NCOA7	0.48	4.904	9.947	<0.005	<0.05
ZKSCAN5	0.369	3.73	9.944	<0.005	<0.05
PLEKHJ1	0.358	4.334	9.943	<0.005	<0.05
TNFSF10	0.634	8.29	9.934	<0.005	<0.05
POMC	0.927	0.922	9.928	<0.005	<0.05
DYNC1LI1	-0.378	5.908	9.925	<0.005	<0.05
PRKAR1A	-0.457	8.4	9.925	<0.005	<0.05
TBC1D3L	-1.058	1.487	9.917	<0.005	<0.05
CREB3	0.439	3.801	9.912	<0.005	<0.05
NUP35	0.525	1.877	9.911	<0.005	<0.05
NOC3L	0.503	3.313	9.91	<0.005	<0.05

FAM3C2P	0.727	0.623	9.906	<0.005	<0.05
PI16	-0.805	2.223	9.897	<0.005	<0.05
TRAIP	1.142	-0.297	9.896	<0.005	<0.05
XPNPEP1	0.403	5.195	9.886	<0.005	<0.05
BTN3A2	0.517	7.795	9.885	<0.005	<0.05
EXTL3	-0.506	6.067	9.884	<0.005	<0.05
FKBP3	0.423	4.24	9.878	<0.005	<0.05
MYO7A	0.917	1.165	9.877	<0.005	<0.05
PADI2	-0.79	7.371	9.875	<0.005	<0.05
DDAH2	0.482	4.822	9.874	<0.005	<0.05
PML	0.596	6.12	9.868	<0.005	<0.05
SAPCD2	1.112	1.86	9.868	<0.005	<0.05
ENSG00000287779	-1.024	-0.575	9.866	<0.005	<0.05
TLR3	0.996	1.448	9.86	<0.005	<0.05
ENSG00000289564	-1.015	0.28	9.857	<0.005	<0.05
PGGHG	-0.814	7.299	9.857	<0.005	<0.05
ZBTB8OS	0.471	4.064	9.855	<0.005	<0.05
NMI	0.589	6.852	9.854	<0.005	<0.05
GYPA	-1.311	1.73	9.851	<0.005	<0.05
HSPA5	0.6	8.147	9.851	<0.005	<0.05
GLUD2	0.635	0.511	9.851	<0.005	<0.05
EIF2AK2	0.776	6.853	9.849	<0.005	<0.05
NACC2	-0.404	6.1	9.846	<0.005	<0.05
DBF4	0.448	3.369	9.833	<0.005	<0.05
NPEPPS	-0.406	6.575	9.824	<0.005	<0.05
MXD4	-0.415	5.865	9.823	<0.005	<0.05
ENSG00000274425	-0.94	7.869	9.814	<0.005	<0.05
C1QBP	0.464	5.264	9.813	<0.005	<0.05
EMC7	0.421	4.782	9.813	<0.005	<0.05
LEO1	0.393	3.927	9.812	<0.005	<0.05

SF3B5	0.478	5.182	9.81	<0.005	<0.05
FBXL17	-0.333	4.045	9.803	<0.005	<0.05
GNAQ	-0.54	7.139	9.796	<0.005	<0.05
MICOS10	0.415	4.336	9.793	<0.005	<0.05
SORL1	-0.79	10.547	9.79	<0.005	<0.05
UQCRH	0.539	5.791	9.783	<0.005	<0.05
SEPHS1	0.458	4.249	9.775	<0.005	<0.05
UQCRFS1	0.446	5.131	9.774	<0.005	<0.05
INPP5A	-0.436	4.168	9.763	<0.005	<0.05
NUDCD1	0.516	2.939	9.758	<0.005	<0.05
LPCAT2	-0.6	6.676	9.752	<0.005	<0.05
SHISA4	-1.322	3.692	9.752	<0.005	<0.05
DOCK5	-0.664	7.107	9.752	<0.005	<0.05
YWHAZP3	0.5	2.103	9.745	<0.005	<0.05
SPRED1	-0.68	1.892	9.742	<0.005	<0.05
CRADD	0.484	2.579	9.733	<0.005	<0.05
TRIM26	0.328	5.84	9.73	<0.005	<0.05
LINC01504	0.693	1.703	9.723	<0.005	<0.05
ZNF496	0.524	3.33	9.723	<0.005	<0.05
KATNBL1	-0.575	5.086	9.719	<0.005	<0.05
ORMDL2	0.445	4.239	9.71	<0.005	<0.05
CXorf38	-0.318	5.637	9.706	<0.005	<0.05
PWP1	0.363	4.588	9.704	<0.005	<0.05
NME2	0.536	6.171	9.704	<0.005	<0.05
RAB3D	-0.565	7.631	9.692	<0.005	<0.05
PKP4	-0.475	4.037	9.683	<0.005	<0.05
GPC1	0.918	-0.354	9.677	<0.005	<0.05
COA3	0.507	3.465	9.672	<0.005	<0.05
MPHOSPH6	0.443	2.832	9.665	<0.005	<0.05
SNHG25	0.767	-0.267	9.664	<0.005	<0.05

SIK1	0.727	1.774	9.662	<0.005	<0.05
GPR162	-0.799	2.883	9.657	<0.005	<0.05
RAD54B	1.269	-0.097	9.656	<0.005	<0.05
MTLN	0.594	2.472	9.652	<0.005	<0.05
LINC02458	-0.955	0.488	9.64	<0.005	<0.05
PPIA	0.506	8.42	9.632	<0.005	<0.05
ENSG00000283782	1.862	3.3	9.628	<0.005	<0.05
ULK1	-0.448	6.294	9.627	<0.005	<0.05
MRPS24	0.559	3.988	9.612	<0.005	<0.05
HMGN4	0.316	6.267	9.609	<0.005	<0.05
FLJ40194	-0.737	-0.451	9.609	<0.005	<0.05
COPS9	0.474	3.959	9.603	<0.005	<0.05
POP4	0.408	4.561	9.601	<0.005	<0.05
NAGLU	0.455	3.234	9.601	<0.005	<0.05
EPHX1	-0.513	4.037	9.597	<0.005	<0.05
ANG	0.863	1.239	9.594	<0.005	<0.05
CD69	0.526	4.212	9.588	<0.005	<0.05
CBX3	0.315	6.403	9.587	<0.005	<0.05
GYPB	-1.682	2.008	9.577	<0.005	<0.05
GARRE1	-0.484	4.147	9.577	<0.005	<0.05
SIGLEC11	1.167	0.008	9.57	<0.005	<0.05
PIGX	-0.453	5.183	9.57	<0.005	<0.05
COX8A	0.517	5.529	9.567	<0.005	<0.05
MIRLET7BHG	-1.007	0.838	9.564	<0.005	<0.05
NCK2	-0.516	6.26	9.561	<0.005	<0.05
HPS1	-0.705	5.94	9.551	<0.005	<0.05
NDUFC2	0.457	4.941	9.549	<0.005	<0.05
KRT1	-1.678	5.407	9.548	<0.005	<0.05
AEN	0.407	3.828	9.544	<0.005	<0.05
SLC22A17	-0.803	0.619	9.539	<0.005	<0.05

SERBP1P5	0.615	0.845	9.535	<0.005	<0.05
ELP5	0.395	3.9	9.525	<0.005	<0.05
ST3GAL5	0.442	4.102	9.525	<0.005	<0.05
GALE	0.527	2.055	9.524	<0.005	<0.05
MRPL39	0.542	3.014	9.523	<0.005	<0.05
ENSG00000260257	-1.005	1.096	9.513	<0.005	<0.05
CNNM3	-0.352	4.982	9.513	<0.005	<0.05
OAZ2	-0.539	7.52	9.506	<0.005	<0.05
HSPE1P18	-1.191	-0.107	9.506	<0.005	<0.05
CAPN2	0.395	7.505	9.506	<0.005	<0.05
TMEM255A	1.21	0.505	9.505	<0.005	<0.05
DBI	0.478	5.782	9.504	<0.005	<0.05
VPS54	0.35	4.323	9.504	<0.005	<0.05
USP14	0.358	4.905	9.501	<0.005	<0.05
ATF4	0.409	7.009	9.497	<0.005	<0.05
C8orf44-SGK3	-1.708	1.702	9.487	<0.005	<0.05
PAPSS2	-0.597	2.06	9.479	<0.005	<0.05
GNA12	-0.549	5.463	9.479	<0.005	<0.05
EDA	-0.884	0.542	9.477	<0.005	<0.05
COPB2	0.375	6.857	9.47	<0.005	<0.05
NAA20	0.359	4.022	9.465	<0.005	<0.05
GCNT4	-0.521	3.344	9.458	<0.005	<0.05
TBXAS1	-0.494	7.336	9.441	<0.005	<0.05
ENSG00000272468	-0.993	1.397	9.439	<0.005	<0.05
LTBP3	-0.548	4.739	9.437	<0.005	<0.05
NFU1	0.452	3.49	9.434	<0.005	<0.05
USP28	0.42	4.991	9.43	<0.005	<0.05
TNNT1	1.191	2.544	9.427	<0.005	<0.05
POLR2H	0.487	3.056	9.427	<0.005	<0.05
VWCE	-1.373	3.621	9.426	<0.005	<0.05

PIGU	0.514	2.763	9.426	<0.005	<0.05
TPRG1L	-0.447	6.15	9.423	<0.005	<0.05
ENSG00000286555	-0.839	1.992	9.422	<0.005	<0.05
TRIM10	-0.919	2.954	9.421	<0.005	<0.05
RAPGEF2	-0.513	6.149	9.417	<0.005	<0.05
SLC43A2	-0.557	7.52	9.416	<0.005	<0.05
LINC02863	-0.753	4.837	9.415	<0.005	<0.05
PER1	-0.851	4.358	9.405	<0.005	<0.05
LINC01215	-0.494	2.972	9.405	<0.005	<0.05
CCDC138	0.88	0.681	9.405	<0.005	<0.05
ENSG00000279088	-0.575	2.714	9.404	<0.005	<0.05
CREM	0.57	3.142	9.403	<0.005	<0.05
ZDHHC16	0.465	3.564	9.402	<0.005	<0.05
PRADC1	0.608	2.35	9.402	<0.005	<0.05
ENSG00000288473	0.619	3.401	9.401	<0.005	<0.05
LIG1	0.487	3.946	9.397	<0.005	<0.05
CCT7	0.4	6.492	9.396	<0.005	<0.05
SLFN12L	-1.469	3.565	9.395	<0.005	<0.05
C8orf76	0.386	3.2	9.394	<0.005	<0.05
RAB11B	-0.375	6.199	9.392	<0.005	<0.05
CPNE5	0.708	4.399	9.385	<0.005	<0.05
CLEC2B	0.584	7.181	9.383	<0.005	<0.05
C14orf132	-0.941	1.122	9.383	<0.005	<0.05
GTF3C6	0.463	4.194	9.379	<0.005	<0.05
NOP16	0.495	3.027	9.374	<0.005	<0.05
HSPA9	0.361	6.681	9.369	<0.005	<0.05
CCR9	0.932	1.123	9.364	<0.005	<0.05
TMC5	-1.263	0.13	9.359	<0.005	<0.05
NDUFC1	0.457	3.671	9.358	<0.005	<0.05
RRAS	0.509	3.768	9.358	<0.005	<0.05

DAPK2	-0.588	5.043	9.354	<0.005	<0.05
CCDC32	0.401	4.097	9.352	<0.005	<0.05
UTP14A	0.391	4.094	9.338	<0.005	<0.05
ENSG00000293339	0.469	4.172	9.338	<0.005	<0.05
HSPH1	0.397	5.611	9.336	<0.005	<0.05
BFAR	0.3	4.912	9.333	<0.005	<0.05
HAVCR2	0.463	4.701	9.329	<0.005	<0.05
PSMC6	0.318	5.479	9.328	<0.005	<0.05
PCBP3	-0.985	0.118	9.327	<0.005	<0.05
C1GALT1C1	0.397	4.231	9.317	<0.005	<0.05
PLIN4	-0.969	2.777	9.317	<0.005	<0.05
LDHAP4	1.269	0.382	9.317	<0.005	<0.05
GADD45GIP1	0.485	4.538	9.315	<0.005	<0.05
RCAN2	1.157	0.183	9.315	<0.005	<0.05
CD180	0.591	5.275	9.306	<0.005	<0.05
CLK3	-0.371	6.18	9.301	<0.005	<0.05
RPE	0.367	3.939	9.301	<0.005	<0.05
RNASEH2A	0.618	2.573	9.299	<0.005	<0.05
MRPS18A	0.525	3.762	9.296	<0.005	<0.05
ALG3	0.46	3.528	9.294	<0.005	<0.05
SNX25	0.593	2.841	9.293	<0.005	<0.05
PPP1CB	-0.39	7.411	9.288	<0.005	<0.05
TRGJP2	1.134	3.481	9.279	<0.005	<0.05
PEX19	0.279	4.93	9.272	<0.005	<0.05
RUVBL1	0.418	4.246	9.272	<0.005	<0.05
ISCA2	0.377	3.702	9.269	<0.005	<0.05
ARSG	-0.402	4.294	9.264	<0.005	<0.05
CCT4	0.353	6.081	9.256	<0.005	<0.05
H3P6	-0.646	7.801	9.251	<0.005	<0.05
MFSD3	0.588	1.339	9.247	<0.005	<0.05

MARCKSL1	-0.444	5.742	9.235	<0.005	<0.05
CMTM2	-0.739	6.466	9.235	<0.005	<0.05
ENSG00000278330	-0.887	-0.405	9.232	<0.005	<0.05
PALD1	0.873	0.157	9.227	<0.005	<0.05
SLC35C1	0.355	4.384	9.226	<0.005	<0.05
MT-ND4	-0.894	9.784	9.22	<0.005	<0.05
TOMM5	0.488	4.19	9.218	<0.005	<0.05
TPD52	0.68	4.065	9.216	<0.005	<0.05
ATP5PD	0.461	5.636	9.207	<0.005	<0.05
EIF4E	0.327	4.749	9.196	<0.005	<0.05
GTF2I	-0.372	6.718	9.194	<0.005	<0.05
POC5	0.437	2.872	9.194	<0.005	<0.05
PSMA1	0.391	5.786	9.188	<0.005	<0.05
TMEM214	0.428	5.006	9.186	<0.005	<0.05
ENSG00000279738	-1.385	1.544	9.184	<0.005	<0.05
TRBV29-1	-1.169	0.795	9.183	<0.005	<0.05
MNT	-0.326	5.183	9.18	<0.005	<0.05
DONSON	0.662	2.211	9.177	<0.005	<0.05
IFI30	0.545	9.022	9.174	<0.005	<0.05
GLO1	0.369	4.986	9.161	<0.005	<0.05
ENSG00000241489	-0.614	7.368	9.153	<0.005	<0.05
GM2A	0.498	5.987	9.139	<0.005	<0.05
ENSG00000276649	-0.712	3.351	9.138	<0.005	<0.05
FHDC1	-1.057	1.834	9.135	<0.005	<0.05
UVSSA	-0.693	4.201	9.135	<0.005	<0.05
TUBA1C	0.456	6.17	9.124	<0.005	<0.05
CHST11	-0.455	7.453	9.123	<0.005	<0.05
TOMM40	0.439	4.027	9.122	<0.005	<0.05
HSDL2	-0.552	5.952	9.119	<0.005	<0.05
LSM3	0.49	4.713	9.116	<0.005	<0.05

ZNRF2	0.381	3.798	9.116	<0.005	<0.05
WNK1	-0.503	8.339	9.114	<0.005	<0.05
ANKRD36BP2	1.352	1.575	9.113	<0.005	<0.05
ZFYVE26	0.393	4.969	9.111	<0.005	<0.05
ENSG00000266302	-1.209	0.728	9.11	<0.005	<0.05
C1QA	1.343	2.871	9.106	<0.005	<0.05
KREMEN1	-1.051	5.629	9.105	<0.005	<0.05
HCP5	0.392	7.31	9.103	<0.005	<0.05
RAB33A	0.571	1.903	9.103	<0.005	<0.05
ENSG00000282804	-1.109	-0.267	9.101	<0.005	<0.05
ALDH4A1	0.512	1.656	9.1	<0.005	<0.05
APOBEC3C	0.439	6.845	9.098	<0.005	<0.05
TTC9	-0.598	4.622	9.094	<0.005	<0.05
UQCC2	0.547	3.326	9.093	<0.005	<0.05
SKIC8	0.395	4.028	9.093	<0.005	<0.05
XYLT1	-0.401	5.222	9.091	<0.005	<0.05
BAMBI	1.095	0.315	9.088	<0.005	<0.05
TMEM160	0.58	3.059	9.088	<0.005	<0.05
NUTM2B-AS1	-0.434	4.012	9.086	<0.005	<0.05
BZW2	0.449	4.011	9.084	<0.005	<0.05
ASCL2	0.649	3.474	9.081	<0.005	<0.05
OSBPL2	-0.38	6.312	9.078	<0.005	<0.05
ZDHHC4	0.465	3.726	9.074	<0.005	<0.05
SLC7A1	0.427	4.616	9.068	<0.005	<0.05
TRPC1	-0.876	-0.252	9.066	<0.005	<0.05
ZNF33A	-0.387	6.498	9.065	<0.005	<0.05
METAP2	0.366	5.153	9.065	<0.005	<0.05
STAT6	-0.429	8.456	9.064	<0.005	<0.05
ENSG00000280060	-0.789	0.139	9.06	<0.005	<0.05
ENSG00000289700	-0.618	3.005	9.06	<0.005	<0.05

ENSG00000288887	0.838	3.563	9.052	<0.005	<0.05
KDM7A-DT	-0.705	3.533	9.044	<0.005	<0.05
CSTF2	0.439	3.227	9.04	<0.005	<0.05
RCAN3	-0.488	5.826	9.039	<0.005	<0.05
MYH9	-0.502	10.904	9.031	<0.005	<0.05
CYB5R1	-0.384	4.772	9.026	<0.005	<0.05
RGCC	-0.483	4.387	9.023	<0.005	<0.05
MRPL54	0.522	4.167	9.023	<0.005	<0.05
SOX6	-0.954	1.328	9.022	<0.005	<0.05
PXMP2	0.667	0.935	9.021	<0.005	<0.05
MRPS26	0.397	3.509	9.014	<0.005	<0.05
CHCHD2	0.484	6.651	9.013	<0.005	<0.05
UTP11	0.387	3.662	9.013	<0.005	<0.05
ENSG00000276418	0.694	1.66	9.01	<0.005	<0.05
PKN2	-0.418	5.793	9.006	<0.005	<0.05
OTX1	-1.185	1.397	9.006	<0.005	<0.05
FHL1	-0.85	4.136	9.004	<0.005	<0.05
NOMO3	0.428	5.286	9.004	<0.005	<0.05
GTPBP4	0.35	4.692	9.003	<0.005	<0.05
HSP90AA1	0.411	8.863	8.999	<0.005	<0.05
P2RX4	0.41	3.847	8.995	<0.005	<0.05
BTK	0.362	6.334	8.993	<0.005	<0.05
LARP1B	0.616	2.674	8.993	<0.005	<0.05
TIPRL	0.275	4.953	8.988	<0.005	<0.05
NTAQ1	0.599	1.45	8.987	<0.005	<0.05
PRDM8	-0.56	3.385	8.986	<0.005	<0.05
NAA15	0.369	4.924	8.985	<0.005	<0.05
MGRN1	-0.397	6.265	8.976	<0.005	<0.05
ENSG00000288924	-0.41	4.027	8.973	<0.005	<0.05
CDC27	0.314	5.388	8.965	<0.005	<0.05

APBB2	-1.226	0.019	8.958	<0.005	<0.05
COPS2	0.338	5.285	8.957	<0.005	<0.05
ABHD5	-0.551	6.405	8.955	<0.005	<0.05
H1-10	0.424	6.351	8.954	<0.005	<0.05
B3GNTL1	-0.809	3.596	8.954	<0.005	<0.05
ERGIC2	0.382	5.362	8.953	<0.005	<0.05
BLNK	0.663	3.131	8.953	<0.005	<0.05
CPNE2	-0.543	4.665	8.95	<0.005	<0.05
PPTC7	-0.369	6.082	8.942	<0.005	<0.05
MT-CYB	-0.741	11.851	8.941	<0.005	<0.05
ENSG00000285238	-0.679	6.81	8.935	<0.005	<0.05
EHD4	0.362	4.664	8.933	<0.005	<0.05
ENSG00000267940	-1.018	0.016	8.927	<0.005	<0.05
MTURN	-0.668	6.808	8.922	<0.005	<0.05
MSL3P1	0.66	0.354	8.919	<0.005	<0.05
CYBB	0.482	9.05	8.917	<0.005	<0.05
ACSS3	-1.022	0.746	8.915	<0.005	<0.05
TSPAN2	-0.63	5.274	8.909	<0.005	<0.05
AK6	0.502	2.692	8.907	<0.005	<0.05
RPP40	0.631	0.625	8.906	<0.005	<0.05
MIR29C	-1.172	4.562	8.904	<0.005	<0.05
HSPA14	0.459	2.987	8.902	<0.005	<0.05
SNRPE	0.49	4.273	8.901	<0.005	<0.05
CYTH2	-0.291	5.028	8.897	<0.005	<0.05
RPS27P19	0.62	1.879	8.887	<0.005	<0.05
STMP1	-0.612	5.957	8.886	<0.005	<0.05
PSMD10	0.394	4.19	8.881	<0.005	<0.05
CCRL2	0.623	2.304	8.864	<0.005	<0.05
JADE1	-0.32	5.592	8.861	<0.005	<0.05
NDUFB2	0.475	4.548	8.859	<0.005	<0.05

NME7	0.54	1.568	8.857	<0.005	<0.05
GRAMD1C	-0.797	2.389	8.851	<0.005	<0.05
STX17	0.342	4.723	8.845	<0.005	<0.05
ANAPC16	-0.293	6.314	8.841	<0.005	<0.05
BTN3A3	0.494	6.926	8.84	<0.005	<0.05
COPS8	0.355	4.206	8.84	<0.005	<0.05
ERF	-0.794	5.14	8.838	<0.005	<0.05
ENSG00000289039	-1.193	3.663	8.828	<0.005	<0.05
DERL2	0.36	4.878	8.825	<0.005	<0.05
EIF6	0.454	5.413	8.817	<0.005	<0.05
APTX	0.392	3.526	8.815	<0.005	<0.05
PSMB1	0.382	6.099	8.814	<0.005	<0.05
BAG2	0.49	2.206	8.812	<0.005	<0.05
SLC35E3	-0.41	3.461	8.81	<0.005	<0.05
AMOTL1	-1.609	2.188	8.809	<0.005	<0.05
ZSCAN18	-0.657	3.38	8.809	<0.005	<0.05
STMN3	-0.488	4.391	8.804	<0.005	<0.05
NPM1P39	0.676	-0.498	8.803	<0.005	<0.05
FOXRED1	0.538	2.979	8.797	<0.005	<0.05
GRIN3A	0.672	0.845	8.796	<0.005	<0.05
ARHGEF40	-0.609	6.132	8.795	<0.005	<0.05
DENND1B	0.469	4.285	8.794	<0.005	<0.05
BMP2K	-0.525	5.823	8.782	<0.005	<0.05
FZR1	-0.352	5.294	8.779	<0.005	<0.05
ENSG00000282339	-0.87	5.655	8.776	<0.005	<0.05
MAPRE3	-0.567	2.311	8.755	<0.005	<0.05
EIF2S2	0.38	6.107	8.751	<0.005	<0.05
DGCR5	-0.871	0.217	8.751	<0.005	<0.05
PTPRN	-1.064	0.427	8.747	<0.005	<0.05
MAL	-0.573	4.252	8.745	<0.005	<0.05

BCORL1	-0.459	4.562	8.744	<0.005	<0.05
MINDY1	-0.543	6.81	8.735	<0.005	<0.05
RFFL	-0.537	6.151	8.729	<0.005	<0.05
LDAH	0.447	3.13	8.725	<0.005	<0.05
COPS5	0.288	4.958	8.721	<0.005	<0.05
PDE7B	-1.007	-0.472	8.717	<0.005	<0.05
NELL2	-0.597	5.081	8.712	<0.005	<0.05
FAM174A	-0.489	3.922	8.711	<0.005	<0.05
MAF1	-0.502	7.034	8.708	<0.005	<0.05
FAF1	0.335	4.348	8.707	<0.005	<0.05
GBP4	0.645	6.939	8.705	<0.005	<0.05
CX3CR1	0.575	8.299	8.697	<0.005	<0.05
CHMP1B	-0.355	6.629	8.69	<0.005	<0.05
COX6A1	0.521	6.342	8.674	<0.005	<0.05
CASC3	-0.48	7.442	8.673	<0.005	<0.05
TBC1D22B	-0.404	4.138	8.67	<0.005	<0.05
DNAJC7	0.354	5.812	8.669	<0.005	<0.05
THOP1	0.487	3.041	8.668	<0.005	<0.05
BEND3	0.657	0.084	8.666	<0.005	<0.05
MRPL9	0.39	4.435	8.666	<0.005	<0.05
POLR3K	0.49	3.045	8.665	<0.005	<0.05
HNRNPA1P8	0.557	0.219	8.66	<0.005	<0.05
PPA2	0.417	4.204	8.659	<0.005	<0.05
MRPL20	0.38	4.682	8.657	<0.005	<0.05
DDB2	0.399	4.106	8.655	<0.005	<0.05
FAM13A	-0.459	4.171	8.654	<0.005	<0.05
TUBBP1	0.712	-0.012	8.654	<0.005	<0.05
TSC22D3	-0.651	9.256	8.653	<0.005	<0.05
IL15	0.577	2.342	8.649	<0.005	<0.05
FFAR3	1.126	1.016	8.648	<0.005	<0.05

RETREG2	-0.455	7.057	8.641	<0.005	<0.05
MRPL33	0.377	4.111	8.635	<0.005	<0.05
TRAPPC14	-0.458	5.417	8.631	<0.005	<0.05
SIAH2	-0.765	6.292	8.626	<0.005	<0.05
ARHGAP9	-0.472	7.903	8.625	<0.005	<0.05
ATP2C1	0.273	5.203	8.624	<0.005	<0.05
ABRACL	0.372	5.253	8.622	<0.005	<0.05
BANP	-0.378	4.467	8.617	<0.005	<0.05
HDAC8	0.427	2.772	8.617	<0.005	<0.05
ENSG00000281938	-2.327	2.458	8.613	<0.005	<0.05
CLDN23	0.773	-0.167	8.612	<0.005	<0.05
IFITM3P2	1.05	1.407	8.61	<0.005	<0.05
IKBKE	0.357	4.787	8.608	<0.005	<0.05
SLC16A1	0.505	3.567	8.606	<0.005	<0.05
MEGF6	-0.703	4.524	8.604	<0.005	<0.05
CES1	0.938	4.62	8.603	<0.005	<0.05
PHLDA3	1.114	-0.766	8.602	<0.005	<0.05
RTRAF	0.381	5.781	8.601	<0.005	<0.05
ETFB	0.789	4.914	8.6	<0.005	<0.05
ABCC6	-0.842	2.015	8.598	<0.005	<0.05
LRP8	0.585	2.529	8.589	<0.005	<0.05
ST20-AS1	-0.572	2.99	8.586	<0.005	<0.05
BANF1P3	0.644	0.186	8.583	<0.005	<0.05
ZNG1DP	0.536	1.25	8.577	<0.005	<0.05
MBOAT1	-0.428	4.527	8.576	<0.005	<0.05
EIF2AK1	-0.595	7.441	8.575	<0.005	<0.05
DLC1	-1.616	-0.224	8.571	<0.005	<0.05
ENSG00000261468	-0.785	0.667	8.569	<0.005	<0.05
ZPR1	0.34	4.742	8.566	<0.005	<0.05
E2F2	0.628	4.169	8.562	<0.005	<0.05

APOL3	0.402	6.153	8.562	<0.005	<0.05
PDGFRB	0.906	2.957	8.56	<0.005	<0.05
SVIL	-0.55	6.762	8.559	<0.005	<0.05
GPX1	-0.736	8.786	8.552	<0.005	<0.05
PACSIN2	-0.501	7.059	8.531	<0.005	<0.05
ZHX1	0.312	4.369	8.524	<0.005	<0.05
GDI2P2	0.523	1.247	8.523	<0.005	<0.05
LINC02785	0.927	0.576	8.522	<0.005	<0.05
ENSG00000290018	-0.651	7.604	8.521	<0.005	<0.05
GOLM2	-0.339	6.277	8.514	<0.005	<0.05
EARS2	0.474	3.096	8.502	<0.005	<0.05
AP2A1	-0.346	6.652	8.5	<0.005	<0.05
TIFA	0.479	4.646	8.498	<0.005	<0.05
MARCHF7	-0.427	7.606	8.497	<0.005	<0.05
CYP4F3	-0.784	6.839	8.495	<0.005	<0.05
MRPS21	0.388	4.521	8.494	<0.005	<0.05
GSTO1	0.443	5.889	8.491	<0.005	<0.05
ENSG00000288882	-1.233	2.844	8.482	<0.005	<0.05
KANK2	-1.011	2.071	8.478	<0.005	<0.05
SRP68	0.365	5.344	8.469	<0.005	<0.05
RNF8	0.407	3.152	8.465	<0.005	<0.05
WDR12	0.417	3.189	8.463	<0.005	<0.05
UQCRHL	0.689	2.49	8.459	<0.005	<0.05
ZNF653	-0.518	2.093	8.455	<0.005	<0.05
IMMT	0.312	5.587	8.454	<0.005	<0.05
SHMT1	0.56	2.916	8.453	<0.005	<0.05
CLEC11A	0.675	1.304	8.453	<0.005	<0.05
IL6R	-0.527	9.023	8.448	<0.005	<0.05
REEP4	0.37	4.074	8.445	<0.005	<0.05
JAML	-0.485	9.325	8.443	<0.005	<0.05

AGER	-0.684	8.091	8.443	<0.005	<0.05
POT1	0.328	3.905	8.441	<0.005	<0.05
PIP4K2A	-0.438	8.007	8.44	<0.005	<0.05
ENSG00000288961	-0.782	-0.189	8.439	<0.005	<0.05
ITM2B	-0.531	10.549	8.429	<0.005	<0.05
NHS	-0.679	3.099	8.418	<0.005	<0.05
ZBTB44	-0.341	6.285	8.416	<0.005	<0.05
HSPA1A	-0.526	7.978	8.414	<0.005	<0.05
RXRA	-0.46	7.979	8.408	<0.005	<0.05
ILVBL	0.467	3.187	8.405	<0.005	<0.05
CNTNAP2	-1.126	1.306	8.401	<0.005	<0.05
ENSG00000283515	-1.244	2.337	8.395	<0.005	<0.05
LINC01765	-1.016	0.577	8.395	<0.005	<0.05
ENSG00000279453	-0.596	1.938	8.393	<0.005	<0.05
ACOT1	0.66	0.512	8.387	<0.005	<0.05
ERG28	0.424	3.623	8.386	<0.005	<0.05
VTA1	0.304	5.263	8.384	<0.005	<0.05
GTF2E2	0.314	4.296	8.384	<0.005	<0.05
GOLT1B	0.354	4.231	8.384	<0.005	<0.05
UBA5	0.494	3.792	8.379	<0.005	<0.05
MRPS15	0.45	3.949	8.378	<0.005	<0.05
CABIN1	-0.384	6.617	8.377	<0.005	<0.05
GPKOW	0.336	4.478	8.368	<0.005	<0.05
LINC02580	-0.604	1.585	8.366	<0.005	<0.05
INTS14	0.371	4.027	8.366	<0.005	<0.05
PDE6D	0.367	3.675	8.357	<0.005	<0.05
METTL6	0.399	2.686	8.353	<0.005	<0.05
PLPP3	0.835	-0.018	8.349	<0.005	<0.05
ENSG00000270210	-0.98	-0.462	8.347	<0.005	<0.05
NAE1	0.328	4.132	8.345	<0.005	<0.05

HIKESHI	0.393	3.526	8.343	<0.005	<0.05
FAM157D	-0.918	2.997	8.34	<0.005	<0.05
CIRBP	-0.403	6.688	8.33	<0.005	<0.05
MRPL11	0.489	4.098	8.329	<0.005	<0.05
PPFIA1	-0.337	5.395	8.324	<0.005	<0.05
SNX27	-0.464	6.127	8.323	<0.005	<0.05
KPNA4	-0.281	6.375	8.322	<0.005	<0.05
WBP2	-0.502	7.915	8.321	<0.005	<0.05
SNRPB2	0.395	5.11	8.316	<0.005	<0.05
CDK5	0.54	2.493	8.314	<0.005	<0.05
VHL	-0.268	6.009	8.311	<0.005	<0.05
PCID2	0.351	4.244	8.309	<0.005	<0.05
MSMO1	0.437	3.427	8.308	<0.005	<0.05
RBM34	0.377	4.617	8.308	<0.005	<0.05
LPAR2	-1.02	1.936	8.301	<0.005	<0.05
EPS15L1	-0.349	5.754	8.298	<0.005	<0.05
MED6	0.322	4.065	8.298	<0.005	<0.05
PSMD1	0.316	5.742	8.293	<0.005	<0.05
ENSG00000261553	1.25	2.029	8.291	<0.005	<0.05
GLUL	-0.521	9.418	8.291	<0.005	<0.05
EPPK1	-0.885	2.078	8.29	<0.005	<0.05
CD300C	0.532	3.836	8.288	<0.005	<0.05
PPP1R14B	0.486	4.061	8.287	<0.005	<0.05
ORAI2	-0.497	7.012	8.286	<0.005	<0.05
DUSP16	-0.38	5.238	8.286	<0.005	<0.05
RNF141	-0.49	6.385	8.279	<0.005	<0.05
MRS2	0.348	3.867	8.274	<0.005	<0.05
GPBAR1	0.524	4.331	8.271	<0.005	<0.05
SGTB	-0.367	4.851	8.269	<0.005	<0.05
ENSG00000284685	-0.642	1.272	8.268	<0.005	<0.05

SLC39A9	0.271	5.312	8.264	<0.005	<0.05
CYB561D2	0.385	3.617	8.257	<0.005	<0.05
UBE2O	-0.758	5.493	8.256	<0.005	<0.05
ATF4P3	0.657	0.66	8.255	<0.005	<0.05
BANF1	0.401	5.039	8.254	<0.005	<0.05
DMAC2	0.355	4.167	8.247	<0.005	<0.05
UCHL5	0.339	4.44	8.247	<0.005	<0.05
LMBRD1	-0.356	6.127	8.246	<0.005	<0.05
MRPL46	0.5	2.703	8.245	<0.005	<0.05
TMEM39A	0.391	4.056	8.241	<0.005	<0.05
RBBP7	0.328	5.508	8.237	<0.005	<0.05
DPEP3	-0.929	1.86	8.236	<0.005	<0.05
ENSG00000289690	-0.816	0.742	8.234	<0.005	<0.05
PSEN2	0.583	1.949	8.23	<0.005	<0.05
SLC9A7	-0.468	3.259	8.228	<0.005	<0.05
SNRPF	0.453	3.955	8.226	<0.005	<0.05
TMEM185B	-0.352	4.617	8.221	<0.005	<0.05
NCOA4	-0.647	9.966	8.217	<0.005	<0.05
GATA2	-0.59	4.301	8.216	<0.005	<0.05
METTL14	0.3	4.588	8.212	<0.005	<0.05
NUDT3	-0.329	6.654	8.209	<0.005	<0.05
STAT1	0.618	8.756	8.206	<0.005	<0.05
TMEM268	0.415	4.126	8.205	<0.005	<0.05
PTMAP9	0.587	0.218	8.204	<0.005	<0.05
TUBA1A	-0.493	8.267	8.203	<0.005	<0.05
GPS2	-0.442	5.369	8.2	<0.005	<0.05
FUCA2	0.44	4.391	8.199	<0.005	<0.05
PLPPR2	-0.63	5.942	8.199	<0.005	<0.05
ANKDD1A	-0.696	3.846	8.198	<0.005	<0.05
EOLA1	0.404	3.581	8.194	<0.005	<0.05

ENSG00000275993	0.652	1.934	8.192	<0.005	<0.05
COX17	0.476	3.847	8.189	<0.005	<0.05
GPN1	0.314	4.555	8.184	<0.005	<0.05
RAD23A	-0.441	5.835	8.184	<0.005	<0.05
MDK	0.979	0.269	8.182	<0.005	<0.05
TAF4	-0.317	4.539	8.176	<0.005	<0.05
CABLES1	0.913	-0.103	8.173	<0.005	<0.05
SEMA3F-AS1	-1.197	-0.047	8.168	<0.005	<0.05
NATD1	-0.554	6.334	8.165	<0.005	<0.05
BOLA1	0.464	1.876	8.163	<0.005	<0.05
ENSG00000261172	-1.03	6.066	8.163	<0.005	<0.05
GATC	0.36	5.11	8.159	<0.005	<0.05
U2	1.752	3.669	8.159	<0.005	<0.05
MTMR10	-0.386	6.333	8.154	<0.005	<0.05
MRPS36	0.401	3.571	8.152	<0.005	<0.05
DDX11L10	-1.299	3.002	8.151	<0.005	<0.05
ENSG00000289172	-1.187	6.785	8.149	<0.005	<0.05
CYB5R3	-0.41	6.512	8.144	<0.005	<0.05
PHF11	0.401	5.473	8.142	<0.005	<0.05
FAM157C	-0.929	5.836	8.138	<0.005	<0.05
ABHD17AP1	0.483	2.016	8.137	<0.005	<0.05
GFM2	0.375	3.787	8.135	<0.005	<0.05
EGFL7	0.783	2.117	8.13	<0.005	<0.05
CD55	-0.57	7.602	8.128	<0.005	<0.05
PTPA	0.378	5.773	8.127	<0.005	<0.05
RPLP0P9	0.531	4.27	8.119	<0.005	<0.05
CXCL16	-0.517	6.145	8.113	<0.005	<0.05
MTIF2	0.338	4.166	8.108	<0.005	<0.05
HDGFL3	-0.45	3.815	8.108	<0.005	<0.05
PNOC	0.775	1.765	8.107	<0.005	<0.05

TSTD2	0.504	3.257	8.1	<0.005	<0.05
PCMTD1	-0.354	6.152	8.1	<0.005	<0.05
GRSF1	0.278	5.564	8.093	<0.005	<0.05
MBOAT2	-0.614	5.197	8.089	<0.005	<0.05
UBL7	-0.404	5.404	8.087	<0.005	<0.05
NR1D2	-0.368	5.157	8.086	<0.005	<0.05
MAP2K3	-0.489	7.262	8.084	<0.005	<0.05
EPHA1	-0.586	2.706	8.081	<0.005	<0.05
MALAT1	-0.91	8.379	8.078	<0.005	<0.05
CYB5B	0.326	5.294	8.077	<0.005	<0.05
MPLKIP	0.364	3.592	8.073	<0.005	<0.05
GUSB	0.333	5.154	8.07	<0.005	<0.05
BNIP1	0.562	1.758	8.067	<0.005	<0.05
ENSG00000287632	-1.19	-0.071	8.063	<0.005	<0.05
PYGL	-0.666	8.046	8.061	<0.005	<0.05
PPIH	0.462	3.734	8.061	<0.005	<0.05
EIF2S2P4	0.551	1.154	8.06	<0.005	<0.05
CCDC86-AS1	-0.801	3.197	8.058	<0.005	<0.05
MEI1	0.545	3.327	8.058	<0.005	<0.05
CTLA4	0.734	2.137	8.051	<0.005	<0.05
MAPK1	-0.439	7.982	8.046	<0.005	<0.05
PBXIP1	-0.455	8.366	8.044	<0.005	<0.05
RASSF2	-0.507	9.411	8.043	<0.005	<0.05
LRRC57	-0.334	3.779	8.039	<0.005	<0.05
EIF1B-AS1	0.694	1.407	8.037	<0.005	<0.05
RNF44	-0.438	7.288	8.036	<0.005	<0.05
IL1RN	0.59	6.959	8.036	<0.005	<0.05
DUT	0.429	4.469	8.033	<0.005	<0.05
HSPA1L	-0.424	2.998	8.033	<0.005	<0.05
PARP6	0.346	4.662	8.03	<0.005	<0.05

BBC3	-0.398	4.759	8.029	<0.005	<0.05
ENSG00000227355	-0.749	1.586	8.029	<0.005	<0.05
MTRR	0.414	4.381	8.025	<0.005	<0.05
ENSG00000284956	-0.632	9	8.021	<0.005	<0.05
PSMB7	0.37	5.687	8.019	<0.005	<0.05
MTX2	0.529	2.279	8.019	<0.005	<0.05
LTV1	0.388	3.862	8.015	<0.005	<0.05
PROC	-1.145	-0.073	8.015	<0.005	<0.05
CEP41	0.437	2.425	8.014	<0.005	<0.05
GMDS	0.469	2.793	8.001	<0.005	<0.05
TMTC4	0.619	1.966	8.001	<0.005	<0.05
ENSG00000289278	-1.103	-0.246	7.995	<0.005	<0.05
ABCG2	-1.725	0.406	7.993	<0.005	<0.05
SOD2	-0.568	10.819	7.99	<0.005	<0.05
PAIP2B	-0.565	2.124	7.989	<0.005	<0.05
ENSG00000260729	1.173	4.281	7.987	<0.005	<0.05
POMGNT1	0.374	3.796	7.984	<0.005	<0.05
TPPP3	-0.787	2.666	7.982	<0.005	<0.05
CEP20	0.329	4.362	7.981	<0.005	<0.05
RUFY4	1.171	0.078	7.981	<0.005	<0.05
TAGAP	-0.465	8.54	7.981	<0.005	<0.05
RFC1	0.295	5.384	7.978	<0.005	<0.05
PTGER4	-0.358	5.61	7.977	<0.005	<0.05
SUCLG1	0.339	5.164	7.976	<0.005	<0.05
UBR7	0.268	4.737	7.973	<0.005	<0.05
WDR45	-0.399	6.222	7.971	<0.005	<0.05
KY	-0.568	1.613	7.969	<0.005	<0.05
MKNK2	-0.424	8.147	7.968	<0.005	<0.05
ZDHHC13	0.377	3.275	7.968	<0.005	<0.05
CSF3R	-0.584	10.82	7.957	<0.005	<0.05

ENO2	-0.406	3.83	7.956	<0.005	<0.05
POLDIP2	0.342	5.263	7.955	<0.005	<0.05
NRSN2	0.715	-0.065	7.948	<0.005	<0.05
CCDC117	0.322	4.927	7.947	<0.005	<0.05
GID4	-0.458	4.138	7.936	<0.005	<0.05
LINC03034	1.176	0.131	7.936	<0.005	<0.05
ERP44	0.346	6.399	7.935	<0.005	<0.05
SYNPO2	0.9	0.486	7.934	<0.005	<0.05
HACD3	0.358	4.204	7.93	<0.005	<0.05
GOLPH3L	0.385	3.715	7.919	<0.005	<0.05
GABPB1-AS1	-0.704	2.692	7.916	<0.005	<0.05
NEMP2	0.399	2.874	7.909	<0.005	<0.05
HAT1	0.322	4.923	7.908	<0.005	<0.05
BOP1	0.42	3.858	7.907	<0.005	<0.05
DBF4B	0.569	1.379	7.9	<0.005	<0.05
OLFML2B	0.828	-0.003	7.896	<0.005	<0.05
MMP25	-0.62	8.915	7.893	<0.005	<0.05
PARP1	0.385	6.688	7.89	<0.005	<0.05
LIN9	0.582	1.357	7.888	<0.005	<0.05
SLC38A5	-0.608	5.31	7.888	<0.005	<0.05
YOD1	-0.669	5.05	7.885	<0.005	<0.05
NOP58	0.361	5.09	7.884	<0.005	<0.05
SRPK2	-0.358	7.242	7.877	<0.005	<0.05
NDUFB9	0.421	5.389	7.875	<0.005	<0.05
CAND1	0.313	5.916	7.872	<0.005	<0.05
CCPG1	-0.502	7.604	7.865	<0.005	<0.05
RRP9	0.47	2.718	7.863	<0.005	<0.05
UFSP2	0.447	3.58	7.863	<0.005	<0.05
USP16	0.3	5.239	7.862	<0.005	<0.05
SNU13	0.357	5.79	7.86	<0.005	<0.05

JOSD2	0.511	2.886	7.856	<0.005	<0.05
ENSG00000278600	-0.88	2.943	7.856	<0.005	<0.05
ENSG00000285952	-0.802	3.589	7.853	<0.005	<0.05
TMLHEP1	0.663	-0.659	7.852	<0.005	<0.05
RECQL	0.375	5.284	7.852	<0.005	<0.05
STOML2	0.421	4.584	7.848	<0.005	<0.05
SMG8	0.309	3.841	7.843	<0.005	<0.05
KLF3	-0.379	7.462	7.841	<0.005	<0.05
ENSG00000289366	0.741	2.373	7.832	<0.005	<0.05
NQO2	-0.567	5.673	7.829	<0.005	<0.05
CCNDBP1	-0.55	6.905	7.826	<0.005	<0.05
ZNF737	-0.428	3.612	7.825	<0.005	<0.05
NDUFS7	0.362	4.445	7.82	<0.005	<0.05
MT-ND6	-0.656	10.513	7.818	<0.005	<0.05
EIF4A1P2	0.604	-0.375	7.816	<0.005	<0.05
RAD51C	0.566	2.53	7.815	<0.005	<0.05
FAM118B	0.378	3.095	7.814	<0.005	<0.05
SLC37A1	0.327	4.068	7.797	<0.005	<0.05
SNRPD2	0.437	6.025	7.796	<0.005	<0.05
CRLS1	0.371	3.948	7.795	<0.005	<0.05
VPS4B	-0.322	6.591	7.795	<0.005	<0.05
NINJ2	-0.549	4.307	7.794	<0.005	<0.05
TRA2B	0.314	6.343	7.793	<0.005	<0.05
MFAP1	0.291	4.958	7.79	<0.005	<0.05
TRGV2	0.725	1.678	7.789	<0.005	<0.05
SPINDOC	0.454	2.826	7.782	<0.005	<0.05
KPNA1	-0.327	6.072	7.779	<0.005	<0.05
UQCRC2	0.33	6.289	7.774	<0.005	<0.05
ZNF865	-0.372	4.988	7.773	<0.005	<0.05
VSIG1	-0.474	2.952	7.773	<0.005	<0.05

PHF13	-0.389	3.351	7.771	<0.005	<0.05
HES4	1.196	1.769	7.767	<0.005	<0.05
SRBD1	0.357	4.661	7.763	<0.005	<0.05
CCDC85B	0.402	4.708	7.759	<0.005	<0.05
MAGOHB	0.463	2.735	7.759	<0.005	<0.05
GPX4	-0.447	6.536	7.755	<0.005	<0.05
KCNIP2	-0.874	-0.292	7.751	<0.005	<0.05
FASTKD3	0.487	2.171	7.749	<0.005	<0.05
MRPL41	0.438	3.696	7.743	<0.005	<0.05
ZUP1	0.385	2.838	7.742	<0.005	<0.05
CCR1	0.597	7.687	7.736	<0.005	<0.05
WASHC1	-0.586	4.129	7.736	<0.005	<0.05
GPR180	0.542	2.677	7.734	<0.005	<0.05
HNRNPA1P54	0.551	-0.694	7.732	<0.005	<0.05
RB1	0.319	5.498	7.731	<0.005	<0.05
GTF2B	0.324	5.459	7.726	<0.005	<0.05
POLA2	0.546	2.775	7.725	<0.005	<0.05
BTN3A1	0.417	7.657	7.722	<0.005	<0.05
ITFG1	0.338	4.906	7.721	<0.005	<0.05
FUNDC2	-0.48	5.947	7.716	<0.005	<0.05
YBX1P10	-0.638	2.421	7.715	<0.005	<0.05
XRCC6P2	0.618	0.226	7.712	<0.005	<0.05
ID2	0.364	6.327	7.7	<0.05	<0.05
MRPL57	0.392	3.839	7.699	<0.05	<0.05
LYPD2	-1.489	0.31	7.693	<0.05	<0.05
MRPL23	0.505	4.024	7.692	<0.05	<0.05
DHX40	-0.291	5.043	7.681	<0.05	<0.05
PTPN22	0.368	5.241	7.672	<0.05	<0.05
LMTK3	-0.527	1.752	7.666	<0.05	<0.05
ENSG00000279267	-0.942	0.59	7.664	<0.05	<0.05

ENSG00000289768	1.35	4.294	7.654	<0.05	<0.05
TIMM21	0.499	2.509	7.653	<0.05	<0.05
PRIM1	0.608	2.728	7.652	<0.05	<0.05
TMEM256	0.5	3.199	7.648	<0.05	<0.05
MARCHF1	0.409	6.198	7.644	<0.05	<0.05
EFCAB6	0.543	1.847	7.637	<0.05	<0.05
H2AC20	0.732	0.522	7.633	<0.05	<0.05
NORAD	-0.374	7.874	7.632	<0.05	<0.05
HS3ST3B1	-0.407	3.85	7.63	<0.05	<0.05
CREB1	-0.34	6.694	7.628	<0.05	<0.05
NDUFS4	0.452	3.666	7.625	<0.05	<0.05
SEC14L5	-0.864	2.044	7.619	<0.05	<0.05
FARSA	0.378	4.57	7.619	<0.05	<0.05
SEC31A	0.295	6.399	7.618	<0.05	<0.05
ENSG00000287255	-1.037	1.046	7.614	<0.05	<0.05
MACROD1	0.59	0.109	7.613	<0.05	<0.05
SUSD6	-0.459	7.366	7.609	<0.05	<0.05
MYO1F	-0.453	9.845	7.609	<0.05	<0.05
LSM1	0.386	4.585	7.608	<0.05	<0.05
DYRK1B	-0.361	4.362	7.602	<0.05	<0.05
CENPO	0.813	2.112	7.602	<0.05	<0.05
ENSG00000293293	0.644	-0.561	7.6	<0.05	<0.05
YIPF5	0.285	4.807	7.596	<0.05	<0.05
ADHFE1	-0.944	1.368	7.592	<0.05	<0.05
GPR18	0.382	3.685	7.586	<0.05	<0.05
CD2AP	0.362	4.801	7.584	<0.05	<0.05
ENSG00000286760	0.837	-0.108	7.583	<0.05	<0.05
LINC00891	0.744	0.356	7.582	<0.05	<0.05
TMEM43	-0.373	6.903	7.58	<0.05	<0.05
ENSG00000274272	-0.87	5.635	7.579	<0.05	<0.05

ATG16L2	-0.835	7.96	7.577	<0.05	<0.05
NTHL1	0.509	2.098	7.576	<0.05	<0.05
MIR646HG	-0.82	1.762	7.574	<0.05	<0.05
RAB18	-0.329	6.171	7.569	<0.05	<0.05
CYP51A1	1.214	3.342	7.566	<0.05	<0.05
ZC3HC1	0.414	2.41	7.561	<0.05	<0.05
LAGE3	0.516	2.535	7.56	<0.05	<0.05
CCSAP	-0.318	4.651	7.553	<0.05	<0.05
TMEM119	1.02	0.814	7.553	<0.05	<0.05
ENSG00000289346	-0.502	2.699	7.551	<0.05	<0.05
ACOX1	-0.46	6.453	7.549	<0.05	<0.05
MICALL2	-0.68	0.585	7.548	<0.05	<0.05
GSK3B	-0.335	6.473	7.545	<0.05	<0.05
IPO4	0.952	2.723	7.537	<0.05	<0.05
MRTO4	0.455	3.605	7.534	<0.05	<0.05
MBOAT7	-0.532	8.812	7.53	<0.05	<0.05
ARF4	0.412	5.914	7.529	<0.05	<0.05
COL18A1	-0.637	5.944	7.528	<0.05	<0.05
IFNG	0.674	1.589	7.526	<0.05	<0.05
TBK1	0.272	5.482	7.525	<0.05	<0.05
MMP24OS	-0.413	5.322	7.522	<0.05	<0.05
RPL15P2	0.556	1.779	7.519	<0.05	<0.05
CNOT3	-0.28	5.655	7.519	<0.05	<0.05
CDK18	0.639	0.735	7.51	<0.05	<0.05
BMERB1	-0.657	2.661	7.507	<0.05	<0.05
SHLD2	0.324	4.455	7.503	<0.05	<0.05
XPOT	0.283	5.463	7.499	<0.05	<0.05
MRPL49	0.269	4.955	7.497	<0.05	<0.05
CHST7	-0.441	3.541	7.495	<0.05	<0.05
LRRC40	0.412	3.204	7.493	<0.05	<0.05

CDC23	0.333	4.021	7.49	<0.05	<0.05
TNRC6C	-0.384	5.49	7.478	<0.05	<0.05
NUAK2	-0.447	6.581	7.476	<0.05	<0.05
SMIM20	0.437	3.051	7.475	<0.05	<0.05
IDH3A	0.417	4.574	7.475	<0.05	<0.05
ENSG00000272501	-0.548	3.811	7.474	<0.05	<0.05
KIAA0319	-0.71	1.725	7.468	<0.05	<0.05
H4C12	0.644	0.077	7.468	<0.05	<0.05
E2F1	0.896	2.923	7.468	<0.05	<0.05
UBASH3B	0.284	5.239	7.468	<0.05	<0.05
SMIM15	0.353	4.305	7.461	<0.05	<0.05
TRAJ49	-1.119	2.462	7.46	<0.05	<0.05
FICD	0.496	2.187	7.458	<0.05	<0.05
NDUFB11	0.424	5.029	7.458	<0.05	<0.05
ATP2B1	-0.419	6.907	7.458	<0.05	<0.05
CBX3P9	0.665	-0.485	7.456	<0.05	<0.05
CNDP2	0.344	6.294	7.456	<0.05	<0.05
OSBPL8	-0.465	7.845	7.455	<0.05	<0.05
CCR8	-0.97	0.309	7.455	<0.05	<0.05
C1orf216	0.407	3.005	7.453	<0.05	<0.05
ATG9B	-0.596	1.713	7.452	<0.05	<0.05
HAUS8	0.414	2.515	7.452	<0.05	<0.05
SNX18	-0.363	6.72	7.448	<0.05	<0.05
CCT5	0.365	6.925	7.442	<0.05	<0.05
C1orf43	0.341	6.655	7.44	<0.05	<0.05
TXNDC17	0.406	4.049	7.439	<0.05	<0.05
CWF19L1	0.269	4.634	7.435	<0.05	<0.05
TSPAN7	-1.461	-0.165	7.433	<0.05	<0.05
SHFL	0.376	5.293	7.427	<0.05	<0.05
SLC35A5	0.325	4.725	7.426	<0.05	<0.05

THEMIS2	-0.406	8.085	7.42	<0.05	<0.05
MTND2P28	-0.698	9.785	7.419	<0.05	<0.05
TRIM39	-0.289	4.42	7.419	<0.05	<0.05
GBP5	0.636	8.74	7.417	<0.05	<0.05
COX18	0.401	3.095	7.413	<0.05	<0.05
RASSF3	-0.418	8.258	7.412	<0.05	<0.05
OMA1	1.287	1.7	7.408	<0.05	<0.05
CXCL6	-0.86	-0.392	7.405	<0.05	<0.05
PLA2G15	0.459	3.024	7.404	<0.05	<0.05
MARCHF6	-0.348	6.521	7.404	<0.05	<0.05
SLX9	0.435	3.11	7.402	<0.05	<0.05
ZNF79	0.359	3.279	7.394	<0.05	<0.05
DHFR	0.729	4.703	7.393	<0.05	<0.05
TATDN2	0.284	6.192	7.39	<0.05	<0.05
TAP2	0.49	7.241	7.375	<0.05	<0.05
RASGRP4	-0.495	7.157	7.375	<0.05	<0.05
MVB12A	0.459	4.052	7.374	<0.05	<0.05
AQP3	0.525	5.533	7.371	<0.05	<0.05
INTS12	0.365	3.392	7.37	<0.05	<0.05
AHSA1	0.269	5.578	7.369	<0.05	<0.05
TRAPPC2B	0.579	1.082	7.364	<0.05	<0.05
ATP5MF	0.403	5.576	7.363	<0.05	<0.05
UBE2E1	0.341	4.588	7.363	<0.05	<0.05
IARS1	0.349	5.148	7.362	<0.05	<0.05
NDUFA13	0.436	5.646	7.361	<0.05	<0.05
INPP4B	-0.488	5.151	7.358	<0.05	<0.05
ATP2A2	0.333	6.07	7.354	<0.05	<0.05
UBASH3A	-0.396	4.365	7.353	<0.05	<0.05
FKBP7	0.852	-0.542	7.351	<0.05	<0.05
POLR2L	0.452	4.585	7.351	<0.05	<0.05

CRACDL	-0.702	1.455	7.351	<0.05	<0.05
TBC1D30	-0.571	1.916	7.349	<0.05	<0.05
ENSG00000243273	0.959	0.998	7.348	<0.05	<0.05
UNC119	-0.346	5.917	7.345	<0.05	<0.05
ATOX1	0.327	4.112	7.343	<0.05	<0.05
ENSG00000289382	0.704	1.585	7.343	<0.05	<0.05
NIBAN1	-0.65	9.43	7.342	<0.05	<0.05
FAM177A1	-0.313	4.246	7.341	<0.05	<0.05
MIX23	0.557	2.089	7.337	<0.05	<0.05
SSB	0.312	5.601	7.336	<0.05	<0.05
TNFRSF10D	-0.56	1.826	7.334	<0.05	<0.05
KAT2B	-0.517	6.552	7.334	<0.05	<0.05
LRRC20	0.61	1.181	7.331	<0.05	<0.05
GABPB1-IT1	-0.494	3.421	7.329	<0.05	<0.05
SFXN4	0.556	2.12	7.327	<0.05	<0.05
PDHA1	0.318	4.658	7.326	<0.05	<0.05
ENSG00000279884	-0.726	3.668	7.326	<0.05	<0.05
RENBP	-0.909	2.476	7.323	<0.05	<0.05
DDX18	0.301	5.973	7.312	<0.05	<0.05
NGLY1	0.297	4.827	7.31	<0.05	<0.05
ENSG00000255508	-0.682	3.901	7.309	<0.05	<0.05
GNGT2	0.495	3.722	7.308	<0.05	<0.05
AHCTF1	-0.466	6.764	7.302	<0.05	<0.05
ADAM28	-0.521	3.819	7.296	<0.05	<0.05
LIMA1	0.386	3.816	7.296	<0.05	<0.05
CAPG	0.453	6.411	7.295	<0.05	<0.05
CLEC4A	0.469	5.608	7.295	<0.05	<0.05
SULF2	-0.565	7.396	7.292	<0.05	<0.05
SLC19A1	-0.45	6.084	7.291	<0.05	<0.05
HNRNPAB	0.349	5.701	7.286	<0.05	<0.05

ENSG00000284292	-1.673	2.961	7.285	<0.05	<0.05
CYB561D1	-0.342	4.693	7.285	<0.05	<0.05
RNASET2	-0.515	8.008	7.284	<0.05	<0.05
GAL3ST4	-0.669	1.328	7.276	<0.05	<0.05
LTB	-0.452	7.97	7.27	<0.05	<0.05
DHRS7	-0.408	6.68	7.27	<0.05	<0.05
IKBIP	-0.516	5.638	7.269	<0.05	<0.05
RNY1	1.575	4.819	7.267	<0.05	<0.05
CRTC2	-0.279	5.926	7.265	<0.05	<0.05
LY75	-0.503	6.307	7.265	<0.05	<0.05
TCF4	0.435	4.327	7.261	<0.05	<0.05
CBX1	-0.293	5.477	7.261	<0.05	<0.05
TSHZ3	-0.503	3.972	7.257	<0.05	<0.05
TTC38	0.606	5.081	7.257	<0.05	<0.05
GNPNAT1	0.408	3.267	7.255	<0.05	<0.05
KLRC3	1.079	3.757	7.254	<0.05	<0.05
TRAFD1	0.414	6.702	7.251	<0.05	<0.05
PCBP2	-0.41	8.77	7.244	<0.05	<0.05
H2BC9	0.865	4.239	7.235	<0.05	<0.05
MRPS14	0.346	3.787	7.229	<0.05	<0.05
TALDO1	-0.543	9.03	7.227	<0.05	<0.05
SLC30A9	0.297	4.547	7.226	<0.05	<0.05
NABP1	-0.647	6.846	7.226	<0.05	<0.05
EXOSC7	0.443	3.044	7.223	<0.05	<0.05
BRWD3	-0.489	6.149	7.222	<0.05	<0.05
EDEM1	0.459	6.388	7.221	<0.05	<0.05
AGO4	-0.486	7.236	7.221	<0.05	<0.05
DPEP2	-0.523	6.88	7.219	<0.05	<0.05
ATG5	0.289	4.504	7.217	<0.05	<0.05
IL13RA1	-0.599	7.678	7.216	<0.05	<0.05

MIS12	0.348	3.901	7.215	<0.05	<0.05
ENSG00000249806	-0.789	-0.668	7.202	<0.05	<0.05
CASP1	0.385	7.688	7.202	<0.05	<0.05
ATP8B4	0.401	3.634	7.198	<0.05	<0.05
MRPL24	0.42	3.843	7.197	<0.05	<0.05
TAGLN2	-0.56	10.188	7.196	<0.05	<0.05
MPV17L2	0.461	2.15	7.196	<0.05	<0.05
TRPV2	0.287	5.301	7.192	<0.05	<0.05
STRN4	-0.311	6.122	7.191	<0.05	<0.05
CD200	-0.609	1.842	7.19	<0.05	<0.05
MYO7B	-0.805	2.872	7.19	<0.05	<0.05
ENSG00000266709	0.835	0.352	7.189	<0.05	<0.05
PBDC1	0.399	3.647	7.188	<0.05	<0.05
RWDD2A	0.581	1.301	7.186	<0.05	<0.05
PNO1	0.421	3.093	7.186	<0.05	<0.05
LINC02979	-0.971	-0.613	7.183	<0.05	<0.05
MCCC2	0.347	4.259	7.181	<0.05	<0.05
SH3BGRL2	-0.772	5.453	7.178	<0.05	<0.05
REPS2	-0.602	5.564	7.176	<0.05	<0.05
ENSG00000268170	-1.136	0.905	7.174	<0.05	<0.05
SLC25A17	0.447	2.637	7.174	<0.05	<0.05
NUDT4	-0.398	5.496	7.17	<0.05	<0.05
AGTPBP1	-0.345	6.582	7.168	<0.05	<0.05
GNAS	-0.492	9.989	7.164	<0.05	<0.05
SLC8A1	-0.486	5.22	7.163	<0.05	<0.05
PSMC1P1	0.438	2.352	7.163	<0.05	<0.05
TYW1	0.335	3.714	7.162	<0.05	<0.05
ENSG00000227598	-0.879	1.888	7.16	<0.05	<0.05
NRGN	-0.676	8.609	7.157	<0.05	<0.05
SLCO3A1	-0.356	6.516	7.153	<0.05	<0.05

CRISPLD2	-0.649	6.803	7.146	<0.05	<0.05
ENSG00000290034	-0.769	3.084	7.144	<0.05	<0.05
LACTB2	0.434	2.27	7.14	<0.05	<0.05
PIWIL4	0.646	1.115	7.136	<0.05	<0.05
LAX1	0.398	5.229	7.131	<0.05	<0.05
ZNF740	0.295	6.639	7.131	<0.05	<0.05
ENSG00000279722	-0.637	0.397	7.127	<0.05	<0.05
SIRPG	-0.566	3.836	7.124	<0.05	<0.05
MFSD13A	0.478	2.388	7.121	<0.05	<0.05
DUSP3	0.356	5.254	7.121	<0.05	<0.05
ABCE1	0.321	5.159	7.118	<0.05	<0.05
LINC00926	-0.892	4.079	7.117	<0.05	<0.05
MRPS34	0.45	4.897	7.1	<0.05	<0.05
MSANTD7	0.325	3.595	7.097	<0.05	<0.05
NANS	0.333	5.064	7.093	<0.05	<0.05
ZDHHC7	-0.287	6.346	7.085	<0.05	<0.05
DDX21	0.303	6.415	7.084	<0.05	<0.05
HPS5	0.312	4.306	7.082	<0.05	<0.05
TBC1D2	0.345	4.558	7.073	<0.05	<0.05
ZNF92	0.349	4.166	7.073	<0.05	<0.05
TRNAU1AP	0.357	3.453	7.072	<0.05	<0.05
IL12RB2	0.693	2.528	7.071	<0.05	<0.05
MESD	0.328	5.049	7.071	<0.05	<0.05
RMDN3	0.362	3.562	7.06	<0.05	<0.05
NAXE	0.376	4.958	7.059	<0.05	<0.05
ENSG00000289130	-0.728	4.356	7.058	<0.05	<0.05
NUP107	0.329	4.204	7.055	<0.05	<0.05
LINC01036	-1.163	2.556	7.055	<0.05	<0.05
RBM45	0.415	2.383	7.054	<0.05	<0.05
MTCH1	-0.279	6.183	7.047	<0.05	<0.05

ENSG00000279744	-0.948	0.124	7.043	<0.05	<0.05
CHCHD1	0.397	3.772	7.042	<0.05	<0.05
CALCOCO1	-0.327	6.656	7.041	<0.05	<0.05
CCT6A	0.3	6.236	7.041	<0.05	<0.05
LINC01480	0.912	0.335	7.038	<0.05	<0.05
ADGRE5	-0.483	9.531	7.031	<0.05	<0.05
FANCM	0.577	1.814	7.028	<0.05	<0.05
ROGDI	-0.448	4.056	7.028	<0.05	<0.05
ENSG00000261471	-0.793	1.805	7.027	<0.05	<0.05
FARS2	0.44	2.668	7.027	<0.05	<0.05
TPMT	0.392	4.383	7.026	<0.05	<0.05
CENPL	0.476	1.902	7.023	<0.05	<0.05
CPM	-0.503	3.3	7.023	<0.05	<0.05
CYB5R4	-0.389	6.706	7.016	<0.05	<0.05
TMEM135	0.463	2.567	7.003	<0.05	<0.05
HMGN2P5	0.394	3.185	7.003	<0.05	<0.05
CHCHD2P2	0.811	-0.802	6.995	<0.05	<0.05
LINC01094	-0.754	1.819	6.991	<0.05	<0.05
HIF1AN	-0.249	5.909	6.991	<0.05	<0.05
ALS2CL	-0.869	2.056	6.984	<0.05	<0.05
GDAP1	0.516	1.578	6.984	<0.05	<0.05
BUB3	0.279	5.724	6.983	<0.05	<0.05
E2F5	0.535	3.56	6.982	<0.05	<0.05
ENSG00000264772	0.834	3.432	6.981	<0.05	<0.05
KAZN	-0.954	2.183	6.97	<0.05	<0.05
GZMA	0.494	6.807	6.967	<0.05	<0.05
MT-TL2	-0.675	5.019	6.966	<0.05	<0.05
CENPBD2P	-0.286	4.377	6.963	<0.05	<0.05
TMEM25	-0.474	1.557	6.962	<0.05	<0.05
NEK7	-0.341	6.457	6.961	<0.05	<0.05

PITPNC1	-0.301	5.637	6.961	<0.05	<0.05
TDG	0.333	4.449	6.959	<0.05	<0.05
TEF	-0.411	3.138	6.956	<0.05	<0.05
SNN	-0.441	7.641	6.955	<0.05	<0.05
ELOF1	-0.527	5.323	6.95	<0.05	<0.05
YARS2	0.409	2.527	6.947	<0.05	<0.05
QRSL1	0.357	3.768	6.947	<0.05	<0.05
ENSG00000261915	-1.218	2.396	6.944	<0.05	<0.05
SLC35E4	0.64	0.693	6.941	<0.05	<0.05

Table S6: Coexpression module M30 MCL subclustering genes - positively correlated with CHIKV acute patients and negatively correlated with chronic individuals.

Supplementary Table S6: Coexpression module M30 MCL subclustering genes - positively correlated with CHIKV acute patients and negatively correlated with chronic individuals.

Clusters	Genes Count	Protein Names
Cluster 1	51	ATP1B3, ATP5F1A, ATP5F1C, ATP5MC1, ATP5MC3, ATP5PD, ATP5PF, ATP5PO, CHCHD2, COA3, COX16, COX17, COX5A, COX6C, COX7A2, COX7B, COX7C, COX8A, CYC1, CYCS, EI24, FH, HSD17B10, IDH2, MTCH2, NDUFA13, NDUFA4, NDUFA8, NDUFA9, NDUFAB1, NDUFB2, NDUFB4, NDUFB7, NDUFB8, NDUFB9, NDUFC2, NDUFS3, NDUFS5, NDUFV2, ROMO1, SLIRP, SUCLG1, TIMM17A, TIMM8B, TMEM256, TOMM22, TOMM5, TXN2, UQCRFS1, UQCRQ, VDAC1
Cluster 2	30	EIF5A, GNL2, ISOC2, MRPL11, MRPL12, MRPL13, MRPL15, MRPL20, MRPL22, MRPL24, MRPL27, MRPL3, MRPL37, MRPL40, MRPL41, MRPL47, MRPL51, MRPL54, MRPL58, MRPL9, MRPS12, MRPS15, MRPS22, MRPS24, MRPS7, RPL26L1, RPS27L, SIL1, SSBP1, TMED3
Cluster 3	22	ALYREF, BANF1, CPSF3, ERH, ILF2, LSM2, LSM3, LSM7, PDCD5, PRMT1, RTRAF, SF3B5, SNRPB, SNRPB2, SNRPC, SNRPD1, SNRPD2, SNRPE, SNRPF, SNRPG, SNU13, TAF9
Cluster 4	20	ANAPC11, BCCIP, EIF2S2, PSMA2, PSMA4, PSMA5, PSMA7, PSMB1, PSMB2, PSMB5, PSMB6, PSMC2, PSMC3, PSMC4, PSMD1, PSMD8, SEM1, SUB1, UBE2N, UBE2S
Cluster 5	15	OSTC, PPIB, RPN2, SEC11C, SEC61A1, SEC61B, SEC61G, SPCS1, SPCS2, SRPRB, SSR3, SSR4, TMEM147, TMEM208, TMEM258
Cluster 6	9	CCT2, CCT3, CCT4, CCT5, CCT6A, CCT7, PFDN6, RAN, RANBP1
Cluster 7	8	AP1S1, ITM2C, JCHAIN, MZB1, PDIA5, PRDX4, TXNDC11, TXNDC5
Cluster 8	5	BOLA2, BOLA2B, CIAO2B, GLRX3, NFU1
Cluster 9	4	FABP5, GARS1, RARS1, TARS1
Cluster 10	4	CUTA, DNAJC7, HSPE1, PRDX1
Cluster 11	4	ATG101, COMMD1, HAUS1, WASHC3
Cluster 12	4	FEN1, KPNA2, SAE1, XRCC6
Cluster 13	4	CALR, DNAJB11, MANF, SDF2L1
Cluster 14	4	AK2, DCTPP1, DTYMK, RRM1
Cluster 15	3	ALG5, ALG8, MPDU1
Cluster 16	3	AIFM1, HAX1, PHB

Cluster 17	2	LGALS1, TMSB10
Cluster 18	2	PDIA3, STMN1
Cluster 19	2	FDPS, PPA1
Cluster 20	2	COPS4, COPS9

Table S7: Coexpression module M17 MCL subclustering genes - positively correlated with CHIKV acute patients and negatively correlated with chronic individuals.

Supplementary Table S7: Coexpression module M17 MCL subclustering genes - positively correlated with CHIKV acute patients and negatively correlated with chronic individuals.

**Clusters**	**Genes Count**	**Protein Names**
Cluster 1	17	CMPK2, DDX60, EPSTI1, HERC5, IFI44L, IFIH1, IFIT1, IFIT2, IFIT3, IFIT5, ISG15, MX1, OAS1, OAS2, OAS3, RSAD2, USP18

Table S8: Coexpression module M28 MCL subclustering genes - negatively correlated with acute patients and positively correlated with CHIKV chronic individuals.

Supplementary Table S8: Coexpression module M28 MCL subclustering genes - negatively correlated with acute patients and positively correlated with CHIKV chronic individuals.

Clusters	Genes Count	Protein Names
Cluster 1	17	ETS2, FOXO3, GSK3B, IL17RA, IL6R, IRS2, MAPK1, MCL1, NAMPT, PIK3CD, RAF1, SP1, STAT3, STAT5B, TGFBR2, UBXN2B, XPO6
Cluster 2	3	CD46, LILRB3, MMP25
Cluster 3	3	NUMB, RBPJ, TBL1X
Cluster 4	3	BCL6, CDK19, PELI1
Cluster 5	2	KDM4B, KDM6B
Cluster 6	2	CPD, IGF2R
Cluster 7	2	DYSF, RIPOR2
Cluster 8	2	KIF13A, NDEL1
Cluster 9	2	SEC14L1, SORL1
Cluster 10	2	CASC3, MSL1
Cluster 11	2	MINDY1, USP4
Cluster 12	2	ACOX1, ELOVL5
Cluster 13	2	OSBPL2, REPS2
Cluster 14	2	GAB2, TBC1D14
Cluster 15	2	MYO1F, RASGRP4

Table S9: Functional enrichments for the genes belonging to Module 30 - positively correlated with CHIKV acute patients and negatively correlated with chronic individuals.

Supplementary Table S9: Functional enrichments for the genes belonging to Module 30 - positively correlated with CHIKV acute patients and negatively correlated with chronic individuals.

	Description	Count in network	Strength	False discovery rate
**Gene Ontology**				
Biological Process	7-methylguanosine cap hypermethylation	6 of 8	1.85	<0.0001
	Positive regulation of establishment of protein localization to telomere	6 of 10	1.75	<0.0001
	Positive regulation of protein localization to Cajal body	6 of 11	1.71	<0.0001
	[2Fe-2S] cluster assembly	3 of 6	1.67	0.0094
	Positive regulation of telomerase RNA localization to Cajal bod	6 of 15	1.57	<0.0001
	Post-translational protein targeting to membrane, translocation	3 of 8	1.55	0.0175
	Mitochondrial electron transport, cytochrome c to oxygen	8 of 23	1.51	<0.0001
	U2-type prespliceosome assembly	8 of 24	1.5	<0.0001
	Mitochondrial electron transport, ubiquinol to cytochrome c	4 of 13	1.46	0.0028
	Protein maturation by iron-sulfur cluster transfer	5 of 17	1.44	0.00037
	Proton motive force-driven mitochondrial ATP synthesis	18 of 64	1.42	<0.0001
	Mitochondrial electron transport, NADH to ubiquinone	13 of 46	1.42	<0.0001
	Proton motive force-driven ATP synthesis	20 of 74	1.4	<0.0001
	Aerobic electron transport chain	23 of 87	1.39	<0.0001
	Mitochondrial ATP synthesis coupled electron transport	23 of 92	1.37	<0.0001
	Oxidative phosphorylation	29 of 122	1.35	<0.0001
	SRP-dependent cotranslational protein targeting to membrane	4 of 17	1.34	0.0065
	Cotranslational protein targeting to membrane	5 of 23	1.31	0.0012
	Purine ribonucleoside triphosphate biosynthetic process	21 of 98	1.3	<0.0001
	Mitochondrial respiratory chain complex I assembly	13 of 61	1.3	<0.0001

	Mitochondrial translation	23 of 112	1.28	<0.0001
	Spliceosomal snRNP assembly	8 of 39	1.28	<0.0001
	Aerobic respiration	32 of 161	1.27	<0.0001
Molecular Function	U1 snRNP binding	3 of 4	1.85	0.0074
	Proteasome-activating activity	3 of 6	1.67	0.0158
	Proton-transporting ATP synthase activity, rotational mechanism	3 of 9	1.5	0.0302
	NADH dehydrogenase activity	13 of 44	1.44	<0.0001
	NADH dehydrogenase (ubiquinone) activity	12 of 41	1.44	<0.0001
	U6 snRNA binding	4 of 14	1.43	0.0067
	Oxidoreduction-driven active transmembrane transporter activity	17 of 71	1.35	<0.0001
Cellular Component	Methylosome	8 of 12	1.8	<0.0001
	pICln-Sm protein complex	4 of 6	1.8	<0.0001
	Mitochondrial proton-transporting ATP synthase, stator stalk	2 of 3	1.8	0.0168
	Chaperonin-containing T-complex	6 of 10	1.75	<0.0001
	U4 snRNP	6 of 10	1.75	<0.0001

Table S10: Functional enrichments for the genes belonging to Module 17 - positively correlated with CHIKV acute patients and negatively correlated with chronic individuals.

Supplementary Table S10: Functional enrichments for the genes belonging to Module 17 - positively correlated with CHIKV acute patients and negatively correlated with chronic individuals.

	Description	Count in network	Strength	False discovery rate
**Gene Ontology**				
Biological Process	Negative regulation of IP-10 production	2 of 3	2.89	0.0024
	Negative regulation of chemokine (C-X-C motif) ligand 2 production	2 of 4	2.76	0.0034
	MDA-5 signaling pathway	2 of 4	2.76	0.0034
	interleukin-27-mediated signaling pathway	3 of 7	2.7	<0.0001
	ISG15-protein conjugation	2 of 6	2.59	0.0053
	Regulation of ribonuclease activity	3 of 10	2.54	<0.0001
	Cellular response to interferon-alpha	3 of 12	2.46	0.00013
	Negative regulation of type I interferon-mediated signaling pathway	4 of 19	2.39	<0.0001
	Negative regulation of viral genome replication	9 of 56	2.27	<0.0001
	Positive regulation of monocyte chemotactic protein-1 production	2 of 14	2.22	0.0201
	Antiviral innate immune response	3 of 22	2.2	0.00054
	Positive regulation of interferon-beta production	5 of 41	2.15	<0.0001
	Cellular response to exogenous dsRNA	2 of 19	2.09	0.0321
	Response to type I interferon	5 of 52	2.05	<0.0001
	Cellular response to type I interferon	3 of 44	1.9	0.0028
	Defense response to virus	14 of 252	1.81	<0.0001
	Positive regulation of tumor necrosis factor production	4 of 101	1.66	0.0007
	Cellular response to virus	3 of 87	1.6	0.0146
	Regulation of response to cytokine stimulus	5 of 163	1.55	0.00013
	Pattern recognition receptor signaling pathway	3 of 101	1.54	0.0218
	Regulation of response to biotic stimulus	7 of 361	1.35	<0.0001
	Innate immune response	13 of 754	1.3	<0.0001
	Positive regulation of cytokine production	6 of 482	1.16	0.0008

Molecular Function	2-5-oligoadenylate synthetase activity	3 of 4	2.94	<0.0001
	Double-stranded RNA binding	5 of 76	1.88	<0.0001
Reactome	OAS antiviral response	3 of 9	2.59	<0.0001
	Interferon alpha/beta signaling	11 of 71	2.25	<0.0001
	Antiviral mechanism by IFN-stimulated genes	8 of 82	2.05	<0.0001
	Negative regulators of DDX58/IFIH1 signaling	3 of 34	2.01	0.0012
	ISG15 antiviral mechanism	5 of 74	1.89	<0.0001
	Interferon Signaling	12 of 199	1.84	<0.0001
	Interferon gamma signaling	3 of 90	1.59	0.0150

Table S11: Functional enrichments for the genes belonging to Module 22 - negatively correlated with the control group, and positively correlated with CHIKV chronic.

Supplementary Table S11: Functional enrichments for the genes belonging to Module 22 - negatively correlated with the control group, and positively correlated with CHIKV chronic.

	Description	Count in network	Strength	False discovery rate
**Gene Ontology**				
Biological Process	Estabelishment of Sertoli cell barrier	2 of 3	2.47	0.0384
	Histone H3-K4 trimethylation	3 of 22	1.78	0.0214
	Histone H3-K4 methylation	6 of 46	1.76	<0.0001
	Peptidyl-lysine trimethylation	4 of 42	1.62	0.004
	Histone lysine methylation	7 of 77	1.6	<0.0001
Molecular Function	Histone methyltransferase activity (H3-K4 specific)	3 of 14	1.97	0.0363

Table S12: Functional enrichments for the genes belonging to Module 28- negatively correlated with acute patients and positively correlated with CHIKV chronic individuals.

Supplementary Table S12: Functional enrichments for the genes belonging to Module 28- negatively correlated with acute patients and positively correlated with CHIKV chronic individuals.

	Description	Count in network	Strength	False discovery rate
**Gene Ontology**				
Biological Process	Negative regulation of metalloendopeptidase activity involved in amyloid precursor protein catabolic process	2 of 2	2.38	0.0286
	Regulation of metalloendopeptidase activity	3 of 7	2.01	0.0085
	T-helper 17 type immune response	3 of 14	1.71	0.0182
	Positive regulation of regulatory T cell differentiation	3 of 23	1.49	0.0399
	T-helper 17 cell lineage commitment	2 of 7	2.1	0.0027
	Growth hormone receptor signaling pathway via JAK-STAT	2 of 10	2.75	0.0034
	Interleukin-6-mediated signaling pathway	1 of 14	2.6	0.0038
	Nuclear membrane organization	4 of 45	1.32	0.0184
	Membrane assembly	4 of 59	1.21	0.0359
	Positive regulation of neuron death	5 of 92	1.11	0.0186
	Extrinsic apoptotic signaling pathway	6 of 113	1.1	0.0089
	Positive regulation of T cell differentiation	5 of 116	1.01	0.0383
Cellular Component	Transcription repressor complex	5 of 77	1.19	0.0250
Subcellular Localization	interleukin-6 receptor complex	2 of 4	3.15	0.0037
Reactome	Negative feedback regulation of MAPK pathway	2 of 6	2.97	0.00086
	MAPK1 (ERK2) activation	2 of 9	2.8	0.0015
	Interleukin-9 signaling	2 of 9	2.8	0.0015
	Interleukin-21 signaling	2 of 10	2.75	0.0015
	Interleukin-6 signaling	2 of 11	2.71	0.0015
	Signaling by Leptin	3 of 11	2.71	0.0015
	Interleukin-15 signaling	3 of 14	2.6	0.0018
	Growth hormone receptor signaling	4 of 23	1.62	0.0036

Interleukin-7 signaling	3 of 25	1.45	0.0353
Interleukin-2 family signaling	4 of 44	1.33	0.0166
Signaling by CSF3 (G-CSF)	3 of 29	1.39	0.0410
Interleukin-4 and Interleukin-13 signaling	5 of 107	1.04	0.0207
